# Supplementary material for: Gas explosion characteristics and spray control mechanism in underground square
Source: PLoS One. 2024 Apr 24;19(4):e0293421. doi: 10.1371/journal.pone.0293421 (PMC11042696; doi:10.1371/journal.pone.0293421)
Supplement: S1 File — Green dots represent representative time nodes in different explosion stages. (PDF) [file pone.0293421.s001.pdf]

t /s	*P* /MPa
60.000	0.00332
60.000	0.00927
60.002	0.02421
60.005	0.01908
60.008	0.01626
60.010	0.01922
60.012	0.02228
60.014	0.02316
60.016	0.02319
60.018	0.02333
60.020	0.02372
60.022	0.0241
60.024	0.02434
60.026	0.02449
60.027	0.02459
60.029	0.02468
60.031	0.02475
60.033	0.0248
60.035	0.02483
60.036	0.02485
60.038	0.02486
60.040	0.02486
60.042	0.02485
60.044	0.02483
60.046	0.0248
60.047	0.02476
60.049	0.02472
60.051	0.02467
60.053	0.02462
60.055	0.02457
60.057	0.02451
60.058	0.02445
60.060	0.02438
60.062	0.02432
60.064	0.02425
60.066	0.02419
60.068	0.02412
60.070	0.02406
60.072	0.024
60.073	0.02393
60.075	0.02387
60.077	0.02381
60.079	0.02375
60.081	0.02369
60.083	0.02363
60.085	0.02357
60.087	0.02351
60.089	0.02345
60.091	0.0234
60.093	0.02334
60.095	0.02329
60.097	0.02323

60.098	0.02318
60.100	0.02312
60.102	0.02307
60.104	0.02302
60.106	0.02297
60.108	0.02291
60.110	0.02286
60.112	0.02281
60.114	0.02276
60.116	0.02271
60.118	0.02266
60.120	0.02261
60.122	0.02256
60.124	0.02251
60.126	0.02245
60.128	0.0224
60.130	0.02235
60.132	0.0223
60.134	0.02225
60.136	0.0222
60.138	0.02215
60.140	0.0221
60.142	0.02204
60.144	0.02199
60.146	0.02194
60.148	0.02189
60.151	0.02184
60.153	0.02178
60.155	0.02173
60.157	0.02168
60.159	0.02163
60.161	0.02157
60.163	0.02152
60.165	0.02147
60.167	0.02142
60.169	0.02136
60.171	0.02131
60.173	0.02126
60.175	0.02121
60.178	0.02115
60.180	0.0211
60.182	0.02105
60.184	0.021
60.186	0.02094
60.188	0.02089
60.190	0.02084
60.193	0.02079
60.195	0.02074
60.197	0.02069
60.199	0.02065
60.201	0.0206
60.203	0.02056
60.205	0.02051

60.208	0.02047
60.210	0.02043
60.212	0.02039
60.214	0.02035
60.216	0.02031
60.219	0.02027
60.221	0.02027
60.223	0.02037
60.225	0.02051
60.227	0.02068
60.229	0.02085
60.231	0.02103
60.234	0.02121
60.236	0.02139
60.238	0.02157
60.240	0.02176
60.242	0.02194
60.244	0.0221
60.245	0.02225
60.247	0.02239
60.249	0.02254
60.251	0.02266
60.253	0.02278
60.255	0.02289
60.257	0.02301
60.259	0.02312
60.261	0.02323
60.262	0.02334
60.264	0.02345
60.266	0.02354
60.268	0.02363
60.270	0.02372
60.271	0.02381
60.273	0.02389
60.275	0.02398
60.277	0.02406
60.279	0.02415
60.280	0.02423
60.282	0.02431
60.284	0.02439
60.285	0.02434
60.287	0.02434
60.289	0.02437
60.291	0.0243
60.292	0.02429
60.294	0.02432
60.296	0.02426
60.298	0.02425
60.299	0.02429
60.301	0.02423
60.303	0.02423
60.305	0.02429
60.306	0.02424

60.308	0.02426
60.310	0.02432
60.312	0.02428
60.313	0.02431
60.315	0.02425
60.317	0.02427
60.319	0.02421
60.320	0.02423
60.322	0.02418
60.324	0.02421
60.326	0.02416
60.327	0.0242
60.329	0.02416
60.331	0.02421
60.332	0.02418
60.334	0.0241
60.336	0.02413
60.338	0.02409
60.339	0.02415
60.341	0.02414
60.343	0.02408
60.345	0.02413
60.346	0.02412
60.348	0.02406
60.350	0.02413
60.351	0.02412
60.353	0.02408
60.355	0.02401
60.357	0.02407
60.358	0.02408
60.360	0.02404
60.362	0.02399
60.364	0.02407
60.365	0.02409
60.367	0.02407
60.369	0.02404
60.370	0.02399
60.372	0.02394
60.374	0.02404
60.376	0.02407
60.377	0.02416
60.379	0.02427
60.381	0.02441
60.382	0.02455
60.384	0.02471
60.386	0.02487
60.387	0.02503
60.389	0.02519
60.390	0.02536
60.392	0.02553
60.394	0.0257
60.395	0.02587
60.397	0.02604

60.398	0.0262
60.400	0.02636
60.401	0.02651
60.403	0.02663
60.404	0.02674
60.406	0.02682
60.407	0.02689
60.409	0.02696
60.410	0.02701
60.412	0.02705
60.413	0.02709
60.415	0.02712
60.416	0.02714
60.418	0.02717
60.419	0.02718
60.420	0.0272
60.422	0.02726
60.423	0.02732
60.425	0.02738
60.426	0.02744
60.428	0.02748
60.429	0.02751
60.431	0.02753
60.432	0.02755
60.434	0.02757
60.435	0.02758
60.436	0.02759
60.438	0.0276
60.439	0.02761
60.441	0.02761
60.442	0.02762
60.444	0.02762
60.445	0.02763
60.446	0.02763
60.448	0.02763
60.449	0.02763
60.451	0.02762
60.452	0.02762
60.454	0.02761
60.455	0.02762
60.456	0.02763
60.458	0.02765
60.459	0.02767
60.461	0.02768
60.462	0.02769
60.463	0.0277
60.465	0.02771
60.466	0.02772
60.468	0.02772
60.469	0.02773
60.471	0.02774
60.472	0.02775
60.473	0.02776

60.475	0.02776
60.476	0.02777
60.478	0.02777
60.479	0.02778
60.480	0.02778
60.482	0.02778
60.483	0.02778
60.485	0.02777
60.486	0.02778
60.487	0.02779
60.489	0.0278
60.490	0.02781
60.492	0.02782
60.493	0.02783
60.494	0.02784
60.496	0.02785
60.497	0.02786
60.498	0.02787
60.500	0.02788
60.501	0.02789
60.503	0.02789
60.504	0.0279
60.505	0.02789
60.507	0.02789
60.508	0.02789
60.510	0.02788
60.511	0.02788
60.512	0.02789
60.514	0.0279
60.515	0.0279
60.516	0.0279
60.518	0.0279
60.519	0.0279
60.521	0.02791
60.522	0.02791
60.523	0.02792
60.525	0.02792
60.526	0.02792
60.527	0.02791
60.529	0.02791
60.530	0.02791
60.531	0.02791
60.533	0.02791
60.534	0.02791
60.536	0.02791
60.537	0.02791
60.538	0.0279
60.540	0.0279
60.541	0.0279
60.542	0.02789
60.544	0.02789
60.545	0.02789
60.547	0.02789

60.548	0.02789
60.549	0.02789
60.551	0.02789
60.552	0.02789
60.553	0.02788
60.555	0.02788
60.556	0.02789
60.557	0.02789
60.559	0.02789
60.560	0.02789
60.562	0.02789
60.563	0.0279
60.564	0.0279
60.566	0.0279
60.567	0.02791
60.568	0.02792
60.570	0.02792
60.571	0.02793
60.572	0.02794
60.574	0.02795
60.575	0.02796
60.577	0.02797
60.578	0.02798
60.579	0.02799
60.581	0.02801
60.582	0.02803
60.583	0.02804
60.585	0.02806
60.586	0.02808
60.587	0.0281
60.589	0.02812
60.590	0.02815
60.592	0.02818
60.593	0.0282
60.594	0.02823
60.596	0.02827
60.597	0.0283
60.598	0.02834
60.600	0.02838
60.601	0.02842
60.602	0.02846
60.604	0.0285
60.605	0.02855
60.607	0.0286
60.608	0.02865
60.609	0.02871
60.611	0.02877
60.612	0.02882
60.613	0.02889
60.615	0.02895
60.616	0.02902
60.617	0.02909
60.619	0.02916

60.620	0.02924
60.622	0.02932
60.623	0.0294
60.624	0.02948
60.626	0.02957
60.627	0.02966
60.628	0.02975
60.630	0.02985
60.631	0.02994
60.632	0.03004
60.634	0.03014
60.635	0.03025
60.637	0.03035
60.638	0.03046
60.639	0.03057
60.641	0.03068
60.642	0.03079
60.643	0.03091
60.645	0.03103
60.646	0.03114
60.647	0.03126
60.649	0.03139
60.650	0.03151
60.651	0.03164
60.653	0.03176
60.654	0.03189
60.656	0.03202
60.657	0.03215
60.658	0.03229
60.660	0.03242
60.661	0.03256
60.662	0.0327
60.664	0.03284
60.665	0.03298
60.666	0.03313
60.668	0.03327
60.669	0.03342
60.670	0.03357
60.672	0.03372
60.673	0.03387
60.675	0.03402
60.676	0.03417
60.677	0.03433
60.679	0.03448
60.680	0.03464
60.681	0.03479
60.683	0.03495
60.684	0.03511
60.685	0.03526
60.687	0.03542
60.688	0.03558
60.689	0.03574
60.691	0.03589

60.692	0.03605
60.694	0.03621
60.695	0.03636
60.696	0.03652
60.698	0.03668
60.699	0.03683
60.700	0.03699
60.702	0.03715
60.703	0.0373
60.704	0.03746
60.706	0.03761
60.707	0.03777
60.708	0.03792
60.710	0.03808
60.711	0.03823
60.712	0.03838
60.714	0.03853
60.715	0.03868
60.717	0.03882
60.718	0.03899
60.719	0.0392
60.721	0.03945
60.722	0.03972
60.723	0.04
60.724	0.04029
60.726	0.04059
60.727	0.04089
60.728	0.04119
60.729	0.04149
60.731	0.0418
60.732	0.0421
60.733	0.04239
60.734	0.04269
60.736	0.04299
60.737	0.04329
60.738	0.04358
60.739	0.04388
60.740	0.04418
60.741	0.04447
60.743	0.04476
60.744	0.04506
60.745	0.04535
60.746	0.04564
60.747	0.04592
60.748	0.04621
60.749	0.04649
60.750	0.04676
60.751	0.04704
60.753	0.04731
60.754	0.04758
60.755	0.04784
60.756	0.04811
60.757	0.04837

60.758	0.04863
60.759	0.04888
60.760	0.04914
60.761	0.04939
60.762	0.04965
60.763	0.0499
60.764	0.05014
60.765	0.05039
60.766	0.05064
60.767	0.05089
60.768	0.05113
60.769	0.05138
60.770	0.05164
60.771	0.05191
60.772	0.05221
60.773	0.05251
60.774	0.05283
60.775	0.05316
60.776	0.0535
60.777	0.05384
60.778	0.0542
60.779	0.05456
60.780	0.05493
60.781	0.05531
60.781	0.05569
60.782	0.05609
60.783	0.05649
60.784	0.05689
60.785	0.05731
60.786	0.05774
60.787	0.05822
60.788	0.05871
60.788	0.05923
60.789	0.05977
60.790	0.06032
60.791	0.06088
60.791	0.06144
60.792	0.06201
60.793	0.06257
60.794	0.06314
60.794	0.0637
60.795	0.06426
60.796	0.06482
60.796	0.06538
60.797	0.06593
60.798	0.06648
60.798	0.06702
60.799	0.06756
60.800	0.06809
60.800	0.06862
60.801	0.06914
60.802	0.06966
60.802	0.07017

60.803	0.07067
60.804	0.07117
60.804	0.07166
60.805	0.07215
60.806	0.07263
60.806	0.0731
60.807	0.07356
60.808	0.07402
60.808	0.07447
60.809	0.07491
60.809	0.07534
60.810	0.07577
60.811	0.07621
60.811	0.07666
60.812	0.07711
60.813	0.07757
60.813	0.07803
60.814	0.07849
60.814	0.07895
60.815	0.07941
60.816	0.07988
60.816	0.08034
60.817	0.0808
60.817	0.08126
60.818	0.08172
60.819	0.08217
60.819	0.08262
60.820	0.08308
60.820	0.08353
60.821	0.08397
60.821	0.08442
60.822	0.08487
60.822	0.08531
60.823	0.08576
60.823	0.0862
60.824	0.08665
60.824	0.0871
60.825	0.08755
60.825	0.08801
60.826	0.08847
60.826	0.08893
60.827	0.08939
60.827	0.08987
60.828	0.09034
60.828	0.09083
60.829	0.09133
60.829	0.09183
60.829	0.09234
60.830	0.09285
60.830	0.09337
60.831	0.0939
60.831	0.09443
60.831	0.09496

60.832	0.09549
60.832	0.096
60.832	0.09651
60.833	0.09701
60.833	0.0975
60.833	0.09798
60.834	0.09844
60.834	0.0989
60.834	0.09934
60.835	0.09978
60.835	0.10021
60.836	0.10064
60.836	0.10107
60.836	0.10149
60.837	0.10191
60.837	0.10232
60.837	0.10275
60.838	0.10319
60.838	0.10364
60.838	0.10409
60.839	0.10453
60.839	0.10498
60.839	0.10544
60.840	0.10589
60.840	0.10634
60.840	0.1068
60.841	0.10725
60.841	0.1077
60.841	0.10815
60.841	0.1086
60.842	0.10904
60.842	0.10948
60.842	0.10992
60.843	0.11036
60.843	0.11079
60.843	0.11122
60.843	0.11165
60.844	0.11208
60.844	0.1125
60.844	0.11292
60.844	0.11333
60.845	0.11375
60.845	0.11416
60.845	0.11457
60.845	0.11498
60.846	0.11538
60.846	0.11576
60.846	0.11614
60.846	0.1165
60.846	0.11687
60.846	0.11724
60.847	0.1176
60.847	0.11797

60.847	0.11834
60.847	0.11871
60.847	0.11908
60.847	0.11945
60.848	0.11982
60.848	0.12019
60.848	0.12055
60.848	0.12091
60.848	0.12127
60.848	0.12163
60.849	0.12198
60.849	0.12234
60.849	0.12269
60.849	0.12304
60.849	0.12338
60.850	0.12373
60.850	0.12407
60.850	0.12442
60.850	0.12476
60.850	0.1251
60.850	0.12543
60.851	0.12577
60.851	0.1261
60.851	0.12644
60.851	0.12677
60.851	0.1271
60.851	0.12742
60.852	0.12775
60.852	0.12807
60.852	0.1284
60.852	0.12872
60.852	0.12904
60.852	0.12936
60.853	0.12968
60.853	0.12999
60.853	0.13031
60.853	0.13062
60.853	0.13094
60.853	0.13125
60.853	0.13156
60.854	0.13187
60.854	0.13218
60.854	0.13248
60.854	0.13279
60.854	0.13309
60.855	0.13339
60.855	0.13369
60.855	0.13399
60.855	0.13428
60.855	0.13458
60.855	0.13487
60.856	0.13516
60.856	0.13545

60.856	0.13573
60.856	0.13602
60.856	0.1363
60.856	0.13658
60.857	0.13686
60.857	0.13714
60.857	0.13742
60.857	0.1377
60.857	0.13798
60.857	0.13825
60.857	0.13853
60.858	0.1388
60.858	0.13907
60.858	0.13934
60.858	0.13961
60.858	0.13988
60.858	0.14014
60.859	0.14041
60.859	0.14067
60.859	0.14093
60.859	0.14119
60.859	0.14145
60.859	0.14171
60.860	0.14196
60.860	0.14222
60.860	0.14247
60.860	0.14273
60.860	0.14298
60.860	0.14323
60.860	0.14348
60.861	0.14373
60.861	0.14398
60.861	0.14423
60.861	0.14447
60.861	0.14472
60.861	0.14496
60.862	0.1452
60.862	0.14544
60.862	0.14568
60.862	0.14592
60.862	0.14616
60.862	0.1464
60.862	0.14664
60.863	0.14687
60.863	0.14711
60.863	0.14734
60.863	0.14758
60.863	0.14781
60.863	0.14804
60.864	0.14827
60.864	0.1485
60.864	0.14873
60.864	0.14896

60.864	0.14918
60.864	0.14941
60.864	0.14963
60.865	0.14986
60.865	0.15008
60.865	0.15031
60.865	0.15053
60.865	0.15075
60.865	0.15097
60.866	0.1512
60.866	0.15142
60.866	0.15164
60.866	0.15186
60.866	0.15208
60.866	0.1523
60.867	0.15253
60.867	0.15275
60.867	0.15297
60.867	0.15318
60.867	0.1534
60.867	0.15362
60.867	0.15384
60.868	0.15406
60.868	0.15428
60.868	0.15449
60.868	0.15471
60.868	0.15493
60.868	0.15514
60.869	0.15536
60.869	0.15557
60.869	0.15579
60.869	0.156
60.869	0.15621
60.869	0.15643
60.869	0.15664
60.870	0.15685
60.870	0.15707
60.870	0.15728
60.870	0.15749
60.870	0.1577
60.870	0.15792
60.870	0.15813
60.871	0.15834
60.871	0.15855
60.871	0.15876
60.871	0.15897
60.871	0.15918
60.871	0.15939
60.872	0.1596
60.872	0.15981
60.872	0.16002
60.872	0.16023
60.872	0.16044

60.872	0.16065
60.872	0.16085
60.873	0.16106
60.873	0.16127
60.873	0.16147
60.873	0.16168
60.873	0.16188
60.873	0.16209
60.873	0.16229
60.874	0.1625
60.874	0.1627
60.874	0.16291
60.874	0.16311
60.874	0.16331
60.874	0.16351
60.875	0.16372
60.875	0.16392
60.875	0.16412
60.875	0.16432
60.875	0.16452
60.875	0.16473
60.875	0.16493
60.876	0.16513
60.876	0.16533
60.876	0.16553
60.876	0.16573
60.876	0.16593
60.876	0.16613
60.876	0.16633
60.877	0.16653
60.877	0.16672
60.877	0.16692
60.877	0.16712
60.877	0.16731
60.877	0.16751
60.877	0.1677
60.878	0.1679
60.878	0.16809
60.878	0.16829
60.878	0.16848
60.878	0.16868
60.878	0.16887
60.878	0.16906
60.879	0.16925
60.879	0.16944
60.879	0.16964
60.879	0.16983
60.879	0.17002
60.879	0.1702
60.879	0.17039
60.880	0.17058
60.880	0.17077
60.880	0.17096

60.880	0.17114
60.880	0.17133
60.880	0.17152
60.880	0.1717
60.881	0.17189
60.881	0.17208
60.881	0.17226
60.881	0.17245
60.881	0.17263
60.881	0.17281
60.881	0.173
60.882	0.17318
60.882	0.17336
60.882	0.17354
60.882	0.17373
60.882	0.17391
60.882	0.17409
60.882	0.17427
60.883	0.17445
60.883	0.17463
60.883	0.17481
60.883	0.17499
60.883	0.17517
60.883	0.17535
60.883	0.17552
60.884	0.1757
60.884	0.17588
60.884	0.17606
60.884	0.17623
60.884	0.17641
60.884	0.17658
60.884	0.17676
60.884	0.17694
60.885	0.17711
60.885	0.17729
60.885	0.17746
60.885	0.17763
60.885	0.17781
60.885	0.17798
60.885	0.17815
60.886	0.17832
60.886	0.1785
60.886	0.17867
60.886	0.17884
60.886	0.17901
60.886	0.17918
60.886	0.17935
60.887	0.17952
60.887	0.17969
60.887	0.17986
60.887	0.18002
60.887	0.18019
60.887	0.18036

60.887	0.18052
60.888	0.18069
60.888	0.18085
60.888	0.18101
60.888	0.18118
60.888	0.18134
60.888	0.1815
60.888	0.18167
60.889	0.18183
60.889	0.18199
60.889	0.18215
60.889	0.18231
60.889	0.18247
60.889	0.18263
60.889	0.18279
60.890	0.18295
60.890	0.18311
60.890	0.18326
60.890	0.18342
60.890	0.18358
60.890	0.18373
60.890	0.18388
60.891	0.18404
60.891	0.18419
60.891	0.18434
60.891	0.1845
60.891	0.18465
60.891	0.1848
60.891	0.18495
60.892	0.1851
60.892	0.18525
60.892	0.1854
60.892	0.18555
60.892	0.1857
60.892	0.18584
60.892	0.18599
60.892	0.18614
60.893	0.18629
60.893	0.18643
60.893	0.18658
60.893	0.18673
60.893	0.18687
60.893	0.18702
60.893	0.18716
60.894	0.1873
60.894	0.18745
60.894	0.18759
60.894	0.18773
60.894	0.18787
60.894	0.18801
60.894	0.18815
60.895	0.18829
60.895	0.18843

60.895	0.18857
60.895	0.18871
60.895	0.18885
60.895	0.18899
60.895	0.18913
60.896	0.18927
60.896	0.18941
60.896	0.18954
60.896	0.18968
60.896	0.18982
60.896	0.18995
60.896	0.19009
60.897	0.19022
60.897	0.19036
60.897	0.19049
60.897	0.19063
60.897	0.19076
60.897	0.19089
60.897	0.19102
60.897	0.19116
60.898	0.19129
60.898	0.19142
60.898	0.19155
60.898	0.19168
60.898	0.19181
60.898	0.19194
60.898	0.19206
60.899	0.19219
60.899	0.19232
60.899	0.19245
60.899	0.19257
60.899	0.1927
60.899	0.19282
60.899	0.19295
60.900	0.19307
60.900	0.19319
60.900	0.19332
60.900	0.19344
60.900	0.19356
60.900	0.19368
60.900	0.1938
60.900	0.19392
60.901	0.19404
60.901	0.19416
60.901	0.19428
60.901	0.1944
60.901	0.19452
60.901	0.19464
60.901	0.19476
60.902	0.19488
60.902	0.19499
60.902	0.19511
60.902	0.19523

60.902	0.19534
60.902	0.19546
60.902	0.19558
60.903	0.19569
60.903	0.1958
60.903	0.19592
60.903	0.19603
60.903	0.19614
60.903	0.19626
60.903	0.19637
60.903	0.19648
60.904	0.19659
60.904	0.1967
60.904	0.19681
60.904	0.19692
60.904	0.19703
60.904	0.19714
60.904	0.19725
60.905	0.19736
60.905	0.19746
60.905	0.19757
60.905	0.19768
60.905	0.19778
60.905	0.19789
60.905	0.198
60.906	0.1981
60.906	0.19821
60.906	0.19831
60.906	0.19841
60.906	0.19852
60.906	0.19862
60.906	0.19872
60.906	0.19882
60.907	0.19892
60.907	0.19903
60.907	0.19913
60.907	0.19923
60.907	0.19933
60.907	0.19943
60.907	0.19953
60.908	0.19963
60.908	0.19972
60.908	0.19982
60.908	0.19992
60.908	0.20002
60.908	0.20012
60.908	0.20022
60.909	0.20032
60.909	0.20041
60.909	0.20051
60.909	0.20061
60.909	0.2007
60.909	0.2008

60.909	0.2009
60.909	0.20099
60.910	0.20109
60.910	0.20118
60.910	0.20128
60.910	0.20137
60.910	0.20147
60.910	0.20156
60.910	0.20166
60.911	0.20175
60.911	0.20184
60.911	0.20194
60.911	0.20203
60.911	0.20212
60.911	0.20221
60.911	0.20231
60.912	0.2024
60.912	0.20249
60.912	0.20258
60.912	0.20267
60.912	0.20276
60.912	0.20285
60.912	0.20294
60.912	0.20303
60.913	0.20312
60.913	0.20321
60.913	0.2033
60.913	0.20339
60.913	0.20348
60.913	0.20357
60.913	0.20366
60.914	0.20374
60.914	0.20383
60.914	0.20392
60.914	0.204
60.914	0.20409
60.914	0.20418
60.914	0.20426
60.915	0.20435
60.915	0.20443
60.915	0.20452
60.915	0.2046
60.915	0.20469
60.915	0.20477
60.915	0.20485
60.915	0.20494
60.916	0.20502
60.916	0.2051
60.916	0.20518
60.916	0.20527
60.916	0.20535
60.916	0.20543
60.916	0.20551

60.917	0.20559
60.917	0.20567
60.917	0.20575
60.917	0.20583
60.917	0.20591
60.917	0.20599
60.917	0.20607
60.918	0.20614
60.918	0.20622
60.918	0.20777
60.918	0.20973
60.918	0.21159
60.918	0.21335
60.918	0.21501
60.918	0.21656
60.919	0.218
60.919	0.21934
60.919	0.22058
60.919	0.22171
60.919	0.22274
60.919	0.22366
60.919	0.22449
60.920	0.22521
60.920	0.22584
60.920	0.22638
60.920	0.22683
60.920	0.22718
60.920	0.22746
60.920	0.22764
60.921	0.22775
60.921	0.22777
60.921	0.22771
60.921	0.22758
60.921	0.22739
60.921	0.22713
60.921	0.22679
60.921	0.22636
60.922	0.22583
60.922	0.2252
60.922	0.22446
60.922	0.22361
60.922	0.22263
60.922	0.22152
60.922	0.22027
60.923	0.21889
60.923	0.21735
60.923	0.21566
60.923	0.21382
60.923	0.21183
60.923	0.20969
60.923	0.20911
60.924	0.20917
60.924	0.20923

60.924	0.20929
60.924	0.20935
60.924	0.20941
60.924	0.20947
60.924	0.20953
60.924	0.20959
60.925	0.20965
60.925	0.20971
60.925	0.20977
60.925	0.20983
60.925	0.20989
60.925	0.20994
60.925	0.21
60.926	0.21006
60.926	0.21012
60.926	0.21132
60.926	0.21264
60.926	0.21389
60.926	0.21507
60.926	0.21616
60.927	0.21717
60.927	0.21808
60.927	0.21888
60.927	0.21957
60.927	0.22014
60.927	0.22059
60.927	0.22091
60.927	0.22111
60.928	0.22118
60.928	0.22112
60.928	0.22092
60.928	0.22059
60.928	0.22012
60.928	0.21952
60.928	0.21878
60.929	0.21792
60.929	0.21692
60.929	0.21581
60.929	0.21459
60.929	0.21325
60.929	0.2118
60.929	0.21158
60.930	0.21164
60.930	0.21169
60.930	0.21174
60.930	0.21179
60.930	0.21184
60.930	0.21189
60.930	0.21194
60.931	0.21199
60.931	0.21204
60.931	0.21209
60.931	0.21214

60.931	0.21219
60.931	0.21224
60.931	0.21229
60.931	0.21234
60.932	0.21239
60.932	0.21244
60.932	0.21249
60.932	0.21357
60.932	0.21503
60.932	0.21649
60.932	0.21794
60.933	0.21936
60.933	0.22076
60.933	0.22215
60.933	0.2235
60.933	0.22478
60.933	0.226
60.933	0.22713
60.934	0.22817
60.934	0.22909
60.934	0.2299
60.934	0.23058
60.934	0.23113
60.934	0.23153
60.934	0.23208
60.934	0.23246
60.935	0.23266
60.935	0.23264
60.935	0.23241
60.935	0.23199
60.935	0.23156
60.935	0.23093
60.935	0.23015
60.936	0.23077
60.936	0.23191
60.936	0.23302
60.936	0.23411
60.936	0.2352
60.936	0.23627
60.936	0.23728
60.937	0.23822
60.937	0.23908
60.937	0.23987
60.937	0.24057
60.937	0.24118
60.937	0.24168
60.937	0.24207
60.938	0.24234
60.938	0.24248
60.938	0.24247
60.938	0.24236
60.938	0.24204
60.938	0.24146

60.938	0.24059
60.938	0.2394
60.939	0.2383
60.939	0.23708
60.939	0.23558
60.939	0.23379
60.939	0.23243
60.939	0.23084
60.939	0.22902
60.940	0.22702
60.940	0.22489
60.940	0.22273
60.940	0.22151
60.940	0.22041
60.940	0.2193
60.940	0.21808
60.941	0.21674
60.941	0.21554
60.941	0.21559
60.941	0.21563
60.941	0.21567
60.941	0.21572
60.941	0.21705
60.942	0.21872
60.942	0.22036
60.942	0.22197
60.942	0.22354
60.942	0.22505
60.942	0.22648
60.942	0.22783
60.942	0.22908
60.943	0.23022
60.943	0.23125
60.943	0.23215
60.943	0.23293
60.943	0.23357
60.943	0.23408
60.943	0.23448
60.944	0.23474
60.944	0.23488
60.944	0.23488
60.944	0.23476
60.944	0.23452
60.944	0.23416
60.944	0.23369
60.945	0.23316
60.945	0.23256
60.945	0.23187
60.945	0.23109
60.945	0.23025
60.945	0.22939
60.945	0.22846
60.946	0.22749

60.946	0.22649
60.946	0.22544
60.946	0.22442
60.946	0.22333
60.946	0.22526
60.946	0.22713
60.946	0.22892
60.947	0.23061
60.947	0.23217
60.947	0.23358
60.947	0.23483
60.947	0.2359
60.947	0.23676
60.947	0.23741
60.948	0.23785
60.948	0.23809
60.948	0.23811
60.948	0.23785
60.948	0.23727
60.948	0.23634
60.948	0.23518
60.949	0.23385
60.949	0.23234
60.949	0.23086
60.949	0.22949
60.949	0.22798
60.949	0.2262
60.949	0.22398
60.950	0.22387
60.950	0.22553
60.950	0.22723
60.950	0.22895
60.950	0.23067
60.950	0.23235
60.950	0.23397
60.950	0.23551
60.951	0.23694
60.951	0.23823
60.951	0.23937
60.951	0.24031
60.951	0.24105
60.951	0.2416
60.951	0.24203
60.952	0.2423
60.952	0.24241
60.952	0.24232
60.952	0.24196
60.952	0.24131
60.952	0.24032
60.952	0.23897
60.953	0.23734
60.953	0.23598
60.953	0.23435

60.953	0.23244
60.953	0.23025
60.953	0.22968
60.953	0.22909
60.953	0.22848
60.954	0.22777
60.954	0.22691
60.954	0.22589
60.954	0.2247
60.954	0.22348
60.954	0.22232
60.954	0.22104
60.955	0.21963
60.955	0.21808
60.955	0.21808
60.955	0.21809
60.955	0.21809
60.955	0.21809
60.955	0.2181
60.956	0.2181
60.956	0.2181
60.956	0.21811
60.956	0.21811
60.956	0.21811
60.956	0.21811
60.956	0.21811
60.957	0.21812
60.957	0.21812
60.957	0.21812
60.957	0.21812
60.957	0.21812
60.957	0.21812
60.957	0.21844
60.957	0.21901
60.958	0.2195
60.958	0.21992
60.958	0.22026
60.958	0.22052
60.958	0.22071
60.958	0.22083
60.958	0.22088
60.959	0.22087
60.959	0.22082
60.959	0.22072
60.959	0.22056
60.959	0.22036
60.959	0.22026
60.959	0.22023
60.960	0.22013
60.960	0.21997
60.960	0.21975
60.960	0.21947
60.960	0.21914

60.960	0.21876
60.960	0.21837
60.961	0.21891
60.961	0.21936
60.961	0.21969
60.961	0.21991
60.961	0.22001
60.961	0.22
60.961	0.21988
60.962	0.21965
60.962	0.21932
60.962	0.2189
60.962	0.21838
60.962	0.21812
60.962	0.2182
60.962	0.21841
60.962	0.21862
60.963	0.21879
60.963	0.21891
60.963	0.21897
60.963	0.21896
60.963	0.21892
60.963	0.21882
60.963	0.21866
60.964	0.2184
60.964	0.21814
60.964	0.21815
60.964	0.21815
60.964	0.21816
60.964	0.21816
60.964	0.21816
60.965	0.21817
60.965	0.21817
60.965	0.21818
60.965	0.21818
60.965	0.21818
60.965	0.21819
60.965	0.21819
60.966	0.2182
60.966	0.2182
60.966	0.21821
60.966	0.21821
60.966	0.21822
60.966	0.21823
60.966	0.21823
60.966	0.21824
60.967	0.21824
60.967	0.21825
60.967	0.21826
60.967	0.21826
60.967	0.21827
60.967	0.21827
60.967	0.21828

60.968	0.21829
60.968	0.21829
60.968	0.2183
60.968	0.21831
60.968	0.21831
60.968	0.21832
60.968	0.21833
60.969	0.21833
60.969	0.21834
60.969	0.21834
60.969	0.21835
60.969	0.21836
60.969	0.21836
60.969	0.21837
60.970	0.21838
60.970	0.21838
60.970	0.21839
60.970	0.21839
60.970	0.2184
60.970	0.21841
60.970	0.21841
60.970	0.21842
60.971	0.21842
60.971	0.21843
60.971	0.21843
60.971	0.21844
60.971	0.21844
60.971	0.21845
60.971	0.21845
60.972	0.21846
60.972	0.21846
60.972	0.21847
60.972	0.21847
60.972	0.21848
60.972	0.21848
60.972	0.21849
60.973	0.21849
60.973	0.21849
60.973	0.2185
60.973	0.2185
60.973	0.2185
60.973	0.21851
60.973	0.21851
60.974	0.21851
60.974	0.21852
60.974	0.21852
60.974	0.21852
60.974	0.21852
60.974	0.21852
60.974	0.21853
60.974	0.21853
60.975	0.21853
60.975	0.21853

60.975	0.21853
60.975	0.21853
60.975	0.21853
60.975	0.21853
60.975	0.21853
60.976	0.21853
60.976	0.2193
60.976	0.22017
60.976	0.22102
60.976	0.22186
60.976	0.22267
60.976	0.22348
60.977	0.22428
60.977	0.22507
60.977	0.22586
60.977	0.22664
60.977	0.22742
60.977	0.2282
60.977	0.22898
60.978	0.22977
60.978	0.2306
60.978	0.23148
60.978	0.23244
60.978	0.23341
60.978	0.23438
60.978	0.23535
60.979	0.23632
60.979	0.23731
60.979	0.2383
60.979	0.23927
60.979	0.24024
60.979	0.24119
60.979	0.24213
60.979	0.24307
60.980	0.24399
60.980	0.24491
60.980	0.24585
60.980	0.2468
60.980	0.24782
60.980	0.24891
60.980	0.25002
60.981	0.25113
60.981	0.25225
60.981	0.25336
60.981	0.25447
60.981	0.25558
60.981	0.25669
60.981	0.25779
60.982	0.25888
60.982	0.25996
60.982	0.26104
60.982	0.26211
60.982	0.26317

60.982	0.26423
60.982	0.26527
60.983	0.2663
60.983	0.26731
60.983	0.26833
60.983	0.26933
60.983	0.27033
60.983	0.27131
60.983	0.27229
60.983	0.27327
60.984	0.27424
60.984	0.27521
60.984	0.27618
60.984	0.27715
60.984	0.27811
60.984	0.27906
60.984	0.28
60.985	0.28093
60.985	0.28186
60.985	0.28277
60.985	0.28368
60.985	0.28497
60.985	0.28635
60.985	0.28773
60.986	0.28909
60.986	0.29044
60.986	0.29178
60.986	0.29311
60.986	0.29444
60.986	0.29575
60.986	0.29711
60.987	0.29847
60.987	0.29983
60.987	0.3012
60.987	0.30256
60.987	0.30392
60.987	0.30528
60.987	0.30672
60.987	0.30875
60.988	0.31075
60.988	0.31272
60.988	0.31467
60.988	0.31659
60.988	0.31847
60.988	0.32031
60.988	0.32212
60.989	0.32389
60.989	0.32562
60.989	0.32731
60.989	0.32896
60.989	0.33057
60.989	0.33213
60.989	0.33365

60.990	0.33513
60.990	0.33657
60.990	0.33797
60.990	0.33934
60.990	0.34066
60.990	0.34195
60.990	0.3432
60.991	0.34442
60.991	0.34561
60.991	0.34676
60.991	0.34789
60.991	0.349
60.991	0.35007
60.991	0.35112
60.991	0.35216
60.992	0.35317
60.992	0.35443
60.992	0.35595
60.992	0.35743
60.992	0.35889
60.992	0.36032
60.992	0.36171
60.993	0.36306
60.993	0.36438
60.993	0.36566
60.993	0.3669
60.993	0.3681
60.993	0.36926
60.993	0.37038
60.994	0.37146
60.994	0.37263
60.994	0.37378
60.994	0.37489
60.994	0.37597
60.994	0.37701
60.994	0.37801
60.995	0.37898
60.995	0.37995
60.995	0.38092
60.995	0.38186
60.995	0.38275
60.995	0.3836
60.995	0.38442
60.995	0.38519
60.996	0.38592
60.996	0.38662
60.996	0.38727
60.996	0.38788
60.996	0.38846
60.996	0.389
60.996	0.3895
60.997	0.38996
60.997	0.39039

60.997	0.39078
60.997	0.39114
60.997	0.39147
60.997	0.39176
60.997	0.39203
60.998	0.39226
60.998	0.39255
60.998	0.39294
60.998	0.3933
60.998	0.39363
60.998	0.39392
60.998	0.39418
60.999	0.39442
60.999	0.39464
60.999	0.39484
60.999	0.395
60.999	0.39513
60.999	0.39522
60.999	0.39528
60.999	0.3953
61.000	0.39529
61.000	0.39523
61.000	0.39514
61.000	0.39501
61.000	0.39484
61.000	0.39462
61.000	0.39437
61.001	0.39408
61.001	0.39375
61.001	0.39389
61.001	0.39417
61.001	0.39444
61.001	0.39476
61.001	0.39507
61.002	0.39539
61.002	0.3957
61.002	0.396
61.002	0.3963
61.002	0.39658
61.002	0.39686
61.002	0.39711
61.003	0.39736
61.003	0.39759
61.003	0.3978
61.003	0.39798
61.003	0.39814
61.003	0.39828
61.003	0.39839
61.003	0.39848
61.004	0.39853
61.004	0.39855
61.004	0.39854
61.004	0.3985

61.004	0.39842
61.004	0.39831
61.004	0.39817
61.005	0.39799
61.005	0.39777
61.005	0.39752
61.005	0.39723
61.005	0.39691
61.005	0.39655
61.005	0.39616
61.006	0.39574
61.006	0.39528
61.006	0.3948
61.006	0.39431
61.006	0.39381
61.006	0.39329
61.006	0.39278
61.007	0.39227
61.007	0.39177
61.007	0.39125
61.007	0.39072
61.007	0.39018
61.007	0.38962
61.007	0.38905
61.008	0.38846
61.008	0.38786
61.008	0.38724
61.008	0.3866
61.008	0.38593
61.008	0.38525
61.008	0.38455
61.008	0.38382
61.009	0.38308
61.009	0.38231
61.009	0.38153
61.009	0.38072
61.009	0.37989
61.009	0.37905
61.009	0.37818
61.010	0.37729
61.010	0.37639
61.010	0.37546
61.010	0.37451
61.010	0.37355
61.010	0.37256
61.010	0.37156
61.011	0.37053
61.011	0.36949
61.011	0.36843
61.011	0.36735
61.011	0.36626
61.011	0.36515
61.011	0.36415

61.012	0.36318
61.012	0.36218
61.012	0.36116
61.012	0.36011
61.012	0.35905
61.012	0.35806
61.012	0.35709
61.013	0.35612
61.013	0.35512
61.013	0.35411
61.013	0.35309
61.013	0.35205
61.013	0.35104
61.013	0.35001
61.013	0.34896
61.014	0.3479
61.014	0.34683
61.014	0.34574
61.014	0.34465
61.014	0.34353
61.014	0.34241
61.014	0.34128
61.015	0.34013
61.015	0.33898
61.015	0.33781
61.015	0.33664
61.015	0.33548
61.015	0.33433
61.015	0.33318
61.016	0.33202
61.016	0.33085
61.016	0.32973
61.016	0.3287
61.016	0.3277
61.016	0.32667
61.016	0.32563
61.017	0.32457
61.017	0.3235
61.017	0.32241
61.017	0.3213
61.017	0.32017
61.017	0.31903
61.017	0.31787
61.018	0.31669
61.018	0.3155
61.018	0.31429
61.018	0.31306
61.018	0.31186
61.018	0.31073
61.018	0.30959
61.019	0.30843
61.019	0.30726
61.019	0.30607

61.019	0.30487
61.019	0.30365
61.019	0.30242
61.019	0.30118
61.020	0.29992
61.020	0.29866
61.020	0.29742
61.020	0.29617
61.020	0.29492
61.020	0.29365
61.020	0.29237
61.021	0.29109
61.021	0.28979
61.021	0.28849
61.021	0.28723
61.021	0.28598
61.021	0.28472
61.021	0.28345
61.022	0.28222
61.022	0.28101
61.022	0.2798
61.022	0.27863
61.022	0.27748
61.022	0.27635
61.023	0.27525
61.023	0.27418
61.023	0.27313
61.023	0.27214
61.023	0.27114
61.023	0.27014
61.023	0.26914
61.024	0.26815
61.024	0.26716
61.024	0.26617
61.024	0.26517
61.024	0.26417
61.024	0.26317
61.024	0.26216
61.025	0.26115
61.025	0.26013
61.025	0.25911
61.025	0.25809
61.025	0.25706
61.025	0.25602
61.025	0.25498
61.026	0.25394
61.026	0.25289
61.026	0.25183
61.026	0.25077
61.026	0.24971
61.026	0.24867
61.027	0.24767
61.027	0.24667

61.027	0.24568
61.027	0.24469
61.027	0.24371
61.027	0.24272
61.027	0.24177
61.028	0.24083
61.028	0.23988
61.028	0.23892
61.028	0.23795
61.028	0.23696
61.028	0.23596
61.028	0.23506
61.029	0.23422
61.029	0.23342
61.029	0.23344
61.029	0.23415
61.029	0.2348
61.029	0.23536
61.029	0.23585
61.030	0.23626
61.030	0.23661
61.030	0.23689
61.030	0.23713
61.030	0.23735
61.030	0.23773
61.031	0.23843
61.031	0.23905
61.031	0.23959
61.031	0.24005
61.031	0.24044
61.031	0.24074
61.031	0.24095
61.032	0.24107
61.032	0.24108
61.032	0.24099
61.032	0.2408
61.032	0.24051
61.032	0.24012
61.033	0.23966
61.033	0.23911
61.033	0.23885
61.033	0.23867
61.033	0.23843
61.033	0.23812
61.033	0.23775
61.034	0.2373
61.034	0.23676
61.034	0.23616
61.034	0.23549
61.034	0.23478
61.034	0.23403
61.034	0.2333
61.035	0.23302

61.035	0.23267
61.035	0.23221
61.035	0.23165
61.035	0.23105
61.035	0.23054
61.036	0.23
61.036	0.22942
61.036	0.22881
61.036	0.22817
61.036	0.22751
61.036	0.22681
61.036	0.22606
61.037	0.22527
61.037	0.22443
61.037	0.22355
61.037	0.22265
61.037	0.22173
61.037	0.22086
61.037	0.22001
61.038	0.21913
61.038	0.21831
61.038	0.21754
61.038	0.21674
61.038	0.21591
61.038	0.21504
61.039	0.21414
61.039	0.21319
61.039	0.21221
61.039	0.2112
61.039	0.21017
61.039	0.20912
61.039	0.20806
61.040	0.207
61.040	0.206
61.040	0.205
61.040	0.20398
61.040	0.20294
61.040	0.20187
61.041	0.20079
61.041	0.1997
61.041	0.1986
61.041	0.1975
61.041	0.19649
61.041	0.19554
61.041	0.19462
61.042	0.19368
61.042	0.19273
61.042	0.19178
61.042	0.19081
61.042	0.19013
61.042	0.19046
61.043	0.19082
61.043	0.19108

61.043	0.19115
61.043	0.19106
61.043	0.1909
61.043	0.1907
61.043	0.19049
61.044	0.1903
61.044	0.19011
61.044	0.18992
61.044	0.18971
61.044	0.18947
61.044	0.18919
61.044	0.18885
61.045	0.18847
61.045	0.18806
61.045	0.18761
61.045	0.18758
61.045	0.18762
61.045	0.18763
61.045	0.18765
61.046	0.18769
61.046	0.18777
61.046	0.18782
61.046	0.18782
61.046	0.18779
61.046	0.18772
61.046	0.18762
61.047	0.18748
61.047	0.18731
61.047	0.1872
61.047	0.18744
61.047	0.18823
61.047	0.18969
61.047	0.19107
61.048	0.19237
61.048	0.19363
61.048	0.19493
61.048	0.19615
61.048	0.19741
61.048	0.19854
61.048	0.1995
61.049	0.20031
61.049	0.20097
61.049	0.20155
61.049	0.20213
61.049	0.20258
61.049	0.2029
61.049	0.20308
61.050	0.20315
61.050	0.2031
61.050	0.20294
61.050	0.2027
61.050	0.20238
61.050	0.20198

61.050	0.20153
61.051	0.20103
61.051	0.20046
61.051	0.20027
61.051	0.19991
61.051	0.19934
61.051	0.19858
61.051	0.1978
61.052	0.19709
61.052	0.19629
61.052	0.19542
61.052	0.19459
61.052	0.19501
61.052	0.19541
61.052	0.19578
61.053	0.19612
61.053	0.19642
61.053	0.19669
61.053	0.19692
61.053	0.19711
61.053	0.19726
61.053	0.19737
61.054	0.19744
61.054	0.19747
61.054	0.19746
61.054	0.19741
61.054	0.19732
61.054	0.1972
61.054	0.19704
61.055	0.19684
61.055	0.1966
61.055	0.19633
61.055	0.19602
61.055	0.19567
61.055	0.19529
61.055	0.19487
61.056	0.19441
61.056	0.19393
61.056	0.19341
61.056	0.19286
61.056	0.19227
61.056	0.19166
61.056	0.19102
61.057	0.19035
61.057	0.18967
61.057	0.18895
61.057	0.18838
61.057	0.18784
61.057	0.18726
61.057	0.18664
61.058	0.18611
61.058	0.18583
61.058	0.18552

61.058	0.18519
61.058	0.18483
61.058	0.18444
61.058	0.18401
61.059	0.18355
61.059	0.18305
61.059	0.1825
61.059	0.18192
61.059	0.18129
61.059	0.18063
61.060	0.17991
61.060	0.17916
61.060	0.17847
61.060	0.17801
61.060	0.17783
61.060	0.1778
61.060	0.17774
61.061	0.17766
61.061	0.17755
61.061	0.17743
61.061	0.17729
61.061	0.17712
61.061	0.17694
61.061	0.17674
61.062	0.17652
61.062	0.17628
61.062	0.17602
61.062	0.17574
61.062	0.1755
61.062	0.17525
61.062	0.17497
61.063	0.17466
61.063	0.17433
61.063	0.17397
61.063	0.17359
61.063	0.17319
61.063	0.17277
61.063	0.17266
61.064	0.17257
61.064	0.17249
61.064	0.17241
61.064	0.17233
61.064	0.17225
61.064	0.17218
61.065	0.17211
61.065	0.17204
61.065	0.17197
61.065	0.17191
61.065	0.17225
61.065	0.1729
61.065	0.17353
61.066	0.17414
61.066	0.17472

61.066	0.17527
61.066	0.17579
61.066	0.17628
61.066	0.17675
61.067	0.17719
61.067	0.17761
61.067	0.17801
61.067	0.17841
61.067	0.17879
61.067	0.17913
61.067	0.17945
61.068	0.17975
61.068	0.18003
61.068	0.18029
61.068	0.18055
61.068	0.18076
61.068	0.18097
61.068	0.18116
61.069	0.18135
61.069	0.18154
61.069	0.18172
61.069	0.18191
61.069	0.18246
61.069	0.18259
61.070	0.18309
61.070	0.1847
61.070	0.18487
61.070	0.18399
61.070	0.18324
61.070	0.18385
61.070	0.18338
61.071	0.1834
61.071	0.18494
61.071	0.1852
61.071	0.18451
61.071	0.18358
61.071	0.18446
61.071	0.18572
61.072	0.18599
61.072	0.18641
61.072	0.18587
61.072	0.1848
61.072	0.185
61.072	0.18516
61.073	0.18537
61.073	0.18571
61.073	0.18542
61.073	0.18521
61.073	0.18514
61.073	0.18497
61.073	0.18463
61.074	0.18413
61.074	0.184

61.074	0.18382
61.074	0.18355
61.074	0.18318
61.074	0.18272
61.074	0.18218
61.075	0.18156
61.075	0.18089
61.075	0.18021
61.075	0.18
61.075	0.17978
61.075	0.17956
61.075	0.1794
61.076	0.1803
61.076	0.18019
61.076	0.17931
61.076	0.17908
61.076	0.17812
61.076	0.17785
61.077	0.1776
61.077	0.17736
61.077	0.17716
61.077	0.17727
61.077	0.17735
61.077	0.1774
61.077	0.17743
61.078	0.17743
61.078	0.1774
61.078	0.17733
61.078	0.17724
61.078	0.17714
61.078	0.17707
61.078	0.17704
61.079	0.17697
61.079	0.17688
61.079	0.17676
61.079	0.17661
61.079	0.17644
61.079	0.17625
61.079	0.17603
61.079	0.1758
61.080	0.17557
61.080	0.17533
61.080	0.17511
61.080	0.17489
61.080	0.17468
61.080	0.17447
61.080	0.17427
61.081	0.17407
61.081	0.17387
61.081	0.17366
61.081	0.17345
61.081	0.17324
61.081	0.17303

61.081	0.17282
61.082	0.17266
61.082	0.17251
61.082	0.17237
61.082	0.17222
61.082	0.17207
61.082	0.17191
61.082	0.17177
61.083	0.17165
61.083	0.17154
61.083	0.17143
61.083	0.17133
61.083	0.17122
61.083	0.17115
61.083	0.17111
61.084	0.17108
61.084	0.17105
61.084	0.17101
61.084	0.17098
61.084	0.17094
61.084	0.1709
61.084	0.17087
61.084	0.17096
61.085	0.17106
61.085	0.17121
61.085	0.1713
61.085	0.17134
61.085	0.17131
61.085	0.17123
61.085	0.17114
61.086	0.17104
61.086	0.17091
61.086	0.17075
61.086	0.17057
61.086	0.17037
61.086	0.17016
61.086	0.16995
61.087	0.16984
61.087	0.16972
61.087	0.16959
61.087	0.16945
61.087	0.16929
61.087	0.16913
61.087	0.16895
61.088	0.16875
61.088	0.16854
61.088	0.16832
61.088	0.16809
61.088	0.16784
61.088	0.16758
61.088	0.16732
61.089	0.16705
61.089	0.16678

61.089	0.1665
61.089	0.16663
61.089	0.16681
61.089	0.16699
61.089	0.16716
61.089	0.16733
61.090	0.16748
61.090	0.16763
61.090	0.16776
61.090	0.16788
61.090	0.16798
61.090	0.16806
61.090	0.16813
61.091	0.16818
61.091	0.16821
61.091	0.16823
61.091	0.16823
61.091	0.16823
61.091	0.16822
61.091	0.16821
61.092	0.16821
61.092	0.16822
61.092	0.16823
61.092	0.16826
61.092	0.16828
61.092	0.16831
61.092	0.16833
61.093	0.16834
61.093	0.16833
61.093	0.1683
61.093	0.16826
61.093	0.16821
61.093	0.16815
61.093	0.16808
61.094	0.168
61.094	0.16792
61.094	0.16784
61.094	0.16776
61.094	0.16768
61.094	0.1676
61.094	0.16751
61.094	0.16743
61.095	0.16734
61.095	0.16727
61.095	0.1672
61.095	0.16713
61.095	0.16707
61.095	0.16699
61.095	0.16691
61.096	0.16682
61.096	0.16671
61.096	0.16659
61.096	0.16648

61.096	0.16637
61.096	0.1664
61.096	0.16673
61.097	0.16704
61.097	0.16734
61.097	0.16763
61.097	0.1679
61.097	0.16815
61.097	0.16838
61.097	0.16859
61.098	0.16879
61.098	0.16897
61.098	0.16913
61.098	0.16927
61.098	0.1694
61.098	0.16951
61.098	0.1696
61.099	0.16968
61.099	0.16975
61.099	0.1698
61.099	0.16997
61.099	0.17023
61.099	0.17047
61.099	0.1707
61.100	0.17093
61.100	0.17114
61.100	0.17133
61.100	0.17152
61.100	0.17169
61.100	0.17183
61.100	0.17197
61.101	0.17208
61.101	0.17217
61.101	0.17225
61.101	0.1723
61.101	0.17233
61.101	0.17234
61.101	0.17234
61.102	0.17231
61.102	0.17226
61.102	0.1722
61.102	0.17212
61.102	0.17204
61.102	0.17195
61.102	0.17186
61.102	0.17175
61.103	0.17163
61.103	0.17149
61.103	0.17133
61.103	0.17115
61.103	0.17097
61.103	0.17082
61.103	0.17067

61.104	0.17051
61.104	0.17034
61.104	0.17023
61.104	0.17012
61.104	0.17
61.104	0.16986
61.104	0.16971
61.105	0.16955
61.105	0.16938
61.105	0.16924
61.105	0.16909
61.105	0.16893
61.105	0.16894
61.105	0.16902
61.106	0.1691
61.106	0.16917
61.106	0.16923
61.106	0.16928
61.106	0.16932
61.106	0.16936
61.106	0.16939
61.107	0.16941
61.107	0.16943
61.107	0.16944
61.107	0.16944
61.107	0.16944
61.107	0.16944
61.107	0.16943
61.108	0.16941
61.108	0.1694
61.108	0.16937
61.108	0.16935
61.108	0.16932
61.108	0.16941
61.108	0.16986
61.109	0.17029
61.109	0.17071
61.109	0.1711
61.109	0.17146
61.109	0.1718
61.109	0.17211
61.109	0.17239
61.110	0.17263
61.110	0.17285
61.110	0.17303
61.110	0.17318
61.110	0.17329
61.110	0.17337
61.110	0.17341
61.111	0.17342
61.111	0.17339
61.111	0.17333
61.111	0.17324

61.111	0.17311
61.111	0.17296
61.111	0.17277
61.112	0.17256
61.112	0.17232
61.112	0.17206
61.112	0.17178
61.112	0.17148
61.112	0.17116
61.112	0.17083
61.113	0.17048
61.113	0.17012
61.113	0.16975
61.113	0.16937
61.113	0.16898
61.113	0.16858
61.113	0.16836
61.114	0.16814
61.114	0.16792
61.114	0.1677
61.114	0.16754
61.114	0.16738
61.114	0.16721
61.114	0.16703
61.115	0.16684
61.115	0.16665
61.115	0.16646
61.115	0.16626
61.115	0.16623
61.115	0.16629
61.115	0.16635
61.116	0.16641
61.116	0.16647
61.116	0.16653
61.116	0.1666
61.116	0.16666
61.116	0.16672
61.116	0.16678
61.117	0.16683
61.117	0.16689
61.117	0.16694
61.117	0.16699
61.117	0.16703
61.117	0.16708
61.117	0.16713
61.118	0.16717
61.118	0.16721
61.118	0.16725
61.118	0.16729
61.118	0.16732
61.118	0.16734
61.118	0.16739
61.119	0.16744

61.119	0.16748
61.119	0.16751
61.119	0.16755
61.119	0.16758
61.119	0.1676
61.119	0.16762
61.119	0.16763
61.120	0.16763
61.120	0.16763
61.120	0.16762
61.120	0.1676
61.120	0.16757
61.120	0.16754
61.120	0.1675
61.121	0.16745
61.121	0.1674
61.121	0.16734
61.121	0.16727
61.121	0.16719
61.121	0.16711
61.121	0.16701
61.122	0.16691
61.122	0.1668
61.122	0.16669
61.122	0.16656
61.122	0.16643
61.122	0.16629
61.122	0.16615
61.123	0.16599
61.123	0.16584
61.123	0.16568
61.123	0.16551
61.123	0.16533
61.123	0.16516
61.123	0.16497
61.124	0.1651
61.124	0.16506
61.124	0.16476
61.124	0.16521
61.124	0.16608
61.124	0.16638
61.124	0.1662
61.125	0.16623
61.125	0.16658
61.125	0.16719
61.125	0.16736
61.125	0.16738
61.125	0.16757
61.126	0.16771
61.126	0.16783
61.126	0.16792
61.126	0.16799
61.126	0.16809

61.126	0.16825
61.126	0.16837
61.127	0.16844
61.127	0.16847
61.127	0.16848
61.127	0.16848
61.127	0.16845
61.127	0.16842
61.127	0.16842
61.128	0.16838
61.128	0.1683
61.128	0.16819
61.128	0.16802
61.128	0.1678
61.128	0.16754
61.128	0.16723
61.129	0.16689
61.129	0.16651
61.129	0.1661
61.129	0.16568
61.129	0.16525
61.129	0.1648
61.129	0.16435
61.130	0.16396
61.130	0.16356
61.130	0.16314
61.130	0.16295
61.130	0.16289
61.130	0.16281
61.130	0.16272
61.131	0.16261
61.131	0.16248
61.131	0.16234
61.131	0.1622
61.131	0.16203
61.131	0.16186
61.131	0.16167
61.132	0.16146
61.132	0.16125
61.132	0.16138
61.132	0.16167
61.132	0.16194
61.132	0.16217
61.133	0.16237
61.133	0.16254
61.133	0.16268
61.133	0.16279
61.133	0.16306
61.133	0.16332
61.133	0.16353
61.134	0.16369
61.134	0.16381
61.134	0.164

61.134	0.16425
61.134	0.16453
61.134	0.16478
61.134	0.16498
61.135	0.16513
61.135	0.16522
61.135	0.16526
61.135	0.16524
61.135	0.16516
61.135	0.16504
61.135	0.16487
61.136	0.16466
61.136	0.16475
61.136	0.16499
61.136	0.16521
61.136	0.1654
61.136	0.16558
61.137	0.16573
61.137	0.16586
61.137	0.16595
61.137	0.16602
61.137	0.16607
61.137	0.16608
61.137	0.16607
61.138	0.16604
61.138	0.16598
61.138	0.16589
61.138	0.16579
61.138	0.16566
61.138	0.16552
61.138	0.16535
61.139	0.16517
61.139	0.16497
61.139	0.16476
61.139	0.16452
61.139	0.16427
61.139	0.16401
61.140	0.16372
61.140	0.16342
61.140	0.1631
61.140	0.16276
61.140	0.1624
61.140	0.16203
61.140	0.16163
61.141	0.16122
61.141	0.1608
61.141	0.16038
61.141	0.15999
61.141	0.15972
61.141	0.15954
61.141	0.15937
61.142	0.15919
61.142	0.159

61.142	0.1588
61.142	0.15893
61.142	0.15944
61.142	0.15994
61.143	0.16044
61.143	0.16094
61.143	0.16144
61.143	0.16194
61.143	0.16244
61.143	0.16297
61.143	0.16349
61.144	0.16399
61.144	0.16445
61.144	0.16489
61.144	0.16528
61.144	0.16565
61.144	0.16597
61.145	0.16626
61.145	0.16652
61.145	0.16674
61.145	0.16693
61.145	0.16709
61.145	0.16722
61.145	0.16732
61.146	0.1674
61.146	0.16745
61.146	0.16748
61.146	0.16749
61.146	0.16747
61.146	0.16744
61.147	0.16738
61.147	0.1673
61.147	0.16721
61.147	0.1671
61.147	0.16697
61.147	0.16683
61.147	0.16668
61.148	0.16651
61.148	0.16634
61.148	0.16616
61.148	0.16616
61.148	0.1662
61.148	0.16622
61.149	0.16624
61.149	0.16624
61.149	0.16624
61.149	0.16621
61.149	0.16618
61.149	0.16613
61.149	0.16606
61.150	0.16598
61.150	0.16588
61.150	0.16575

61.150	0.16561
61.150	0.16545
61.150	0.16526
61.151	0.16506
61.151	0.16483
61.151	0.16458
61.151	0.1643
61.151	0.16401
61.151	0.16369
61.152	0.16335
61.152	0.16299
61.152	0.16321
61.152	0.16371
61.152	0.16423
61.152	0.16471
61.152	0.16514
61.153	0.16552
61.153	0.16585
61.153	0.16614
61.153	0.16638
61.153	0.16658
61.153	0.16674
61.154	0.16685
61.154	0.16693
61.154	0.16697
61.154	0.16697
61.154	0.16693
61.154	0.16685
61.154	0.16675
61.155	0.16661
61.155	0.16645
61.155	0.16626
61.155	0.16627
61.155	0.16651
61.155	0.16674
61.156	0.16695
61.156	0.16715
61.156	0.16734
61.156	0.16752
61.156	0.16768
61.156	0.16782
61.156	0.16795
61.157	0.16805
61.157	0.16814
61.157	0.1682
61.157	0.16825
61.157	0.16828
61.157	0.16828
61.158	0.16826
61.158	0.16823
61.158	0.16817
61.158	0.1681
61.158	0.168

61.158	0.16788
61.158	0.16775
61.159	0.16759
61.159	0.16742
61.159	0.16722
61.159	0.167
61.159	0.16677
61.159	0.16651
61.159	0.16624
61.160	0.16594
61.160	0.16562
61.160	0.16529
61.160	0.16494
61.160	0.16457
61.160	0.16419
61.161	0.16379
61.161	0.16337
61.161	0.16295
61.161	0.16251
61.161	0.16206
61.161	0.1616
61.161	0.16114
61.162	0.16067
61.162	0.16019
61.162	0.15988
61.162	0.15994
61.162	0.16001
61.162	0.16007
61.163	0.16013
61.163	0.16019
61.163	0.1604
61.163	0.16072
61.163	0.16104
61.163	0.16137
61.163	0.16168
61.164	0.16197
61.164	0.16224
61.164	0.16249
61.164	0.16272
61.164	0.16292
61.164	0.1631
61.164	0.16326
61.165	0.16341
61.165	0.16353
61.165	0.16364
61.165	0.16373
61.165	0.1638
61.165	0.16387
61.166	0.16393
61.166	0.164
61.166	0.16406
61.166	0.1641
61.166	0.16413

61.166	0.16414
61.166	0.16413
61.167	0.1641
61.167	0.16406
61.167	0.16401
61.167	0.16394
61.167	0.16394
61.167	0.16408
61.167	0.16422
61.168	0.16435
61.168	0.16447
61.168	0.16458
61.168	0.16468
61.168	0.16478
61.168	0.16486
61.169	0.16493
61.169	0.165
61.169	0.16505
61.169	0.1651
61.169	0.16513
61.169	0.16515
61.169	0.16517
61.170	0.16517
61.170	0.16516
61.170	0.16514
61.170	0.16511
61.170	0.16507
61.170	0.16501
61.171	0.16494
61.171	0.16486
61.171	0.16476
61.171	0.16465
61.171	0.16452
61.171	0.16438
61.171	0.16422
61.172	0.16406
61.172	0.16387
61.172	0.16368
61.172	0.16347
61.172	0.16325
61.172	0.16301
61.172	0.16277
61.173	0.16251
61.173	0.16224
61.173	0.16196
61.173	0.16167
61.173	0.16136
61.173	0.16104
61.174	0.16081
61.174	0.16113
61.174	0.16145
61.174	0.16176
61.174	0.16208

61.174	0.16239
61.174	0.16269
61.175	0.16298
61.175	0.16327
61.175	0.16355
61.175	0.16381
61.175	0.16407
61.175	0.16432
61.175	0.16456
61.176	0.16479
61.176	0.16501
61.176	0.16521
61.176	0.16541
61.176	0.16559
61.176	0.16577
61.177	0.16593
61.177	0.16608
61.177	0.16621
61.177	0.16634
61.177	0.16645
61.177	0.16655
61.177	0.16664
61.178	0.16671
61.178	0.16677
61.178	0.16682
61.178	0.16685
61.178	0.16687
61.178	0.1669
61.178	0.16692
61.179	0.16693
61.179	0.16692
61.179	0.16691
61.179	0.16688
61.179	0.16684
61.179	0.16679
61.180	0.16673
61.180	0.16665
61.180	0.16656
61.180	0.16646
61.180	0.16635
61.180	0.16623
61.180	0.16609
61.181	0.16595
61.181	0.1658
61.181	0.16563
61.181	0.16546
61.181	0.16528
61.181	0.16509
61.182	0.1649
61.182	0.16469
61.182	0.16448
61.182	0.16426
61.182	0.16403

61.182	0.1638
61.182	0.16356
61.183	0.16339
61.183	0.1632
61.183	0.16301
61.183	0.16281
61.183	0.16261
61.183	0.16239
61.184	0.16217
61.184	0.16193
61.184	0.16169
61.184	0.16143
61.184	0.16116
61.184	0.16089
61.184	0.1606
61.185	0.16031
61.185	0.16001
61.185	0.1597
61.185	0.15939
61.185	0.15907
61.185	0.15874
61.185	0.15841
61.186	0.15807
61.186	0.15811
61.186	0.15817
61.186	0.15823
61.186	0.15829
61.186	0.15834
61.186	0.15837
61.187	0.15839
61.187	0.15841
61.187	0.15842
61.187	0.15843
61.187	0.15844
61.187	0.15846
61.187	0.15847
61.188	0.15849
61.188	0.15849
61.188	0.15849
61.188	0.15848
61.188	0.15846
61.188	0.15845
61.188	0.15843
61.189	0.15841
61.189	0.1584
61.189	0.15839
61.189	0.15839
61.189	0.15838
61.189	0.15836
61.189	0.15832
61.189	0.15825
61.190	0.15817
61.190	0.15808

61.190	0.15799
61.190	0.1579
61.190	0.15781
61.190	0.15772
61.190	0.15763
61.191	0.15753
61.191	0.15744
61.191	0.15736
61.191	0.15733
61.191	0.15735
61.191	0.1574
61.191	0.15749
61.192	0.1575
61.192	0.15744
61.192	0.15742
61.192	0.15743
61.192	0.15743
61.192	0.15741
61.192	0.15739
61.193	0.15736
61.193	0.15733
61.193	0.15729
61.193	0.15724
61.193	0.15719
61.193	0.15714
61.193	0.15712
61.194	0.1571
61.194	0.15708
61.194	0.15707
61.194	0.15705
61.194	0.15703
61.194	0.15701
61.194	0.15698
61.195	0.15694
61.195	0.1569
61.195	0.15689
61.195	0.1569
61.195	0.15691
61.195	0.1569
61.195	0.15689
61.195	0.15687
61.196	0.15685
61.196	0.15681
61.196	0.15677
61.196	0.15673
61.196	0.15667
61.196	0.15671
61.196	0.15704
61.197	0.15737
61.197	0.16005
61.197	0.16008
61.197	0.15831
61.197	0.15861

61.197	0.15891
61.197	0.1592
61.198	0.15948
61.198	0.15976
61.198	0.16003
61.198	0.1603
61.198	0.16055
61.198	0.16083
61.198	0.16118
61.199	0.16152
61.199	0.16189
61.199	0.16222
61.199	0.16252
61.199	0.1628
61.199	0.16311
61.199	0.16342
61.200	0.16371
61.200	0.16401
61.200	0.16432
61.200	0.16462
61.200	0.1649
61.200	0.16517
61.200	0.16543
61.200	0.16568
61.201	0.16591
61.201	0.16613
61.201	0.16633
61.201	0.16653
61.201	0.1667
61.201	0.16687
61.201	0.16703
61.202	0.16718
61.202	0.16731
61.202	0.16743
61.202	0.16754
61.202	0.16764
61.202	0.1682
61.202	0.16877
61.203	0.16786
61.203	0.16791
61.203	0.16794
61.203	0.16796
61.203	0.168
61.203	0.16802
61.203	0.16804
61.204	0.16806
61.204	0.16808
61.204	0.16808
61.204	0.16807
61.204	0.16805
61.204	0.16808
61.204	0.1681
61.205	0.16807

61.205	0.16801
61.205	0.1679
61.205	0.16776
61.205	0.1676
61.205	0.16742
61.205	0.16727
61.205	0.16712
61.206	0.16699
61.206	0.16685
61.206	0.1667
61.206	0.16654
61.206	0.16638
61.206	0.1662
61.206	0.16602
61.207	0.16584
61.207	0.16564
61.207	0.16544
61.207	0.16523
61.207	0.16501
61.207	0.16479
61.207	0.16456
61.208	0.16432
61.208	0.16409
61.208	0.16384
61.208	0.1636
61.208	0.16335
61.208	0.16316
61.208	0.16303
61.209	0.16288
61.209	0.16274
61.209	0.16258
61.209	0.16241
61.209	0.16223
61.209	0.16204
61.209	0.16183
61.210	0.16162
61.210	0.16139
61.210	0.16115
61.210	0.1609
61.210	0.16064
61.210	0.16038
61.210	0.16011
61.210	0.15983
61.211	0.15955
61.211	0.15926
61.211	0.15898
61.211	0.15869
61.211	0.15839
61.211	0.15809
61.211	0.15779
61.212	0.15748
61.212	0.15717
61.212	0.15685

61.212	0.15652
61.212	0.15618
61.212	0.15583
61.212	0.15548
61.213	0.15511
61.213	0.15473
61.213	0.15434
61.213	0.15438
61.213	0.15476
61.213	0.15514
61.213	0.1555
61.214	0.15587
61.214	0.15622
61.214	0.15657
61.214	0.15691
61.214	0.15724
61.214	0.15756
61.214	0.15788
61.214	0.15819
61.215	0.15849
61.215	0.15878
61.215	0.15906
61.215	0.15933
61.215	0.1596
61.215	0.15986
61.215	0.16011
61.216	0.16035
61.216	0.16058
61.216	0.1608
61.216	0.16102
61.216	0.16123
61.216	0.16143
61.216	0.16162
61.217	0.1618
61.217	0.16198
61.217	0.16215
61.217	0.16231
61.217	0.16247
61.217	0.16262
61.217	0.16276
61.218	0.16289
61.218	0.16302
61.218	0.16315
61.218	0.16327
61.218	0.16338
61.218	0.16348
61.218	0.16358
61.219	0.16368
61.219	0.16377
61.219	0.16386
61.219	0.16394
61.219	0.16401
61.219	0.16408

61.219	0.16415
61.219	0.16421
61.220	0.16426
61.220	0.16431
61.220	0.16436
61.220	0.1644
61.220	0.16444
61.220	0.16447
61.220	0.1645
61.221	0.16453
61.221	0.16455
61.221	0.16457
61.221	0.16458
61.221	0.16459
61.221	0.1646
61.221	0.1646
61.222	0.1646
61.222	0.16459
61.222	0.16458
61.222	0.16457
61.222	0.16455
61.222	0.16452
61.222	0.16449
61.223	0.16445
61.223	0.16441
61.223	0.16437
61.223	0.16432
61.223	0.16426
61.223	0.1642
61.223	0.16413
61.223	0.16406
61.224	0.16398
61.224	0.1639
61.224	0.16381
61.224	0.16371
61.224	0.16361
61.224	0.1635
61.224	0.16338
61.225	0.16326
61.225	0.16313
61.225	0.16299
61.225	0.16285
61.225	0.1627
61.225	0.16254
61.225	0.16237
61.226	0.1622
61.226	0.16202
61.226	0.16183
61.226	0.16164
61.226	0.16155
61.226	0.16211
61.226	0.16267
61.227	0.16321

61.227	0.16376
61.227	0.1643
61.227	0.16483
61.227	0.16537
61.227	0.16589
61.227	0.16642
61.228	0.16694
61.228	0.16748
61.228	0.16803
61.228	0.16861
61.228	0.16919
61.228	0.16978
61.228	0.17036
61.228	0.17095
61.229	0.17154
61.229	0.17212
61.229	0.1727
61.229	0.17327
61.229	0.17384
61.229	0.17441
61.229	0.17497
61.230	0.17552
61.230	0.17606
61.230	0.1766
61.230	0.17713
61.230	0.17765
61.230	0.17817
61.230	0.17868
61.231	0.17918
61.231	0.17968
61.231	0.18017
61.231	0.18066
61.231	0.18113
61.231	0.18161
61.231	0.18208
61.232	0.18256
61.232	0.18308
61.232	0.1836
61.232	0.18412
61.232	0.18463
61.232	0.18512
61.232	0.18562
61.233	0.1861
61.233	0.18657
61.233	0.18703
61.233	0.18748
61.233	0.18792
61.233	0.18835
61.233	0.18877
61.233	0.18917
61.234	0.18957
61.234	0.18995
61.234	0.19032

61.234	0.19068
61.234	0.19109
61.234	0.19153
61.234	0.19197
61.235	0.1924
61.235	0.19282
61.235	0.19324
61.235	0.19365
61.235	0.19405
61.235	0.19444
61.235	0.19482
61.236	0.1952
61.236	0.19557
61.236	0.19593
61.236	0.19628
61.236	0.19662
61.236	0.19696
61.236	0.19728
61.237	0.1976
61.237	0.19791
61.237	0.19821
61.237	0.19851
61.237	0.1988
61.237	0.19908
61.237	0.19935
61.237	0.19962
61.238	0.19987
61.238	0.20012
61.238	0.20037
61.238	0.2006
61.238	0.20083
61.238	0.20106
61.238	0.20127
61.239	0.20148
61.239	0.20169
61.239	0.20188
61.239	0.20207
61.239	0.20226
61.239	0.20244
61.239	0.20261
61.240	0.20277
61.240	0.20293
61.240	0.20308
61.240	0.20322
61.240	0.20336
61.240	0.20348
61.240	0.20361
61.241	0.20372
61.241	0.20382
61.241	0.20392
61.241	0.20401
61.241	0.20409
61.241	0.20417

61.241	0.20423
61.242	0.20428
61.242	0.20433
61.242	0.20437
61.242	0.20439
61.242	0.20441
61.242	0.20442
61.242	0.20441
61.242	0.2044
61.243	0.20438
61.243	0.20435
61.243	0.2043
61.243	0.20425
61.243	0.20419
61.243	0.20412
61.243	0.20403
61.244	0.20394
61.244	0.20384
61.244	0.20372
61.244	0.2036
61.244	0.20347
61.244	0.20333
61.244	0.20318
61.245	0.20302
61.245	0.20285
61.245	0.20268
61.245	0.2025
61.245	0.2023
61.245	0.20211
61.245	0.20192
61.246	0.20171
61.246	0.20151
61.246	0.20129
61.246	0.20113
61.246	0.20109
61.246	0.20104
61.246	0.20098
61.247	0.20092
61.247	0.20085
61.247	0.20077
61.247	0.20069
61.247	0.2006
61.247	0.2005
61.247	0.20039
61.248	0.20028
61.248	0.20017
61.248	0.20004
61.248	0.19992
61.248	0.19978
61.248	0.19964
61.248	0.1995
61.248	0.19935
61.249	0.1992

61.249	0.19904
61.249	0.19888
61.249	0.19872
61.249	0.19855
61.249	0.19839
61.249	0.19822
61.250	0.19804
61.250	0.19787
61.250	0.19768
61.250	0.19749
61.250	0.1973
61.250	0.1971
61.250	0.1969
61.251	0.19669
61.251	0.19648
61.251	0.19632
61.251	0.1962
61.251	0.19607
61.251	0.19594
61.251	0.1958
61.252	0.19565
61.252	0.1955
61.252	0.19534
61.252	0.19518
61.252	0.19501
61.252	0.19483
61.252	0.19465
61.253	0.19448
61.253	0.19431
61.253	0.19412
61.253	0.19394
61.253	0.19374
61.253	0.19355
61.253	0.19334
61.254	0.19313
61.254	0.19291
61.254	0.19269
61.254	0.19246
61.254	0.19223
61.254	0.19199
61.254	0.19175
61.255	0.1915
61.255	0.19124
61.255	0.19098
61.255	0.19071
61.255	0.19044
61.255	0.19016
61.255	0.18988
61.256	0.1896
61.256	0.1893
61.256	0.18901
61.256	0.18871
61.256	0.18841

61.256	0.1881
61.256	0.18779
61.256	0.18747
61.257	0.18715
61.257	0.18683
61.257	0.1865
61.257	0.18617
61.257	0.18584
61.257	0.18551
61.257	0.18517
61.258	0.18483
61.258	0.18448
61.258	0.18414
61.258	0.18379
61.258	0.18343
61.258	0.18308
61.258	0.18272
61.259	0.18237
61.259	0.18201
61.259	0.18164
61.259	0.18128
61.259	0.18092
61.259	0.18056
61.259	0.18023
61.260	0.17991
61.260	0.17961
61.260	0.17931
61.260	0.17901
61.260	0.17871
61.260	0.17841
61.260	0.1781
61.261	0.17779
61.261	0.17748
61.261	0.17717
61.261	0.17685
61.261	0.17653
61.261	0.17621
61.261	0.17589
61.262	0.17556
61.262	0.17524
61.262	0.17491
61.262	0.17457
61.262	0.17424
61.262	0.1739
61.262	0.17356
61.263	0.17321
61.263	0.17287
61.263	0.17252
61.263	0.17217
61.263	0.17182
61.263	0.17147
61.263	0.17111
61.264	0.17075

61.264	0.17039
61.264	0.17003
61.264	0.16966
61.264	0.1693
61.264	0.16893
61.264	0.16856
61.265	0.16819
61.265	0.16783
61.265	0.16746
61.265	0.16709
61.265	0.16672
61.265	0.16635
61.265	0.16598
61.266	0.16561
61.266	0.16523
61.266	0.16492
61.266	0.16522
61.266	0.16552
61.266	0.16581
61.266	0.1661
61.266	0.16638
61.267	0.16665
61.267	0.16692
61.267	0.16718
61.267	0.16744
61.267	0.16769
61.267	0.16794
61.267	0.16818
61.268	0.16841
61.268	0.16864
61.268	0.16886
61.268	0.16908
61.268	0.16929
61.268	0.16949
61.268	0.16969
61.269	0.16988
61.269	0.17006
61.269	0.17024
61.269	0.17042
61.269	0.17058
61.269	0.17074
61.269	0.1709
61.270	0.17105
61.270	0.17119
61.270	0.17133
61.270	0.17146
61.270	0.1716
61.270	0.17174
61.270	0.17187
61.271	0.172
61.271	0.17212
61.271	0.17223
61.271	0.17233

61.271	0.17243
61.271	0.17252
61.271	0.1726
61.272	0.17268
61.272	0.17275
61.272	0.17281
61.272	0.17287
61.272	0.17292
61.272	0.17297
61.272	0.17301
61.273	0.17304
61.273	0.17307
61.273	0.17309
61.273	0.17311
61.273	0.17312
61.273	0.17313
61.273	0.17313
61.273	0.17313
61.274	0.17312
61.274	0.17311
61.274	0.17309
61.274	0.17307
61.274	0.17304
61.274	0.173
61.274	0.17297
61.275	0.17292
61.275	0.17287
61.275	0.17282
61.275	0.17276
61.275	0.1727
61.275	0.17263
61.275	0.17255
61.276	0.17247
61.276	0.17239
61.276	0.1723
61.276	0.1722
61.276	0.1721
61.276	0.17199
61.276	0.17188
61.277	0.17176
61.277	0.17164
61.277	0.17151
61.277	0.17138
61.277	0.17124
61.277	0.1711
61.277	0.17095
61.278	0.1708
61.278	0.17064
61.278	0.17047
61.278	0.1703
61.278	0.17012
61.278	0.16994
61.278	0.16975

61.279	0.16955
61.279	0.16935
61.279	0.16914
61.279	0.16892
61.279	0.1687
61.279	0.16847
61.279	0.16824
61.279	0.168
61.280	0.16776
61.280	0.16751
61.280	0.16725
61.280	0.16699
61.280	0.16672
61.280	0.16644
61.280	0.16616
61.281	0.16587
61.281	0.16558
61.281	0.16528
61.281	0.16497
61.281	0.16466
61.281	0.16434
61.281	0.16402
61.282	0.16369
61.282	0.16335
61.282	0.16301
61.282	0.16266
61.282	0.16231
61.282	0.16195
61.282	0.16159
61.283	0.16122
61.283	0.16084
61.283	0.16046
61.283	0.16008
61.283	0.15969
61.283	0.1593
61.283	0.1589
61.284	0.1585
61.284	0.15809
61.284	0.15768
61.284	0.15727
61.284	0.15685
61.284	0.15643
61.284	0.15601
61.284	0.15558
61.285	0.15514
61.285	0.15471
61.285	0.15427
61.285	0.15383
61.285	0.15339
61.285	0.15296
61.285	0.15252
61.286	0.15208
61.286	0.15163

61.286	0.15118
61.286	0.15074
61.286	0.15028
61.286	0.14983
61.286	0.14938
61.287	0.14892
61.287	0.14846
61.287	0.148
61.287	0.14754
61.287	0.14708
61.287	0.14661
61.287	0.14615
61.288	0.14569
61.288	0.14525
61.288	0.14481
61.288	0.14437
61.288	0.14393
61.288	0.14349
61.288	0.14304
61.289	0.14259
61.289	0.14214
61.289	0.14169
61.289	0.14166
61.289	0.14078
61.289	0.14032
61.289	0.13986
61.290	0.1394
61.290	0.13894
61.290	0.13847
61.290	0.13801
61.290	0.13888
61.290	0.13908
61.290	0.13921
61.290	0.14019
61.291	0.13873
61.291	0.13628
61.291	0.13722
61.291	0.13765
61.291	0.13708
61.291	0.13565
61.291	0.13733
61.292	0.1378
61.292	0.13629
61.292	0.13614
61.292	0.13508
61.292	0.1348
61.292	0.135
61.292	0.13407
61.293	0.13601
61.293	0.13562
61.293	0.1352
61.293	0.13579
61.293	0.13423

61.293	0.13361
61.293	0.13353
61.294	0.13344
61.294	0.13462
61.294	0.13384
61.294	0.13433
61.294	0.13477
61.294	0.13306
61.294	0.13291
61.295	0.13287
61.295	0.13282
61.295	0.1333
61.295	0.1339
61.295	0.13268
61.295	0.13262
61.295	0.13257
61.295	0.13286
61.296	0.1326
61.296	0.13293
61.296	0.13233
61.296	0.13226
61.296	0.13219
61.296	0.13211
61.296	0.13203
61.297	0.13195
61.297	0.13185
61.297	0.13176
61.297	0.13166
61.297	0.13156
61.297	0.13146
61.297	0.13138
61.298	0.13129
61.298	0.13119
61.298	0.1311
61.298	0.13101
61.298	0.13091
61.298	0.13083
61.298	0.13074
61.299	0.13065
61.299	0.13054
61.299	0.13045
61.299	0.13036
61.299	0.13025
61.299	0.13013
61.299	0.13001
61.300	0.1299
61.300	0.12978
61.300	0.12966
61.300	0.12954
61.300	0.1294
61.300	0.12927
61.300	0.12914
61.301	0.12901

61.301	0.12887
61.301	0.12874
61.301	0.1286
61.301	0.12846
61.301	0.12832
61.301	0.12817
61.302	0.12803
61.302	0.12789
61.302	0.12774
61.302	0.1276
61.302	0.12745
61.302	0.12732
61.302	0.12718
61.303	0.12702
61.303	0.12686
61.303	0.12671
61.303	0.12657
61.303	0.12643
61.303	0.12629
61.303	0.12614
61.304	0.126
61.304	0.12585
61.304	0.1257
61.304	0.12555
61.304	0.1254
61.304	0.12524
61.304	0.12508
61.304	0.12492
61.305	0.12475
61.305	0.12459
61.305	0.12442
61.305	0.12425
61.305	0.12408
61.305	0.1239
61.305	0.12372
61.306	0.12354
61.306	0.12341
61.306	0.12362
61.306	0.12383
61.306	0.12405
61.306	0.12426
61.306	0.12446
61.307	0.12467
61.307	0.12486
61.307	0.12506
61.307	0.12525
61.307	0.12544
61.307	0.12562
61.307	0.1258
61.308	0.12598
61.308	0.12614
61.308	0.12631
61.308	0.12647

61.308	0.12662
61.308	0.12677
61.308	0.12692
61.309	0.12706
61.309	0.12719
61.309	0.12732
61.309	0.12744
61.309	0.12763
61.309	0.12791
61.309	0.12818
61.310	0.12848
61.310	0.1288
61.310	0.12911
61.310	0.12941
61.310	0.1297
61.310	0.12998
61.310	0.13026
61.311	0.13052
61.311	0.13078
61.311	0.13102
61.311	0.13126
61.311	0.1315
61.311	0.13174
61.311	0.13196
61.312	0.13217
61.312	0.13237
61.312	0.13256
61.312	0.13273
61.312	0.13288
61.312	0.13303
61.312	0.13316
61.313	0.13328
61.313	0.13338
61.313	0.13348
61.313	0.13356
61.313	0.13362
61.313	0.13368
61.313	0.13372
61.314	0.13375
61.314	0.13377
61.314	0.13378
61.314	0.13378
61.314	0.13377
61.314	0.13375
61.314	0.13372
61.315	0.13368
61.315	0.13364
61.315	0.13358
61.315	0.13352
61.315	0.13345
61.315	0.13337
61.315	0.13329
61.316	0.1332

61.316	0.13311
61.316	0.13301
61.316	0.13291
61.316	0.1328
61.316	0.13268
61.316	0.13257
61.316	0.13244
61.317	0.13232
61.317	0.13219
61.317	0.13206
61.317	0.13192
61.317	0.13178
61.317	0.13164
61.317	0.13149
61.318	0.13135
61.318	0.1312
61.318	0.13104
61.318	0.13089
61.318	0.13073
61.318	0.13057
61.318	0.13041
61.319	0.13025
61.319	0.13008
61.319	0.12991
61.319	0.12974
61.319	0.12957
61.319	0.1294
61.319	0.12922
61.320	0.12904
61.320	0.12886
61.320	0.12868
61.320	0.12849
61.320	0.1283
61.320	0.12811
61.320	0.12792
61.321	0.12773
61.321	0.12753
61.321	0.12734
61.321	0.12714
61.321	0.12694
61.321	0.12673
61.321	0.12653
61.322	0.12632
61.322	0.12611
61.322	0.1259
61.322	0.12569
61.322	0.12547
61.322	0.12526
61.322	0.12504
61.323	0.12482
61.323	0.1246
61.323	0.12438
61.323	0.12427

61.323	0.12416
61.323	0.12405
61.323	0.12393
61.324	0.12381
61.324	0.12369
61.324	0.12357
61.324	0.12344
61.324	0.12331
61.324	0.12318
61.324	0.12305
61.325	0.12291
61.325	0.12277
61.325	0.12263
61.325	0.12249
61.325	0.12234
61.325	0.1222
61.325	0.12205
61.326	0.1219
61.326	0.12174
61.326	0.12159
61.326	0.12145
61.326	0.12132
61.326	0.12118
61.326	0.12104
61.327	0.12089
61.327	0.12074
61.327	0.12059
61.327	0.12044
61.327	0.12029
61.327	0.12013
61.327	0.11997
61.328	0.11981
61.328	0.11964
61.328	0.11948
61.328	0.11931
61.328	0.11914
61.328	0.11896
61.328	0.11879
61.329	0.11861
61.329	0.11843
61.329	0.11824
61.329	0.11806
61.329	0.11787
61.329	0.11769
61.329	0.1175
61.330	0.11733
61.330	0.11733
61.330	0.11734
61.330	0.11734
61.330	0.11733
61.330	0.11731
61.330	0.11728
61.331	0.11736

61.331	0.11752
61.331	0.11768
61.331	0.11783
61.331	0.11798
61.331	0.11812
61.331	0.11826
61.332	0.1184
61.332	0.11854
61.332	0.11867
61.332	0.11881
61.332	0.11894
61.332	0.11907
61.332	0.1192
61.333	0.11932
61.333	0.11943
61.333	0.11953
61.333	0.11964
61.333	0.11975
61.333	0.11985
61.333	0.11994
61.334	0.12003
61.334	0.12012
61.334	0.1202
61.334	0.12029
61.334	0.12037
61.334	0.12046
61.334	0.12054
61.335	0.12063
61.335	0.1207
61.335	0.12077
61.335	0.12084
61.335	0.12091
61.335	0.12097
61.335	0.12102
61.336	0.12107
61.336	0.1211
61.336	0.12115
61.336	0.12118
61.336	0.12121
61.336	0.12124
61.336	0.12127
61.337	0.12129
61.337	0.12132
61.337	0.12135
61.337	0.12138
61.337	0.12141
61.337	0.12144
61.337	0.12145
61.338	0.12147
61.338	0.12148
61.338	0.12148
61.338	0.12147
61.338	0.12145

61.338	0.12143
61.338	0.12141
61.339	0.12137
61.339	0.12145
61.339	0.12152
61.339	0.12159
61.339	0.12164
61.339	0.12169
61.339	0.12173
61.340	0.12176
61.340	0.12179
61.340	0.1218
61.340	0.12181
61.340	0.12181
61.340	0.12181
61.340	0.12181
61.341	0.1218
61.341	0.12178
61.341	0.12176
61.341	0.12173
61.341	0.1217
61.341	0.12166
61.341	0.12161
61.342	0.12156
61.342	0.12151
61.342	0.12144
61.342	0.12137
61.342	0.1213
61.342	0.12122
61.342	0.12114
61.343	0.12105
61.343	0.12096
61.343	0.12086
61.343	0.12075
61.343	0.12065
61.343	0.12053
61.343	0.12042
61.344	0.1203
61.344	0.12017
61.344	0.12005
61.344	0.11992
61.344	0.11979
61.344	0.11966
61.344	0.11952
61.345	0.1204
61.345	0.12014
61.345	0.11908
61.345	0.11893
61.345	0.11878
61.345	0.11998
61.345	0.11952
61.346	0.1183
61.346	0.11813

61.346	0.11797
61.346	0.1178
61.346	0.11763
61.346	0.11745
61.347	0.11907
61.347	0.11907
61.347	0.11819
61.347	0.11699
61.347	0.11659
61.347	0.11642
61.347	0.11673
61.348	0.11897
61.348	0.11876
61.348	0.11759
61.348	0.11614
61.348	0.11582
61.348	0.11574
61.348	0.11565
61.349	0.11557
61.349	0.11549
61.349	0.1154
61.349	0.11532
61.349	0.11523
61.349	0.11514
61.349	0.11506
61.350	0.11497
61.350	0.11487
61.350	0.11484
61.350	0.11499
61.350	0.11514
61.350	0.11529
61.350	0.11544
61.351	0.11559
61.351	0.11575
61.351	0.1159
61.351	0.11608
61.351	0.11629
61.351	0.11649
61.351	0.1167
61.352	0.1169
61.352	0.11711
61.352	0.11732
61.352	0.11752
61.352	0.11773
61.352	0.11793
61.352	0.11814
61.353	0.11834
61.353	0.11854
61.353	0.11874
61.353	0.11894
61.353	0.11913
61.353	0.11932
61.353	0.11951

61.354	0.1197
61.354	0.11988
61.354	0.12006
61.354	0.12023
61.354	0.1204
61.354	0.12057
61.354	0.12073
61.355	0.12089
61.355	0.12104
61.355	0.12118
61.355	0.12132
61.355	0.12145
61.355	0.12158
61.355	0.1217
61.356	0.12181
61.356	0.12191
61.356	0.12201
61.356	0.1221
61.356	0.12218
61.356	0.12225
61.356	0.12231
61.356	0.12237
61.357	0.12241
61.357	0.12245
61.357	0.12248
61.357	0.1225
61.357	0.12251
61.357	0.12251
61.357	0.1225
61.358	0.12249
61.358	0.12246
61.358	0.12243
61.358	0.12238
61.358	0.12233
61.358	0.12227
61.358	0.1222
61.359	0.12218
61.359	0.12219
61.359	0.1222
61.359	0.12219
61.359	0.12218
61.359	0.12217
61.359	0.12215
61.360	0.12212
61.360	0.12208
61.360	0.12204
61.360	0.12199
61.360	0.12193
61.360	0.12187
61.360	0.1218
61.361	0.12172
61.361	0.12164
61.361	0.12155

61.361	0.12145
61.361	0.12135
61.361	0.12124
61.361	0.12112
61.362	0.12099
61.362	0.12086
61.362	0.12072
61.362	0.12058
61.362	0.12043
61.362	0.12027
61.362	0.12011
61.363	0.11994
61.363	0.11976
61.363	0.11958
61.363	0.11939
61.363	0.1192
61.363	0.119
61.363	0.11879
61.364	0.11858
61.364	0.11836
61.364	0.11814
61.364	0.11792
61.364	0.1177
61.364	0.11775
61.364	0.11779
61.365	0.11782
61.365	0.11784
61.365	0.11785
61.365	0.11786
61.365	0.11786
61.365	0.11789
61.365	0.11796
61.365	0.11802
61.366	0.11807
61.366	0.11811
61.366	0.11814
61.366	0.11816
61.366	0.11818
61.366	0.11818
61.366	0.11818
61.367	0.11817
61.367	0.11815
61.367	0.11812
61.367	0.11808
61.367	0.11803
61.367	0.11798
61.367	0.11792
61.368	0.11785
61.368	0.11777
61.368	0.11769
61.368	0.1176
61.368	0.11751
61.368	0.1174

61.368	0.11729
61.369	0.11718
61.369	0.11707
61.369	0.11695
61.369	0.11683
61.369	0.11671
61.369	0.11658
61.369	0.11644
61.370	0.1163
61.370	0.11616
61.370	0.116
61.370	0.11585
61.370	0.11569
61.370	0.11552
61.370	0.11535
61.371	0.11518
61.371	0.115
61.371	0.11481
61.371	0.11463
61.371	0.11444
61.371	0.11424
61.371	0.11404
61.371	0.11384
61.372	0.11364
61.372	0.11343
61.372	0.11322
61.372	0.113
61.372	0.11279
61.372	0.11257
61.372	0.11235
61.373	0.11235
61.373	0.11238
61.373	0.11241
61.373	0.11244
61.373	0.11251
61.373	0.11259
61.373	0.11267
61.374	0.11276
61.374	0.11284
61.374	0.11291
61.374	0.11299
61.374	0.11306
61.374	0.11314
61.374	0.11321
61.375	0.11327
61.375	0.11334
61.375	0.1134
61.375	0.11347
61.375	0.11353
61.375	0.11359
61.375	0.11365
61.376	0.1137
61.376	0.11376

61.376	0.11381
61.376	0.11386
61.376	0.11391
61.376	0.11396
61.376	0.11401
61.377	0.11406
61.377	0.1141
61.377	0.11415
61.377	0.11419
61.377	0.11423
61.377	0.11427
61.377	0.11431
61.377	0.11435
61.378	0.11438
61.378	0.11442
61.378	0.11445
61.378	0.11448
61.378	0.11451
61.378	0.11454
61.378	0.11456
61.379	0.11458
61.379	0.11461
61.379	0.11463
61.379	0.11465
61.379	0.11466
61.379	0.11468
61.379	0.11469
61.380	0.1147
61.380	0.11471
61.380	0.11472
61.380	0.11473
61.380	0.11473
61.380	0.11474
61.380	0.11474
61.381	0.11474
61.381	0.11473
61.381	0.11473
61.381	0.11473
61.381	0.11472
61.381	0.11471
61.381	0.1147
61.382	0.11469
61.382	0.11468
61.382	0.11466
61.382	0.11465
61.382	0.11463
61.382	0.11467
61.382	0.1147
61.383	0.11473
61.383	0.11476
61.383	0.11479
61.383	0.11482
61.383	0.11484

61.383	0.11487
61.383	0.11489
61.384	0.11491
61.384	0.11493
61.384	0.11494
61.384	0.11495
61.384	0.11496
61.384	0.11497
61.384	0.11498
61.385	0.11499
61.385	0.11499
61.385	0.11499
61.385	0.11499
61.385	0.11498
61.385	0.11498
61.385	0.11497
61.386	0.11496
61.386	0.11495
61.386	0.11494
61.386	0.11492
61.386	0.11491
61.386	0.11489
61.386	0.11487
61.387	0.11485
61.387	0.11483
61.387	0.11481
61.387	0.11478
61.387	0.11476
61.387	0.11473
61.387	0.1147
61.388	0.11467
61.388	0.11464
61.388	0.1146
61.388	0.11457
61.388	0.11453
61.388	0.11449
61.388	0.11445
61.389	0.1144
61.389	0.11436
61.389	0.11431
61.389	0.11426
61.389	0.11421
61.389	0.11416
61.389	0.1141
61.390	0.11404
61.390	0.11398
61.390	0.11392
61.390	0.11386
61.390	0.11379
61.390	0.11372
61.390	0.11365
61.391	0.11358
61.391	0.11351

61.391	0.11343
61.391	0.11336
61.391	0.11328
61.391	0.1132
61.391	0.11311
61.392	0.11303
61.392	0.11295
61.392	0.11286
61.392	0.11277
61.392	0.11269
61.392	0.1126
61.392	0.11255
61.393	0.11252
61.393	0.1125
61.393	0.11248
61.393	0.11246
61.393	0.11244
61.393	0.11241
61.393	0.11241
61.394	0.11249
61.394	0.11257
61.394	0.11265
61.394	0.11272
61.394	0.1128
61.394	0.11288
61.394	0.11296
61.395	0.11304
61.395	0.11312
61.395	0.11319
61.395	0.11327
61.395	0.11334
61.395	0.11342
61.395	0.11349
61.396	0.11356
61.396	0.11363
61.396	0.1137
61.396	0.11377
61.396	0.11383
61.396	0.1139
61.396	0.11396
61.397	0.11403
61.397	0.11408
61.397	0.11414
61.397	0.1142
61.397	0.11425
61.397	0.1143
61.398	0.11434
61.398	0.11439
61.398	0.11443
61.398	0.11447
61.398	0.1145
61.398	0.11454
61.398	0.11457

61.399	0.1146
61.399	0.11463
61.399	0.11466
61.399	0.11468
61.399	0.11471
61.399	0.11473
61.399	0.11475
61.400	0.11477
61.400	0.11478
61.400	0.1148
61.400	0.11481
61.400	0.11481
61.400	0.11482
61.400	0.11482
61.401	0.11482
61.401	0.11481
61.401	0.1148
61.401	0.11479
61.401	0.11478
61.401	0.11476
61.401	0.11474
61.402	0.11471
61.402	0.11468
61.402	0.11465
61.402	0.11462
61.402	0.11459
61.402	0.11455
61.402	0.11451
61.403	0.11447
61.403	0.11442
61.403	0.11437
61.403	0.11432
61.403	0.11427
61.403	0.11422
61.404	0.11416
61.404	0.1141
61.404	0.11411
61.404	0.11418
61.404	0.11425
61.404	0.11432
61.404	0.11438
61.405	0.11445
61.405	0.1145
61.405	0.11456
61.405	0.11461
61.405	0.11465
61.405	0.11469
61.405	0.11473
61.406	0.11477
61.406	0.1148
61.406	0.11483
61.406	0.11485
61.406	0.11488

61.406	0.1149
61.406	0.11492
61.407	0.11494
61.407	0.11495
61.407	0.11496
61.407	0.11497
61.407	0.11498
61.407	0.11498
61.407	0.11498
61.408	0.11497
61.408	0.11496
61.408	0.11495
61.408	0.11494
61.408	0.11491
61.408	0.11489
61.408	0.11486
61.409	0.11483
61.409	0.1148
61.409	0.11476
61.409	0.11472
61.409	0.11468
61.409	0.11463
61.409	0.11458
61.410	0.11453
61.410	0.11447
61.410	0.11441
61.410	0.11435
61.410	0.11429
61.410	0.11422
61.411	0.11415
61.411	0.11407
61.411	0.114
61.411	0.11392
61.411	0.11384
61.411	0.11376
61.411	0.11367
61.412	0.11359
61.412	0.1135
61.412	0.11341
61.412	0.11331
61.412	0.11322
61.412	0.11312
61.412	0.11302
61.413	0.11292
61.413	0.11282
61.413	0.11271
61.413	0.1126
61.413	0.11249
61.413	0.11238
61.413	0.11227
61.414	0.11216
61.414	0.11204
61.414	0.11192

61.414	0.11181
61.414	0.11169
61.414	0.11157
61.414	0.11144
61.415	0.11132
61.415	0.11122
61.415	0.11112
61.415	0.11102
61.415	0.11091
61.415	0.11081
61.415	0.1107
61.416	0.11066
61.416	0.11075
61.416	0.11084
61.416	0.11092
61.416	0.111
61.416	0.11108
61.417	0.11116
61.417	0.11123
61.417	0.11131
61.417	0.11137
61.417	0.11144
61.417	0.11151
61.417	0.11157
61.418	0.11163
61.418	0.11168
61.418	0.11174
61.418	0.11179
61.418	0.11184
61.418	0.11188
61.418	0.11193
61.419	0.11198
61.419	0.11203
61.419	0.11207
61.419	0.11212
61.419	0.11216
61.419	0.11219
61.419	0.11223
61.420	0.11226
61.420	0.11229
61.420	0.11232
61.420	0.11234
61.420	0.11236
61.420	0.11238
61.420	0.11239
61.421	0.1124
61.421	0.11241
61.421	0.11242
61.421	0.11242
61.421	0.11242
61.421	0.11242
61.422	0.11242
61.422	0.11241

61.422	0.1124
61.422	0.11239
61.422	0.11237
61.422	0.11235
61.422	0.11233
61.423	0.11231
61.423	0.11228
61.423	0.11225
61.423	0.11222
61.423	0.11219
61.423	0.11215
61.423	0.11211
61.424	0.11207
61.424	0.11203
61.424	0.11198
61.424	0.11193
61.424	0.11188
61.424	0.11183
61.424	0.11178
61.425	0.11172
61.425	0.11166
61.425	0.1116
61.425	0.11153
61.425	0.11147
61.425	0.1114
61.425	0.11133
61.426	0.11126
61.426	0.11119
61.426	0.11112
61.426	0.11104
61.426	0.11096
61.426	0.11088
61.427	0.1108
61.427	0.11072
61.427	0.11063
61.427	0.11055
61.427	0.11046
61.427	0.11037
61.427	0.11029
61.428	0.1102
61.428	0.11011
61.428	0.11001
61.428	0.10992
61.428	0.10983
61.428	0.10973
61.428	0.10963
61.429	0.10953
61.429	0.10943
61.429	0.10933
61.429	0.10923
61.429	0.10913
61.429	0.10902
61.429	0.10892

61.430	0.10881
61.430	0.1087
61.430	0.10859
61.430	0.1085
61.430	0.10841
61.430	0.10831
61.430	0.10822
61.431	0.10812
61.431	0.10803
61.431	0.10793
61.431	0.10783
61.431	0.10787
61.431	0.10794
61.432	0.10801
61.432	0.10808
61.432	0.10815
61.432	0.10822
61.432	0.10828
61.432	0.10835
61.432	0.10841
61.433	0.10847
61.433	0.10853
61.433	0.10859
61.433	0.10865
61.433	0.1087
61.433	0.10876
61.433	0.10881
61.434	0.10886
61.434	0.10891
61.434	0.10896
61.434	0.109
61.434	0.10905
61.434	0.10909
61.434	0.10913
61.435	0.10917
61.435	0.10921
61.435	0.10925
61.435	0.10929
61.435	0.10932
61.435	0.10936
61.436	0.10939
61.436	0.10942
61.436	0.10945
61.436	0.10949
61.436	0.10953
61.436	0.10956
61.436	0.10959
61.437	0.10963
61.437	0.10966
61.437	0.10969
61.437	0.10971
61.437	0.10974
61.437	0.10976

61.437	0.10978
61.438	0.1098
61.438	0.10981
61.438	0.10983
61.438	0.10984
61.438	0.10985
61.438	0.10985
61.438	0.10986
61.439	0.10986
61.439	0.10986
61.439	0.10985
61.439	0.10985
61.439	0.10984
61.439	0.10983
61.440	0.10982
61.440	0.1098
61.440	0.10978
61.440	0.10976
61.440	0.10974
61.440	0.10972
61.440	0.10969
61.441	0.10966
61.441	0.10963
61.441	0.10959
61.441	0.10956
61.441	0.1096
61.441	0.10974
61.441	0.10987
61.442	0.11
61.442	0.11013
61.442	0.11025
61.442	0.11037
61.442	0.11049
61.442	0.1106
61.442	0.11071
61.443	0.11081
61.443	0.11091
61.443	0.11101
61.443	0.1111
61.443	0.11119
61.443	0.11128
61.444	0.11136
61.444	0.11144
61.444	0.11151
61.444	0.11158
61.444	0.11165
61.444	0.11171
61.444	0.11177
61.445	0.11182
61.445	0.11188
61.445	0.11192
61.445	0.11197
61.445	0.11201

61.445	0.11204
61.445	0.11207
61.446	0.1121
61.446	0.11213
61.446	0.11215
61.446	0.11217
61.446	0.11218
61.446	0.11219
61.447	0.1122
61.447	0.1122
61.447	0.1122
61.447	0.1122
61.447	0.1122
61.447	0.11219
61.447	0.11217
61.448	0.11217
61.448	0.11216
61.448	0.11216
61.448	0.11215
61.448	0.11214
61.448	0.11212
61.448	0.1121
61.449	0.11208
61.449	0.11205
61.449	0.11202
61.449	0.11198
61.449	0.11194
61.449	0.1119
61.449	0.11185
61.450	0.1118
61.450	0.11175
61.450	0.11169
61.450	0.11163
61.450	0.11156
61.450	0.1115
61.451	0.11142
61.451	0.11135
61.451	0.11127
61.451	0.11119
61.451	0.1111
61.451	0.11101
61.451	0.11092
61.452	0.11083
61.452	0.11073
61.452	0.11063
61.452	0.11052
61.452	0.11041
61.452	0.1103
61.452	0.11019
61.453	0.11007
61.453	0.10995
61.453	0.10983
61.453	0.1097

61.453	0.10957
61.453	0.10944
61.454	0.10931
61.454	0.10917
61.454	0.10904
61.454	0.10889
61.454	0.10875
61.454	0.10861
61.454	0.10846
61.455	0.10831
61.455	0.10816
61.455	0.108
61.455	0.10784
61.455	0.10769
61.455	0.10753
61.456	0.10737
61.456	0.1072
61.456	0.10704
61.456	0.10687
61.456	0.1067
61.456	0.10653
61.456	0.10636
61.457	0.10619
61.457	0.10601
61.457	0.10584
61.457	0.10566
61.457	0.10548
61.457	0.1053
61.457	0.10512
61.458	0.10494
61.458	0.10476
61.458	0.10457
61.458	0.10439
61.458	0.10421
61.458	0.10402
61.459	0.10383
61.459	0.10365
61.459	0.10346
61.459	0.10327
61.459	0.10309
61.459	0.1029
61.459	0.10271
61.460	0.10255
61.460	0.10241
61.460	0.10228
61.460	0.10215
61.460	0.10201
61.460	0.10187
61.460	0.10173
61.461	0.10159
61.461	0.10145
61.461	0.10148
61.461	0.10161

61.461	0.10174
61.461	0.10187
61.462	0.10199
61.462	0.10212
61.462	0.10224
61.462	0.10235
61.462	0.10247
61.462	0.10258
61.462	0.10269
61.463	0.10279
61.463	0.10289
61.463	0.10299
61.463	0.10308
61.463	0.10317
61.463	0.10326
61.464	0.10334
61.464	0.10341
61.464	0.10349
61.464	0.10356
61.464	0.10362
61.464	0.10369
61.464	0.10374
61.465	0.1038
61.465	0.10385
61.465	0.1039
61.465	0.10394
61.465	0.10398
61.465	0.10401
61.465	0.10404
61.466	0.10407
61.466	0.10409
61.466	0.10411
61.466	0.10413
61.466	0.10414
61.466	0.10415
61.467	0.10416
61.467	0.10416
61.467	0.10416
61.467	0.10415
61.467	0.10415
61.467	0.10414
61.467	0.10412
61.468	0.10411
61.468	0.10409
61.468	0.10407
61.468	0.10404
61.468	0.10402
61.468	0.10405
61.469	0.10408
61.469	0.1041
61.469	0.10413
61.469	0.10414
61.469	0.10416

61.469	0.10417
61.469	0.10418
61.470	0.10419
61.470	0.10419
61.470	0.10419
61.470	0.10419
61.470	0.10419
61.470	0.10418
61.471	0.10417
61.471	0.10415
61.471	0.10413
61.471	0.10411
61.471	0.10409
61.471	0.10406
61.471	0.10403
61.472	0.10399
61.472	0.10396
61.472	0.10392
61.472	0.10387
61.472	0.10383
61.472	0.10378
61.472	0.10372
61.473	0.10367
61.473	0.10361
61.473	0.10354
61.473	0.10348
61.473	0.10341
61.473	0.10334
61.474	0.10326
61.474	0.10318
61.474	0.1031
61.474	0.10302
61.474	0.10293
61.474	0.10284
61.474	0.10275
61.475	0.10266
61.475	0.10256
61.475	0.10246
61.475	0.10236
61.475	0.10225
61.475	0.10214
61.476	0.10203
61.476	0.10192
61.476	0.1018
61.476	0.10169
61.476	0.10157
61.476	0.10145
61.476	0.10132
61.477	0.1012
61.477	0.10107
61.477	0.10094
61.477	0.10081
61.477	0.10068

61.477	0.10054
61.478	0.10041
61.478	0.10027
61.478	0.10013
61.478	0.1
61.478	0.09985
61.478	0.09971
61.478	0.09957
61.479	0.09943
61.479	0.09928
61.479	0.09914
61.479	0.09899
61.479	0.09885
61.479	0.0987
61.480	0.09855
61.480	0.0984
61.480	0.09825
61.480	0.0981
61.480	0.09795
61.480	0.09781
61.480	0.09769
61.481	0.09756
61.481	0.09744
61.481	0.09731
61.481	0.09721
61.481	0.09766
61.481	0.09811
61.482	0.09855
61.482	0.09899
61.482	0.09943
61.482	0.09986
61.482	0.10028
61.482	0.1007
61.482	0.10111
61.483	0.10151
61.483	0.10191
61.483	0.10229
61.483	0.10267
61.483	0.10304
61.483	0.10339
61.483	0.10374
61.484	0.10407
61.484	0.1044
61.484	0.10471
61.484	0.10501
61.484	0.1053
61.484	0.10557
61.485	0.10584
61.485	0.1061
61.485	0.10634
61.485	0.10657
61.485	0.10679
61.485	0.10699

61.485	0.10718
61.486	0.10735
61.486	0.10751
61.486	0.10766
61.486	0.10779
61.486	0.10791
61.486	0.10802
61.487	0.10811
61.487	0.10819
61.487	0.10826
61.487	0.10832
61.487	0.10837
61.487	0.10841
61.487	0.10844
61.488	0.10845
61.488	0.10846
61.488	0.10846
61.488	0.10844
61.488	0.10842
61.488	0.10839
61.489	0.10835
61.489	0.1083
61.489	0.10825
61.489	0.10819
61.489	0.10812
61.489	0.10804
61.490	0.10795
61.490	0.10786
61.490	0.10777
61.490	0.10766
61.490	0.10756
61.490	0.10745
61.490	0.10733
61.491	0.10721
61.491	0.10708
61.491	0.10696
61.491	0.10684
61.491	0.10671
61.491	0.10657
61.492	0.10644
61.492	0.1063
61.492	0.10616
61.492	0.10602
61.492	0.10588
61.492	0.10573
61.492	0.10558
61.493	0.10543
61.493	0.10528
61.493	0.10513
61.493	0.10498
61.493	0.10483
61.493	0.10468
61.494	0.10453

61.494	0.10437
61.494	0.10422
61.494	0.10407
61.494	0.10391
61.494	0.10376
61.495	0.1036
61.495	0.10345
61.495	0.10329
61.495	0.10314
61.495	0.10308
61.495	0.10305
61.495	0.10303
61.496	0.10303
61.496	0.10303
61.496	0.10304
61.496	0.10307
61.496	0.10312
61.496	0.10316
61.497	0.10322
61.497	0.10329
61.497	0.10338
61.497	0.10347
61.497	0.10359
61.497	0.10371
61.498	0.10384
61.498	0.10399
61.498	0.10414
61.498	0.10432
61.498	0.10449
61.498	0.10469
61.498	0.10489
61.499	0.10511
61.499	0.10534
61.499	0.10557
61.499	0.10581
61.499	0.10607
61.499	0.10633
61.500	0.1066
61.500	0.10688
61.500	0.10716
61.500	0.10745
61.500	0.10776
61.500	0.10807
61.501	0.10839
61.501	0.10871
61.501	0.10905
61.501	0.10939
61.501	0.10973
61.501	0.11007
61.501	0.11041
61.502	0.11075
61.502	0.11108
61.502	0.11142

61.502	0.11175
61.502	0.11208
61.502	0.1124
61.503	0.11272
61.503	0.11304
61.503	0.11336
61.503	0.11366
61.503	0.11397
61.503	0.11427
61.504	0.11456
61.504	0.11485
61.504	0.11514
61.504	0.11541
61.504	0.11568
61.504	0.11594
61.505	0.1162
61.505	0.11645
61.505	0.1167
61.505	0.11693
61.505	0.11716
61.505	0.11738
61.505	0.11759
61.506	0.11779
61.506	0.11798
61.506	0.11816
61.506	0.11833
61.506	0.11849
61.506	0.11864
61.507	0.11878
61.507	0.11891
61.507	0.11903
61.507	0.11914
61.507	0.11923
61.507	0.11932
61.508	0.1194
61.508	0.11946
61.508	0.11952
61.508	0.11967
61.508	0.11986
61.508	0.12005
61.509	0.12022
61.509	0.12038
61.509	0.12053
61.509	0.12067
61.509	0.12079
61.509	0.1209
61.510	0.121
61.510	0.12109
61.510	0.12116
61.510	0.12122
61.510	0.12127
61.510	0.1213
61.511	0.12132

61.511	0.12133
61.511	0.12133
61.511	0.12131
61.511	0.12128
61.511	0.12124
61.511	0.12119
61.512	0.12113
61.512	0.12107
61.512	0.12101
61.512	0.12094
61.512	0.12088
61.512	0.12081
61.513	0.12074
61.513	0.12067
61.513	0.12058
61.513	0.12049
61.513	0.12039
61.513	0.12028
61.514	0.12017
61.514	0.12005
61.514	0.11992
61.514	0.11978
61.514	0.11964
61.514	0.11949
61.515	0.11933
61.515	0.11917
61.515	0.11901
61.515	0.11883
61.515	0.11865
61.515	0.11846
61.516	0.11827
61.516	0.11807
61.516	0.11787
61.516	0.11766
61.516	0.11745
61.516	0.11723
61.517	0.117
61.517	0.11677
61.517	0.11654
61.517	0.1163
61.517	0.11605
61.517	0.11581
61.518	0.11555
61.518	0.11529
61.518	0.11503
61.518	0.11477
61.518	0.11449
61.518	0.11422
61.519	0.11394
61.519	0.11366
61.519	0.11338
61.519	0.11309
61.519	0.11279

61.519	0.1125
61.520	0.1122
61.520	0.1119
61.520	0.1116
61.520	0.11129
61.520	0.11098
61.520	0.11068
61.521	0.11037
61.521	0.11006
61.521	0.10975
61.521	0.10943
61.521	0.10912
61.521	0.1088
61.522	0.10848
61.522	0.10816
61.522	0.10784
61.522	0.10751
61.522	0.10719
61.522	0.10686
61.523	0.10653
61.523	0.1062
61.523	0.10588
61.523	0.10555
61.523	0.10524
61.523	0.10499
61.524	0.10476
61.524	0.10455
61.524	0.10435
61.524	0.10451
61.524	0.10518
61.524	0.10464
61.525	0.10373
61.525	0.1036
61.525	0.10347
61.525	0.10335
61.525	0.10354
61.525	0.10377
61.526	0.10359
61.526	0.10317
61.526	0.10298
61.526	0.10293
61.526	0.10288
61.526	0.10283
61.527	0.10279
61.527	0.10276
61.527	0.10273
61.527	0.10276
61.527	0.1028
61.527	0.10286
61.528	0.10294
61.528	0.10302
61.528	0.1031
61.528	0.10318

61.528	0.10326
61.528	0.10333
61.529	0.1034
61.529	0.10347
61.529	0.10353
61.529	0.10359
61.529	0.10365
61.529	0.1037
61.530	0.10374
61.530	0.10378
61.530	0.10381
61.530	0.10384
61.530	0.10386
61.530	0.10387
61.531	0.10388
61.531	0.10388
61.531	0.10387
61.531	0.10385
61.531	0.10383
61.531	0.1038
61.532	0.10375
61.532	0.10371
61.532	0.10365
61.532	0.10358
61.532	0.10351
61.532	0.10342
61.533	0.10333
61.533	0.10323
61.533	0.10312
61.533	0.103
61.533	0.10287
61.533	0.10274
61.534	0.10259
61.534	0.10244
61.534	0.10228
61.534	0.10211
61.534	0.10194
61.534	0.10176
61.535	0.10158
61.535	0.10138
61.535	0.10118
61.535	0.10098
61.535	0.10077
61.536	0.10055
61.536	0.10032
61.536	0.10009
61.536	0.09985
61.536	0.09961
61.536	0.09937
61.537	0.09911
61.537	0.09886
61.537	0.0986
61.537	0.09833

61.537	0.09806
61.537	0.09779
61.538	0.09752
61.538	0.09724
61.538	0.09696
61.538	0.09667
61.538	0.0964
61.538	0.09614
61.539	0.09588
61.539	0.09563
61.539	0.09538
61.539	0.09513
61.539	0.09489
61.539	0.09476
61.540	0.09477
61.540	0.09478
61.540	0.09479
61.540	0.09479
61.540	0.0948
61.540	0.0948
61.541	0.0948
61.541	0.0948
61.541	0.0948
61.541	0.0948
61.541	0.09479
61.542	0.09479
61.542	0.09478
61.542	0.09478
61.542	0.09477
61.542	0.09476
61.542	0.09475
61.543	0.09474
61.543	0.09473
61.543	0.09472
61.543	0.09471
61.543	0.09469
61.543	0.09468
61.544	0.09466
61.544	0.09465
61.544	0.09463
61.544	0.09461
61.544	0.0946
61.544	0.09458
61.545	0.09456
61.545	0.09454
61.545	0.09452
61.545	0.0945
61.545	0.09448
61.546	0.09446
61.546	0.09444
61.546	0.09442
61.546	0.0944
61.546	0.09438

61.546	0.09436
61.547	0.09434
61.547	0.09436
61.547	0.09439
61.547	0.09442
61.547	0.09444
61.547	0.09447
61.548	0.09449
61.548	0.09451
61.548	0.09454
61.548	0.09456
61.548	0.09458
61.548	0.0946
61.549	0.09461
61.549	0.09463
61.549	0.09465
61.549	0.09466
61.549	0.09467
61.550	0.09468
61.550	0.0947
61.550	0.09471
61.550	0.09471
61.550	0.09472
61.550	0.09473
61.551	0.09473
61.551	0.09474
61.551	0.09474
61.551	0.09474
61.551	0.09474
61.551	0.09474
61.552	0.09474
61.552	0.09474
61.552	0.09473
61.552	0.09473
61.552	0.09473
61.553	0.09472
61.553	0.09472
61.553	0.09472
61.553	0.09471
61.553	0.0947
61.553	0.09469
61.554	0.09468
61.554	0.09467
61.554	0.09466
61.554	0.09464
61.554	0.09462
61.554	0.09461
61.555	0.09459
61.555	0.09456
61.555	0.09454
61.555	0.09452
61.555	0.09449
61.556	0.09446

61.556	0.09444
61.556	0.09441
61.556	0.09437
61.556	0.09434
61.556	0.09431
61.557	0.09427
61.557	0.09424
61.557	0.0942
61.557	0.09416
61.557	0.09412
61.557	0.09407
61.558	0.09403
61.558	0.09398
61.558	0.09393
61.558	0.09388
61.558	0.09383
61.559	0.09378
61.559	0.09372
61.559	0.09367
61.559	0.09361
61.559	0.09355
61.559	0.09349
61.560	0.09343
61.560	0.09336
61.560	0.0933
61.560	0.09323
61.560	0.09316
61.561	0.09309
61.561	0.09302
61.561	0.09294
61.561	0.09287
61.561	0.09279
61.561	0.09272
61.562	0.09265
61.562	0.09257
61.562	0.0925
61.562	0.09242
61.562	0.09234
61.563	0.09226
61.563	0.09218
61.563	0.0921
61.563	0.09202
61.563	0.09193
61.563	0.09185
61.564	0.09257
61.564	0.09201
61.564	0.09158
61.564	0.09149
61.564	0.0914
61.565	0.0913
61.565	0.09121
61.565	0.09111
61.565	0.09101

61.565	0.09101
61.565	0.09123
61.566	0.09088
61.566	0.09087
61.566	0.09087
61.566	0.09086
61.566	0.09084
61.567	0.09173
61.567	0.09113
61.567	0.09111
61.567	0.0909
61.567	0.09073
61.567	0.09079
61.568	0.09068
61.568	0.09066
61.568	0.09063
61.568	0.09059
61.568	0.09056
61.569	0.0906
61.569	0.09267
61.569	0.092
61.569	0.09048
61.569	0.09036
61.569	0.09031
61.570	0.09027
61.570	0.09022
61.570	0.09017
61.570	0.09029
61.570	0.09007
61.571	0.09002
61.571	0.08997
61.571	0.08992
61.571	0.08986
61.571	0.09025
61.571	0.09
61.572	0.08969
61.572	0.08963
61.572	0.08957
61.572	0.08949
61.572	0.08951
61.573	0.09147
61.573	0.0908
61.573	0.08931
61.573	0.08926
61.573	0.08905
61.573	0.08897
61.574	0.0889
61.574	0.08882
61.574	0.08876
61.574	0.08911
61.574	0.09063
61.575	0.0899
61.575	0.08893

61.575	0.08861
61.575	0.08824
61.575	0.08813
61.576	0.08803
61.576	0.08794
61.576	0.08784
61.576	0.08898
61.576	0.08848
61.576	0.08754
61.577	0.08781
61.577	0.08748
61.577	0.08982
61.577	0.08961
61.577	0.08839
61.578	0.08754
61.578	0.08795
61.578	0.08726
61.578	0.08696
61.578	0.08691
61.578	0.08688
61.579	0.0877
61.579	0.08701
61.579	0.08679
61.579	0.08675
61.579	0.08672
61.580	0.08669
61.580	0.08666
61.580	0.08662
61.580	0.08659
61.580	0.08655
61.581	0.08689
61.581	0.08775
61.581	0.08823
61.581	0.08754
61.581	0.0865
61.581	0.08652
61.582	0.08654
61.582	0.08656
61.582	0.08832
61.582	0.08813
61.582	0.08753
61.583	0.08942
61.583	0.08929
61.583	0.08873
61.583	0.08881
61.583	0.08812
61.583	0.08664
61.584	0.08664
61.584	0.08664
61.584	0.08664
61.584	0.08664
61.584	0.08663
61.585	0.08663

61.585	0.08662
61.585	0.08661
61.585	0.0866
61.585	0.08659
61.585	0.08657
61.586	0.08656
61.586	0.08703
61.586	0.08652
61.586	0.0865
61.586	0.08763
61.587	0.09022
61.587	0.08945
61.587	0.08756
61.587	0.08639
61.587	0.08636
61.588	0.08633
61.588	0.0863
61.588	0.08627
61.588	0.08624
61.588	0.0862
61.588	0.08617
61.589	0.08719
61.589	0.08993
61.589	0.08924
61.589	0.08866
61.589	0.08795
61.590	0.08624
61.590	0.08591
61.590	0.08587
61.590	0.08583
61.590	0.08641
61.590	0.0872
61.591	0.08662
61.591	0.0857
61.591	0.08697
61.591	0.08627
61.591	0.08552
61.592	0.08548
61.592	0.08543
61.592	0.08539
61.592	0.08534
61.592	0.0853
61.593	0.08526
61.593	0.08522
61.593	0.08572
61.593	0.08513
61.593	0.08926
61.593	0.08973
61.594	0.08717
61.594	0.08496
61.594	0.08493
61.594	0.0849
61.594	0.08487

61.595	0.08484
61.595	0.08574
61.595	0.08833
61.595	0.08762
61.595	0.08607
61.595	0.08535
61.596	0.0868
61.596	0.08629
61.596	0.08678
61.596	0.08894
61.596	0.08783
61.597	0.08447
61.597	0.08638
61.597	0.08761
61.597	0.08628
61.597	0.08474
61.597	0.0844
61.598	0.08424
61.598	0.08581
61.598	0.08453
61.598	0.08934
61.598	0.08979
61.599	0.08741
61.599	0.08674
61.599	0.08508
61.599	0.08399
61.599	0.08397
61.600	0.08396
61.600	0.08408
61.600	0.08605
61.600	0.08542
61.600	0.08393
61.600	0.08499
61.601	0.0844
61.601	0.08385
61.601	0.08383
61.601	0.08382
61.601	0.0838
61.602	0.08379
61.602	0.08377
61.602	0.08378
61.602	0.08374
61.602	0.08372
61.602	0.0837
61.603	0.08369
61.603	0.08367
61.603	0.08365
61.603	0.08363
61.603	0.08361
61.604	0.08359
61.604	0.08358
61.604	0.08356
61.604	0.08354

61.604	0.08352
61.604	0.08433
61.605	0.08378
61.605	0.08346
61.605	0.08344
61.605	0.08341
61.605	0.08339
61.606	0.08337
61.606	0.08334
61.606	0.08332
61.606	0.08329
61.606	0.08326
61.606	0.08323
61.607	0.0832
61.607	0.08317
61.607	0.08314
61.607	0.08311
61.607	0.08307
61.608	0.08304
61.608	0.083
61.608	0.08297
61.608	0.08294
61.608	0.08501
61.608	0.08297
61.609	0.08521
61.609	0.08514
61.609	0.08455
61.609	0.08533
61.609	0.08339
61.610	0.08259
61.610	0.08255
61.610	0.0825
61.610	0.08245
61.610	0.0824
61.610	0.08235
61.611	0.08299
61.611	0.08224
61.611	0.08254
61.611	0.08235
61.611	0.08207
61.611	0.08201
61.612	0.08196
61.612	0.0819
61.612	0.08184
61.612	0.08178
61.612	0.08176
61.613	0.08175
61.613	0.08173
61.613	0.08171
61.613	0.0817
61.613	0.08168
61.613	0.08166
61.614	0.08164

61.614	0.08162
61.614	0.08159
61.614	0.08157
61.614	0.08154
61.615	0.08152
61.615	0.08149
61.615	0.08146
61.615	0.08143
61.615	0.0814
61.615	0.08137
61.616	0.08133
61.616	0.0813
61.616	0.08126
61.616	0.08122
61.616	0.0812
61.617	0.0812
61.617	0.08119
61.617	0.08118
61.617	0.08116
61.617	0.08115
61.617	0.08114
61.618	0.08112
61.618	0.0811
61.618	0.08108
61.618	0.08106
61.618	0.08104
61.618	0.08102
61.619	0.08099
61.619	0.08097
61.619	0.08094
61.619	0.08092
61.619	0.08089
61.620	0.08086
61.620	0.08158
61.620	0.0808
61.620	0.08183
61.620	0.08073
61.620	0.08222
61.621	0.08218
61.621	0.08064
61.621	0.08059
61.621	0.08056
61.621	0.08052
61.621	0.08048
61.622	0.08044
61.622	0.0804
61.622	0.08036
61.622	0.08032
61.622	0.08028
61.623	0.08023
61.623	0.08019
61.623	0.08076
61.623	0.08038

61.623	0.08006
61.623	0.08002
61.624	0.08071
61.624	0.0808
61.624	0.08029
61.624	0.08118
61.624	0.08107
61.625	0.07974
61.625	0.0797
61.625	0.08008
61.625	0.07962
61.625	0.08008
61.625	0.07951
61.626	0.07946
61.626	0.07942
61.626	0.07937
61.626	0.07932
61.626	0.07927
61.626	0.08018
61.627	0.07949
61.627	0.07914
61.627	0.0791
61.627	0.07944
61.627	0.07918
61.628	0.079
61.628	0.07896
61.628	0.07921
61.628	0.0789
61.628	0.07887
61.628	0.07884
61.629	0.07881
61.629	0.07879
61.629	0.07885
61.629	0.07873
61.629	0.07893
61.630	0.08042
61.630	0.07877
61.630	0.08071
61.630	0.07996
61.630	0.08114
61.630	0.08113
61.631	0.07875
61.631	0.0785
61.631	0.07847
61.631	0.07847
61.631	0.07899
61.631	0.07941
61.632	0.07877
61.632	0.07834
61.632	0.07831
61.632	0.07829
61.632	0.07826
61.633	0.07824

61.633	0.07851
61.633	0.07825
61.633	0.07814
61.633	0.07835
61.633	0.07808
61.634	0.07805
61.634	0.07802
61.634	0.07799
61.634	0.07796
61.634	0.07807
61.635	0.07929
61.635	0.07868
61.635	0.07784
61.635	0.07848
61.635	0.0781
61.635	0.07774
61.636	0.07853
61.636	0.07808
61.636	0.07765
61.636	0.07762
61.636	0.07758
61.636	0.07755
61.637	0.07789
61.637	0.07974
61.637	0.0793
61.637	0.07808
61.637	0.07738
61.638	0.07734
61.638	0.07731
61.638	0.078
61.638	0.07985
61.638	0.07943
61.638	0.07825
61.639	0.07873
61.639	0.0779
61.639	0.07819
61.639	0.07919
61.639	0.07873
61.640	0.07775
61.640	0.0791
61.640	0.07838
61.640	0.0771
61.640	0.07676
61.640	0.07671
61.641	0.07746
61.641	0.07852
61.641	0.07787
61.641	0.07726
61.641	0.07659
61.642	0.07695
61.642	0.07662
61.642	0.07645
61.642	0.07631

61.642	0.07673
61.642	0.0776
61.643	0.07736
61.643	0.07684
61.643	0.07607
61.643	0.07603
61.643	0.07609
61.644	0.07669
61.644	0.07642
61.644	0.07615
61.644	0.07791
61.644	0.07733
61.645	0.0785
61.645	0.07799
61.645	0.07645
61.645	0.07726
61.645	0.07839
61.645	0.07746
61.646	0.07773
61.646	0.07819
61.646	0.07729
61.646	0.07742
61.646	0.07675
61.647	0.07574
61.647	0.0761
61.647	0.07586
61.647	0.0765
61.647	0.07608
61.647	0.07568
61.648	0.07572
61.648	0.07885
61.648	0.0772
61.648	0.07841
61.648	0.07775
61.649	0.07632
61.649	0.07568
61.649	0.07595
61.649	0.07562
61.649	0.07624
61.650	0.07559
61.650	0.07519
61.650	0.07523
61.650	0.07648
61.650	0.07563
61.650	0.07661
61.651	0.07604
61.651	0.07539
61.651	0.07508
61.651	0.07502
61.651	0.075
61.652	0.07499
61.652	0.07497
61.652	0.07552

61.652	0.07622
61.652	0.07555
61.653	0.07533
61.653	0.0755
61.653	0.07554
61.653	0.07525
61.653	0.0748
61.654	0.07479
61.654	0.07471
61.654	0.07485
61.654	0.07498
61.654	0.07495
61.654	0.07554
61.655	0.07469
61.655	0.07443
61.655	0.07438
61.655	0.07471
61.655	0.07512
61.656	0.07444
61.656	0.07422
61.656	0.07418
61.656	0.0744
61.656	0.07413
61.657	0.07458
61.657	0.07488
61.657	0.07468
61.657	0.0747
61.657	0.07438
61.658	0.07473
61.658	0.0741
61.658	0.07459
61.658	0.07431
61.658	0.07389
61.659	0.07384
61.659	0.0737
61.659	0.07365
61.659	0.07354
61.659	0.07376
61.659	0.07433
61.660	0.07385
61.660	0.07466
61.660	0.07422
61.660	0.07387
61.660	0.07443
61.661	0.07375
61.661	0.07354
61.661	0.0733
61.661	0.07334
61.661	0.07335
61.662	0.07335
61.662	0.07335
61.662	0.07335
61.662	0.07336

61.662	0.07337
61.663	0.07338
61.663	0.07339
61.663	0.0734
61.663	0.07341
61.663	0.07342
61.664	0.07343
61.664	0.07344
61.664	0.07357
61.664	0.07361
61.664	0.07355
61.665	0.07347
61.665	0.07347
61.665	0.07379
61.665	0.07398
61.665	0.074
61.666	0.0739
61.666	0.07378
61.666	0.0738
61.666	0.07377
61.666	0.07373
61.666	0.07371
61.667	0.07367
61.667	0.07369
61.667	0.0737
61.667	0.07372
61.667	0.07373
61.668	0.07374
61.668	0.07377
61.668	0.07402
61.668	0.07386
61.668	0.07387
61.669	0.0739
61.669	0.07393
61.669	0.07396
61.669	0.07398
61.669	0.07401
61.670	0.07404
61.670	0.07406
61.670	0.07428
61.670	0.07412
61.670	0.07414
61.671	0.07417
61.671	0.07419
61.671	0.07421
61.671	0.07424
61.671	0.07426
61.672	0.07428
61.672	0.0743
61.672	0.07432
61.672	0.07434
61.672	0.07447
61.673	0.07437

61.673	0.07439
61.673	0.07441
61.673	0.07442
61.673	0.07444
61.674	0.07445
61.674	0.07446
61.674	0.07448
61.674	0.07449
61.674	0.0745
61.675	0.07451
61.675	0.07452
61.675	0.07452
61.675	0.07453
61.675	0.07453
61.676	0.07453
61.676	0.07455
61.676	0.07454
61.676	0.07453
61.676	0.07453
61.677	0.07453
61.677	0.07453
61.677	0.07452
61.677	0.07452
61.677	0.07451
61.677	0.0745
61.678	0.07449
61.678	0.07448
61.678	0.07447
61.678	0.07446
61.678	0.07444
61.679	0.07443
61.679	0.07441
61.679	0.07439
61.679	0.07437
61.679	0.07435
61.680	0.07433
61.680	0.07431
61.680	0.07429
61.680	0.07427
61.680	0.07424
61.681	0.07422
61.681	0.07419
61.681	0.07416
61.681	0.07413
61.681	0.0741
61.682	0.07407
61.682	0.07404
61.682	0.074
61.682	0.07397
61.682	0.07393
61.683	0.07389
61.683	0.07386
61.683	0.07382

61.683	0.07378
61.683	0.07374
61.684	0.07369
61.684	0.07365
61.684	0.07361
61.684	0.07356
61.684	0.07352
61.685	0.07347
61.685	0.07342
61.685	0.07337
61.685	0.07332
61.685	0.07327
61.686	0.07322
61.686	0.07317
61.686	0.07312
61.686	0.07306
61.686	0.07301
61.687	0.07295
61.687	0.0729
61.687	0.07284
61.687	0.07279
61.687	0.07273
61.687	0.07268
61.688	0.07265
61.688	0.07261
61.688	0.07258
61.688	0.07254
61.688	0.07251
61.689	0.07247
61.689	0.07243
61.689	0.07239
61.689	0.07235
61.689	0.07231
61.690	0.07228
61.690	0.07223
61.690	0.07219
61.690	0.07215
61.690	0.07211
61.691	0.07207
61.691	0.07203
61.691	0.07198
61.691	0.07194
61.691	0.0719
61.692	0.07185
61.692	0.07181
61.692	0.07176
61.692	0.07172
61.692	0.07167
61.693	0.07163
61.693	0.07158
61.693	0.07153
61.693	0.07149
61.693	0.07144

61.694	0.07139
61.694	0.07134
61.694	0.0713
61.694	0.07125
61.694	0.07121
61.694	0.07116
61.695	0.07111
61.695	0.07107
61.695	0.07102
61.695	0.07097
61.695	0.07092
61.696	0.07088
61.696	0.07085
61.696	0.07086
61.696	0.07087
61.696	0.07087
61.697	0.07088
61.697	0.07089
61.697	0.07089
61.697	0.0709
61.697	0.07091
61.698	0.07091
61.698	0.07091
61.698	0.07092
61.698	0.07092
61.698	0.07092
61.699	0.07093
61.699	0.07094
61.699	0.07094
61.699	0.07095
61.699	0.07095
61.700	0.07096
61.700	0.07096
61.700	0.07096
61.700	0.07097
61.700	0.07097
61.701	0.07097
61.701	0.07097
61.701	0.07097
61.701	0.07097
61.701	0.07096
61.702	0.07096
61.702	0.07096
61.702	0.07095
61.702	0.07094
61.702	0.07094
61.702	0.07093
61.703	0.07092
61.703	0.07091
61.703	0.0709
61.703	0.07089
61.703	0.07087
61.704	0.07086

61.704	0.07084
61.704	0.07083
61.704	0.07081
61.704	0.07079
61.705	0.07077
61.705	0.07078
61.705	0.07079
61.705	0.0708
61.705	0.07081
61.706	0.07081
61.706	0.07082
61.706	0.07082
61.706	0.07083
61.706	0.07083
61.707	0.07083
61.707	0.07083
61.707	0.07083
61.707	0.07083
61.707	0.07082
61.708	0.07082
61.708	0.07081
61.708	0.07081
61.708	0.0708
61.708	0.07079
61.709	0.07078
61.709	0.07077
61.709	0.07076
61.709	0.07074
61.709	0.07073
61.710	0.07071
61.710	0.0707
61.710	0.07068
61.710	0.07066
61.710	0.07064
61.710	0.07062
61.711	0.0706
61.711	0.07057
61.711	0.07055
61.711	0.07053
61.711	0.0705
61.712	0.07047
61.712	0.07045
61.712	0.07042
61.712	0.07039
61.712	0.07036
61.713	0.07033
61.713	0.0703
61.713	0.07027
61.713	0.07024
61.713	0.0702
61.714	0.07017
61.714	0.07013
61.714	0.0701

61.714	0.07006
61.714	0.07002
61.715	0.06998
61.715	0.06994
61.715	0.0699
61.715	0.06986
61.715	0.06981
61.716	0.06977
61.716	0.06973
61.716	0.06968
61.716	0.06964
61.716	0.06959
61.717	0.06954
61.717	0.0695
61.717	0.06945
61.717	0.0694
61.717	0.06935
61.718	0.0693
61.718	0.06925
61.718	0.0692
61.718	0.06915
61.718	0.0691
61.719	0.06905
61.719	0.069
61.719	0.06895
61.719	0.06894
61.719	0.06895
61.719	0.06896
61.720	0.06897
61.720	0.06898
61.720	0.06898
61.720	0.06899
61.720	0.069
61.721	0.069
61.721	0.06901
61.721	0.06901
61.721	0.06901
61.721	0.06901
61.722	0.06901
61.722	0.06901
61.722	0.06901
61.722	0.06901
61.722	0.06901
61.723	0.069
61.723	0.069
61.723	0.06899
61.723	0.06898
61.723	0.06898
61.724	0.06897
61.724	0.06896
61.724	0.06895
61.724	0.06894
61.724	0.06892

61.725	0.06891
61.725	0.0689
61.725	0.06888
61.725	0.06886
61.725	0.06885
61.726	0.06883
61.726	0.06881
61.726	0.06879
61.726	0.06877
61.726	0.06875
61.727	0.06872
61.727	0.0687
61.727	0.06868
61.727	0.06865
61.727	0.06862
61.728	0.0686
61.728	0.06857
61.728	0.06854
61.728	0.06851
61.728	0.06848
61.729	0.06845
61.729	0.06841
61.729	0.06838
61.729	0.06834
61.729	0.06831
61.730	0.06827
61.730	0.06823
61.730	0.06819
61.730	0.06816
61.730	0.06812
61.731	0.06807
61.731	0.06803
61.731	0.06799
61.731	0.06798
61.731	0.06798
61.732	0.06797
61.732	0.06797
61.732	0.06796
61.732	0.06795
61.732	0.06794
61.733	0.06793
61.733	0.06792
61.733	0.0679
61.733	0.06789
61.733	0.06788
61.734	0.06786
61.734	0.06784
61.734	0.06783
61.734	0.06781
61.734	0.06779
61.735	0.06777
61.735	0.06775
61.735	0.06772

61.735	0.0677
61.735	0.06768
61.736	0.06765
61.736	0.06763
61.736	0.0676
61.736	0.06757
61.736	0.06754
61.737	0.06751
61.737	0.06748
61.737	0.06745
61.737	0.06742
61.737	0.06738
61.738	0.06735
61.738	0.06731
61.738	0.06728
61.738	0.06724
61.738	0.0672
61.739	0.06717
61.739	0.06713
61.739	0.06709
61.739	0.06704
61.739	0.067
61.740	0.06696
61.740	0.06692
61.740	0.06687
61.740	0.06683
61.740	0.06678
61.741	0.06674
61.741	0.06669
61.741	0.0669
61.741	0.0666
61.741	0.06655
61.742	0.0665
61.742	0.06645
61.742	0.0664
61.742	0.06688
61.742	0.0663
61.743	0.06624
61.743	0.06619
61.743	0.06614
61.743	0.06608
61.743	0.06683
61.744	0.06601
61.744	0.06592
61.744	0.06591
61.744	0.06593
61.744	0.06671
61.745	0.06596
61.745	0.06597
61.745	0.06598
61.745	0.066
61.745	0.06657
61.746	0.06601

61.746	0.06602
61.746	0.06603
61.746	0.06603
61.746	0.06651
61.747	0.06603
61.747	0.06603
61.747	0.06603
61.747	0.06663
61.747	0.06603
61.748	0.06602
61.748	0.06601
61.748	0.0663
61.748	0.06599
61.748	0.06643
61.749	0.06682
61.749	0.06595
61.749	0.06616
61.749	0.06592
61.749	0.06743
61.750	0.06715
61.750	0.06586
61.750	0.06584
61.750	0.06581
61.750	0.06578
61.751	0.06576
61.751	0.06573
61.751	0.06569
61.751	0.06566
61.751	0.06563
61.752	0.06559
61.752	0.06555
61.752	0.06552
61.752	0.06547
61.752	0.06543
61.753	0.06539
61.753	0.06534
61.753	0.0653
61.753	0.06525
61.753	0.0652
61.754	0.06515
61.754	0.0651
61.754	0.06504
61.754	0.06499
61.755	0.06493
61.755	0.06487
61.755	0.06481
61.755	0.06475
61.755	0.06469
61.756	0.06462
61.756	0.06456
61.756	0.06449
61.756	0.06442
61.756	0.06435

61.757	0.06428
61.757	0.06421
61.757	0.06413
61.757	0.06406
61.757	0.06398
61.758	0.06391
61.758	0.06383
61.758	0.06375
61.758	0.06367
61.758	0.06364
61.759	0.06361
61.759	0.06358
61.759	0.06355
61.759	0.06352
61.759	0.06348
61.760	0.06345
61.760	0.06341
61.760	0.06338
61.760	0.06334
61.760	0.0633
61.761	0.06326
61.761	0.06322
61.761	0.06318
61.761	0.06314
61.761	0.06309
61.762	0.06305
61.762	0.06301
61.762	0.06296
61.762	0.06291
61.762	0.06287
61.763	0.06282
61.763	0.06277
61.763	0.06272
61.763	0.06267
61.763	0.06261
61.764	0.06256
61.764	0.06251
61.764	0.06245
61.764	0.0624
61.764	0.06234
61.765	0.06228
61.765	0.06223
61.765	0.06217
61.765	0.06211
61.765	0.06205
61.766	0.06199
61.766	0.06192
61.766	0.06186
61.766	0.06182
61.766	0.06181
61.767	0.06179
61.767	0.06177
61.767	0.06176

61.767	0.06174
61.767	0.06172
61.768	0.0617
61.768	0.06167
61.768	0.06165
61.768	0.06163
61.769	0.06161
61.769	0.06158
61.769	0.06155
61.769	0.06153
61.769	0.0615
61.770	0.06147
61.770	0.06144
61.770	0.06142
61.770	0.06139
61.770	0.06135
61.771	0.06132
61.771	0.06129
61.771	0.06126
61.771	0.06122
61.771	0.06119
61.772	0.06115
61.772	0.06112
61.772	0.06108
61.772	0.06104
61.773	0.061
61.773	0.06097
61.773	0.06093
61.773	0.06089
61.773	0.06085
61.774	0.0608
61.774	0.06076
61.774	0.06072
61.774	0.06068
61.774	0.06063
61.775	0.06059
61.775	0.06054
61.775	0.0605
61.775	0.06045
61.776	0.0604
61.776	0.06036
61.776	0.06031
61.776	0.06041
61.776	0.06148
61.777	0.06116
61.777	0.06047
61.777	0.06041
61.777	0.06044
61.777	0.06047
61.778	0.0605
61.778	0.06054
61.778	0.06084
61.778	0.0606

61.779	0.06062
61.779	0.06065
61.779	0.06068
61.779	0.06125
61.779	0.06073
61.780	0.06076
61.780	0.06078
61.780	0.06081
61.780	0.06083
61.781	0.06086
61.781	0.06088
61.781	0.0609
61.781	0.06092
61.781	0.06094
61.782	0.06097
61.782	0.06103
61.782	0.06109
61.782	0.06116
61.783	0.06122
61.783	0.06129
61.783	0.06135
61.783	0.06142
61.783	0.06149
61.784	0.06155
61.784	0.06162
61.784	0.06168
61.784	0.06175
61.785	0.06181
61.785	0.06187
61.785	0.06193
61.785	0.06199
61.786	0.06205
61.786	0.06211
61.786	0.06216
61.786	0.06222
61.786	0.06227
61.787	0.06232
61.787	0.06237
61.787	0.06242
61.787	0.06246
61.788	0.06251
61.788	0.06255
61.788	0.06259
61.788	0.06263
61.788	0.06267
61.789	0.0627
61.789	0.06274
61.789	0.06277
61.789	0.0628
61.790	0.06283
61.790	0.06285
61.790	0.06288
61.790	0.0629

61.791	0.06312
61.791	0.06294
61.791	0.06296
61.791	0.06297
61.792	0.06299
61.792	0.063
61.792	0.06301
61.792	0.06302
61.792	0.06303
61.793	0.06339
61.793	0.06304
61.793	0.06304
61.793	0.06304
61.794	0.06304
61.794	0.06304
61.794	0.06304
61.794	0.06304
61.795	0.06304
61.795	0.06304
61.795	0.06305
61.795	0.06309
61.795	0.06314
61.796	0.06319
61.796	0.06324
61.796	0.06329
61.796	0.06334
61.797	0.06338
61.797	0.06343
61.797	0.06348
61.797	0.06352
61.798	0.06357
61.798	0.06361
61.798	0.06365
61.798	0.0637
61.799	0.06374
61.799	0.06378
61.799	0.06382
61.799	0.06386
61.800	0.0639
61.800	0.06393
61.800	0.06397
61.800	0.064
61.800	0.06404
61.801	0.06407
61.801	0.0641
61.801	0.06414
61.801	0.06417
61.802	0.06419
61.802	0.06422
61.802	0.06425
61.802	0.06427
61.803	0.0643
61.803	0.06432

61.803	0.06434
61.803	0.06436
61.804	0.06437
61.804	0.06439
61.804	0.06441
61.804	0.06442
61.805	0.06443
61.805	0.06444
61.805	0.06445
61.805	0.06445
61.806	0.06446
61.806	0.06446
61.806	0.06446
61.806	0.06448
61.807	0.06458
61.807	0.06468
61.807	0.06478
61.807	0.06488
61.807	0.06497
61.808	0.06506
61.808	0.06515
61.808	0.06524
61.808	0.06533
61.809	0.06541
61.809	0.06549
61.809	0.06557
61.809	0.06565
61.810	0.06572
61.810	0.06579
61.810	0.06586
61.810	0.06593
61.811	0.06599
61.811	0.06605
61.811	0.06611
61.811	0.06616
61.812	0.06621
61.812	0.06626
61.812	0.0663
61.812	0.06634
61.813	0.06637
61.813	0.06641
61.813	0.06644
61.813	0.06646
61.814	0.06648
61.814	0.0665
61.814	0.06651
61.814	0.06652
61.815	0.06653
61.815	0.06653
61.815	0.06653
61.815	0.06652
61.815	0.06651
61.816	0.0665

61.816	0.06648
61.816	0.06646
61.816	0.06643
61.817	0.0664
61.817	0.06637
61.817	0.06633
61.817	0.06629
61.818	0.06624
61.818	0.06619
61.818	0.06614
61.818	0.06608
61.819	0.06602
61.819	0.06596
61.819	0.06589
61.819	0.06582
61.820	0.06575
61.820	0.06567
61.820	0.06559
61.820	0.06551
61.821	0.06542
61.821	0.06533
61.821	0.06524
61.821	0.06514
61.822	0.06504
61.822	0.06494
61.822	0.06484
61.822	0.06473
61.823	0.06462
61.823	0.06451
61.823	0.06439
61.823	0.06428
61.824	0.06416
61.824	0.06403
61.824	0.06391
61.824	0.06378
61.825	0.06365
61.825	0.06352
61.825	0.06339
61.825	0.06325
61.826	0.06312
61.826	0.06298
61.826	0.06311
61.826	0.06346
61.826	0.06265
61.827	0.06263
61.827	0.06273
61.827	0.06285
61.827	0.06298
61.828	0.06312
61.828	0.06327
61.828	0.06343
61.828	0.0636
61.829	0.06376

61.829	0.06392
61.829	0.06408
61.829	0.06424
61.830	0.0644
61.830	0.06455
61.830	0.0647
61.830	0.06485
61.831	0.06499
61.831	0.06512
61.831	0.06525
61.831	0.06538
61.832	0.06551
61.832	0.06562
61.832	0.06574
61.832	0.06584
61.833	0.06594
61.833	0.06604
61.833	0.06613
61.833	0.06621
61.834	0.06629
61.834	0.06636
61.834	0.06642
61.834	0.06648
61.835	0.06653
61.835	0.06657
61.835	0.0666
61.835	0.06663
61.836	0.06665
61.836	0.06667
61.836	0.06667
61.836	0.06668
61.837	0.06667
61.837	0.06666
61.837	0.06664
61.837	0.06661
61.838	0.06658
61.838	0.06654
61.838	0.06649
61.838	0.06644
61.839	0.06638
61.839	0.06631
61.839	0.06624
61.839	0.06617
61.840	0.06608
61.840	0.066
61.840	0.0659
61.840	0.0658
61.841	0.0657
61.841	0.06559
61.841	0.06547
61.841	0.06535
61.842	0.06523
61.842	0.0651

61.842	0.06497
61.842	0.06484
61.843	0.0647
61.843	0.06455
61.843	0.06441
61.843	0.06426
61.844	0.0641
61.844	0.06395
61.844	0.06379
61.844	0.06363
61.845	0.06346
61.845	0.06344
61.845	0.06366
61.845	0.06296
61.846	0.06279
61.846	0.06261
61.846	0.06361
61.846	0.06387
61.847	0.06238
61.847	0.06202
61.847	0.06196
61.847	0.0619
61.848	0.06368
61.848	0.06439
61.848	0.06294
61.848	0.06272
61.849	0.06298
61.849	0.06195
61.849	0.06219
61.849	0.06212
61.850	0.0622
61.850	0.06228
61.850	0.06236
61.850	0.06244
61.851	0.06252
61.851	0.06259
61.851	0.06267
61.851	0.06274
61.852	0.0628
61.852	0.06324
61.852	0.06381
61.852	0.06299
61.853	0.06305
61.853	0.0631
61.853	0.06314
61.854	0.06319
61.854	0.06323
61.854	0.06326
61.854	0.06329
61.855	0.06331
61.855	0.06333
61.855	0.06335
61.855	0.06336

61.856	0.06336
61.856	0.06336
61.856	0.06336
61.856	0.06336
61.857	0.0634
61.857	0.06344
61.857	0.06347
61.857	0.0635
61.858	0.06352
61.858	0.06353
61.858	0.06354
61.858	0.06355
61.859	0.06354
61.859	0.06353
61.859	0.06352
61.860	0.0635
61.860	0.06347
61.860	0.06343
61.860	0.06339
61.861	0.06335
61.861	0.0633
61.861	0.06324
61.861	0.06317
61.862	0.0631
61.862	0.06303
61.862	0.06294
61.862	0.06286
61.863	0.06276
61.863	0.06267
61.863	0.06256
61.863	0.06245
61.864	0.06234
61.864	0.06222
61.864	0.0621
61.865	0.06197
61.865	0.06184
61.865	0.06177
61.865	0.06173
61.866	0.06169
61.866	0.06164
61.866	0.06159
61.866	0.06153
61.867	0.06148
61.867	0.06142
61.867	0.06135
61.867	0.06128
61.868	0.06121
61.868	0.06114
61.868	0.06107
61.869	0.06099
61.869	0.06091
61.869	0.06083
61.869	0.06074

61.870	0.06066
61.870	0.06057
61.870	0.06048
61.870	0.06038
61.871	0.06029
61.871	0.06019
61.871	0.0601
61.871	0.06
61.872	0.0599
61.872	0.05979
61.872	0.05969
61.873	0.05958
61.873	0.05968
61.873	0.05937
61.873	0.05926
61.874	0.05915
61.874	0.05917
61.874	0.05893
61.874	0.05882
61.875	0.05878
61.875	0.0586
61.875	0.05898
61.875	0.05899
61.876	0.05869
61.876	0.05855
61.876	0.05869
61.877	0.05848
61.877	0.05847
61.877	0.05846
61.877	0.05844
61.878	0.05842
61.878	0.0584
61.878	0.05838
61.878	0.05836
61.879	0.05836
61.879	0.05842
61.879	0.05848
61.880	0.05853
61.880	0.05858
61.880	0.05862
61.880	0.05866
61.881	0.0587
61.881	0.05872
61.881	0.05875
61.881	0.05877
61.882	0.05878
61.882	0.05968
61.882	0.05943
61.882	0.05879
61.883	0.0594
61.883	0.05877
61.883	0.05939
61.884	0.05872

61.884	0.05869
61.884	0.05865
61.884	0.05861
61.885	0.05856
61.885	0.05851
61.885	0.05845
61.885	0.05838
61.886	0.05831
61.886	0.05824
61.886	0.05816
61.887	0.05808
61.887	0.05799
61.887	0.0579
61.887	0.0578
61.888	0.0577
61.888	0.05759
61.888	0.05748
61.888	0.05737
61.889	0.05725
61.889	0.05712
61.889	0.057
61.890	0.05687
61.890	0.05673
61.890	0.05659
61.890	0.05645
61.891	0.05631
61.891	0.05617
61.891	0.05626
61.892	0.05622
61.892	0.05572
61.892	0.05619
61.892	0.05653
61.893	0.05537
61.893	0.05528
61.893	0.05519
61.893	0.0551
61.894	0.05503
61.894	0.05497
61.894	0.05491
61.895	0.05485
61.895	0.05479
61.895	0.05473
61.895	0.05467
61.896	0.05491
61.896	0.05533
61.896	0.05529
61.896	0.05479
61.897	0.05437
61.897	0.05432
61.897	0.05426
61.898	0.05421
61.898	0.05416
61.898	0.05412

61.898	0.05408
61.899	0.05403
61.899	0.05476
61.899	0.05468
61.899	0.05407
61.900	0.05386
61.900	0.05382
61.900	0.05442
61.901	0.05461
61.901	0.0548
61.901	0.05387
61.901	0.05416
61.902	0.05397
61.902	0.05401
61.902	0.05405
61.903	0.05409
61.903	0.05413
61.903	0.05417
61.903	0.05421
61.904	0.05428
61.904	0.05435
61.904	0.05441
61.904	0.05447
61.905	0.05453
61.905	0.05458
61.905	0.05463
61.906	0.05467
61.906	0.05471
61.906	0.05475
61.906	0.05478
61.907	0.05481
61.907	0.05483
61.907	0.05485
61.907	0.05487
61.908	0.05488
61.908	0.05489
61.908	0.05489
61.908	0.05489
61.909	0.05488
61.909	0.05487
61.909	0.05486
61.910	0.05485
61.910	0.05484
61.910	0.05482
61.910	0.0548
61.911	0.05477
61.911	0.05474
61.911	0.05471
61.911	0.05467
61.912	0.05463
61.912	0.05458
61.912	0.05453
61.913	0.05448

61.913	0.05442
61.913	0.05436
61.913	0.05429
61.914	0.05422
61.914	0.05415
61.914	0.05407
61.914	0.05399
61.915	0.05391
61.915	0.05383
61.915	0.05389
61.915	0.05364
61.916	0.05355
61.916	0.05345
61.916	0.05336
61.917	0.05329
61.917	0.05366
61.917	0.05305
61.917	0.05294
61.918	0.05283
61.918	0.05272
61.918	0.05262
61.918	0.05251
61.919	0.0524
61.919	0.05238
61.919	0.05219
61.919	0.05208
61.920	0.05197
61.920	0.05186
61.920	0.05175
61.920	0.05167
61.921	0.05158
61.921	0.0515
61.921	0.05143
61.922	0.05194
61.922	0.05206
61.922	0.05127
61.922	0.05219
61.923	0.0521
61.923	0.05257
61.923	0.05156
61.923	0.05098
61.924	0.05091
61.924	0.05172
61.924	0.05202
61.924	0.05211
61.925	0.05224
61.925	0.05108
61.925	0.0505
61.925	0.05052
61.926	0.05197
61.926	0.05205
61.926	0.05144
61.927	0.05207

61.927	0.05224
61.927	0.05087
61.927	0.05077
61.928	0.05177
61.928	0.0518
61.928	0.05087
61.928	0.0509
61.929	0.0513
61.929	0.05144
61.929	0.05169
61.929	0.05213
61.930	0.05157
61.930	0.05111
61.930	0.05209
61.930	0.0511
61.931	0.05112
61.931	0.05107
61.931	0.05108
61.932	0.05108
61.932	0.05107
61.932	0.05106
61.932	0.05114
61.933	0.05174
61.933	0.05185
61.933	0.051
61.933	0.05098
61.934	0.05111
61.934	0.05122
61.934	0.05088
61.934	0.05084
61.935	0.0508
61.935	0.05077
61.935	0.05092
61.935	0.05065
61.936	0.0506
61.936	0.05054
61.936	0.05071
61.936	0.05083
61.937	0.05034
61.937	0.05027
61.937	0.0502
61.938	0.05012
61.938	0.05004
61.938	0.04996
61.938	0.04987
61.939	0.04983
61.939	0.04998
61.939	0.05048
61.939	0.05044
61.940	0.05081
61.940	0.05072
61.940	0.04989
61.940	0.0491

61.941	0.04899
61.941	0.04889
61.941	0.04928
61.941	0.04939
61.942	0.04912
61.942	0.04929
61.942	0.04833
61.942	0.04893
61.943	0.04907
61.943	0.04856
61.943	0.04943
61.944	0.04961
61.944	0.04852
61.944	0.04886
61.944	0.04853
61.945	0.04879
61.945	0.0491
61.945	0.0484
61.945	0.04881
61.946	0.04784
61.946	0.04812
61.946	0.04846
61.946	0.04775
61.947	0.04804
61.947	0.04796
61.947	0.04777
61.947	0.04834
61.948	0.04823
61.948	0.04741
61.948	0.04764
61.948	0.04733
61.949	0.04719
61.949	0.04732
61.949	0.04741
61.949	0.04771
61.950	0.04792
61.950	0.04697
61.950	0.04698
61.950	0.047
61.951	0.04737
61.951	0.04752
61.951	0.04703
61.952	0.04731
61.952	0.04705
61.952	0.04706
61.952	0.04707
61.953	0.04731
61.953	0.04709
61.953	0.0471
61.953	0.04711
61.954	0.04712
61.954	0.04713
61.954	0.04714

61.954	0.04715
61.955	0.04716
61.955	0.04717
61.955	0.04718
61.955	0.04719
61.956	0.0472
61.956	0.04721
61.956	0.04722
61.956	0.04723
61.957	0.04724
61.957	0.04725
61.957	0.04725
61.957	0.04726
61.958	0.04727
61.958	0.04728
61.958	0.04729
61.958	0.04729
61.959	0.0473
61.959	0.04731
61.959	0.04732
61.959	0.04732
61.960	0.04733
61.960	0.04734
61.960	0.04735
61.960	0.04735
61.961	0.04736
61.961	0.04737
61.961	0.04737
61.961	0.04738
61.962	0.04739
61.962	0.04739
61.962	0.0474
61.962	0.04741
61.963	0.04741
61.963	0.04742
61.963	0.04743
61.963	0.04743
61.964	0.04744
61.964	0.04744
61.964	0.04745
61.964	0.04745
61.965	0.04745
61.965	0.04746
61.965	0.04746
61.965	0.04746
61.966	0.04746
61.966	0.04747
61.966	0.04747
61.966	0.04747
61.967	0.04747
61.967	0.04747
61.967	0.04748
61.967	0.04748

61.968	0.04748
61.968	0.04748
61.968	0.04748
61.968	0.04748
61.969	0.04748
61.969	0.04748
61.969	0.04748
61.969	0.04748
61.970	0.04748
61.970	0.04747
61.970	0.04747
61.970	0.04747
61.971	0.04746
61.971	0.04746
61.971	0.04746
61.971	0.04745
61.972	0.04745
61.972	0.04744
61.972	0.04744
61.972	0.04743
61.973	0.04742
61.973	0.04742
61.973	0.04741
61.973	0.0474
61.974	0.0474
61.974	0.04739
61.974	0.04738
61.974	0.04737
61.975	0.04736
61.975	0.04735
61.975	0.04733
61.975	0.04732
61.976	0.04731
61.976	0.04729
61.976	0.04728
61.976	0.04727
61.977	0.04725
61.977	0.04724
61.977	0.04722
61.977	0.04721
61.978	0.04719
61.978	0.04718
61.978	0.04716
61.978	0.04714
61.979	0.04712
61.979	0.04711
61.979	0.04709
61.979	0.04707
61.980	0.04705
61.980	0.04703
61.980	0.04701
61.980	0.04699
61.981	0.04697

61.981	0.04695
61.981	0.04693
61.981	0.04691
61.982	0.04688
61.982	0.04686
61.982	0.04684
61.982	0.04681
61.983	0.04679
61.983	0.04677
61.983	0.04674
61.983	0.04672
61.984	0.0467
61.984	0.04667
61.984	0.04664
61.984	0.04662
61.985	0.04659
61.985	0.04657
61.985	0.04654
61.985	0.04651
61.986	0.04649
61.986	0.04646
61.986	0.04643
61.986	0.0464
61.987	0.04638
61.987	0.04635
61.987	0.04632
61.987	0.04629
61.988	0.04626
61.988	0.04624
61.988	0.04621
61.988	0.04618
61.989	0.04615
61.989	0.04611
61.989	0.04608
61.989	0.04605
61.990	0.04602
61.990	0.04599
61.990	0.04595
61.990	0.04592
61.991	0.04589
61.991	0.04586
61.991	0.04582
61.991	0.04579
61.992	0.04576
61.992	0.04572
61.992	0.04569
61.992	0.04566
61.993	0.04562
61.993	0.04559
61.993	0.04555
61.993	0.04552
61.994	0.04548
61.994	0.04544

61.994	0.04541
61.995	0.04537
61.995	0.04534
61.995	0.0453
61.995	0.04526
61.996	0.04523
61.996	0.04519
61.996	0.04515
61.996	0.04512
61.997	0.04508
61.997	0.04504
61.997	0.045
61.997	0.04497
61.998	0.04493
61.998	0.04489
61.998	0.04485
61.998	0.04481
61.999	0.04478
61.999	0.04474
61.999	0.0447
61.999	0.04466
62.000	0.04462
62.000	0.04458
62.000	0.04454
62.000	0.0445
62.001	0.04447
62.001	0.04443
62.001	0.04439
62.001	0.04435
62.002	0.04431
62.002	0.04427
62.002	0.04423
62.002	0.04419
62.003	0.04415
62.003	0.04411
62.003	0.04407
62.003	0.04402
62.004	0.04398
62.004	0.04394
62.004	0.0439
62.005	0.04386
62.005	0.04382
62.005	0.04377
62.005	0.04373
62.006	0.04369
62.006	0.04365
62.006	0.0436
62.006	0.04356
62.007	0.04352
62.007	0.04348
62.007	0.04343
62.007	0.04339
62.008	0.04335

62.008	0.0433
62.008	0.04326
62.008	0.04322
62.009	0.04317
62.009	0.04313
62.009	0.04309
62.009	0.04304
62.010	0.043
62.010	0.04296
62.010	0.04291
62.010	0.04287
62.011	0.04283
62.011	0.04278
62.011	0.04274
62.012	0.0427
62.012	0.04265
62.012	0.04261
62.012	0.04257
62.013	0.04252
62.013	0.04248
62.013	0.04243
62.013	0.04239
62.014	0.04235
62.014	0.0423
62.014	0.04226
62.014	0.04222
62.015	0.04218
62.015	0.04213
62.015	0.04209
62.015	0.04205
62.016	0.04201
62.016	0.04196
62.016	0.04192
62.016	0.04188
62.017	0.04184
62.017	0.0418
62.017	0.04176
62.018	0.04172
62.018	0.04168
62.018	0.04163
62.018	0.04159
62.019	0.04155
62.019	0.04151
62.019	0.04147
62.019	0.04143
62.020	0.04139
62.020	0.04135
62.020	0.04131
62.020	0.04127
62.021	0.04123
62.021	0.04119
62.021	0.04116
62.021	0.04112

62.022	0.04108
62.022	0.04104
62.022	0.041
62.022	0.04097
62.023	0.04093
62.023	0.04089
62.023	0.04085
62.024	0.04082
62.024	0.04078
62.024	0.04075
62.024	0.04071
62.025	0.04068
62.025	0.04064
62.025	0.04061
62.025	0.04058
62.026	0.04054
62.026	0.04051
62.026	0.04048
62.026	0.04044
62.027	0.04041
62.027	0.04038
62.027	0.04035
62.027	0.04031
62.028	0.04028
62.028	0.04025
62.028	0.04022
62.029	0.04019
62.029	0.04016
62.029	0.04013
62.029	0.0401
62.030	0.04007
62.030	0.04005
62.030	0.04002
62.030	0.03999
62.031	0.03997
62.031	0.03996
62.031	0.03994
62.031	0.03993
62.032	0.03992
62.032	0.0399
62.032	0.03989
62.032	0.03987
62.033	0.03985
62.033	0.03984
62.033	0.03982
62.034	0.0398
62.034	0.03978
62.034	0.03976
62.034	0.03974
62.035	0.03972
62.035	0.0397
62.035	0.03968
62.035	0.03965

62.036	0.03963
62.036	0.03961
62.036	0.03958
62.036	0.03956
62.037	0.03953
62.037	0.0395
62.037	0.03948
62.037	0.03945
62.038	0.03942
62.038	0.03939
62.038	0.03936
62.039	0.03933
62.039	0.0393
62.039	0.03927
62.039	0.03924
62.040	0.03921
62.040	0.03918
62.040	0.03915
62.040	0.03913
62.041	0.03911
62.041	0.03909
62.041	0.03907
62.041	0.03905
62.042	0.03904
62.042	0.03902
62.042	0.039
62.043	0.03898
62.043	0.03896
62.043	0.03894
62.043	0.03892
62.044	0.03891
62.044	0.03889
62.044	0.03888
62.044	0.03886
62.045	0.03885
62.045	0.03883
62.045	0.03882
62.045	0.03881
62.046	0.03879
62.046	0.03878
62.046	0.03876
62.047	0.03875
62.047	0.03874
62.047	0.03872
62.047	0.03871
62.048	0.03869
62.048	0.03868
62.048	0.03867
62.048	0.03865
62.049	0.03864
62.049	0.03863
62.049	0.03861
62.049	0.0386

62.050	0.03858
62.050	0.03857
62.050	0.03855
62.051	0.03854
62.051	0.03853
62.051	0.03851
62.051	0.0385
62.052	0.03848
62.052	0.03847
62.052	0.03845
62.052	0.03844
62.053	0.03842
62.053	0.03841
62.053	0.0384
62.053	0.03838
62.054	0.03837
62.054	0.03835
62.054	0.03834
62.055	0.03833
62.055	0.03831
62.055	0.0383
62.055	0.03829
62.056	0.03827
62.056	0.03826
62.056	0.03825
62.056	0.03823
62.057	0.03822
62.057	0.0382
62.057	0.03819
62.057	0.03818
62.058	0.03816
62.058	0.03815
62.058	0.03814
62.059	0.03812
62.059	0.03811
62.059	0.03809
62.059	0.03808
62.060	0.03807
62.060	0.03805
62.060	0.03804
62.060	0.03802
62.061	0.03801
62.061	0.03799
62.061	0.03798
62.062	0.03796
62.062	0.03795
62.062	0.03794
62.062	0.03792
62.063	0.03791
62.063	0.03789
62.063	0.03788
62.063	0.03786
62.064	0.03785

62.064	0.03783
62.064	0.03782
62.064	0.0378
62.065	0.03779
62.065	0.03777
62.065	0.03776
62.066	0.03774
62.066	0.03773
62.066	0.03771
62.066	0.0377
62.067	0.03768
62.067	0.03766
62.067	0.03765
62.067	0.03763
62.068	0.03762
62.068	0.0376
62.068	0.03758
62.069	0.03757
62.069	0.03755
62.069	0.03753
62.069	0.03752
62.070	0.0375
62.070	0.03748
62.070	0.03747
62.070	0.03745
62.071	0.03743
62.071	0.03741
62.071	0.0374
62.072	0.03738
62.072	0.03736
62.072	0.03734
62.072	0.03732
62.073	0.03731
62.073	0.03729
62.073	0.03727
62.073	0.03725
62.074	0.03723
62.074	0.03721
62.074	0.03719
62.075	0.03717
62.075	0.03715
62.075	0.03713
62.075	0.03711
62.076	0.03709
62.076	0.03707
62.076	0.03705
62.077	0.03703
62.077	0.037
62.077	0.03698
62.077	0.03696
62.078	0.03694
62.078	0.03692
62.078	0.03689

62.078	0.03687
62.079	0.03685
62.079	0.03682
62.079	0.0368
62.080	0.03677
62.080	0.03675
62.080	0.03672
62.080	0.0367
62.081	0.03667
62.081	0.03665
62.081	0.03662
62.082	0.0366
62.082	0.03657
62.082	0.03655
62.082	0.03652
62.083	0.03649
62.083	0.03646
62.083	0.03644
62.084	0.03641
62.084	0.03638
62.084	0.03635
62.084	0.03633
62.085	0.0363
62.085	0.03627
62.085	0.03624
62.085	0.03621
62.086	0.03618
62.086	0.03615
62.086	0.03612
62.087	0.03609
62.087	0.03606
62.087	0.03603
62.087	0.036
62.088	0.03597
62.088	0.03594
62.088	0.03591
62.089	0.03589
62.089	0.03586
62.089	0.03583
62.089	0.03579
62.090	0.03576
62.090	0.03573
62.090	0.0357
62.091	0.03567
62.091	0.03564
62.091	0.03561
62.091	0.03557
62.092	0.03554
62.092	0.03551
62.092	0.03548
62.093	0.03544
62.093	0.03541
62.093	0.0354

62.094	0.03539
62.094	0.03538
62.094	0.03536
62.094	0.03535
62.095	0.03534
62.095	0.03532
62.095	0.03531
62.096	0.0353
62.096	0.03528
62.096	0.03526
62.096	0.03525
62.097	0.03523
62.097	0.03521
62.097	0.0352
62.098	0.03518
62.098	0.03544
62.098	0.03543
62.098	0.03512
62.099	0.0351
62.099	0.03508
62.099	0.03506
62.100	0.03504
62.100	0.03571
62.100	0.03575
62.101	0.03497
62.101	0.03498
62.101	0.03498
62.101	0.03489
62.102	0.03558
62.102	0.03609
62.102	0.03613
62.103	0.03487
62.103	0.03477
62.103	0.03507
62.104	0.03509
62.104	0.03497
62.104	0.03651
62.104	0.03655
62.105	0.03528
62.105	0.03528
62.105	0.0354
62.106	0.03483
62.106	0.03664
62.106	0.03677
62.106	0.03558
62.107	0.03545
62.107	0.03542
62.107	0.03531
62.108	0.03699
62.108	0.0371
62.108	0.03589
62.109	0.03544
62.109	0.03538

62.109	0.03567
62.109	0.03746
62.110	0.03742
62.110	0.03664
62.110	0.0362
62.111	0.03713
62.111	0.03736
62.111	0.03811
62.112	0.03634
62.112	0.03749
62.112	0.03802
62.112	0.03766
62.113	0.03844
62.113	0.03767
62.113	0.03695
62.114	0.03782
62.114	0.03777
62.114	0.03725
62.115	0.03734
62.115	0.03795
62.115	0.03794
62.115	0.03762
62.116	0.03771
62.116	0.03815
62.116	0.03813
62.117	0.03796
62.117	0.03865
62.117	0.03839
62.118	0.03833
62.118	0.03855
62.118	0.03836
62.118	0.03843
62.119	0.03864
62.119	0.03857
62.119	0.0395
62.120	0.03871
62.120	0.03938
62.120	0.03883
62.121	0.03889
62.121	0.03895
62.121	0.03901
62.121	0.03906
62.122	0.03911
62.122	0.03916
62.122	0.0392
62.123	0.03925
62.123	0.03929
62.123	0.03933
62.124	0.03937
62.124	0.0394
62.124	0.03943
62.124	0.03946
62.125	0.03949

62.125	0.03952
62.125	0.03954
62.126	0.03956
62.126	0.03958
62.126	0.0396
62.127	0.03962
62.127	0.03963
62.127	0.03964
62.127	0.03966
62.128	0.03966
62.128	0.03967
62.128	0.03968
62.129	0.03968
62.129	0.03968
62.129	0.03968
62.130	0.03968
62.130	0.03967
62.130	0.03967
62.130	0.03966
62.131	0.03965
62.131	0.03964
62.131	0.03963
62.132	0.03962
62.132	0.0396
62.132	0.03958
62.133	0.03957
62.133	0.03955
62.133	0.03953
62.134	0.03951
62.134	0.03948
62.134	0.03946
62.134	0.03943
62.135	0.03941
62.135	0.03938
62.135	0.03935
62.136	0.03979
62.136	0.03981
62.136	0.03927
62.137	0.03924
62.137	0.03921
62.137	0.03917
62.138	0.03914
62.138	0.03911
62.138	0.03908
62.138	0.03912
62.139	0.03901
62.139	0.03897
62.139	0.03948
62.140	0.03894
62.140	0.03898
62.140	0.03887
62.141	0.03879
62.141	0.03876

62.141	0.03872
62.142	0.03883
62.142	0.03865
62.142	0.03862
62.143	0.0386
62.143	0.03976
62.143	0.03974
62.143	0.03875
62.144	0.03853
62.144	0.03851
62.144	0.0385
62.145	0.03848
62.145	0.03846
62.145	0.03845
62.146	0.03843
62.146	0.03946
62.146	0.0394
62.147	0.03846
62.147	0.03836
62.147	0.03834
62.148	0.03832
62.148	0.03867
62.148	0.03827
62.148	0.03825
62.149	0.03929
62.149	0.03923
62.149	0.03818
62.150	0.03946
62.150	0.03924
62.150	0.03878
62.151	0.03928
62.151	0.03894
62.151	0.03802
62.152	0.03886
62.152	0.03883
62.152	0.03841
62.153	0.03803
62.153	0.03851
62.153	0.03826
62.154	0.03802
62.154	0.03789
62.154	0.0379
62.155	0.03793
62.155	0.0387
62.155	0.03864
62.156	0.03806
62.156	0.03805
62.156	0.03809
62.157	0.03812
62.157	0.03815
62.157	0.03818
62.157	0.03821
62.158	0.03824

62.158	0.03844
62.158	0.03836
62.159	0.03833
62.159	0.03836
62.159	0.03838
62.160	0.03841
62.160	0.03843
62.160	0.03846
62.161	0.03848
62.161	0.0385
62.161	0.03853
62.162	0.03855
62.162	0.03857
62.162	0.03858
62.163	0.0386
62.163	0.03862
62.163	0.03863
62.164	0.03865
62.164	0.03866
62.164	0.03867
62.165	0.03868
62.165	0.03868
62.165	0.03869
62.166	0.03869
62.166	0.0387
62.166	0.0387
62.167	0.0387
62.167	0.0387
62.167	0.03869
62.168	0.03869
62.168	0.03868
62.168	0.03867
62.169	0.03866
62.169	0.03864
62.169	0.03863
62.170	0.03861
62.170	0.03859
62.170	0.03857
62.171	0.03855
62.171	0.03852
62.171	0.03849
62.172	0.03846
62.172	0.03843
62.172	0.0384
62.173	0.03836
62.173	0.03832
62.173	0.03828
62.174	0.03824
62.174	0.03819
62.174	0.03815
62.175	0.0381
62.175	0.03805
62.175	0.0383

62.176	0.03794
62.176	0.03788
62.176	0.03783
62.177	0.03776
62.177	0.0377
62.177	0.03764
62.178	0.03757
62.178	0.0375
62.178	0.03744
62.179	0.03736
62.179	0.03729
62.179	0.0376
62.180	0.03739
62.180	0.03706
62.180	0.03699
62.181	0.0369
62.181	0.03696
62.181	0.03679
62.182	0.03666
62.182	0.03691
62.182	0.03668
62.183	0.0364
62.183	0.03631
62.183	0.03691
62.184	0.03662
62.184	0.03641
62.184	0.03663
62.185	0.03617
62.185	0.0366
62.185	0.03657
62.186	0.036
62.186	0.03582
62.187	0.03539
62.187	0.03529
62.187	0.0352
62.188	0.0351
62.188	0.03501
62.188	0.03496
62.189	0.03481
62.189	0.03525
62.189	0.03497
62.190	0.03543
62.190	0.03522
62.190	0.03466
62.191	0.03535
62.191	0.03542
62.191	0.03468
62.192	0.03425
62.192	0.03499
62.192	0.03472
62.193	0.03502
62.193	0.03479
62.193	0.03413

62.194	0.03393
62.194	0.03433
62.194	0.03495
62.195	0.03459
62.195	0.03428
62.196	0.03428
62.196	0.03411
62.196	0.03439
62.197	0.03401
62.197	0.03397
62.197	0.03408
62.198	0.03451
62.198	0.03429
62.198	0.03396
62.199	0.03371
62.199	0.0335
62.199	0.03372
62.200	0.03409
62.200	0.03403
62.200	0.03402
62.201	0.0337
62.201	0.03402
62.201	0.03389
62.202	0.03418
62.202	0.03412
62.202	0.0343
62.203	0.0341
62.203	0.034
62.204	0.0338
62.204	0.03404
62.204	0.03441
62.205	0.03428
62.205	0.03327
62.205	0.0341
62.206	0.03373
62.206	0.0342
62.206	0.03436
62.207	0.03431
62.207	0.03375
62.207	0.03338
62.208	0.03384
62.208	0.03347
62.208	0.03452
62.209	0.03445
62.209	0.03344
62.209	0.03329
62.210	0.03299
62.210	0.03371
62.210	0.0346
62.211	0.03455
62.211	0.03418
62.212	0.03387
62.212	0.03427

62.212	0.03454
62.213	0.0345
62.213	0.03345
62.213	0.03353
62.214	0.03332
62.214	0.03399
62.214	0.03478
62.215	0.03469
62.215	0.03352
62.215	0.03326
62.216	0.0338
62.216	0.03456
62.216	0.03453
62.217	0.03443
62.217	0.03389
62.217	0.03397
62.218	0.03459
62.218	0.03453
62.218	0.03387
62.219	0.03427
62.219	0.03379
62.220	0.03453
62.220	0.03447
62.220	0.03364
62.221	0.0332
62.221	0.03401
62.221	0.03441
62.222	0.03434
62.222	0.03327
62.222	0.03335
62.223	0.03321
62.223	0.03424
62.223	0.03416
62.224	0.03354
62.224	0.03333
62.224	0.0325
62.225	0.03403
62.225	0.03394
62.225	0.03283
62.226	0.03249
62.226	0.03233
62.226	0.03377
62.227	0.03367
62.227	0.03256
62.228	0.03405
62.228	0.0335
62.228	0.03329
62.229	0.03255
62.229	0.03196
62.229	0.03344
62.230	0.03304
62.230	0.03201
62.230	0.03166

62.231	0.03292
62.231	0.03308
62.231	0.03266
62.232	0.03225
62.232	0.03264
62.232	0.03245
62.233	0.03265
62.233	0.03228
62.233	0.03232
62.234	0.03213
62.234	0.03155
62.234	0.03229
62.235	0.03197
62.235	0.03178
62.236	0.03162
62.236	0.03128
62.236	0.03185
62.237	0.03145
62.237	0.03084
62.237	0.03079
62.238	0.0313
62.238	0.03142
62.238	0.03102
62.239	0.03105
62.239	0.03096
62.239	0.03077
62.240	0.03098
62.240	0.03059
62.240	0.0306
62.241	0.03041
62.241	0.03051
62.241	0.03067
62.242	0.03031
62.242	0.03021
62.242	0.03017
62.243	0.02977
62.243	0.02961
62.244	0.03004
62.244	0.02983
62.244	0.0298
62.245	0.02938
62.245	0.02931
62.245	0.02911
62.246	0.02943
62.246	0.02934
62.246	0.02989
62.247	0.02954
62.247	0.02892
62.247	0.02889
62.248	0.02913
62.248	0.02903
62.248	0.02877
62.249	0.029

62.249	0.0287
62.249	0.02884
62.250	0.02873
62.250	0.02868
62.250	0.02917
62.251	0.02884
62.251	0.02863
62.252	0.02872
62.252	0.02917
62.252	0.02884
62.253	0.02849
62.253	0.02838
62.253	0.02897
62.254	0.02865
62.254	0.02832
62.254	0.02832
62.255	0.02828
62.255	0.02878
62.255	0.02855
62.256	0.0286
62.256	0.02862
62.256	0.0284
62.257	0.02828
62.257	0.02829
62.257	0.0283
62.258	0.0283
62.258	0.02831
62.258	0.02838
62.259	0.02832
62.259	0.02832
62.259	0.02833
62.260	0.02833
62.260	0.02833
62.261	0.02834
62.261	0.02834
62.261	0.02834
62.262	0.02834
62.262	0.02835
62.262	0.02835
62.263	0.02835
62.263	0.02835
62.263	0.02834
62.264	0.02834
62.264	0.02834
62.264	0.02834
62.265	0.02833
62.265	0.02833
62.265	0.02833
62.266	0.02832
62.266	0.02831
62.266	0.0283
62.267	0.02871
62.267	0.02829

62.267	0.02863
62.268	0.02826
62.268	0.02825
62.268	0.02824
62.269	0.02822
62.269	0.02821
62.270	0.02819
62.270	0.02817
62.270	0.02815
62.271	0.02813
62.271	0.02811
62.271	0.02809
62.272	0.02807
62.272	0.02804
62.272	0.02801
62.273	0.02824
62.273	0.02811
62.273	0.02802
62.274	0.0279
62.274	0.02787
62.274	0.02783
62.275	0.0278
62.275	0.02778
62.275	0.02775
62.276	0.02773
62.276	0.0277
62.276	0.02767
62.277	0.02764
62.277	0.02761
62.277	0.02758
62.278	0.02755
62.278	0.02751
62.279	0.02748
62.279	0.02744
62.279	0.0274
62.280	0.02736
62.280	0.02732
62.280	0.02737
62.281	0.02725
62.281	0.02718
62.281	0.02713
62.282	0.02708
62.282	0.02727
62.282	0.02703
62.283	0.02693
62.283	0.02687
62.283	0.02709
62.284	0.02694
62.284	0.0267
62.284	0.02728
62.285	0.02659
62.285	0.02713
62.285	0.02668

62.286	0.02666
62.286	0.02658
62.286	0.02628
62.287	0.02622
62.287	0.02615
62.288	0.02613
62.288	0.02607
62.288	0.02606
62.289	0.02606
62.289	0.02605
62.289	0.02604
62.290	0.02603
62.290	0.02603
62.290	0.02602
62.291	0.02601
62.291	0.026
62.291	0.02599
62.292	0.02598
62.292	0.02597
62.292	0.02596
62.293	0.02594
62.293	0.02593
62.293	0.02592
62.294	0.0259
62.294	0.02589
62.294	0.02587
62.295	0.02586
62.295	0.02584
62.295	0.02582
62.296	0.02581
62.296	0.02579
62.297	0.02577
62.297	0.02575
62.297	0.02574
62.298	0.02572
62.298	0.0257
62.298	0.02568
62.299	0.02566
62.299	0.02564
62.299	0.02562
62.300	0.02559
62.300	0.02557
62.300	0.02555
62.301	0.02552
62.301	0.0255
62.301	0.02547
62.302	0.02545
62.302	0.02543
62.302	0.02541
62.303	0.02538
62.303	0.02536
62.303	0.02534
62.304	0.02531

62.304	0.02529
62.304	0.02526
62.305	0.02524
62.305	0.02521
62.306	0.02519
62.306	0.02516
62.306	0.02513
62.307	0.0251
62.307	0.02508
62.307	0.02505
62.308	0.02502
62.308	0.02499
62.308	0.02498
62.309	0.02496
62.309	0.02494
62.309	0.02493
62.310	0.02491
62.310	0.02489
62.310	0.02488
62.311	0.02486
62.311	0.02484
62.311	0.02483
62.312	0.02481
62.312	0.02479
62.312	0.02477
62.313	0.02476
62.313	0.02474
62.314	0.02472
62.314	0.0247
62.314	0.02469
62.315	0.02467
62.315	0.02465
62.315	0.02463
62.316	0.02461
62.316	0.02459
62.316	0.02457
62.317	0.02455
62.317	0.02453
62.317	0.02452
62.318	0.0245
62.318	0.02448
62.318	0.02446
62.319	0.02444
62.319	0.02442
62.319	0.0244
62.320	0.02438
62.320	0.02436
62.320	0.02434
62.321	0.02432
62.321	0.0243
62.322	0.02429
62.322	0.02427
62.322	0.02425

62.323	0.02424
62.323	0.02422
62.323	0.0242
62.324	0.02418
62.324	0.02416
62.324	0.02414
62.325	0.02412
62.325	0.0241
62.325	0.02408
62.326	0.02406
62.326	0.02404
62.326	0.02402
62.327	0.024
62.327	0.02398
62.327	0.02396
62.328	0.02395
62.328	0.02393
62.328	0.02391
62.329	0.02389
62.329	0.02387
62.329	0.02385
62.330	0.02383
62.330	0.02381
62.331	0.02379
62.331	0.02377
62.331	0.02375
62.332	0.02373
62.332	0.02372
62.332	0.0237
62.333	0.02368
62.333	0.02366
62.333	0.02364
62.334	0.02362
62.334	0.0236
62.334	0.02357
62.335	0.02355
62.335	0.02354
62.335	0.02352
62.336	0.0235
62.336	0.02349
62.336	0.02347
62.337	0.02345
62.337	0.02344
62.337	0.02342
62.338	0.0234
62.338	0.02337
62.338	0.02335
62.339	0.02333
62.339	0.02332
62.339	0.02332
62.340	0.02332
62.340	0.02332
62.340	0.02332

62.341	0.02332
62.341	0.02332
62.341	0.02331
62.342	0.02331
62.342	0.02331
62.342	0.02331
62.343	0.02331
62.343	0.0233
62.343	0.0233
62.344	0.0233
62.344	0.0233
62.344	0.02329
62.345	0.02329
62.345	0.02328
62.345	0.02328
62.346	0.02327
62.346	0.02327
62.346	0.02326
62.347	0.02325
62.347	0.02325
62.347	0.02324
62.348	0.02324
62.348	0.02323
62.348	0.02322
62.349	0.02322
62.349	0.02321
62.349	0.0232
62.350	0.02319
62.350	0.02318
62.350	0.02317
62.351	0.02317
62.351	0.02316
62.351	0.02315
62.352	0.02313
62.352	0.02312
62.352	0.02311
62.353	0.0231
62.353	0.02309
62.353	0.02308
62.354	0.02306
62.354	0.02305
62.354	0.02304
62.355	0.02302
62.355	0.02301
62.355	0.02299
62.356	0.02298
62.356	0.02296
62.357	0.02294
62.357	0.02293
62.357	0.02291
62.358	0.02289
62.358	0.02287
62.358	0.02286

62.359	0.02284
62.359	0.02282
62.359	0.0228
62.360	0.02278
62.360	0.02276
62.360	0.02273
62.361	0.02271
62.361	0.02269
62.361	0.02267
62.362	0.02264
62.362	0.02262
62.362	0.0226
62.363	0.02258
62.363	0.02256
62.363	0.02254
62.364	0.02251
62.364	0.02249
62.364	0.02247
62.365	0.02244
62.365	0.02242
62.365	0.02239
62.366	0.02237
62.366	0.02234
62.366	0.02232
62.367	0.02229
62.367	0.02227
62.367	0.02224
62.368	0.02221
62.368	0.02219
62.368	0.02216
62.369	0.02213
62.369	0.0221
62.370	0.02208
62.370	0.02205
62.370	0.02202
62.371	0.02199
62.371	0.02196
62.371	0.02193
62.372	0.0219
62.372	0.02187
62.372	0.02184
62.373	0.02181
62.373	0.02179
62.373	0.02179
62.374	0.02178
62.374	0.02177
62.374	0.02177
62.375	0.02176
62.375	0.02175
62.375	0.02174
62.376	0.02174
62.376	0.02173
62.376	0.02172

62.377	0.02171
62.377	0.0217
62.377	0.02169
62.378	0.02168
62.378	0.02167
62.379	0.02166
62.379	0.02166
62.379	0.02167
62.380	0.02168
62.380	0.02168
62.380	0.02169
62.381	0.0217
62.381	0.0217
62.381	0.02171
62.382	0.02172
62.382	0.02172
62.382	0.02172
62.383	0.02173
62.383	0.02173
62.383	0.02175
62.384	0.02176
62.384	0.02177
62.385	0.02178
62.385	0.02179
62.385	0.0218
62.386	0.02181
62.386	0.02181
62.386	0.02181
62.387	0.02182
62.387	0.02182
62.387	0.02182
62.388	0.02182
62.388	0.02182
62.388	0.02181
62.389	0.02181
62.389	0.02181
62.390	0.0218
62.390	0.02179
62.390	0.02178
62.391	0.02177
62.391	0.02176
62.391	0.02175
62.392	0.02174
62.392	0.02173
62.392	0.02171
62.393	0.0217
62.393	0.02168
62.394	0.02166
62.394	0.02165
62.394	0.02163
62.395	0.02161
62.395	0.02159
62.395	0.02157

62.396	0.02155
62.396	0.02153
62.396	0.0215
62.397	0.02148
62.397	0.02146
62.398	0.02143
62.398	0.02141
62.398	0.02139
62.399	0.02136
62.399	0.02134
62.399	0.02131
62.400	0.02128
62.400	0.02126
62.400	0.02123
62.401	0.0212
62.401	0.02118
62.402	0.02115
62.402	0.02112
62.402	0.0211
62.403	0.02107
62.403	0.02104
62.403	0.02102
62.404	0.02101
62.404	0.021
62.405	0.02098
62.405	0.02097
62.405	0.02096
62.406	0.02094
62.406	0.02092
62.407	0.02091
62.407	0.02089
62.407	0.02087
62.408	0.02085
62.408	0.02083
62.408	0.02081
62.409	0.02078
62.409	0.02076
62.410	0.02074
62.410	0.02071
62.410	0.02069
62.411	0.02066
62.411	0.02064
62.411	0.02061
62.412	0.02058
62.412	0.02055
62.413	0.02053
62.413	0.0205
62.413	0.02047
62.414	0.02045
62.414	0.02042
62.415	0.02039
62.415	0.02036
62.415	0.02035

62.416	0.02038
62.416	0.0204
62.417	0.02042
62.417	0.02044
62.417	0.02047
62.418	0.02049
62.418	0.02051
62.419	0.02053
62.419	0.02056
62.419	0.02058
62.420	0.0206
62.420	0.02061
62.420	0.02063
62.421	0.02065
62.421	0.02069
62.422	0.02073
62.422	0.02077
62.422	0.02081
62.423	0.02085
62.423	0.02088
62.424	0.02092
62.424	0.02095
62.424	0.02098
62.425	0.02101
62.425	0.02104
62.426	0.02107
62.426	0.02109
62.426	0.02111
62.427	0.02113
62.427	0.02115
62.428	0.02117
62.428	0.02118
62.428	0.02119
62.429	0.0212
62.429	0.02121
62.430	0.02122
62.430	0.02122
62.430	0.02122
62.431	0.02122
62.431	0.02122
62.432	0.02121
62.432	0.0212
62.432	0.02119
62.433	0.02118
62.433	0.02119
62.434	0.02122
62.434	0.02124
62.434	0.02127
62.435	0.02129
62.435	0.02132
62.436	0.02134
62.436	0.02136
62.436	0.02138

62.437	0.0214
62.437	0.02142
62.438	0.02144
62.438	0.02145
62.438	0.02147
62.439	0.02148
62.439	0.0215
62.440	0.02151
62.440	0.02152
62.440	0.02154
62.441	0.02155
62.441	0.02156
62.442	0.02157
62.442	0.02158
62.442	0.02159
62.443	0.0216
62.443	0.02161
62.444	0.02162
62.444	0.02172
62.444	0.02163
62.445	0.02164
62.445	0.02165
62.446	0.02166
62.446	0.0217
62.446	0.02183
62.447	0.02177
62.447	0.02181
62.448	0.02184
62.448	0.02188
62.448	0.02191
62.449	0.02194
62.449	0.02207
62.450	0.02201
62.450	0.02203
62.450	0.02206
62.451	0.02209
62.451	0.02211
62.452	0.02213
62.452	0.02215
62.452	0.02217
62.453	0.02219
62.453	0.02221
62.454	0.02222
62.454	0.02223
62.454	0.02224
62.455	0.02225
62.455	0.02226
62.456	0.02264
62.456	0.02239
62.456	0.02227
62.457	0.02228
62.457	0.02228
62.458	0.02228

62.458	0.02228
62.459	0.02227
62.459	0.0225
62.459	0.02228
62.460	0.02227
62.460	0.02228
62.461	0.0223
62.461	0.02233
62.461	0.02235
62.462	0.02237
62.462	0.02239
62.463	0.02244
62.463	0.02243
62.463	0.02245
62.464	0.02247
62.464	0.02249
62.465	0.0225
62.465	0.02252
62.465	0.02254
62.466	0.02255
62.466	0.02256
62.467	0.02258
62.467	0.02259
62.468	0.0226
62.468	0.02261
62.468	0.02262
62.469	0.02262
62.469	0.02263
62.470	0.02263
62.470	0.02264
62.470	0.02264
62.471	0.02264
62.471	0.02264
62.472	0.02264
62.472	0.02263
62.472	0.02263
62.473	0.02262
62.473	0.02261
62.474	0.0226
62.474	0.02258
62.475	0.02257
62.475	0.02255
62.475	0.02254
62.476	0.02252
62.476	0.02249
62.477	0.02247
62.477	0.02245
62.477	0.02242
62.478	0.02239
62.478	0.02236
62.479	0.02232
62.479	0.02229
62.479	0.02225

62.480	0.02221
62.480	0.02217
62.481	0.02213
62.481	0.02208
62.482	0.02204
62.482	0.02199
62.482	0.02194
62.483	0.02189
62.483	0.02183
62.484	0.02178
62.484	0.02172
62.484	0.02166
62.485	0.0216
62.485	0.02154
62.486	0.02148
62.486	0.02141
62.487	0.02135
62.487	0.02128
62.487	0.02121
62.488	0.02114
62.488	0.02107
62.489	0.021
62.489	0.02093
62.489	0.02086
62.490	0.02078
62.490	0.02071
62.491	0.0208
62.491	0.02064
62.492	0.02048
62.492	0.0204
62.492	0.02042
62.493	0.02024
62.493	0.02049
62.494	0.0202
62.494	0.0201
62.495	0.02036
62.495	0.02005
62.495	0.02005
62.496	0.02019
62.496	0.02013
62.497	0.02004
62.497	0.01997
62.498	0.02001
62.498	0.01985
62.498	0.01992
62.499	0.01991
62.499	0.01996
62.500	0.01963
62.500	0.01935
62.501	0.01982
62.501	0.01953
62.501	0.01976
62.502	0.01948

62.502	0.01927
62.503	0.01977
62.503	0.01974
62.504	0.01943
62.504	0.01925
62.504	0.01917
62.505	0.01915
62.505	0.01959
62.506	0.0196
62.506	0.01945
62.507	0.01941
62.507	0.0194
62.507	0.01921
62.508	0.01925
62.508	0.01938
62.509	0.01907
62.509	0.01887
62.510	0.01884
62.510	0.01926
62.510	0.01897
62.511	0.01913
62.511	0.01906
62.512	0.01922
62.512	0.0189
62.513	0.01859
62.513	0.01903
62.513	0.01909
62.514	0.01876
62.514	0.01875
62.515	0.01859
62.515	0.01859
62.516	0.01886
62.516	0.01858
62.517	0.01858
62.517	0.0188
62.517	0.01857
62.518	0.01857
62.518	0.01856
62.519	0.01856
62.519	0.01855
62.520	0.01854
62.520	0.01854
62.520	0.01853
62.521	0.01852
62.521	0.01851
62.522	0.01849
62.522	0.01848
62.523	0.01846
62.523	0.01845
62.524	0.01843
62.524	0.01841
62.524	0.01839
62.525	0.01837

62.525	0.01835
62.526	0.01832
62.526	0.0183
62.527	0.01827
62.527	0.01824
62.528	0.01821
62.528	0.01818
62.528	0.01814
62.529	0.01811
62.529	0.01807
62.530	0.01803
62.530	0.01799
62.531	0.01795
62.531	0.01792
62.532	0.01788
62.532	0.01785
62.532	0.01781
62.533	0.01777
62.533	0.01773
62.534	0.01768
62.534	0.01764
62.535	0.01759
62.535	0.01755
62.536	0.0175
62.536	0.01745
62.536	0.0174
62.537	0.01735
62.537	0.0173
62.538	0.01725
62.538	0.01719
62.539	0.01714
62.539	0.01709
62.540	0.01703
62.540	0.01697
62.540	0.01692
62.541	0.01686
62.541	0.01681
62.542	0.01675
62.542	0.01669
62.543	0.01663
62.543	0.01658
62.544	0.01653
62.544	0.01649
62.545	0.01644
62.545	0.01641
62.545	0.01639
62.546	0.01636
62.546	0.01634
62.547	0.01631
62.547	0.01629
62.548	0.01626
62.548	0.01623
62.549	0.01621

62.549	0.01618
62.550	0.01615
62.550	0.01612
62.550	0.0161
62.551	0.01607
62.551	0.01604
62.552	0.01601
62.552	0.01598
62.553	0.01595
62.553	0.01592
62.554	0.0159
62.554	0.01587
62.554	0.01585
62.555	0.01582
62.555	0.01579
62.556	0.01577
62.556	0.01574
62.557	0.01571
62.557	0.01568
62.558	0.01565
62.558	0.01563
62.559	0.0156
62.559	0.01558
62.559	0.01557
62.560	0.01556
62.560	0.01554
62.561	0.01553
62.561	0.01551
62.562	0.0155
62.562	0.01548
62.563	0.01546
62.563	0.01545
62.564	0.01543
62.564	0.01541
62.565	0.01539
62.565	0.01537
62.565	0.01535
62.566	0.01532
62.566	0.0153
62.567	0.01528
62.567	0.01525
62.568	0.01523
62.568	0.0152
62.569	0.01517
62.569	0.01515
62.570	0.01512
62.570	0.01509
62.570	0.01506
62.571	0.01503
62.571	0.015
62.572	0.01496
62.572	0.01493
62.573	0.0149

62.573	0.01486
62.574	0.01483
62.574	0.01479
62.575	0.01475
62.575	0.01472
62.575	0.01468
62.576	0.01464
62.576	0.0146
62.577	0.01458
62.577	0.01458
62.578	0.01458
62.578	0.01458
62.579	0.01458
62.579	0.01458
62.580	0.01458
62.580	0.01457
62.580	0.01456
62.581	0.01456
62.581	0.01455
62.582	0.01454
62.582	0.01453
62.583	0.01451
62.583	0.0145
62.584	0.01448
62.584	0.01446
62.585	0.01444
62.585	0.01442
62.585	0.0144
62.586	0.01437
62.586	0.01435
62.587	0.01432
62.587	0.01429
62.588	0.01426
62.588	0.01422
62.589	0.01419
62.589	0.01415
62.590	0.01411
62.590	0.01407
62.590	0.01403
62.591	0.01399
62.591	0.01395
62.592	0.0139
62.592	0.01385
62.593	0.0138
62.593	0.01375
62.594	0.0137
62.594	0.01365
62.594	0.0136
62.595	0.01354
62.595	0.01349
62.596	0.01343
62.596	0.01341
62.597	0.01341

62.597	0.01341
62.598	0.01341
62.598	0.01341
62.599	0.01341
62.599	0.01341
62.599	0.01341
62.600	0.0134
62.600	0.0134
62.601	0.0134
62.601	0.0134
62.602	0.0134
62.602	0.01339
62.603	0.01339
62.603	0.01339
62.603	0.01339
62.604	0.01338
62.604	0.01338
62.605	0.01337
62.605	0.01337
62.606	0.01337
62.606	0.01337
62.607	0.01338
62.607	0.01338
62.608	0.01338
62.608	0.01338
62.608	0.01338
62.609	0.01338
62.609	0.01338
62.610	0.01338
62.610	0.01338
62.611	0.01338
62.611	0.01337
62.612	0.01337
62.612	0.01337
62.612	0.01337
62.613	0.01336
62.613	0.01336
62.614	0.01336
62.614	0.01335
62.615	0.01335
62.615	0.01335
62.616	0.01334
62.616	0.01334
62.617	0.01333
62.617	0.01333
62.617	0.01332
62.618	0.01332
62.618	0.01331
62.619	0.0133
62.619	0.0133
62.620	0.01329
62.620	0.01328
62.621	0.01328

62.621	0.01327
62.621	0.01326
62.622	0.01325
62.622	0.01325
62.623	0.01324
62.623	0.01323
62.624	0.01322
62.624	0.01321
62.625	0.0132
62.625	0.01319
62.626	0.01318
62.626	0.01317
62.626	0.01316
62.627	0.01315
62.627	0.01314
62.628	0.01313
62.628	0.01312
62.629	0.01311
62.629	0.01309
62.630	0.01308
62.630	0.01307
62.631	0.01306
62.631	0.01304
62.631	0.01303
62.632	0.01302
62.632	0.013
62.633	0.01299
62.633	0.01298
62.634	0.01296
62.634	0.01295
62.635	0.01293
62.635	0.01292
62.636	0.0129
62.636	0.01289
62.637	0.01287
62.637	0.01286
62.637	0.01284
62.638	0.01283
62.638	0.01281
62.639	0.01279
62.639	0.01278
62.640	0.01276
62.640	0.01274
62.641	0.01273
62.641	0.01271
62.642	0.01269
62.642	0.01267
62.643	0.01266
62.643	0.01264
62.644	0.01262
62.644	0.0126
62.644	0.01258
62.645	0.01256

62.645	0.01254
62.646	0.01252
62.646	0.0125
62.647	0.01248
62.647	0.01246
62.648	0.01244
62.648	0.01242
62.649	0.0124
62.649	0.01238
62.650	0.01236
62.650	0.01234
62.651	0.01232
62.651	0.0123
62.652	0.01228
62.652	0.01226
62.653	0.01224
62.653	0.01222
62.653	0.01219
62.654	0.01217
62.654	0.01215
62.655	0.01213
62.655	0.0121
62.656	0.01208
62.656	0.01206
62.657	0.01204
62.657	0.01201
62.658	0.01199
62.658	0.01197
62.659	0.01195
62.659	0.01192
62.660	0.0119
62.660	0.01188
62.661	0.01185
62.661	0.01183
62.662	0.01181
62.662	0.01178
62.663	0.01176
62.663	0.01174
62.664	0.01171
62.664	0.01169
62.665	0.01166
62.665	0.01164
62.666	0.01162
62.666	0.01159
62.667	0.01157
62.667	0.01155
62.668	0.01152
62.668	0.0115
62.669	0.01148
62.669	0.01145
62.670	0.01143
62.670	0.01141
62.671	0.01138

62.671	0.01136
62.672	0.01134
62.672	0.01132
62.673	0.01129
62.673	0.01127
62.674	0.01125
62.674	0.01123
62.675	0.01121
62.675	0.01118
62.676	0.01116
62.676	0.01114
62.677	0.01112
62.677	0.0111
62.678	0.01107
62.678	0.01105
62.679	0.01103
62.679	0.01101
62.680	0.01099
62.680	0.01097
62.681	0.01095
62.681	0.01093
62.682	0.01091
62.682	0.0109
62.683	0.01089
62.683	0.01088
62.684	0.01087
62.684	0.01086
62.685	0.01085
62.685	0.01084
62.686	0.01083
62.686	0.01081
62.687	0.0108
62.688	0.01079
62.688	0.01077
62.689	0.01076
62.689	0.01075
62.690	0.01073
62.690	0.01072
62.691	0.0107
62.691	0.01068
62.692	0.01067
62.692	0.01065
62.693	0.01063
62.693	0.01062
62.694	0.01061
62.694	0.0106
62.695	0.01059
62.695	0.01058
62.696	0.01057
62.696	0.01055
62.697	0.01054
62.698	0.01053
62.698	0.01052

62.699	0.01051
62.699	0.01049
62.700	0.01048
62.700	0.01047
62.701	0.01045
62.701	0.01044
62.702	0.01042
62.702	0.01041
62.703	0.01039
62.703	0.01037
62.704	0.01036
62.704	0.01034
62.705	0.01033
62.706	0.01033
62.706	0.01032
62.707	0.01031
62.707	0.0103
62.708	0.0103
62.708	0.01029
62.709	0.01028
62.709	0.01027
62.710	0.01026
62.710	0.01025
62.711	0.01024
62.711	0.01023
62.712	0.01022
62.713	0.01021
62.713	0.01021
62.714	0.01021
62.714	0.01022
62.715	0.01022
62.715	0.01022
62.716	0.01022
62.716	0.01022
62.717	0.01022
62.717	0.01022
62.718	0.01022
62.718	0.01022
62.719	0.01022
62.720	0.01022
62.720	0.01022
62.721	0.01022
62.721	0.01023
62.722	0.01024
62.722	0.01025
62.723	0.01026
62.723	0.01027
62.724	0.01027
62.724	0.01028
62.725	0.01029
62.726	0.01029
62.726	0.01029
62.727	0.0103

62.727	0.0103
62.728	0.0103
62.728	0.0103
62.729	0.0103
62.729	0.0103
62.730	0.01029
62.731	0.01029
62.731	0.01028
62.732	0.01028
62.732	0.01027
62.733	0.01026
62.733	0.01025
62.734	0.01024
62.734	0.01023
62.735	0.01022
62.736	0.0102
62.736	0.01019
62.737	0.01017
62.737	0.01015
62.738	0.01013
62.738	0.01012
62.739	0.0101
62.739	0.01007
62.740	0.01005
62.741	0.01003
62.741	0.01001
62.742	0.00998
62.742	0.00996
62.743	0.00994
62.743	0.00992
62.744	0.00989
62.744	0.00987
62.745	0.00985
62.746	0.00983
62.746	0.0098
62.747	0.00978
62.747	0.00975
62.748	0.00984
62.748	0.00973
62.749	0.00967
62.750	0.00964
62.750	0.00961
62.751	0.00958
62.751	0.00955
62.752	0.00952
62.752	0.00949
62.753	0.00946
62.754	0.00943
62.754	0.00949
62.755	0.00941
62.755	0.00946
62.756	0.00976
62.756	0.00949

62.757	0.00943
62.758	0.00963
62.758	0.00955
62.759	0.0096
62.759	0.00966
62.760	0.00971
62.761	0.00977
62.761	0.00982
62.762	0.00987
62.762	0.00993
62.763	0.00998
62.763	0.01003
62.764	0.01008
62.765	0.01013
62.765	0.01018
62.766	0.01023
62.766	0.01028
62.767	0.01032
62.768	0.01037
62.768	0.01042
62.769	0.01046
62.769	0.0105
62.770	0.01054
62.771	0.01058
62.771	0.01062
62.772	0.01066
62.772	0.0107
62.773	0.01073
62.774	0.01076
62.774	0.0108
62.775	0.01083
62.775	0.01086
62.776	0.01089
62.777	0.01091
62.777	0.01094
62.778	0.01096
62.778	0.01099
62.779	0.01101
62.780	0.01103
62.780	0.01105
62.781	0.01106
62.781	0.01108
62.782	0.01109
62.783	0.0111
62.783	0.01111
62.784	0.01112
62.785	0.01112
62.785	0.01113
62.786	0.01113
62.786	0.01113
62.787	0.01112
62.788	0.01112
62.788	0.01111

62.789	0.0111
62.789	0.01109
62.790	0.01108
62.791	0.01107
62.791	0.01105
62.792	0.01103
62.793	0.01101
62.793	0.01098
62.794	0.01096
62.794	0.01093
62.795	0.0109
62.796	0.01087
62.796	0.01084
62.797	0.0108
62.798	0.01076
62.798	0.01072
62.799	0.01068
62.799	0.01064
62.800	0.0106
62.801	0.01055
62.801	0.0105
62.802	0.01045
62.803	0.0104
62.803	0.01035
62.804	0.01029
62.805	0.01024
62.805	0.01018
62.806	0.01021
62.806	0.0101
62.807	0.0101
62.808	0.0101
62.808	0.0101
62.809	0.01009
62.810	0.01009
62.810	0.01008
62.811	0.01007
62.812	0.01006
62.812	0.01005
62.813	0.01004
62.813	0.01002
62.814	0.01001
62.815	0.00999
62.815	0.00997
62.816	0.00995
62.817	0.00993
62.817	0.00991
62.818	0.00989
62.819	0.00986
62.819	0.00984
62.820	0.00981
62.821	0.00978
62.821	0.00975
62.822	0.00972

62.823	0.00969
62.823	0.00965
62.824	0.00964
62.825	0.00958
62.825	0.00955
62.826	0.00951
62.827	0.00947
62.827	0.00943
62.828	0.00939
62.828	0.00935
62.829	0.0093
62.830	0.00926
62.830	0.00921
62.831	0.00917
62.832	0.00912
62.832	0.00907
62.833	0.00902
62.834	0.00907
62.834	0.009
62.835	0.00902
62.836	0.00884
62.836	0.00889
62.837	0.00876
62.838	0.00872
62.838	0.00869
62.839	0.00863
62.840	0.00887
62.841	0.00861
62.841	0.00863
62.842	0.00861
62.843	0.00843
62.843	0.00841
62.844	0.00835
62.845	0.00832
62.845	0.00828
62.846	0.00824
62.847	0.00832
62.847	0.00817
62.848	0.00816
62.849	0.00808
62.849	0.00804
62.850	0.008
62.851	0.00798
62.851	0.00791
62.852	0.00789
62.853	0.00782
62.853	0.00777
62.854	0.00773
62.855	0.00768
62.856	0.00763
62.856	0.00759
62.857	0.00758
62.858	0.00756

62.858	0.00755
62.859	0.00753
62.860	0.00751
62.860	0.00749
62.861	0.00747
62.862	0.00745
62.862	0.00743
62.863	0.00741
62.864	0.00739
62.865	0.00737
62.865	0.00734
62.866	0.00732
62.867	0.00729
62.867	0.00726
62.868	0.00724
62.869	0.00722
62.870	0.00719
62.870	0.00717
62.871	0.00714
62.872	0.00711
62.872	0.00709
62.873	0.00706
62.874	0.00703
62.875	0.007
62.875	0.00697
62.876	0.00694
62.877	0.0069
62.877	0.00687
62.878	0.00684
62.879	0.0068
62.880	0.00677
62.880	0.00674
62.881	0.00671
62.882	0.0067
62.882	0.00668
62.883	0.00666
62.884	0.00664
62.885	0.00662
62.885	0.0066
62.886	0.00658
62.887	0.00655
62.888	0.00653
62.888	0.0065
62.889	0.00647
62.890	0.00644
62.891	0.00641
62.891	0.00637
62.892	0.00634
62.893	0.0063
62.893	0.00626
62.894	0.00622
62.895	0.00618
62.896	0.00614

62.896	0.0061
62.897	0.00605
62.898	0.006
62.899	0.00596
62.899	0.00591
62.900	0.00586
62.901	0.00583
62.902	0.0058
62.902	0.00578
62.903	0.00576
62.904	0.00573
62.905	0.00571
62.905	0.00568
62.906	0.00566
62.907	0.00563
62.908	0.0056
62.908	0.00557
62.909	0.00555
62.910	0.00552
62.911	0.00549
62.911	0.00546
62.912	0.00543
62.913	0.0054
62.914	0.00536
62.914	0.00533
62.915	0.0053
62.916	0.00527
62.917	0.00526
62.918	0.00526
62.918	0.00526
62.919	0.00525
62.920	0.00525
62.921	0.00524
62.921	0.00523
62.922	0.00522
62.923	0.00521
62.924	0.0052
62.924	0.00518
62.925	0.00517
62.926	0.00518
62.927	0.00515
62.927	0.00512
62.928	0.0051
62.929	0.00508
62.930	0.00507
62.930	0.00507
62.931	0.00503
62.932	0.00506
62.933	0.00501
62.933	0.005
62.934	0.00501
62.935	0.00501
62.936	0.00502

62.936	0.00504
62.937	0.00503
62.938	0.00504
62.938	0.00505
62.939	0.00506
62.940	0.00506
62.941	0.00507
62.941	0.00508
62.942	0.00508
62.943	0.00509
62.944	0.00509
62.944	0.0051
62.945	0.00511
62.946	0.00511
62.947	0.00512
62.947	0.00512
62.948	0.00513
62.949	0.00513
62.950	0.00514
62.950	0.00514
62.951	0.00514
62.952	0.00515
62.952	0.00515
62.953	0.00515
62.954	0.00516
62.955	0.00516
62.955	0.00516
62.956	0.00516
62.957	0.00516
62.958	0.00517
62.958	0.00517
62.959	0.00517
62.960	0.00517
62.960	0.00517
62.961	0.00516
62.962	0.00516
62.963	0.00516
62.963	0.00516
62.964	0.00516
62.965	0.00515
62.966	0.00515
62.966	0.00515
62.967	0.00514
62.968	0.00514
62.968	0.00513
62.969	0.00513
62.970	0.00512
62.971	0.00512
62.971	0.00511
62.972	0.0051
62.973	0.0051
62.974	0.00509
62.974	0.00508

62.975	0.00507
62.976	0.00506
62.977	0.00506
62.977	0.00505
62.978	0.00504
62.979	0.00503
62.979	0.00502
62.980	0.00501
62.981	0.00499
62.982	0.00498
62.982	0.00497
62.983	0.00496
62.984	0.00495
62.985	0.00493
62.985	0.00492
62.986	0.00491
62.987	0.00489
62.988	0.00488
62.988	0.00487
62.989	0.00485
62.990	0.00484
62.991	0.00482
62.991	0.00481
62.992	0.00479
62.993	0.00478
62.994	0.00476
62.994	0.00475
62.995	0.00473
62.996	0.00472
62.997	0.0047
62.997	0.00468
62.998	0.00467
62.999	0.00465
63.000	0.00463
63.000	0.00462
63.001	0.00461
63.002	0.00461
63.003	0.00461
63.003	0.00461
63.004	0.00462
63.005	0.00462
63.006	0.00462
63.006	0.00462
63.007	0.00462
63.008	0.00462
63.009	0.00462
63.009	0.00462
63.010	0.00462
63.011	0.00461
63.012	0.00461
63.013	0.0046
63.013	0.0046
63.014	0.00459

63.015	0.00459
63.016	0.00458
63.017	0.00457
63.017	0.00456
63.018	0.00455
63.019	0.00454
63.020	0.00453
63.021	0.00452
63.021	0.00451
63.022	0.0045
63.023	0.00449
63.024	0.00448
63.025	0.00448
63.025	0.00447
63.026	0.00447
63.027	0.00446
63.028	0.00445
63.029	0.00445
63.029	0.00444
63.030	0.00443
63.031	0.00442
63.032	0.00441
63.033	0.0044
63.034	0.00439
63.034	0.00438
63.035	0.00437
63.036	0.00436
63.037	0.00434
63.038	0.00434
63.039	0.00434
63.039	0.00434
63.040	0.00434
63.041	0.00433
63.042	0.00433
63.043	0.00433
63.044	0.00434
63.045	0.00434
63.045	0.00435
63.046	0.00436
63.047	0.00436
63.048	0.00437
63.049	0.00437
63.050	0.00437
63.051	0.00438
63.051	0.00438
63.052	0.00438
63.053	0.00437
63.054	0.00437
63.055	0.00437
63.056	0.00436
63.057	0.00436
63.058	0.00435
63.058	0.00434

63.059	0.00434
63.060	0.00433
63.061	0.00431
63.062	0.0043
63.063	0.00429
63.064	0.00427
63.065	0.00426
63.066	0.00424
63.067	0.00422
63.067	0.0042
63.068	0.00418
63.069	0.00416
63.070	0.00414
63.071	0.00411
63.072	0.00411
63.073	0.00411
63.074	0.0041
63.075	0.0041
63.076	0.00409
63.077	0.00409
63.078	0.00408
63.079	0.00408
63.079	0.00407
63.080	0.00406
63.081	0.00405
63.082	0.00404
63.083	0.00403
63.084	0.00401
63.085	0.004
63.086	0.00399
63.087	0.00397
63.088	0.00396
63.089	0.00396
63.090	0.00395
63.091	0.00395
63.092	0.00396
63.093	0.00398
63.094	0.004
63.095	0.00401
63.096	0.00403
63.097	0.00405
63.098	0.00406
63.099	0.00408
63.100	0.00409
63.101	0.0041
63.102	0.00412
63.103	0.00413
63.104	0.00414
63.105	0.00415
63.106	0.00415
63.107	0.00416
63.108	0.00417
63.109	0.00417

63.110	0.00417
63.111	0.00417
63.112	0.00417
63.113	0.00417
63.114	0.00416
63.115	0.00415
63.116	0.00414
63.117	0.00415
63.118	0.00416
63.119	0.00417
63.120	0.00418
63.121	0.00418
63.123	0.00418
63.124	0.00418
63.125	0.00418
63.126	0.00417
63.127	0.00416
63.128	0.00415
63.129	0.00413
63.130	0.00411
63.131	0.00409
63.132	0.00407
63.134	0.00404
63.135	0.00402
63.136	0.00399
63.137	0.00395
63.138	0.00392
63.139	0.00388
63.141	0.00384
63.142	0.0038
63.143	0.00375
63.144	0.00371
63.145	0.00366
63.147	0.00361
63.148	0.00356
63.149	0.0035
63.150	0.00345
63.151	0.00339
63.153	0.00333
63.154	0.00327
63.155	0.00321
63.156	0.00315
63.158	0.00309
63.159	0.00302
63.160	0.00296
63.162	0.00289
63.163	0.00282
63.164	0.00275
63.165	0.00268
63.167	0.00261
63.168	0.00257
63.169	0.00254
63.171	0.00253

63.172	0.00252
63.173	0.00251
63.175	0.00249
63.176	0.00248
63.177	0.00246
63.179	0.00244
63.180	0.00242
63.182	0.0024
63.183	0.00237
63.184	0.00235
63.186	0.00232
63.187	0.00228
63.189	0.00225
63.190	0.00223
63.191	0.0022
63.193	0.00218
63.194	0.00215
63.196	0.00211
63.197	0.00208
63.199	0.00204
63.200	0.002
63.201	0.00196
63.203	0.00192
63.204	0.0019
63.206	0.00187
63.207	0.00183
63.209	0.0018
63.210	0.00177
63.211	0.00173
63.213	0.0017
63.214	0.00167
63.216	0.00165
63.217	0.00163
63.219	0.0016
63.220	0.00158
63.222	0.00155
63.223	0.00153
63.224	0.00151
63.226	0.00149
63.227	0.00148
63.229	0.00147
63.230	0.00146
63.232	0.00146
63.233	0.00145
63.234	0.00144
63.236	0.00144
63.237	0.00144
63.239	0.00146
63.240	0.00148
63.242	0.00149
63.243	0.0015
63.244	0.00151
63.246	0.00151

63.247	0.00151
63.249	0.00151
63.250	0.0015
63.252	0.0015
63.253	0.00148
63.254	0.00147
63.256	0.00145
63.257	0.00143
63.259	0.00141
63.260	0.00139
63.262	0.00136
63.263	0.00136
63.265	0.00136
63.266	0.00136
63.267	0.00135
63.269	0.00135
63.270	0.00134
63.272	0.00134
63.273	0.00133
63.275	0.00134
63.276	0.00136
63.277	0.00139
63.279	0.00141
63.280	0.00143
63.282	0.00145
63.283	0.00146
63.285	0.00148
63.286	0.00149
63.288	0.0015
63.289	0.0015
63.290	0.00151
63.292	0.00151
63.293	0.00151
63.295	0.00151
63.296	0.00151
63.298	0.0015
63.299	0.00149
63.300	0.00148
63.302	0.00147
63.303	0.00146
63.305	0.00144
63.306	0.00143
63.308	0.00141
63.309	0.00139
63.311	0.00137
63.312	0.00135
63.313	0.00132
63.315	0.0013
63.316	0.00128
63.318	0.00125
63.319	0.00123
63.321	0.00125
63.322	0.00127

63.323	0.0013
63.325	0.00133
63.326	0.00135
63.328	0.00137
63.329	0.00139
63.331	0.00141
63.332	0.00142
63.334	0.00144
63.335	0.00145
63.336	0.00146
63.338	0.00146
63.339	0.00146
63.341	0.00146
63.342	0.00146
63.344	0.00145
63.345	0.00144
63.347	0.00143
63.348	0.00142
63.349	0.0014
63.351	0.00138
63.352	0.00136
63.354	0.00133
63.355	0.00131
63.357	0.00128
63.358	0.00125
63.360	0.00122
63.361	0.00121
63.362	0.00121
63.364	0.0012
63.365	0.00119
63.367	0.00119
63.368	0.00118
63.370	0.00118
63.371	0.00118
63.373	0.00119
63.374	0.00119
63.375	0.00119
63.377	0.00119
63.378	0.0012
63.380	0.00121
63.381	0.00121
63.383	0.00122
63.384	0.00122
63.386	0.00123
63.387	0.00123
63.389	0.00123
63.390	0.00123
63.391	0.00123
63.393	0.00122
63.394	0.00122
63.396	0.00122
63.397	0.00122
63.399	0.00122

63.400	0.00121
63.402	0.0012
63.403	0.00119
63.404	0.00118
63.406	0.00117
63.407	0.00116
63.409	0.00114
63.410	0.00112
63.412	0.00111
63.413	0.00109
63.415	0.00107
63.416	0.00105
63.418	0.00104
63.419	0.00102
63.420	0.00101
63.422	0.001
63.423	0.00102
63.425	0.00106
63.426	0.00106
63.428	0.00108
63.429	0.00111
63.431	0.00114
63.432	0.00116
63.434	0.00118
63.435	0.00121
63.436	0.00122
63.438	0.00124
63.439	0.00125
63.441	0.00127
63.442	0.00128
63.444	0.00128
63.445	0.00129
63.447	0.00129
63.448	0.00128
63.450	0.00128
63.451	0.00127
63.452	0.00126
63.454	0.00124
63.455	0.00123
63.457	0.00121
63.458	0.00118
63.460	0.00116
63.461	0.00113
63.463	0.0011
63.464	0.00106
63.466	0.00103
63.467	0.00099
63.468	0.00095
63.470	0.0009
63.471	0.00086
63.473	0.00081
63.474	0.00077
63.476	0.00075

63.477	0.00073
63.479	0.00072
63.480	0.0007
63.482	0.00068
63.483	0.00065
63.485	0.00063
63.486	0.0006
63.487	0.00057
63.489	0.00054
63.490	0.00051
63.492	0.00048
63.493	0.00044
63.495	0.00041
63.496	0.00037
63.498	0.00033
63.499	0.00029
63.501	0.00025
63.502	0.00023
63.504	0.00025
63.505	0.00026
63.507	0.00027
63.508	0.00028
63.509	0.00029
63.511	0.0003
63.512	0.0003
63.514	0.00031
63.515	0.00031
63.517	0.00032
63.518	0.00032
63.520	0.00033
63.521	0.00033
63.523	0.00034
63.524	0.00034
63.526	0.00035
63.527	0.00036
63.529	0.00037
63.530	0.00038
63.531	0.00039
63.533	0.00041
63.534	0.00043
63.536	0.00045
63.537	0.00047
63.539	0.00048
63.540	0.0005
63.542	0.00051
63.543	0.00053
63.545	0.00055
63.546	0.00056
63.548	0.00058
63.549	0.00059
63.551	0.0006
63.552	0.00062
63.554	0.00063

63.555	0.00064
63.556	0.00065
63.558	0.00067
63.559	0.00068
63.561	0.0007
63.562	0.00071
63.564	0.00073
63.565	0.00074
63.567	0.00075
63.568	0.00076
63.570	0.00077
63.571	0.00078
63.573	0.00079
63.574	0.0008
63.576	0.0008
63.577	0.00081
63.579	0.00082
63.580	0.00082
63.582	0.00083
63.583	0.00083
63.584	0.00085
63.586	0.00087
63.587	0.00088
63.589	0.0009
63.590	0.00092
63.592	0.00093
63.593	0.00094
63.595	0.00096
63.596	0.00097
63.598	0.00098
63.599	0.00099
63.601	0.001
63.602	0.00101
63.604	0.00102
63.605	0.00103
63.607	0.00103
63.608	0.00104
63.610	0.00104
63.611	0.00104
63.613	0.00105
63.614	0.00105
63.615	0.00105
63.617	0.00105
63.618	0.00104
63.620	0.00104
63.621	0.00104
63.623	0.00104
63.624	0.00103
63.626	0.00103
63.627	0.00102
63.629	0.00102
63.630	0.00101
63.632	0.00101

63.633	0.001
63.635	0.001
63.636	0.001
63.638	0.00099
63.639	0.001
63.641	0.00101
63.642	0.00101
63.644	0.00102
63.645	0.00103
63.647	0.00102
63.648	0.00103
63.650	0.00103
63.651	0.00103
63.652	0.00104
63.654	0.00104
63.655	0.00104
63.657	0.00104
63.658	0.00103
63.660	0.00103
63.661	0.00103
63.663	0.00103
63.664	0.00102
63.666	0.00102
63.667	0.00101
63.669	0.001
63.670	0.001
63.672	0.00099
63.673	0.00098
63.675	0.00097
63.676	0.00096
63.678	0.00095
63.679	0.00094
63.681	0.00092
63.682	0.00091
63.684	0.0009
63.685	0.00089
63.687	0.00088
63.688	0.00088
63.690	0.00088
63.691	0.00087
63.693	0.00087
63.694	0.00087
63.696	0.00086
63.697	0.00086
63.698	0.00085
63.700	0.00084
63.701	0.00084
63.703	0.00083
63.704	0.00082
63.706	0.00081
63.707	0.0008
63.709	0.0008
63.710	0.00079

63.712	0.00078
63.713	0.00076
63.715	0.00075
63.716	0.00074
63.718	0.00073
63.719	0.00072
63.721	0.00071
63.722	0.0007
63.724	0.00069
63.725	0.00067
63.727	0.00066
63.728	0.00065
63.730	0.00064
63.731	0.00063
63.733	0.00062
63.734	0.0006
63.736	0.00059
63.737	0.00058
63.739	0.00058
63.740	0.00056
63.742	0.00055
63.743	0.00054
63.745	0.00054
63.746	0.00054
63.748	0.00053
63.749	0.00053
63.751	0.00052
63.752	0.00052
63.754	0.00051
63.755	0.0005
63.757	0.0005
63.758	0.00049
63.760	0.00048
63.761	0.00047
63.763	0.00046
63.764	0.00045
63.766	0.00044
63.767	0.00043
63.769	0.00042
63.770	0.00041
63.772	0.00039
63.773	0.00038
63.775	0.00036
63.776	0.00035
63.778	0.00033
63.779	0.00031
63.781	0.00029
63.782	0.00027
63.783	0.00025
63.785	0.00023
63.786	0.00021
63.788	0.00019
63.789	0.00017

63.791	0.00015
63.792	0.00014
63.794	0.00012
63.795	0.0001
63.797	0.0001
63.798	0.0001
63.800	0.00011
63.801	0.00014
63.803	0.00016
63.804	0.00018
63.806	0.0002
63.807	0.00022
63.809	0.00024
63.810	0.00026
63.812	0.00028
63.813	0.00029
63.815	0.0003
63.816	0.00032
63.818	0.00033
63.819	0.00034
63.821	0.00035
63.822	0.00035
63.824	0.00036
63.825	0.00037
63.827	0.00037
63.828	0.00037
63.830	0.00038
63.831	0.00038
63.833	0.00038
63.834	0.00039
63.836	0.0004
63.837	0.00041
63.839	0.00043
63.840	0.00044
63.842	0.00045
63.843	0.00046
63.845	0.00047
63.846	0.00047
63.848	0.00049
63.849	0.00049
63.851	0.0005
63.852	0.0005
63.854	0.00053
63.855	0.00054
63.857	0.00052
63.858	0.00053
63.860	0.00053
63.862	0.00053
63.863	0.00054
63.865	0.00054
63.866	0.00055
63.868	0.00059
63.869	0.00056

63.871	0.00056
63.872	0.00056
63.874	0.00057
63.875	0.00057
63.877	0.00058
63.878	0.00061
63.880	0.00058
63.881	0.00059
63.883	0.00059
63.884	0.00059
63.886	0.00061
63.887	0.00059
63.889	0.0006
63.890	0.00061
63.892	0.00062
63.893	0.00063
63.895	0.00063
63.896	0.00064
63.898	0.00064
63.899	0.00065
63.901	0.00067
63.902	0.00068
63.904	0.00069
63.905	0.0007
63.907	0.0007
63.908	0.00071
63.910	0.00072
63.911	0.00073
63.913	0.00073
63.914	0.00074
63.916	0.00074
63.917	0.00075
63.919	0.00075
63.920	0.00075
63.922	0.00076
63.923	0.00076
63.925	0.00076
63.926	0.00076
63.928	0.00076
63.929	0.00076
63.931	0.00076
63.932	0.00076
63.934	0.00075
63.935	0.00075
63.937	0.00076
63.938	0.00076
63.940	0.00077
63.942	0.00077
63.943	0.00078
63.945	0.00078
63.946	0.00079
63.948	0.00079
63.949	0.00079

63.951	0.00079
63.952	0.0008
63.954	0.0008
63.955	0.0008
63.957	0.0008
63.958	0.0008
63.960	0.00079
63.961	0.00079
63.963	0.00079
63.964	0.00078
63.966	0.00078
63.967	0.00078
63.969	0.00077
63.970	0.00076
63.972	0.00075
63.973	0.00075
63.975	0.00074
63.976	0.00073
63.978	0.00072
63.979	0.00072
63.981	0.0007
63.982	0.0007
63.984	0.00068
63.985	0.00067
63.987	0.00066
63.989	0.00068
63.990	0.00066
63.992	0.00065
63.993	0.00065
63.995	0.00065
63.996	0.00065
63.998	0.00064
63.999	0.00064
64.001	0.00063
64.002	0.00063
64.004	0.00063
64.005	0.00062
64.007	0.00061
64.008	0.00061
64.010	0.0006
64.011	0.00059
64.013	0.00058
64.014	0.00058
64.016	0.00057
64.017	0.00056
64.019	0.00056
64.020	0.00054
64.022	0.00053
64.023	0.00053
64.025	0.00051
64.027	0.00051
64.028	0.00048
64.030	0.00047

64.031	0.00047
64.033	0.00046
64.034	0.00045
64.036	0.00043
64.037	0.00042
64.039	0.00041
64.040	0.00041
64.042	0.0004
64.043	0.00041
64.045	0.00038
