# Supplementary material for: Gas explosion characteristics and spray control mechanism in underground square
Source: PLoS One. 2024 Apr 24;19(4):e0293421. doi: 10.1371/journal.pone.0293421 (PMC11042696; doi:10.1371/journal.pone.0293421)
Supplement: S3 File — The reference column reflects the value of explosion overpressure under different spray pressures, and the curve highlights the influence of different spray pressures on the propagation velocity of explosion overpressure. (PDF) [file pone.0293421.s003.pdf]

P_w	P_{max}	Overpressure propagation rate
MPa	MPa	$MPa \cdot s^{-1}$
0	0.399	0.00654
0.2	0.413	0.00679
0.3	0.341	0.0056
0.4	0.315	0.00518
0.5	0.293	0.00482
0.6	0.284	0.00466
0.7	0.286	0.00469
0.8	0.29	0.00476
0.9	0.291	0.00478
1.0	0.292	0.00479
1.1	0.294	0.00483
1.2	0.297	0.00488
1.3	0.299	0.00491
1.4	0.301	0.00494
1.5	0.302	0.00496
1.6	0.303	0.00498
