# Supplementary material for: Gas explosion characteristics and spray control mechanism in underground square
Source: PLoS One. 2024 Apr 24;19(4):e0293421. doi: 10.1371/journal.pone.0293421 (PMC11042696; doi:10.1371/journal.pone.0293421)
Supplement: S4 File — The black and red curve represent the change value of explosion overpressure with time under no spray system and optimal explosion suppression spray pressure, respectively. The green dots represent the representative time nodes of different stages of explosion under the optimal explosion suppression spray pressure. (PDF) [file pone.0293421.s004.pdf]

P_w =0MPa		P_w =0.6MPa	
t	*P*	*t*	*P*
s	MPa	s	MPa
60.000	0.00332	60.000	0.00332
60.000	0.00927	60.000	0.00927
60.002	0.02421	60.002	0.02421
60.005	0.01908	60.005	0.01908
60.008	0.01626	60.008	0.01626
60.010	0.01922	60.010	0.01922
60.012	0.02228	60.012	0.02228
60.014	0.02316	60.014	0.02316
60.016	0.02319	60.016	0.02319
60.018	0.02333	60.018	0.02333
60.020	0.02372	60.020	0.02372
60.022	0.0241	60.022	0.0241
60.024	0.02434	60.024	0.02434
60.026	0.02449	60.026	0.02449
60.027	0.02459	60.027	0.02459
60.029	0.02468	60.029	0.02468
60.031	0.02475	60.031	0.02475
60.033	0.0248	60.033	0.0248
60.035	0.02483	60.035	0.02483
60.036	0.02485	60.036	0.02485
60.038	0.02486	60.038	0.02486
60.040	0.02486	60.040	0.02486
60.042	0.02485	60.042	0.02485
60.044	0.02483	60.044	0.02483
60.046	0.0248	60.046	0.0248
60.047	0.02476	60.047	0.02476
60.049	0.02472	60.049	0.02472
60.051	0.02467	60.051	0.02467
60.053	0.02462	60.053	0.02462
60.055	0.02457	60.055	0.02457
60.057	0.02451	60.057	0.02451
60.058	0.02445	60.058	0.02445
60.060	0.02438	60.060	0.02438
60.062	0.02432	60.062	0.02432
60.064	0.02425	60.064	0.02425
60.066	0.02419	60.066	0.02419
60.068	0.02412	60.068	0.02412
60.070	0.02406	60.070	0.02406
60.072	0.024	60.072	0.024
60.073	0.02393	60.073	0.02393
60.075	0.02387	60.075	0.02387
60.077	0.02381	60.077	0.02381
60.079	0.02375	60.079	0.02375
60.081	0.02369	60.081	0.02369
60.083	0.02363	60.083	0.02363
60.085	0.02357	60.085	0.02357
60.087	0.02351	60.087	0.02351
60.089	0.02345	60.089	0.02345
60.091	0.0234	60.091	0.0234
60.093	0.02334	60.093	0.02334

60.095	0.02329	60.095	0.02329
60.097	0.02323	60.097	0.02323
60.098	0.02318	60.098	0.0233
60.100	0.02312	60.100	0.02358
60.102	0.02307	60.102	0.02397
60.104	0.02302	60.104	0.02439
60.106	0.02297	60.106	0.02483
60.108	0.02291	60.108	0.02526
60.110	0.02286	60.109	0.02537
60.112	0.02281	60.111	0.0256
60.114	0.02276	60.113	0.02557
60.116	0.02271	60.114	0.02538
60.118	0.02266	60.116	0.02542
60.120	0.02261	60.118	0.02565
60.122	0.02256	60.120	0.02564
60.124	0.02251	60.121	0.02546
60.126	0.02245	60.123	0.02554
60.128	0.0224	60.125	0.02545
60.130	0.02235	60.127	0.02559
60.132	0.0223	60.128	0.02554
60.134	0.02225	60.130	0.02536
60.136	0.0222	60.132	0.02546
60.138	0.02215	60.134	0.02539
60.140	0.0221	60.135	0.02557
60.142	0.02204	60.137	0.02556
60.144	0.02199	60.139	0.02542
60.146	0.02194	60.141	0.02556
60.148	0.02189	60.142	0.02553
60.151	0.02184	60.144	0.02539
60.153	0.02178	60.146	0.02554
60.155	0.02173	60.147	0.02553
60.157	0.02168	60.149	0.02541
60.159	0.02163	60.151	0.02558
60.161	0.02157	60.153	0.02559
60.163	0.02152	60.154	0.0255
60.165	0.02147	60.156	0.02534
60.167	0.02142	60.158	0.02551
60.169	0.02136	60.159	0.02552
60.171	0.02131	60.161	0.02545
60.173	0.02126	60.163	0.02532
60.175	0.02121	60.165	0.02551
60.178	0.02115	60.166	0.02555
60.180	0.0211	60.168	0.02551
60.182	0.02105	60.170	0.02541
60.184	0.021	60.172	0.02528
60.186	0.02094	60.173	0.02549
60.188	0.02089	60.175	0.02556
60.190	0.02084	60.177	0.02554
60.193	0.02079	60.178	0.02548
60.195	0.02074	60.180	0.02539
60.197	0.02069	60.182	0.02529
60.199	0.02065	60.184	0.0252
60.201	0.0206	60.185	0.02544

60.203	0.02056	60.187	0.02554
60.205	0.02051	60.189	0.02557
60.208	0.02047	60.190	0.02555
60.210	0.02043	60.192	0.02551
60.212	0.02039	60.194	0.02546
60.214	0.02035	60.195	0.02541
60.216	0.02031	60.197	0.02536
60.219	0.02027	60.199	0.02532
60.221	0.02027	60.201	0.02529
60.223	0.02037	60.202	0.02527
60.225	0.02051	60.204	0.02525
60.227	0.02068	60.206	0.02524
60.229	0.02085	60.207	0.02524
60.231	0.02103	60.209	0.02524
60.234	0.02121	60.211	0.02526
60.236	0.02139	60.213	0.02527
60.238	0.02157	60.214	0.0253
60.240	0.02176	60.216	0.02536
60.242	0.02194	60.218	0.02551
60.244	0.0221	60.219	0.02569
60.245	0.02225	60.221	0.02591
60.247	0.02239	60.223	0.02613
60.249	0.02254	60.224	0.02637
60.251	0.02266	60.226	0.02661
60.253	0.02278	60.227	0.02685
60.255	0.02289	60.229	0.02709
60.257	0.02301	60.231	0.02733
60.259	0.02312	60.232	0.02757
60.261	0.02323	60.234	0.0278
60.262	0.02334	60.235	0.02799
60.264	0.02345	60.237	0.02815
60.266	0.02354	60.238	0.02826
60.268	0.02363	60.240	0.02835
60.270	0.02372	60.241	0.02842
60.271	0.02381	60.243	0.02848
60.273	0.02389	60.244	0.02854
60.275	0.02398	60.245	0.02858
60.277	0.02406	60.247	0.02862
60.279	0.02415	60.248	0.02866
60.280	0.02423	60.250	0.02869
60.282	0.02431	60.251	0.02872
60.284	0.02439	60.253	0.02875
60.285	0.02434	60.254	0.02877
60.287	0.02434	60.256	0.02883
60.289	0.02437	60.257	0.0289
60.291	0.0243	60.259	0.02897
60.292	0.02429	60.260	0.02902
60.294	0.02432	60.262	0.02907
60.296	0.02426	60.263	0.02911
60.298	0.02425	60.264	0.02914
60.299	0.02429	60.266	0.02916
60.301	0.02423	60.267	0.02919
60.303	0.02423	60.269	0.02921

60.305	0.02429	60.270	0.02922
60.306	0.02424	60.272	0.02924
60.308	0.02426	60.273	0.02925
60.310	0.02432	60.274	0.02927
60.312	0.02428	60.276	0.02928
60.313	0.02431	60.277	0.02928
60.315	0.02425	60.279	0.02929
60.317	0.02427	60.280	0.0293
60.319	0.02421	60.282	0.0293
60.320	0.02423	60.283	0.02933
60.322	0.02418	60.284	0.02936
60.324	0.02421	60.286	0.0294
60.326	0.02416	60.287	0.02943
60.327	0.0242	60.289	0.02946
60.329	0.02416	60.290	0.02948
60.331	0.02421	60.291	0.02951
60.332	0.02418	60.293	0.02953
60.334	0.0241	60.294	0.02955
60.336	0.02413	60.296	0.02958
60.338	0.02409	60.297	0.0296
60.339	0.02415	60.299	0.02962
60.341	0.02414	60.300	0.02963
60.343	0.02408	60.301	0.02965
60.345	0.02413	60.303	0.02966
60.346	0.02412	60.304	0.02966
60.348	0.02406	60.306	0.02967
60.350	0.02413	60.307	0.02969
60.351	0.02412	60.308	0.02971
60.353	0.02408	60.310	0.02973
60.355	0.02401	60.311	0.02976
60.357	0.02407	60.312	0.02978
60.358	0.02408	60.314	0.02981
60.360	0.02404	60.315	0.02983
60.362	0.02399	60.317	0.02986
60.364	0.02407	60.318	0.02988
60.365	0.02409	60.319	0.0299
60.367	0.02407	60.321	0.02991
60.369	0.02404	60.322	0.02992
60.370	0.02399	60.324	0.02993
60.372	0.02394	60.325	0.02994
60.374	0.02404	60.326	0.02996
60.376	0.02407	60.328	0.02998
60.377	0.02416	60.329	0.02999
60.379	0.02427	60.330	0.03
60.381	0.02441	60.332	0.03001
60.382	0.02455	60.333	0.03002
60.384	0.02471	60.335	0.03003
60.386	0.02487	60.336	0.03005
60.387	0.02503	60.337	0.03006
60.389	0.02519	60.339	0.03007
60.390	0.02536	60.340	0.03008
60.392	0.02553	60.341	0.03009
60.394	0.0257	60.343	0.0301

60.395	0.02587	60.344	0.03011
60.397	0.02604	60.345	0.03012
60.398	0.0262	60.347	0.03013
60.400	0.02636	60.348	0.03013
60.401	0.02651	60.350	0.03014
60.403	0.02663	60.351	0.03015
60.404	0.02674	60.352	0.03015
60.406	0.02682	60.354	0.03016
60.407	0.02689	60.355	0.03017
60.409	0.02696	60.356	0.03018
60.410	0.02701	60.358	0.03018
60.412	0.02705	60.359	0.03019
60.413	0.02709	60.361	0.0302
60.415	0.02712	60.362	0.03021
60.416	0.02714	60.363	0.03022
60.418	0.02717	60.365	0.03024
60.419	0.02718	60.366	0.03025
60.420	0.0272	60.367	0.03026
60.422	0.02726	60.369	0.03028
60.423	0.02732	60.370	0.03029
60.425	0.02738	60.371	0.03031
60.426	0.02744	60.373	0.03033
60.428	0.02748	60.374	0.03034
60.429	0.02751	60.376	0.03036
60.431	0.02753	60.377	0.03039
60.432	0.02755	60.378	0.03041
60.434	0.02757	60.380	0.03043
60.435	0.02758	60.381	0.03046
60.436	0.02759	60.382	0.03049
60.438	0.0276	60.384	0.03052
60.439	0.02761	60.385	0.03055
60.441	0.02761	60.386	0.03058
60.442	0.02762	60.388	0.03062
60.444	0.02762	60.389	0.03065
60.445	0.02763	60.391	0.03069
60.446	0.02763	60.392	0.03073
60.448	0.02763	60.393	0.03078
60.449	0.02763	60.395	0.03082
60.451	0.02762	60.396	0.03087
60.452	0.02762	60.397	0.03092
60.454	0.02761	60.399	0.03098
60.455	0.02762	60.400	0.03103
60.456	0.02763	60.401	0.03109
60.458	0.02765	60.403	0.03116
60.459	0.02767	60.404	0.03122
60.461	0.02768	60.406	0.03129
60.462	0.02769	60.407	0.03137
60.463	0.0277	60.408	0.03144
60.465	0.02771	60.410	0.03153
60.466	0.02772	60.411	0.03161
60.468	0.02772	60.412	0.0317
60.469	0.02773	60.414	0.03179
60.471	0.02774	60.415	0.03189

60.472	0.02775	60.416	0.03199
60.473	0.02776	60.418	0.0321
60.475	0.02776	60.419	0.03221
60.476	0.02777	60.420	0.03233
60.478	0.02777	60.422	0.03245
60.479	0.02778	60.423	0.03258
60.480	0.02778	60.425	0.03271
60.482	0.02778	60.426	0.03285
60.483	0.02778	60.427	0.03299
60.485	0.02777	60.429	0.03314
60.486	0.02778	60.430	0.03329
60.487	0.02779	60.431	0.03344
60.489	0.0278	60.433	0.03361
60.490	0.02781	60.434	0.03377
60.492	0.02782	60.435	0.03394
60.493	0.02783	60.437	0.03411
60.494	0.02784	60.438	0.03428
60.496	0.02785	60.440	0.03446
60.497	0.02786	60.441	0.03464
60.498	0.02787	60.442	0.03482
60.500	0.02788	60.444	0.03501
60.501	0.02789	60.445	0.0352
60.503	0.02789	60.446	0.03539
60.504	0.0279	60.448	0.03558
60.505	0.02789	60.449	0.03577
60.507	0.02789	60.450	0.03597
60.508	0.02789	60.452	0.03617
60.510	0.02788	60.453	0.03637
60.511	0.02788	60.454	0.03658
60.512	0.02789	60.456	0.03678
60.514	0.0279	60.457	0.03699
60.515	0.0279	60.459	0.03721
60.516	0.0279	60.460	0.03742
60.518	0.0279	60.461	0.03764
60.519	0.0279	60.463	0.03785
60.521	0.02791	60.464	0.03807
60.522	0.02791	60.465	0.0383
60.523	0.02792	60.467	0.03852
60.525	0.02792	60.468	0.03875
60.526	0.02792	60.469	0.03897
60.527	0.02791	60.471	0.0392
60.529	0.02791	60.472	0.03943
60.530	0.02791	60.473	0.03966
60.531	0.02791	60.475	0.03989
60.533	0.02791	60.476	0.04012
60.534	0.02791	60.477	0.04035
60.536	0.02791	60.479	0.04058
60.537	0.02791	60.480	0.04081
60.538	0.0279	60.482	0.04104
60.540	0.0279	60.483	0.04127
60.541	0.0279	60.484	0.04149
60.542	0.02789	60.486	0.04172
60.544	0.02789	60.487	0.04194

60.545	0.02789	60.488	0.04217
60.547	0.02789	60.490	0.04239
60.548	0.02789	60.491	0.04261
60.549	0.02789	60.492	0.04284
60.551	0.02789	60.494	0.04306
60.552	0.02789	60.495	0.04328
60.553	0.02788	60.496	0.04349
60.555	0.02788	60.498	0.04371
60.556	0.02789	60.499	0.04392
60.557	0.02789	60.500	0.04413
60.559	0.02789	60.502	0.04436
60.560	0.02789	60.503	0.0447
60.562	0.02789	60.504	0.0451
60.563	0.0279	60.506	0.04553
60.564	0.0279	60.507	0.04599
60.566	0.0279	60.508	0.04645
60.567	0.02791	60.510	0.0469
60.568	0.02792	60.511	0.04736
60.570	0.02792	60.512	0.04782
60.571	0.02793	60.513	0.04828
60.572	0.02794	60.514	0.04872
60.574	0.02795	60.516	0.04916
60.575	0.02796	60.517	0.04959
60.577	0.02797	60.518	0.05001
60.578	0.02798	60.519	0.05042
60.579	0.02799	60.520	0.05082
60.581	0.02801	60.521	0.05122
60.582	0.02803	60.522	0.05162
60.583	0.02804	60.524	0.05201
60.585	0.02806	60.525	0.0524
60.586	0.02808	60.526	0.05278
60.587	0.0281	60.527	0.05315
60.589	0.02812	60.528	0.05352
60.590	0.02815	60.529	0.05388
60.592	0.02818	60.530	0.05424
60.593	0.0282	60.531	0.05459
60.594	0.02823	60.532	0.05492
60.596	0.02827	60.533	0.05525
60.597	0.0283	60.534	0.05558
60.598	0.02834	60.535	0.0559
60.600	0.02838	60.536	0.05621
60.601	0.02842	60.537	0.05651
60.602	0.02846	60.538	0.0568
60.604	0.0285	60.539	0.05708
60.605	0.02855	60.540	0.05736
60.607	0.0286	60.541	0.05763
60.608	0.02865	60.542	0.0579
60.609	0.02871	60.543	0.05815
60.611	0.02877	60.544	0.05841
60.612	0.02882	60.545	0.05865
60.613	0.02889	60.546	0.0589
60.615	0.02895	60.547	0.05913
60.616	0.02902	60.548	0.05936

60.617	0.02909	60.549	0.05959
60.619	0.02916	60.550	0.05981
60.620	0.02924	60.551	0.06003
60.622	0.02932	60.552	0.06025
60.623	0.0294	60.553	0.06046
60.624	0.02948	60.554	0.06067
60.626	0.02957	60.555	0.06089
60.627	0.02966	60.556	0.06114
60.628	0.02975	60.557	0.06141
60.630	0.02985	60.558	0.0617
60.631	0.02994	60.559	0.06203
60.632	0.03004	60.560	0.0624
60.634	0.03014	60.560	0.06283
60.635	0.03025	60.561	0.06329
60.637	0.03035	60.562	0.06379
60.638	0.03046	60.563	0.0643
60.639	0.03057	60.564	0.06484
60.641	0.03068	60.565	0.06539
60.642	0.03079	60.565	0.06596
60.643	0.03091	60.566	0.06653
60.645	0.03103	60.567	0.06711
60.646	0.03114	60.568	0.06769
60.647	0.03126	60.569	0.06827
60.649	0.03139	60.569	0.06884
60.650	0.03151	60.570	0.06941
60.651	0.03164	60.571	0.06997
60.653	0.03176	60.572	0.07052
60.654	0.03189	60.572	0.07107
60.656	0.03202	60.573	0.07162
60.657	0.03215	60.574	0.07216
60.658	0.03229	60.574	0.07269
60.660	0.03242	60.575	0.07322
60.661	0.03256	60.576	0.07374
60.662	0.0327	60.577	0.07425
60.664	0.03284	60.577	0.07476
60.665	0.03298	60.578	0.07526
60.666	0.03313	60.579	0.07574
60.668	0.03327	60.579	0.07622
60.669	0.03342	60.580	0.07668
60.670	0.03357	60.581	0.07714
60.672	0.03372	60.581	0.07759
60.673	0.03387	60.582	0.07803
60.675	0.03402	60.583	0.07846
60.676	0.03417	60.583	0.07887
60.677	0.03433	60.584	0.07928
60.679	0.03448	60.585	0.07967
60.680	0.03464	60.585	0.08004
60.681	0.03479	60.586	0.0804
60.683	0.03495	60.587	0.08074
60.684	0.03511	60.587	0.08106
60.685	0.03526	60.588	0.08136
60.687	0.03542	60.589	0.08164
60.688	0.03558	60.589	0.08191

60.689	0.03574	60.590	0.08216
60.691	0.03589	60.591	0.08245
60.692	0.03605	60.591	0.08275
60.694	0.03621	60.592	0.08308
60.695	0.03636	60.593	0.08343
60.696	0.03652	60.593	0.08379
60.698	0.03668	60.594	0.08416
60.699	0.03683	60.595	0.08455
60.700	0.03699	60.595	0.08494
60.702	0.03715	60.596	0.08534
60.703	0.0373	60.597	0.08575
60.704	0.03746	60.597	0.08616
60.706	0.03761	60.598	0.08656
60.707	0.03777	60.599	0.08698
60.708	0.03792	60.599	0.08739
60.710	0.03808	60.600	0.0878
60.711	0.03823	60.601	0.08821
60.712	0.03838	60.601	0.08862
60.714	0.03853	60.602	0.08903
60.715	0.03868	60.602	0.08944
60.717	0.03882	60.603	0.08985
60.718	0.03899	60.604	0.09025
60.719	0.0392	60.604	0.09066
60.721	0.03945	60.605	0.09106
60.722	0.03972	60.605	0.09147
60.723	0.04	60.606	0.09187
60.724	0.04029	60.607	0.09228
60.726	0.04059	60.607	0.09269
60.727	0.04089	60.608	0.0931
60.728	0.04119	60.608	0.09351
60.729	0.04149	60.609	0.09393
60.731	0.0418	60.609	0.09435
60.732	0.0421	60.610	0.09478
60.733	0.04239	60.611	0.09521
60.734	0.04269	60.611	0.09565
60.736	0.04299	60.612	0.09609
60.737	0.04329	60.612	0.09654
60.738	0.04358	60.613	0.09699
60.739	0.04388	60.613	0.09745
60.740	0.04418	60.614	0.09792
60.741	0.04447	60.614	0.0984
60.743	0.04476	60.615	0.09888
60.744	0.04506	60.615	0.09937
60.745	0.04535	60.616	0.09988
60.746	0.04564	60.616	0.10038
60.747	0.04592	60.617	0.1009
60.748	0.04621	60.617	0.10143
60.749	0.04649	60.618	0.10197
60.750	0.04676	60.618	0.10251
60.751	0.04704	60.619	0.10306
60.753	0.04731	60.619	0.10361
60.754	0.04758	60.620	0.1042
60.755	0.04784	60.620	0.10481

60.756	0.04811	60.621	0.10546
60.757	0.04837	60.621	0.10614
60.758	0.04863	60.622	0.10686
60.759	0.04888	60.622	0.10762
60.760	0.04914	60.623	0.10842
60.761	0.04939	60.623	0.10925
60.762	0.04965	60.623	0.11011
60.763	0.0499	60.624	0.111
60.764	0.05014	60.624	0.11189
60.765	0.05039	60.625	0.11276
60.766	0.05064	60.625	0.1136
60.767	0.05089	60.625	0.11442
60.768	0.05113	60.626	0.1152
60.769	0.05138	60.626	0.11596
60.770	0.05164	60.626	0.11669
60.771	0.05191	60.627	0.11739
60.772	0.05221	60.627	0.11808
60.773	0.05251	60.627	0.11874
60.774	0.05283	60.628	0.11938
60.775	0.05316	60.628	0.12002
60.776	0.0535	60.629	0.12064
60.777	0.05384	60.629	0.12125
60.778	0.0542	60.629	0.12186
60.779	0.05456	60.630	0.12245
60.780	0.05493	60.630	0.12304
60.781	0.05531	60.630	0.12362
60.781	0.05569	60.631	0.12419
60.782	0.05609	60.631	0.12474
60.783	0.05649	60.631	0.12528
60.784	0.05689	60.632	0.1258
60.785	0.05731	60.632	0.1263
60.786	0.05774	60.633	0.12677
60.787	0.05822	60.633	0.12723
60.788	0.05871	60.633	0.12769
60.788	0.05923	60.634	0.12815
60.789	0.05977	60.634	0.12863
60.790	0.06032	60.634	0.12913
60.791	0.06088	60.635	0.12965
60.791	0.06144	60.635	0.13021
60.792	0.06201	60.635	0.13079
60.793	0.06257	60.636	0.13139
60.794	0.06314	60.636	0.132
60.794	0.0637	60.636	0.13261
60.795	0.06426	60.637	0.13323
60.796	0.06482	60.637	0.13385
60.796	0.06538	60.637	0.13447
60.797	0.06593	60.637	0.13512
60.798	0.06648	60.638	0.13581
60.798	0.06702	60.638	0.13652
60.799	0.06756	60.638	0.13726
60.800	0.06809	60.638	0.13802
60.800	0.06862	60.639	0.13877
60.801	0.06914	60.639	0.13952

60.802	0.06966	60.639	0.14027
60.802	0.07017	60.639	0.141
60.803	0.07067	60.639	0.14174
60.804	0.07117	60.639	0.14247
60.804	0.07166	60.640	0.14319
60.805	0.07215	60.640	0.14391
60.806	0.07263	60.640	0.14462
60.806	0.0731	60.640	0.14533
60.807	0.07356	60.640	0.14603
60.808	0.07402	60.641	0.14672
60.808	0.07447	60.641	0.14741
60.809	0.07491	60.641	0.14809
60.809	0.07534	60.641	0.14876
60.810	0.07577	60.641	0.14942
60.811	0.07621	60.641	0.15007
60.811	0.07666	60.642	0.15072
60.812	0.07711	60.642	0.15136
60.813	0.07757	60.642	0.152
60.813	0.07803	60.642	0.15262
60.814	0.07849	60.642	0.15324
60.814	0.07895	60.643	0.15386
60.815	0.07941	60.643	0.15447
60.816	0.07988	60.643	0.15507
60.816	0.08034	60.643	0.15567
60.817	0.0808	60.643	0.15626
60.817	0.08126	60.644	0.15685
60.818	0.08172	60.644	0.15744
60.819	0.08217	60.644	0.15802
60.819	0.08262	60.644	0.1586
60.820	0.08308	60.644	0.15917
60.820	0.08353	60.644	0.15973
60.821	0.08397	60.645	0.16029
60.821	0.08442	60.645	0.16085
60.822	0.08487	60.645	0.1614
60.822	0.08531	60.645	0.16194
60.823	0.08576	60.645	0.16248
60.823	0.0862	60.646	0.16302
60.824	0.08665	60.646	0.16355
60.824	0.0871	60.646	0.16408
60.825	0.08755	60.646	0.16461
60.825	0.08801	60.646	0.16513
60.826	0.08847	60.646	0.16565
60.826	0.08893	60.647	0.16617
60.827	0.08939	60.647	0.16668
60.827	0.08987	60.647	0.16719
60.828	0.09034	60.647	0.1677
60.828	0.09083	60.647	0.1682
60.829	0.09133	60.648	0.1687
60.829	0.09183	60.648	0.16919
60.829	0.09234	60.648	0.16968
60.830	0.09285	60.648	0.17017
60.830	0.09337	60.648	0.17066
60.831	0.0939	60.648	0.17114

60.831	0.09443	60.649	0.17162
60.831	0.09496	60.649	0.1721
60.832	0.09549	60.649	0.17258
60.832	0.096	60.649	0.17305
60.832	0.09651	60.649	0.17352
60.833	0.09701	60.649	0.17398
60.833	0.0975	60.650	0.17444
60.833	0.09798	60.650	0.1749
60.834	0.09844	60.650	0.17535
60.834	0.0989	60.650	0.1758
60.834	0.09934	60.650	0.17625
60.835	0.09978	60.650	0.1767
60.835	0.10021	60.651	0.17714
60.836	0.10064	60.651	0.17759
60.836	0.10107	60.651	0.17803
60.836	0.10149	60.651	0.17847
60.837	0.10191	60.651	0.17891
60.837	0.10232	60.651	0.17935
60.837	0.10275	60.652	0.17978
60.838	0.10319	60.652	0.18022
60.838	0.10364	60.652	0.18065
60.838	0.10409	60.652	0.18108
60.839	0.10453	60.652	0.18151
60.839	0.10498	60.652	0.18193
60.839	0.10544	60.653	0.18236
60.840	0.10589	60.653	0.18278
60.840	0.10634	60.653	0.1832
60.840	0.1068	60.653	0.18362
60.841	0.10725	60.653	0.18404
60.841	0.1077	60.653	0.18446
60.841	0.10815	60.654	0.18488
60.841	0.1086	60.654	0.18529
60.842	0.10904	60.654	0.1857
60.842	0.10948	60.654	0.18612
60.842	0.10992	60.654	0.18653
60.843	0.11036	60.654	0.18693
60.843	0.11079	60.655	0.18734
60.843	0.11122	60.655	0.18774
60.843	0.11165	60.655	0.18815
60.844	0.11208	60.655	0.18855
60.844	0.1125	60.655	0.18894
60.844	0.11292	60.655	0.18934
60.844	0.11333	60.656	0.18974
60.845	0.11375	60.656	0.19013
60.845	0.11416	60.656	0.19052
60.845	0.11457	60.656	0.19091
60.845	0.11498	60.656	0.1913
60.846	0.11538	60.656	0.19169
60.846	0.11576	60.656	0.19207
60.846	0.11614	60.657	0.19246
60.846	0.1165	60.657	0.19284
60.846	0.11687	60.657	0.19322
60.846	0.11724	60.657	0.1936

60.847	0.1176	60.657	0.19398
60.847	0.11797	60.657	0.19436
60.847	0.11834	60.658	0.19473
60.847	0.11871	60.658	0.1951
60.847	0.11908	60.658	0.19548
60.847	0.11945	60.658	0.19585
60.848	0.11982	60.658	0.19622
60.848	0.12019	60.658	0.19658
60.848	0.12055	60.659	0.19695
60.848	0.12091	60.659	0.19731
60.848	0.12127	60.659	0.19768
60.848	0.12163	60.659	0.19804
60.849	0.12198	60.659	0.1984
60.849	0.12234	60.659	0.19875
60.849	0.12269	60.659	0.19911
60.849	0.12304	60.660	0.19946
60.849	0.12338	60.660	0.19982
60.850	0.12373	60.660	0.20017
60.850	0.12407	60.660	0.20052
60.850	0.12442	60.660	0.20087
60.850	0.12476	60.660	0.20121
60.850	0.1251	60.661	0.20156
60.850	0.12543	60.661	0.2019
60.851	0.12577	60.661	0.20225
60.851	0.1261	60.661	0.20259
60.851	0.12644	60.661	0.20293
60.851	0.12677	60.661	0.20326
60.851	0.1271	60.662	0.2036
60.851	0.12742	60.662	0.20393
60.852	0.12775	60.662	0.20426
60.852	0.12807	60.662	0.20459
60.852	0.1284	60.662	0.20492
60.852	0.12872	60.662	0.20525
60.852	0.12904	60.663	0.20557
60.852	0.12936	60.663	0.2059
60.853	0.12968	60.663	0.20622
60.853	0.12999	60.663	0.20655
60.853	0.13031	60.663	0.20687
60.853	0.13062	60.663	0.20719
60.853	0.13094	60.663	0.20751
60.853	0.13125	60.664	0.20783
60.853	0.13156	60.664	0.20815
60.854	0.13187	60.664	0.20847
60.854	0.13218	60.664	0.20878
60.854	0.13248	60.664	0.2091
60.854	0.13279	60.664	0.20941
60.854	0.13309	60.665	0.20972
60.855	0.13339	60.665	0.21004
60.855	0.13369	60.665	0.21035
60.855	0.13399	60.665	0.21065
60.855	0.13428	60.665	0.21096
60.855	0.13458	60.665	0.21127
60.855	0.13487	60.666	0.21157

60.856	0.13516	60.666	0.21188
60.856	0.13545	60.666	0.21218
60.856	0.13573	60.666	0.21249
60.856	0.13602	60.666	0.21279
60.856	0.1363	60.666	0.21309
60.856	0.13658	60.667	0.21338
60.857	0.13686	60.667	0.21368
60.857	0.13714	60.667	0.21397
60.857	0.13742	60.667	0.21426
60.857	0.1377	60.667	0.21455
60.857	0.13798	60.667	0.21484
60.857	0.13825	60.667	0.21512
60.857	0.13853	60.668	0.21541
60.858	0.1388	60.668	0.21569
60.858	0.13907	60.668	0.21597
60.858	0.13934	60.668	0.21625
60.858	0.13961	60.668	0.21653
60.858	0.13988	60.668	0.21681
60.858	0.14014	60.669	0.21708
60.859	0.14041	60.669	0.21736
60.859	0.14067	60.669	0.21763
60.859	0.14093	60.669	0.2179
60.859	0.14119	60.669	0.21817
60.859	0.14145	60.669	0.21844
60.859	0.14171	60.670	0.21871
60.860	0.14196	60.670	0.21898
60.860	0.14222	60.670	0.21925
60.860	0.14247	60.670	0.21951
60.860	0.14273	60.670	0.21977
60.860	0.14298	60.670	0.22003
60.860	0.14323	60.671	0.22029
60.860	0.14348	60.671	0.22055
60.861	0.14373	60.671	0.2208
60.861	0.14398	60.671	0.22106
60.861	0.14423	60.671	0.22131
60.861	0.14447	60.671	0.22156
60.861	0.14472	60.671	0.22181
60.861	0.14496	60.672	0.22205
60.862	0.1452	60.672	0.2223
60.862	0.14544	60.672	0.22254
60.862	0.14568	60.672	0.22279
60.862	0.14592	60.672	0.22303
60.862	0.14616	60.672	0.22327
60.862	0.1464	60.673	0.22352
60.862	0.14664	60.673	0.22376
60.863	0.14687	60.673	0.224
60.863	0.14711	60.673	0.22425
60.863	0.14734	60.673	0.22449
60.863	0.14758	60.673	0.22473
60.863	0.14781	60.674	0.22497
60.863	0.14804	60.674	0.2252
60.864	0.14827	60.674	0.22544
60.864	0.1485	60.674	0.22567

60.864	0.14873	60.674	0.22591
60.864	0.14896	60.674	0.22614
60.864	0.14918	60.674	0.22637
60.864	0.14941	60.675	0.2266
60.864	0.14963	60.675	0.22682
60.865	0.14986	60.675	0.22705
60.865	0.15008	60.675	0.22728
60.865	0.15031	60.675	0.22751
60.865	0.15053	60.675	0.22774
60.865	0.15075	60.676	0.22797
60.865	0.15097	60.676	0.22819
60.866	0.1512	60.676	0.22842
60.866	0.15142	60.676	0.22865
60.866	0.15164	60.676	0.22887
60.866	0.15186	60.676	0.22909
60.866	0.15208	60.676	0.22932
60.866	0.1523	60.677	0.22954
60.867	0.15253	60.677	0.22975
60.867	0.15275	60.677	0.22997
60.867	0.15297	60.677	0.23018
60.867	0.15318	60.677	0.2304
60.867	0.1534	60.677	0.23061
60.867	0.15362	60.678	0.23081
60.867	0.15384	60.678	0.23102
60.868	0.15406	60.678	0.23122
60.868	0.15428	60.678	0.23143
60.868	0.15449	60.678	0.23163
60.868	0.15471	60.678	0.23183
60.868	0.15493	60.679	0.23202
60.868	0.15514	60.679	0.23222
60.869	0.15536	60.679	0.23241
60.869	0.15557	60.679	0.2326
60.869	0.15579	60.679	0.23279
60.869	0.156	60.679	0.23298
60.869	0.15621	60.679	0.23317
60.869	0.15643	60.680	0.23335
60.869	0.15664	60.680	0.23354
60.870	0.15685	60.680	0.23372
60.870	0.15707	60.680	0.23391
60.870	0.15728	60.680	0.23409
60.870	0.15749	60.680	0.23428
60.870	0.1577	60.681	0.23446
60.870	0.15792	60.681	0.23464
60.870	0.15813	60.681	0.23482
60.871	0.15834	60.681	0.235
60.871	0.15855	60.681	0.23518
60.871	0.15876	60.681	0.23535
60.871	0.15897	60.681	0.23553
60.871	0.15918	60.682	0.2357
60.871	0.15939	60.682	0.23588
60.872	0.1596	60.682	0.23605
60.872	0.15981	60.682	0.23622
60.872	0.16002	60.682	0.23639

60.872	0.16023	60.682	0.23656
60.872	0.16044	60.683	0.23672
60.872	0.16065	60.683	0.23689
60.872	0.16085	60.683	0.23706
60.873	0.16106	60.683	0.23723
60.873	0.16127	60.683	0.23739
60.873	0.16147	60.683	0.23756
60.873	0.16168	60.684	0.23773
60.873	0.16188	60.684	0.2379
60.873	0.16209	60.684	0.23806
60.873	0.16229	60.684	0.23823
60.874	0.1625	60.684	0.2384
60.874	0.1627	60.684	0.23856
60.874	0.16291	60.684	0.23873
60.874	0.16311	60.685	0.23889
60.874	0.16331	60.685	0.23906
60.874	0.16351	60.685	0.23923
60.875	0.16372	60.685	0.23939
60.875	0.16392	60.685	0.23955
60.875	0.16412	60.685	0.23972
60.875	0.16432	60.686	0.23988
60.875	0.16452	60.686	0.24004
60.875	0.16473	60.686	0.2402
60.875	0.16493	60.686	0.24035
60.876	0.16513	60.686	0.24051
60.876	0.16533	60.686	0.24067
60.876	0.16553	60.687	0.24083
60.876	0.16573	60.687	0.24098
60.876	0.16593	60.687	0.24114
60.876	0.16613	60.687	0.2413
60.876	0.16633	60.687	0.24145
60.877	0.16653	60.687	0.24161
60.877	0.16672	60.687	0.24176
60.877	0.16692	60.688	0.24192
60.877	0.16712	60.688	0.24207
60.877	0.16731	60.688	0.24222
60.877	0.16751	60.688	0.24238
60.877	0.1677	60.688	0.24253
60.878	0.1679	60.688	0.24268
60.878	0.16809	60.689	0.24284
60.878	0.16829	60.689	0.24299
60.878	0.16848	60.689	0.24314
60.878	0.16868	60.689	0.24329
60.878	0.16887	60.689	0.24344
60.878	0.16906	60.689	0.2436
60.879	0.16925	60.689	0.24375
60.879	0.16944	60.690	0.24389
60.879	0.16964	60.690	0.24404
60.879	0.16983	60.690	0.24419
60.879	0.17002	60.690	0.24434
60.879	0.1702	60.690	0.24448
60.879	0.17039	60.690	0.24463
60.880	0.17058	60.691	0.24478

60.880	0.17077	60.691	0.24492
60.880	0.17096	60.691	0.24507
60.880	0.17114	60.691	0.24521
60.880	0.17133	60.691	0.24536
60.880	0.17152	60.691	0.2455
60.880	0.1717	60.691	0.24564
60.881	0.17189	60.692	0.24579
60.881	0.17208	60.692	0.24593
60.881	0.17226	60.692	0.24607
60.881	0.17245	60.692	0.24622
60.881	0.17263	60.692	0.24636
60.881	0.17281	60.692	0.24651
60.881	0.173	60.693	0.24666
60.882	0.17318	60.693	0.24681
60.882	0.17336	60.693	0.24696
60.882	0.17354	60.693	0.24711
60.882	0.17373	60.693	0.24725
60.882	0.17391	60.693	0.2474
60.882	0.17409	60.693	0.24755
60.882	0.17427	60.694	0.2477
60.883	0.17445	60.694	0.24785
60.883	0.17463	60.694	0.24799
60.883	0.17481	60.694	0.24814
60.883	0.17499	60.694	0.24829
60.883	0.17517	60.694	0.24844
60.883	0.17535	60.695	0.24859
60.883	0.17552	60.695	0.24874
60.884	0.1757	60.695	0.24888
60.884	0.17588	60.695	0.24903
60.884	0.17606	60.695	0.24918
60.884	0.17623	60.695	0.24933
60.884	0.17641	60.695	0.24948
60.884	0.17658	60.696	0.24963
60.884	0.17676	60.696	0.24978
60.884	0.17694	60.696	0.24993
60.885	0.17711	60.696	0.25008
60.885	0.17729	60.696	0.25023
60.885	0.17746	60.696	0.25038
60.885	0.17763	60.697	0.25053
60.885	0.17781	60.697	0.25068
60.885	0.17798	60.697	0.25083
60.885	0.17815	60.697	0.25098
60.886	0.17832	60.697	0.25113
60.886	0.1785	60.697	0.25128
60.886	0.17867	60.697	0.25143
60.886	0.17884	60.698	0.25158
60.886	0.17901	60.698	0.25173
60.886	0.17918	60.698	0.25188
60.886	0.17935	60.698	0.25203
60.887	0.17952	60.698	0.25218
60.887	0.17969	60.698	0.25233
60.887	0.17986	60.698	0.25247
60.887	0.18002	60.699	0.25262

60.887	0.18019	60.699	0.25277
60.887	0.18036	60.699	0.25292
60.887	0.18052	60.699	0.25307
60.888	0.18069	60.699	0.25322
60.888	0.18085	60.699	0.25338
60.888	0.18101	60.700	0.25353
60.888	0.18118	60.700	0.25367
60.888	0.18134	60.700	0.25382
60.888	0.1815	60.700	0.25397
60.888	0.18167	60.700	0.25412
60.889	0.18183	60.700	0.25427
60.889	0.18199	60.700	0.25441
60.889	0.18215	60.701	0.25456
60.889	0.18231	60.701	0.2547
60.889	0.18247	60.701	0.25485
60.889	0.18263	60.701	0.255
60.889	0.18279	60.701	0.25514
60.890	0.18295	60.701	0.25529
60.890	0.18311	60.701	0.25543
60.890	0.18326	60.702	0.25558
60.890	0.18342	60.702	0.25572
60.890	0.18358	60.702	0.25587
60.890	0.18373	60.702	0.25601
60.890	0.18388	60.702	0.25616
60.891	0.18404	60.702	0.2563
60.891	0.18419	60.702	0.25645
60.891	0.18434	60.703	0.25659
60.891	0.1845	60.703	0.25673
60.891	0.18465	60.703	0.25687
60.891	0.1848	60.703	0.25701
60.891	0.18495	60.703	0.25715
60.892	0.1851	60.703	0.25729
60.892	0.18525	60.703	0.25743
60.892	0.1854	60.704	0.25757
60.892	0.18555	60.704	0.25771
60.892	0.1857	60.704	0.25785
60.892	0.18584	60.704	0.25799
60.892	0.18599	60.704	0.25812
60.892	0.18614	60.704	0.25826
60.893	0.18629	60.705	0.25839
60.893	0.18643	60.705	0.25853
60.893	0.18658	60.705	0.25866
60.893	0.18673	60.705	0.25879
60.893	0.18687	60.705	0.25892
60.893	0.18702	60.705	0.25904
60.893	0.18716	60.705	0.25917
60.894	0.1873	60.706	0.2593
60.894	0.18745	60.706	0.25942
60.894	0.18759	60.706	0.25955
60.894	0.18773	60.706	0.25967
60.894	0.18787	60.706	0.25979
60.894	0.18801	60.706	0.25992
60.894	0.18815	60.706	0.26004

60.895	0.18829	60.707	0.26016
60.895	0.18843	60.707	0.26028
60.895	0.18857	60.707	0.2604
60.895	0.18871	60.707	0.26052
60.895	0.18885	60.707	0.26064
60.895	0.18899	60.707	0.26075
60.895	0.18913	60.707	0.26087
60.896	0.18927	60.708	0.26098
60.896	0.18941	60.708	0.2611
60.896	0.18954	60.708	0.26121
60.896	0.18968	60.708	0.26132
60.896	0.18982	60.708	0.26143
60.896	0.18995	60.708	0.26155
60.896	0.19009	60.708	0.26166
60.897	0.19022	60.709	0.26176
60.897	0.19036	60.709	0.26187
60.897	0.19049	60.709	0.26198
60.897	0.19063	60.709	0.26208
60.897	0.19076	60.709	0.26219
60.897	0.19089	60.709	0.26229
60.897	0.19102	60.709	0.26239
60.897	0.19116	60.710	0.26249
60.898	0.19129	60.710	0.26259
60.898	0.19142	60.710	0.26269
60.898	0.19155	60.710	0.26278
60.898	0.19168	60.710	0.26288
60.898	0.19181	60.710	0.26297
60.898	0.19194	60.710	0.26306
60.898	0.19206	60.711	0.26315
60.899	0.19219	60.711	0.26324
60.899	0.19232	60.711	0.26333
60.899	0.19245	60.711	0.26342
60.899	0.19257	60.711	0.26351
60.899	0.1927	60.711	0.26359
60.899	0.19282	60.711	0.26368
60.899	0.19295	60.712	0.26376
60.900	0.19307	60.712	0.26384
60.900	0.19319	60.712	0.26392
60.900	0.19332	60.712	0.264
60.900	0.19344	60.712	0.26408
60.900	0.19356	60.712	0.26416
60.900	0.19368	60.713	0.26424
60.900	0.1938	60.713	0.26432
60.900	0.19392	60.713	0.26439
60.901	0.19404	60.713	0.26447
60.901	0.19416	60.713	0.26454
60.901	0.19428	60.713	0.26462
60.901	0.1944	60.713	0.26469
60.901	0.19452	60.714	0.26477
60.901	0.19464	60.714	0.26484
60.901	0.19476	60.714	0.26491
60.902	0.19488	60.714	0.26498
60.902	0.19499	60.714	0.26505

60.902	0.19511	60.714	0.26512
60.902	0.19523	60.714	0.26519
60.902	0.19534	60.715	0.26526
60.902	0.19546	60.715	0.26533
60.902	0.19558	60.715	0.2654
60.903	0.19569	60.715	0.26547
60.903	0.1958	60.715	0.26553
60.903	0.19592	60.715	0.2656
60.903	0.19603	60.715	0.26566
60.903	0.19614	60.716	0.26573
60.903	0.19626	60.716	0.26579
60.903	0.19637	60.716	0.26586
60.903	0.19648	60.716	0.26592
60.904	0.19659	60.716	0.26599
60.904	0.1967	60.716	0.26605
60.904	0.19681	60.716	0.26611
60.904	0.19692	60.717	0.26618
60.904	0.19703	60.717	0.26624
60.904	0.19714	60.717	0.2663
60.904	0.19725	60.717	0.26637
60.905	0.19736	60.717	0.26643
60.905	0.19746	60.717	0.26649
60.905	0.19757	60.717	0.26655
60.905	0.19768	60.718	0.26661
60.905	0.19778	60.718	0.26667
60.905	0.19789	60.718	0.26673
60.905	0.198	60.718	0.26679
60.906	0.1981	60.718	0.26685
60.906	0.19821	60.718	0.26691
60.906	0.19831	60.719	0.26697
60.906	0.19841	60.719	0.26703
60.906	0.19852	60.719	0.26708
60.906	0.19862	60.719	0.26714
60.906	0.19872	60.719	0.2672
60.906	0.19882	60.719	0.26725
60.907	0.19892	60.719	0.26731
60.907	0.19903	60.720	0.26737
60.907	0.19913	60.720	0.26742
60.907	0.19923	60.720	0.26747
60.907	0.19933	60.720	0.26753
60.907	0.19943	60.720	0.26758
60.907	0.19953	60.720	0.26764
60.908	0.19963	60.720	0.26769
60.908	0.19972	60.721	0.26775
60.908	0.19982	60.721	0.26781
60.908	0.19992	60.721	0.26787
60.908	0.20002	60.721	0.26793
60.908	0.20012	60.721	0.26799
60.908	0.20022	60.721	0.26805
60.909	0.20032	60.721	0.26811
60.909	0.20041	60.722	0.26817
60.909	0.20051	60.722	0.26824
60.909	0.20061	60.722	0.2683

60.909	0.2007	60.722	0.26837
60.909	0.2008	60.722	0.26843
60.909	0.2009	60.722	0.2685
60.909	0.20099	60.723	0.26856
60.910	0.20109	60.723	0.26863
60.910	0.20118	60.723	0.2687
60.910	0.20128	60.723	0.26877
60.910	0.20137	60.723	0.26883
60.910	0.20147	60.723	0.2689
60.910	0.20156	60.723	0.26897
60.910	0.20166	60.724	0.26904
60.911	0.20175	60.724	0.2691
60.911	0.20184	60.724	0.26917
60.911	0.20194	60.724	0.26924
60.911	0.20203	60.724	0.26931
60.911	0.20212	60.724	0.26938
60.911	0.20221	60.724	0.26944
60.911	0.20231	60.725	0.26951
60.912	0.2024	60.725	0.26958
60.912	0.20249	60.725	0.26965
60.912	0.20258	60.725	0.26972
60.912	0.20267	60.725	0.26978
60.912	0.20276	60.725	0.26985
60.912	0.20285	60.725	0.26992
60.912	0.20294	60.726	0.26998
60.912	0.20303	60.726	0.27005
60.913	0.20312	60.726	0.27012
60.913	0.20321	60.726	0.27018
60.913	0.2033	60.726	0.27025
60.913	0.20339	60.726	0.27031
60.913	0.20348	60.726	0.27037
60.913	0.20357	60.727	0.27044
60.913	0.20366	60.727	0.2705
60.914	0.20374	60.727	0.27056
60.914	0.20383	60.727	0.27062
60.914	0.20392	60.727	0.27069
60.914	0.204	60.727	0.27075
60.914	0.20409	60.728	0.27081
60.914	0.20418	60.728	0.27086
60.914	0.20426	60.728	0.27092
60.915	0.20435	60.728	0.27098
60.915	0.20443	60.728	0.27104
60.915	0.20452	60.728	0.27109
60.915	0.2046	60.728	0.27115
60.915	0.20469	60.729	0.2712
60.915	0.20477	60.729	0.27126
60.915	0.20485	60.729	0.27131
60.915	0.20494	60.729	0.27137
60.916	0.20502	60.729	0.27142
60.916	0.2051	60.729	0.27147
60.916	0.20518	60.729	0.27152
60.916	0.20527	60.730	0.27158
60.916	0.20535	60.730	0.27163

60.916	0.20543	60.730	0.27168
60.916	0.20551	60.730	0.27173
60.917	0.20559	60.730	0.27178
60.917	0.20567	60.730	0.27183
60.917	0.20575	60.730	0.27187
60.917	0.20583	60.731	0.27192
60.917	0.20591	60.731	0.27197
60.917	0.20599	60.731	0.27201
60.917	0.20607	60.731	0.27206
60.918	0.20614	60.731	0.27211
60.918	0.20622	60.731	0.27215
60.918	0.20777	60.732	0.2722
60.918	0.20973	60.732	0.27224
60.918	0.21159	60.732	0.27229
60.918	0.21335	60.732	0.27233
60.918	0.21501	60.732	0.27237
60.918	0.21656	60.732	0.27241
60.919	0.218	60.732	0.27246
60.919	0.21934	60.733	0.2725
60.919	0.22058	60.733	0.27254
60.919	0.22171	60.733	0.27258
60.919	0.22274	60.733	0.27261
60.919	0.22366	60.733	0.27265
60.919	0.22449	60.733	0.27269
60.920	0.22521	60.733	0.27273
60.920	0.22584	60.734	0.27276
60.920	0.22638	60.734	0.2728
60.920	0.22683	60.734	0.27283
60.920	0.22718	60.734	0.27286
60.920	0.22746	60.734	0.2729
60.920	0.22764	60.734	0.27293
60.921	0.22775	60.735	0.27296
60.921	0.22777	60.735	0.27298
60.921	0.22771	60.735	0.27301
60.921	0.22758	60.735	0.27304
60.921	0.22739	60.735	0.27308
60.921	0.22713	60.735	0.27311
60.921	0.22679	60.735	0.27314
60.921	0.22636	60.736	0.27318
60.922	0.22583	60.736	0.27321
60.922	0.2252	60.736	0.27325
60.922	0.22446	60.736	0.27328
60.922	0.22361	60.736	0.27332
60.922	0.22263	60.736	0.27336
60.922	0.22152	60.736	0.27339
60.922	0.22027	60.737	0.27343
60.923	0.21889	60.737	0.27347
60.923	0.21735	60.737	0.2735
60.923	0.21566	60.737	0.27354
60.923	0.21382	60.737	0.27358
60.923	0.21183	60.737	0.27362
60.923	0.20969	60.737	0.27367
60.923	0.20911	60.738	0.27371

60.924	0.20917	60.738	0.27376
60.924	0.20923	60.738	0.27381
60.924	0.20929	60.738	0.27386
60.924	0.20935	60.738	0.27391
60.924	0.20941	60.738	0.27397
60.924	0.20947	60.738	0.27402
60.924	0.20953	60.739	0.27407
60.924	0.20959	60.739	0.27413
60.925	0.20965	60.739	0.27418
60.925	0.20971	60.739	0.27423
60.925	0.20977	60.739	0.27429
60.925	0.20983	60.739	0.27434
60.925	0.20989	60.740	0.2744
60.925	0.20994	60.740	0.27445
60.925	0.21	60.740	0.2745
60.926	0.21006	60.740	0.27456
60.926	0.21012	60.740	0.27461
60.926	0.21132	60.740	0.27466
60.926	0.21264	60.740	0.27472
60.926	0.21389	60.741	0.27477
60.926	0.21507	60.741	0.27483
60.926	0.21616	60.741	0.27489
60.927	0.21717	60.741	0.27494
60.927	0.21808	60.741	0.275
60.927	0.21888	60.741	0.27506
60.927	0.21957	60.741	0.27512
60.927	0.22014	60.742	0.27518
60.927	0.22059	60.742	0.27524
60.927	0.22091	60.742	0.2753
60.927	0.22111	60.742	0.27536
60.928	0.22118	60.742	0.27542
60.928	0.22112	60.742	0.27548
60.928	0.22092	60.742	0.27553
60.928	0.22059	60.742	0.27559
60.928	0.22012	60.743	0.27565
60.928	0.21952	60.743	0.27571
60.928	0.21878	60.743	0.27576
60.929	0.21792	60.743	0.27582
60.929	0.21692	60.743	0.27588
60.929	0.21581	60.743	0.27593
60.929	0.21459	60.743	0.27599
60.929	0.21325	60.744	0.27604
60.929	0.2118	60.744	0.2761
60.929	0.21158	60.744	0.27616
60.930	0.21164	60.744	0.27621
60.930	0.21169	60.744	0.27627
60.930	0.21174	60.744	0.27633
60.930	0.21179	60.744	0.27638
60.930	0.21184	60.745	0.27644
60.930	0.21189	60.745	0.27649
60.930	0.21194	60.745	0.27655
60.931	0.21199	60.745	0.27661
60.931	0.21204	60.745	0.27666

60.931	0.21209	60.745	0.27672
60.931	0.21214	60.745	0.27678
60.931	0.21219	60.746	0.27683
60.931	0.21224	60.746	0.27689
60.931	0.21229	60.746	0.27694
60.931	0.21234	60.746	0.277
60.932	0.21239	60.746	0.27705
60.932	0.21244	60.746	0.27711
60.932	0.21249	60.746	0.27716
60.932	0.21357	60.747	0.27722
60.932	0.21503	60.747	0.27727
60.932	0.21649	60.747	0.27732
60.932	0.21794	60.747	0.27737
60.933	0.21936	60.747	0.27743
60.933	0.22076	60.747	0.27748
60.933	0.22215	60.747	0.27753
60.933	0.2235	60.748	0.27758
60.933	0.22478	60.748	0.27763
60.933	0.226	60.748	0.27768
60.933	0.22713	60.748	0.27773
60.934	0.22817	60.748	0.27777
60.934	0.22909	60.748	0.27782
60.934	0.2299	60.748	0.27787
60.934	0.23058	60.749	0.27792
60.934	0.23113	60.749	0.27796
60.934	0.23153	60.749	0.27801
60.934	0.23208	60.749	0.27806
60.934	0.23246	60.749	0.2781
60.935	0.23266	60.749	0.27815
60.935	0.23264	60.749	0.27819
60.935	0.23241	60.749	0.27824
60.935	0.23199	60.750	0.27828
60.935	0.23156	60.750	0.27832
60.935	0.23093	60.750	0.27837
60.935	0.23015	60.750	0.27841
60.936	0.23077	60.750	0.27845
60.936	0.23191	60.750	0.27849
60.936	0.23302	60.750	0.27853
60.936	0.23411	60.751	0.27858
60.936	0.2352	60.751	0.27862
60.936	0.23627	60.751	0.27866
60.936	0.23728	60.751	0.2787
60.937	0.23822	60.751	0.27874
60.937	0.23908	60.751	0.27878
60.937	0.23987	60.751	0.27882
60.937	0.24057	60.752	0.27885
60.937	0.24118	60.752	0.27889
60.937	0.24168	60.752	0.27893
60.937	0.24207	60.752	0.27897
60.938	0.24234	60.752	0.27901
60.938	0.24248	60.752	0.27905
60.938	0.24247	60.752	0.27908
60.938	0.24236	60.753	0.27912

60.938	0.24204	60.753	0.27916
60.938	0.24146	60.753	0.27919
60.938	0.24059	60.753	0.27923
60.938	0.2394	60.753	0.27926
60.939	0.2383	60.753	0.2793
60.939	0.23708	60.753	0.27933
60.939	0.23558	60.753	0.27937
60.939	0.23379	60.754	0.2794
60.939	0.23243	60.754	0.27944
60.939	0.23084	60.754	0.27947
60.939	0.22902	60.754	0.2795
60.940	0.22702	60.754	0.27954
60.940	0.22489	60.754	0.27957
60.940	0.22273	60.754	0.27961
60.940	0.22151	60.755	0.27964
60.940	0.22041	60.755	0.27967
60.940	0.2193	60.755	0.27971
60.940	0.21808	60.755	0.27974
60.941	0.21674	60.755	0.27977
60.941	0.21554	60.755	0.2798
60.941	0.21559	60.755	0.27984
60.941	0.21563	60.756	0.27987
60.941	0.21567	60.756	0.2799
60.941	0.21572	60.756	0.27993
60.941	0.21705	60.756	0.27996
60.942	0.21872	60.756	0.28
60.942	0.22036	60.756	0.28003
60.942	0.22197	60.756	0.28006
60.942	0.22354	60.757	0.28009
60.942	0.22505	60.757	0.28012
60.942	0.22648	60.757	0.28015
60.942	0.22783	60.757	0.28018
60.942	0.22908	60.757	0.28021
60.943	0.23022	60.757	0.28024
60.943	0.23125	60.757	0.28027
60.943	0.23215	60.757	0.2803
60.943	0.23293	60.758	0.28033
60.943	0.23357	60.758	0.28036
60.943	0.23408	60.758	0.28038
60.943	0.23448	60.758	0.28041
60.944	0.23474	60.758	0.28044
60.944	0.23488	60.758	0.28047
60.944	0.23488	60.758	0.2805
60.944	0.23476	60.759	0.28052
60.944	0.23452	60.759	0.28055
60.944	0.23416	60.759	0.28058
60.944	0.23369	60.759	0.2806
60.945	0.23316	60.759	0.28063
60.945	0.23256	60.759	0.28066
60.945	0.23187	60.759	0.28069
60.945	0.23109	60.760	0.28071
60.945	0.23025	60.760	0.28074
60.945	0.22939	60.760	0.28077

60.945	0.22846	60.760	0.28079
60.946	0.22749	60.760	0.28082
60.946	0.22649	60.760	0.28084
60.946	0.22544	60.760	0.28087
60.946	0.22442	60.761	0.2809
60.946	0.22333	60.761	0.28092
60.946	0.22526	60.761	0.28095
60.946	0.22713	60.761	0.28097
60.946	0.22892	60.761	0.281
60.947	0.23061	60.761	0.28102
60.947	0.23217	60.761	0.28105
60.947	0.23358	60.762	0.28107
60.947	0.23483	60.762	0.2811
60.947	0.2359	60.762	0.28112
60.947	0.23676	60.762	0.28115
60.947	0.23741	60.762	0.28118
60.948	0.23785	60.762	0.2812
60.948	0.23809	60.762	0.28123
60.948	0.23811	60.762	0.28125
60.948	0.23785	60.763	0.28128
60.948	0.23727	60.763	0.2813
60.948	0.23634	60.763	0.28133
60.948	0.23518	60.763	0.28135
60.949	0.23385	60.763	0.28138
60.949	0.23234	60.763	0.28141
60.949	0.23086	60.763	0.28143
60.949	0.22949	60.764	0.28146
60.949	0.22798	60.764	0.28148
60.949	0.2262	60.764	0.28151
60.949	0.22398	60.764	0.28154
60.950	0.22387	60.764	0.28156
60.950	0.22553	60.764	0.28159
60.950	0.22723	60.764	0.28162
60.950	0.22895	60.765	0.28164
60.950	0.23067	60.765	0.28167
60.950	0.23235	60.765	0.2817
60.950	0.23397	60.765	0.28172
60.950	0.23551	60.765	0.28175
60.951	0.23694	60.765	0.28178
60.951	0.23823	60.765	0.28181
60.951	0.23937	60.766	0.28183
60.951	0.24031	60.766	0.28186
60.951	0.24105	60.766	0.28189
60.951	0.2416	60.766	0.28191
60.951	0.24203	60.766	0.28194
60.952	0.2423	60.766	0.28197
60.952	0.24241	60.766	0.282
60.952	0.24232	60.767	0.28203
60.952	0.24196	60.767	0.28205
60.952	0.24131	60.767	0.28208
60.952	0.24032	60.767	0.28211
60.952	0.23897	60.767	0.28214
60.953	0.23734	60.767	0.28216

60.953	0.23598	60.767	0.28219
60.953	0.23435	60.768	0.28222
60.953	0.23244	60.768	0.28225
60.953	0.23025	60.768	0.28227
60.953	0.22968	60.768	0.2823
60.953	0.22909	60.768	0.28233
60.953	0.22848	60.768	0.28235
60.954	0.22777	60.768	0.28238
60.954	0.22691	60.768	0.28241
60.954	0.22589	60.769	0.28244
60.954	0.2247	60.769	0.28246
60.954	0.22348	60.769	0.28249
60.954	0.22232	60.769	0.28252
60.954	0.22104	60.769	0.28254
60.955	0.21963	60.769	0.28257
60.955	0.21808	60.769	0.2826
60.955	0.21808	60.770	0.28262
60.955	0.21809	60.770	0.28265
60.955	0.21809	60.770	0.28268
60.955	0.21809	60.770	0.2827
60.955	0.2181	60.770	0.28273
60.956	0.2181	60.770	0.28275
60.956	0.2181	60.770	0.28278
60.956	0.21811	60.771	0.28281
60.956	0.21811	60.771	0.28283
60.956	0.21811	60.771	0.28286
60.956	0.21811	60.771	0.28288
60.956	0.21811	60.771	0.28291
60.957	0.21812	60.771	0.28293
60.957	0.21812	60.771	0.28296
60.957	0.21812	60.772	0.28298
60.957	0.21812	60.772	0.28301
60.957	0.21812	60.772	0.28303
60.957	0.21812	60.772	0.28306
60.957	0.21844	60.772	0.28308
60.957	0.21901	60.772	0.28311
60.958	0.2195	60.772	0.28313
60.958	0.21992	60.773	0.28316
60.958	0.22026	60.773	0.28318
60.958	0.22052	60.773	0.2832
60.958	0.22071	60.773	0.28323
60.958	0.22083	60.773	0.28325
60.958	0.22088	60.773	0.28328
60.959	0.22087	60.773	0.2833
60.959	0.22082	60.774	0.28332
60.959	0.22072	60.774	0.28334
60.959	0.22056	60.774	0.28337
60.959	0.22036	60.774	0.28339
60.959	0.22026	60.774	0.28341
60.959	0.22023	60.774	0.28343
60.960	0.22013	60.774	0.28345
60.960	0.21997	60.775	0.28348
60.960	0.21975	60.775	0.2835

60.960	0.21947	60.775	0.28352
60.960	0.21914	60.775	0.28354
60.960	0.21876	60.775	0.28356
60.960	0.21837	60.775	0.28358
60.961	0.21891	60.775	0.2836
60.961	0.21936	60.776	0.28361
60.961	0.21969	60.776	0.28363
60.961	0.21991	60.776	0.28365
60.961	0.22001	60.776	0.28367
60.961	0.22	60.776	0.28368
60.961	0.21988	60.776	0.2837
60.962	0.21965	60.776	0.28372
60.962	0.21932	60.777	0.28373
60.962	0.2189	60.777	0.28375
60.962	0.21838	60.777	0.28376
60.962	0.21812	60.777	0.28378
60.962	0.2182	60.777	0.28379
60.962	0.21841	60.777	0.2838
60.962	0.21862	60.777	0.28382
60.963	0.21879	60.778	0.28383
60.963	0.21891	60.778	0.28384
60.963	0.21897	60.778	0.28385
60.963	0.21896	60.778	0.28386
60.963	0.21892	60.778	0.28387
60.963	0.21882	60.778	0.28388
60.963	0.21866	60.778	0.28389
60.964	0.2184	60.779	0.2839
60.964	0.21814	60.779	0.28391
60.964	0.21815	60.779	0.28392
60.964	0.21815	60.779	0.28393
60.964	0.21816	60.779	0.28394
60.964	0.21816	60.779	0.28394
60.964	0.21816	60.779	0.28395
60.965	0.21817	60.780	0.28396
60.965	0.21817	60.780	0.28397
60.965	0.21818	60.780	0.28397
60.965	0.21818	60.780	0.28398
60.965	0.21818	60.780	0.28399
60.965	0.21819	60.780	0.28399
60.965	0.21819	60.780	0.284
60.966	0.2182	60.781	0.284
60.966	0.2182	60.781	0.28401
60.966	0.21821	60.781	0.28401
60.966	0.21821	60.781	0.28402
60.966	0.21822	60.781	0.28402
60.966	0.21823	60.781	0.28403
60.966	0.21823	60.781	0.28403
60.966	0.21824	60.782	0.28404
60.967	0.21824	60.782	0.28404
60.967	0.21825	60.782	0.28404
60.967	0.21826	60.782	0.28405
60.967	0.21826	60.782	0.28405
60.967	0.21827	60.782	0.28406

60.967	0.21827	60.782	0.28406
60.967	0.21828	60.783	0.28406
60.968	0.21829	60.783	0.28406
60.968	0.21829	60.783	0.28407
60.968	0.2183	60.783	0.28407
60.968	0.21831	60.783	0.28407
60.968	0.21831	60.783	0.28407
60.968	0.21832	60.783	0.28407
60.968	0.21833	60.784	0.28408
60.969	0.21833	60.784	0.28408
60.969	0.21834	60.784	0.28408
60.969	0.21834	60.784	0.28408
60.969	0.21835	60.784	0.28408
60.969	0.21836	60.784	0.28408
60.969	0.21836	60.784	0.28408
60.969	0.21837	60.785	0.28408
60.970	0.21838	60.785	0.28408
60.970	0.21838	60.785	0.28408
60.970	0.21839	60.785	0.28408
60.970	0.21839	60.785	0.28408
60.970	0.2184	60.785	0.28408
60.970	0.21841	60.785	0.28408
60.970	0.21841	60.786	0.28407
60.970	0.21842	60.786	0.28407
60.971	0.21842	60.786	0.28407
60.971	0.21843	60.786	0.28407
60.971	0.21843	60.786	0.28406
60.971	0.21844	60.786	0.28406
60.971	0.21844	60.786	0.28406
60.971	0.21845	60.787	0.28405
60.971	0.21845	60.787	0.28405
60.972	0.21846	60.787	0.28404
60.972	0.21846	60.787	0.28403
60.972	0.21847	60.787	0.28403
60.972	0.21847	60.787	0.28402
60.972	0.21848	60.787	0.28401
60.972	0.21848	60.788	0.284
60.972	0.21849	60.788	0.284
60.973	0.21849	60.788	0.28399
60.973	0.21849	60.788	0.28398
60.973	0.2185	60.788	0.28397
60.973	0.2185	60.788	0.28395
60.973	0.2185	60.789	0.28394
60.973	0.21851	60.789	0.28393
60.973	0.21851	60.789	0.28392
60.974	0.21851	60.789	0.2839
60.974	0.21852	60.789	0.28389
60.974	0.21852	60.789	0.28387
60.974	0.21852	60.789	0.28386
60.974	0.21852	60.790	0.28384
60.974	0.21852	60.790	0.28382
60.974	0.21853	60.790	0.28381
60.974	0.21853	60.790	0.28379

60.975	0.21853	60.790	0.28377
60.975	0.21853	60.790	0.28375
60.975	0.21853	60.790	0.28373
60.975	0.21853	60.791	0.28371
60.975	0.21853	60.791	0.28369
60.975	0.21853	60.791	0.28366
60.975	0.21853	60.791	0.28364
60.976	0.21853	60.791	0.28362
60.976	0.2193	60.791	0.28359
60.976	0.22017	60.791	0.28357
60.976	0.22102	60.792	0.28354
60.976	0.22186	60.792	0.28352
60.976	0.22267	60.792	0.28349
60.976	0.22348	60.792	0.28347
60.977	0.22428	60.792	0.28344
60.977	0.22507	60.792	0.28341
60.977	0.22586	60.792	0.28338
60.977	0.22664	60.793	0.28336
60.977	0.22742	60.793	0.28333
60.977	0.2282	60.793	0.2833
60.977	0.22898	60.793	0.28327
60.978	0.22977	60.793	0.28324
60.978	0.2306	60.793	0.28321
60.978	0.23148	60.794	0.28318
60.978	0.23244	60.794	0.28314
60.978	0.23341	60.794	0.28311
60.978	0.23438	60.794	0.28308
60.978	0.23535	60.794	0.28305
60.979	0.23632	60.794	0.28301
60.979	0.23731	60.794	0.28298
60.979	0.2383	60.795	0.28294
60.979	0.23927	60.795	0.28291
60.979	0.24024	60.795	0.28288
60.979	0.24119	60.795	0.28284
60.979	0.24213	60.795	0.28281
60.979	0.24307	60.795	0.28277
60.980	0.24399	60.795	0.28273
60.980	0.24491	60.796	0.2827
60.980	0.24585	60.796	0.28266
60.980	0.2468	60.796	0.28262
60.980	0.24782	60.796	0.28259
60.980	0.24891	60.796	0.28255
60.980	0.25002	60.796	0.28251
60.981	0.25113	60.796	0.28247
60.981	0.25225	60.797	0.28244
60.981	0.25336	60.797	0.2824
60.981	0.25447	60.797	0.28236
60.981	0.25558	60.797	0.28232
60.981	0.25669	60.797	0.28228
60.981	0.25779	60.797	0.28225
60.982	0.25888	60.798	0.28221
60.982	0.25996	60.798	0.28217
60.982	0.26104	60.798	0.28213

60.982	0.26211	60.798	0.2821
60.982	0.26317	60.798	0.28206
60.982	0.26423	60.798	0.28202
60.982	0.26527	60.798	0.28198
60.983	0.2663	60.799	0.28194
60.983	0.26731	60.799	0.2819
60.983	0.26833	60.799	0.28187
60.983	0.26933	60.799	0.28183
60.983	0.27033	60.799	0.28179
60.983	0.27131	60.799	0.28176
60.983	0.27229	60.799	0.28172
60.983	0.27327	60.800	0.28169
60.984	0.27424	60.800	0.28166
60.984	0.27521	60.800	0.28163
60.984	0.27618	60.800	0.2816
60.984	0.27715	60.800	0.28157
60.984	0.27811	60.800	0.28154
60.984	0.27906	60.801	0.28151
60.984	0.28	60.801	0.28148
60.985	0.28093	60.801	0.28145
60.985	0.28186	60.801	0.28143
60.985	0.28277	60.801	0.2814
60.985	0.28368	60.801	0.28138
60.985	0.28497	60.801	0.28135
60.985	0.28635	60.802	0.28133
60.985	0.28773	60.802	0.28131
60.986	0.28909	60.802	0.28129
60.986	0.29044	60.802	0.28127
60.986	0.29178	60.802	0.28125
60.986	0.29311	60.802	0.28123
60.986	0.29444	60.802	0.28122
60.986	0.29575	60.803	0.2812
60.986	0.29711	60.803	0.28118
60.987	0.29847	60.803	0.28117
60.987	0.29983	60.803	0.28115
60.987	0.3012	60.803	0.28114
60.987	0.30256	60.803	0.28112
60.987	0.30392	60.803	0.28111
60.987	0.30528	60.804	0.2811
60.987	0.30672	60.804	0.28109
60.987	0.30875	60.804	0.28107
60.988	0.31075	60.804	0.28106
60.988	0.31272	60.804	0.28105
60.988	0.31467	60.804	0.28105
60.988	0.31659	60.804	0.28104
60.988	0.31847	60.805	0.28103
60.988	0.32031	60.805	0.28102
60.988	0.32212	60.805	0.28101
60.989	0.32389	60.805	0.28101
60.989	0.32562	60.805	0.281
60.989	0.32731	60.805	0.28099
60.989	0.32896	60.806	0.28099
60.989	0.33057	60.806	0.28098

60.989	0.33213	60.806	0.28098
60.989	0.33365	60.806	0.28097
60.990	0.33513	60.806	0.28097
60.990	0.33657	60.806	0.28097
60.990	0.33797	60.806	0.28097
60.990	0.33934	60.807	0.28097
60.990	0.34066	60.807	0.28097
60.990	0.34195	60.807	0.28097
60.990	0.3432	60.807	0.28097
60.991	0.34442	60.807	0.28097
60.991	0.34561	60.807	0.28097
60.991	0.34676	60.807	0.28097
60.991	0.34789	60.808	0.28097
60.991	0.349	60.808	0.28098
60.991	0.35007	60.808	0.28098
60.991	0.35112	60.808	0.28098
60.991	0.35216	60.808	0.28099
60.992	0.35317	60.808	0.28099
60.992	0.35443	60.808	0.281
60.992	0.35595	60.809	0.281
60.992	0.35743	60.809	0.28101
60.992	0.35889	60.809	0.28102
60.992	0.36032	60.809	0.28102
60.992	0.36171	60.809	0.28103
60.993	0.36306	60.809	0.28104
60.993	0.36438	60.809	0.28105
60.993	0.36566	60.810	0.28106
60.993	0.3669	60.810	0.28106
60.993	0.3681	60.810	0.28107
60.993	0.36926	60.810	0.28108
60.993	0.37038	60.810	0.28109
60.994	0.37146	60.810	0.2811
60.994	0.37263	60.810	0.28111
60.994	0.37378	60.811	0.28112
60.994	0.37489	60.811	0.28113
60.994	0.37597	60.811	0.28114
60.994	0.37701	60.811	0.28115
60.994	0.37801	60.811	0.28116
60.995	0.37898	60.811	0.28118
60.995	0.37995	60.811	0.28119
60.995	0.38092	60.812	0.2812
60.995	0.38186	60.812	0.28121
60.995	0.38275	60.812	0.28122
60.995	0.3836	60.812	0.28123
60.995	0.38442	60.812	0.28124
60.995	0.38519	60.812	0.28125
60.996	0.38592	60.812	0.28126
60.996	0.38662	60.813	0.28128
60.996	0.38727	60.813	0.28129
60.996	0.38788	60.813	0.2813
60.996	0.38846	60.813	0.28131
60.996	0.389	60.813	0.28132
60.996	0.3895	60.813	0.28133

60.997	0.38996	60.813	0.28134
60.997	0.39039	60.814	0.28135
60.997	0.39078	60.814	0.28136
60.997	0.39114	60.814	0.28137
60.997	0.39147	60.814	0.28138
60.997	0.39176	60.814	0.28139
60.997	0.39203	60.814	0.2814
60.998	0.39226	60.815	0.28141
60.998	0.39255	60.815	0.28142
60.998	0.39294	60.815	0.28143
60.998	0.3933	60.815	0.28144
60.998	0.39363	60.815	0.28144
60.998	0.39392	60.815	0.28145
60.998	0.39418	60.815	0.28146
60.999	0.39442	60.816	0.28147
60.999	0.39464	60.816	0.28147
60.999	0.39484	60.816	0.28148
60.999	0.395	60.816	0.28149
60.999	0.39513	60.816	0.2815
60.999	0.39522	60.816	0.2815
60.999	0.39528	60.816	0.28151
60.999	0.3953	60.817	0.28151
61.000	0.39529	60.817	0.28152
61.000	0.39523	60.817	0.28153
61.000	0.39514	60.817	0.28153
61.000	0.39501	60.817	0.28153
61.000	0.39484	60.817	0.28154
61.000	0.39462	60.817	0.28154
61.000	0.39437	60.818	0.28155
61.001	0.39408	60.818	0.28155
61.001	0.39375	60.818	0.28155
61.001	0.39389	60.818	0.28156
61.001	0.39417	60.818	0.28156
61.001	0.39444	60.818	0.28156
61.001	0.39476	60.818	0.28156
61.001	0.39507	60.819	0.28157
61.002	0.39539	60.819	0.28157
61.002	0.3957	60.819	0.28157
61.002	0.396	60.819	0.28157
61.002	0.3963	60.819	0.28157
61.002	0.39658	60.819	0.28157
61.002	0.39686	60.819	0.28157
61.002	0.39711	60.820	0.28157
61.003	0.39736	60.820	0.28156
61.003	0.39759	60.820	0.28156
61.003	0.3978	60.820	0.28156
61.003	0.39798	60.820	0.28156
61.003	0.39814	60.820	0.28156
61.003	0.39828	60.820	0.28155
61.003	0.39839	60.821	0.28155
61.003	0.39848	60.821	0.28155
61.004	0.39853	60.821	0.28155
61.004	0.39855	60.821	0.28154

61.004	0.39854	60.821	0.28154
61.004	0.3985	60.821	0.28154
61.004	0.39842	60.821	0.28153
61.004	0.39831	60.822	0.28153
61.004	0.39817	60.822	0.28152
61.005	0.39799	60.822	0.28152
61.005	0.39777	60.822	0.28151
61.005	0.39752	60.822	0.28151
61.005	0.39723	60.822	0.2815
61.005	0.39691	60.822	0.2815
61.005	0.39655	60.823	0.28149
61.005	0.39616	60.823	0.28149
61.006	0.39574	60.823	0.28148
61.006	0.39528	60.823	0.28148
61.006	0.3948	60.823	0.28147
61.006	0.39431	60.823	0.28146
61.006	0.39381	60.823	0.28146
61.006	0.39329	60.824	0.28145
61.006	0.39278	60.824	0.28144
61.007	0.39227	60.824	0.28144
61.007	0.39177	60.824	0.28143
61.007	0.39125	60.824	0.28142
61.007	0.39072	60.824	0.28141
61.007	0.39018	60.824	0.28141
61.007	0.38962	60.825	0.2814
61.007	0.38905	60.825	0.28139
61.008	0.38846	60.825	0.28138
61.008	0.38786	60.825	0.28137
61.008	0.38724	60.825	0.28137
61.008	0.3866	60.825	0.28136
61.008	0.38593	60.825	0.28135
61.008	0.38525	60.826	0.28134
61.008	0.38455	60.826	0.28134
61.008	0.38382	60.826	0.28133
61.009	0.38308	60.826	0.28132
61.009	0.38231	60.826	0.28131
61.009	0.38153	60.826	0.2813
61.009	0.38072	60.826	0.28129
61.009	0.37989	60.827	0.28128
61.009	0.37905	60.827	0.28127
61.009	0.37818	60.827	0.28127
61.010	0.37729	60.827	0.28126
61.010	0.37639	60.827	0.28125
61.010	0.37546	60.827	0.28124
61.010	0.37451	60.827	0.28123
61.010	0.37355	60.828	0.28122
61.010	0.37256	60.828	0.28122
61.010	0.37156	60.828	0.28121
61.011	0.37053	60.828	0.2812
61.011	0.36949	60.828	0.28119
61.011	0.36843	60.828	0.28118
61.011	0.36735	60.828	0.28117
61.011	0.36626	60.829	0.28117

61.011	0.36515	60.829	0.28116
61.011	0.36415	60.829	0.28115
61.012	0.36318	60.829	0.28114
61.012	0.36218	60.829	0.28113
61.012	0.36116	60.829	0.28112
61.012	0.36011	60.830	0.28111
61.012	0.35905	60.830	0.28111
61.012	0.35806	60.830	0.2811
61.012	0.35709	60.830	0.28109
61.013	0.35612	60.830	0.28108
61.013	0.35512	60.830	0.28107
61.013	0.35411	60.830	0.28106
61.013	0.35309	60.831	0.28105
61.013	0.35205	60.831	0.28105
61.013	0.35104	60.831	0.28104
61.013	0.35001	60.831	0.28103
61.013	0.34896	60.831	0.28102
61.014	0.3479	60.831	0.28101
61.014	0.34683	60.831	0.281
61.014	0.34574	60.832	0.28099
61.014	0.34465	60.832	0.28098
61.014	0.34353	60.832	0.28098
61.014	0.34241	60.832	0.28097
61.014	0.34128	60.832	0.28096
61.015	0.34013	60.832	0.28095
61.015	0.33898	60.832	0.28094
61.015	0.33781	60.833	0.28093
61.015	0.33664	60.833	0.28092
61.015	0.33548	60.833	0.28091
61.015	0.33433	60.833	0.2809
61.015	0.33318	60.833	0.28089
61.016	0.33202	60.833	0.28088
61.016	0.33085	60.833	0.28087
61.016	0.32973	60.834	0.28086
61.016	0.3287	60.834	0.28085
61.016	0.3277	60.834	0.28084
61.016	0.32667	60.834	0.28083
61.016	0.32563	60.834	0.28082
61.017	0.32457	60.834	0.28081
61.017	0.3235	60.834	0.2808
61.017	0.32241	60.835	0.28079
61.017	0.3213	60.835	0.28078
61.017	0.32017	60.835	0.28076
61.017	0.31903	60.835	0.28075
61.017	0.31787	60.835	0.28074
61.018	0.31669	60.835	0.28073
61.018	0.3155	60.835	0.28072
61.018	0.31429	60.836	0.2807
61.018	0.31306	60.836	0.28069
61.018	0.31186	60.836	0.28068
61.018	0.31073	60.836	0.28066
61.018	0.30959	60.836	0.28065
61.019	0.30843	60.836	0.28064

61.019	0.30726	60.836	0.28062
61.019	0.30607	60.837	0.28061
61.019	0.30487	60.837	0.28059
61.019	0.30365	60.837	0.28058
61.019	0.30242	60.837	0.28057
61.019	0.30118	60.837	0.28055
61.020	0.29992	60.837	0.28054
61.020	0.29866	60.837	0.28052
61.020	0.29742	60.838	0.2805
61.020	0.29617	60.838	0.28049
61.020	0.29492	60.838	0.28047
61.020	0.29365	60.838	0.28046
61.020	0.29237	60.838	0.28044
61.021	0.29109	60.838	0.28042
61.021	0.28979	60.839	0.28041
61.021	0.28849	60.839	0.28039
61.021	0.28723	60.839	0.28037
61.021	0.28598	60.839	0.28036
61.021	0.28472	60.839	0.28034
61.021	0.28345	60.839	0.28032
61.022	0.28222	60.839	0.2803
61.022	0.28101	60.840	0.28029
61.022	0.2798	60.840	0.28027
61.022	0.27863	60.840	0.28025
61.022	0.27748	60.840	0.28023
61.022	0.27635	60.840	0.28021
61.023	0.27525	60.840	0.28019
61.023	0.27418	60.840	0.28017
61.023	0.27313	60.841	0.28015
61.023	0.27214	60.841	0.28013
61.023	0.27114	60.841	0.28012
61.023	0.27014	60.841	0.2801
61.023	0.26914	60.841	0.28008
61.024	0.26815	60.841	0.28006
61.024	0.26716	60.841	0.28004
61.024	0.26617	60.842	0.28001
61.024	0.26517	60.842	0.27999
61.024	0.26417	60.842	0.27997
61.024	0.26317	60.842	0.27995
61.024	0.26216	60.842	0.27993
61.025	0.26115	60.842	0.27991
61.025	0.26013	60.842	0.27989
61.025	0.25911	60.843	0.27987
61.025	0.25809	60.843	0.27985
61.025	0.25706	60.843	0.27982
61.025	0.25602	60.843	0.2798
61.025	0.25498	60.843	0.27978
61.026	0.25394	60.843	0.27976
61.026	0.25289	60.844	0.27973
61.026	0.25183	60.844	0.27971
61.026	0.25077	60.844	0.27969
61.026	0.24971	60.844	0.27967
61.026	0.24867	60.844	0.27964

61.027	0.24767	60.844	0.27962
61.027	0.24667	60.844	0.2796
61.027	0.24568	60.845	0.27957
61.027	0.24469	60.845	0.27955
61.027	0.24371	60.845	0.27953
61.027	0.24272	60.845	0.2795
61.027	0.24177	60.845	0.27948
61.028	0.24083	60.845	0.27945
61.028	0.23988	60.845	0.27943
61.028	0.23892	60.846	0.27941
61.028	0.23795	60.846	0.27938
61.028	0.23696	60.846	0.27936
61.028	0.23596	60.846	0.27934
61.028	0.23506	60.846	0.27931
61.029	0.23422	60.846	0.27929
61.029	0.23342	60.846	0.27927
61.029	0.23344	60.847	0.27924
61.029	0.23415	60.847	0.27922
61.029	0.2348	60.847	0.27919
61.029	0.23536	60.847	0.27917
61.029	0.23585	60.847	0.27915
61.030	0.23626	60.847	0.27912
61.030	0.23661	60.848	0.2791
61.030	0.23689	60.848	0.27907
61.030	0.23713	60.848	0.27905
61.030	0.23735	60.848	0.27903
61.030	0.23773	60.848	0.279
61.031	0.23843	60.848	0.27898
61.031	0.23905	60.848	0.27895
61.031	0.23959	60.849	0.27893
61.031	0.24005	60.849	0.27891
61.031	0.24044	60.849	0.27888
61.031	0.24074	60.849	0.27886
61.031	0.24095	60.849	0.27883
61.032	0.24107	60.849	0.27881
61.032	0.24108	60.849	0.27879
61.032	0.24099	60.850	0.27876
61.032	0.2408	60.850	0.27874
61.032	0.24051	60.850	0.27871
61.032	0.24012	60.850	0.27869
61.033	0.23966	60.850	0.27867
61.033	0.23911	60.850	0.27864
61.033	0.23885	60.850	0.27862
61.033	0.23867	60.851	0.27859
61.033	0.23843	60.851	0.27857
61.033	0.23812	60.851	0.27855
61.033	0.23775	60.851	0.27852
61.034	0.2373	60.851	0.2785
61.034	0.23676	60.851	0.27848
61.034	0.23616	60.852	0.27845
61.034	0.23549	60.852	0.27843
61.034	0.23478	60.852	0.27841
61.034	0.23403	60.852	0.27838

61.034	0.2333	60.852	0.27836
61.035	0.23302	60.852	0.27834
61.035	0.23267	60.852	0.27831
61.035	0.23221	60.853	0.27829
61.035	0.23165	60.853	0.27827
61.035	0.23105	60.853	0.27825
61.035	0.23054	60.853	0.27822
61.036	0.23	60.853	0.2782
61.036	0.22942	60.853	0.27818
61.036	0.22881	60.853	0.27815
61.036	0.22817	60.854	0.27813
61.036	0.22751	60.854	0.27811
61.036	0.22681	60.854	0.27809
61.036	0.22606	60.854	0.27807
61.037	0.22527	60.854	0.27804
61.037	0.22443	60.854	0.27802
61.037	0.22355	60.854	0.278
61.037	0.22265	60.855	0.27798
61.037	0.22173	60.855	0.27796
61.037	0.22086	60.855	0.27794
61.037	0.22001	60.855	0.27792
61.038	0.21913	60.855	0.2779
61.038	0.21831	60.855	0.27787
61.038	0.21754	60.856	0.27785
61.038	0.21674	60.856	0.27783
61.038	0.21591	60.856	0.27781
61.038	0.21504	60.856	0.27779
61.039	0.21414	60.856	0.27777
61.039	0.21319	60.856	0.27775
61.039	0.21221	60.856	0.27773
61.039	0.2112	60.857	0.27771
61.039	0.21017	60.857	0.27769
61.039	0.20912	60.857	0.27767
61.039	0.20806	60.857	0.27765
61.040	0.207	60.857	0.27763
61.040	0.206	60.857	0.2776
61.040	0.205	60.857	0.27758
61.040	0.20398	60.858	0.27756
61.040	0.20294	60.858	0.27754
61.040	0.20187	60.858	0.27752
61.041	0.20079	60.858	0.2775
61.041	0.1997	60.858	0.27748
61.041	0.1986	60.858	0.27746
61.041	0.1975	60.858	0.27744
61.041	0.19649	60.859	0.27742
61.041	0.19554	60.859	0.2774
61.041	0.19462	60.859	0.27738
61.042	0.19368	60.859	0.27736
61.042	0.19273	60.859	0.27734
61.042	0.19178	60.859	0.27731
61.042	0.19081	60.860	0.27729
61.042	0.19013	60.860	0.27727
61.042	0.19046	60.860	0.27725

61.043	0.19082	60.860	0.27723
61.043	0.19108	60.860	0.27721
61.043	0.19115	60.860	0.27719
61.043	0.19106	60.860	0.27717
61.043	0.1909	60.861	0.27715
61.043	0.1907	60.861	0.27713
61.043	0.19049	60.861	0.27711
61.044	0.1903	60.861	0.27709
61.044	0.19011	60.861	0.27707
61.044	0.18992	60.861	0.27705
61.044	0.18971	60.861	0.27703
61.044	0.18947	60.862	0.27701
61.044	0.18919	60.862	0.27699
61.044	0.18885	60.862	0.27697
61.045	0.18847	60.862	0.27695
61.045	0.18806	60.862	0.27693
61.045	0.18761	60.862	0.27691
61.045	0.18758	60.862	0.27689
61.045	0.18762	60.863	0.27687
61.045	0.18763	60.863	0.27685
61.045	0.18765	60.863	0.27683
61.046	0.18769	60.863	0.27682
61.046	0.18777	60.863	0.2768
61.046	0.18782	60.863	0.27678
61.046	0.18782	60.864	0.27676
61.046	0.18779	60.864	0.27675
61.046	0.18772	60.864	0.27673
61.046	0.18762	60.864	0.27671
61.047	0.18748	60.864	0.2767
61.047	0.18731	60.864	0.27668
61.047	0.1872	60.864	0.27666
61.047	0.18744	60.865	0.27665
61.047	0.18823	60.865	0.27663
61.047	0.18969	60.865	0.27662
61.047	0.19107	60.865	0.27661
61.048	0.19237	60.865	0.27659
61.048	0.19363	60.865	0.27658
61.048	0.19493	60.865	0.27657
61.048	0.19615	60.866	0.27655
61.048	0.19741	60.866	0.27654
61.048	0.19854	60.866	0.27653
61.048	0.1995	60.866	0.27652
61.049	0.20031	60.866	0.27651
61.049	0.20097	60.866	0.2765
61.049	0.20155	60.866	0.27649
61.049	0.20213	60.867	0.27648
61.049	0.20258	60.867	0.27647
61.049	0.2029	60.867	0.27646
61.049	0.20308	60.867	0.27646
61.050	0.20315	60.867	0.27645
61.050	0.2031	60.867	0.27644
61.050	0.20294	60.867	0.27643
61.050	0.2027	60.868	0.27643

61.050	0.20238	60.868	0.27642
61.050	0.20198	60.868	0.27642
61.050	0.20153	60.868	0.27641
61.051	0.20103	60.868	0.27641
61.051	0.20046	60.868	0.2764
61.051	0.20027	60.868	0.2764
61.051	0.19991	60.869	0.2764
61.051	0.19934	60.869	0.2764
61.051	0.19858	60.869	0.27639
61.051	0.1978	60.869	0.27639
61.052	0.19709	60.869	0.27639
61.052	0.19629	60.869	0.27639
61.052	0.19542	60.869	0.27639
61.052	0.19459	60.870	0.27639
61.052	0.19501	60.870	0.27639
61.052	0.19541	60.870	0.27639
61.052	0.19578	60.870	0.27639
61.053	0.19612	60.870	0.27639
61.053	0.19642	60.870	0.27639
61.053	0.19669	60.870	0.27639
61.053	0.19692	60.871	0.27639
61.053	0.19711	60.871	0.27639
61.053	0.19726	60.871	0.27639
61.053	0.19737	60.871	0.2764
61.054	0.19744	60.871	0.2764
61.054	0.19747	60.871	0.2764
61.054	0.19746	60.871	0.27641
61.054	0.19741	60.872	0.27641
61.054	0.19732	60.872	0.27641
61.054	0.1972	60.872	0.27642
61.054	0.19704	60.872	0.27642
61.055	0.19684	60.872	0.27642
61.055	0.1966	60.872	0.27643
61.055	0.19633	60.872	0.27643
61.055	0.19602	60.873	0.27644
61.055	0.19567	60.873	0.27644
61.055	0.19529	60.873	0.27645
61.055	0.19487	60.873	0.27645
61.056	0.19441	60.873	0.27646
61.056	0.19393	60.873	0.27646
61.056	0.19341	60.873	0.27647
61.056	0.19286	60.874	0.27647
61.056	0.19227	60.874	0.27648
61.056	0.19166	60.874	0.27648
61.056	0.19102	60.874	0.27649
61.057	0.19035	60.874	0.2765
61.057	0.18967	60.874	0.2765
61.057	0.18895	60.874	0.27651
61.057	0.18838	60.875	0.27651
61.057	0.18784	60.875	0.27652
61.057	0.18726	60.875	0.27652
61.057	0.18664	60.875	0.27653
61.058	0.18611	60.875	0.27654

61.058	0.18583	60.875	0.27654
61.058	0.18552	60.875	0.27655
61.058	0.18519	60.876	0.27655
61.058	0.18483	60.876	0.27656
61.058	0.18444	60.876	0.27657
61.058	0.18401	60.876	0.27657
61.059	0.18355	60.876	0.27658
61.059	0.18305	60.876	0.27658
61.059	0.1825	60.876	0.27659
61.059	0.18192	60.877	0.27659
61.059	0.18129	60.877	0.2766
61.059	0.18063	60.877	0.2766
61.060	0.17991	60.877	0.27661
61.060	0.17916	60.877	0.27662
61.060	0.17847	60.877	0.27662
61.060	0.17801	60.877	0.27663
61.060	0.17783	60.878	0.27663
61.060	0.1778	60.878	0.27664
61.060	0.17774	60.878	0.27664
61.061	0.17766	60.878	0.27665
61.061	0.17755	60.878	0.27665
61.061	0.17743	60.878	0.27666
61.061	0.17729	60.878	0.27666
61.061	0.17712	60.879	0.27667
61.061	0.17694	60.879	0.27668
61.061	0.17674	60.879	0.27668
61.062	0.17652	60.879	0.27669
61.062	0.17628	60.879	0.2767
61.062	0.17602	60.879	0.2767
61.062	0.17574	60.879	0.27671
61.062	0.1755	60.880	0.27672
61.062	0.17525	60.880	0.27673
61.062	0.17497	60.880	0.27673
61.063	0.17466	60.880	0.27674
61.063	0.17433	60.880	0.27675
61.063	0.17397	60.880	0.27676
61.063	0.17359	60.880	0.27677
61.063	0.17319	60.881	0.27678
61.063	0.17277	60.881	0.27678
61.063	0.17266	60.881	0.27679
61.064	0.17257	60.881	0.2768
61.064	0.17249	60.881	0.27681
61.064	0.17241	60.881	0.27682
61.064	0.17233	60.881	0.27683
61.064	0.17225	60.882	0.27684
61.064	0.17218	60.882	0.27685
61.065	0.17211	60.882	0.27687
61.065	0.17204	60.882	0.27688
61.065	0.17197	60.882	0.27689
61.065	0.17191	60.882	0.2769
61.065	0.17225	60.882	0.27691
61.065	0.1729	60.883	0.27692
61.065	0.17353	60.883	0.27694

61.066	0.17414	60.883	0.27695
61.066	0.17472	60.883	0.27696
61.066	0.17527	60.883	0.27698
61.066	0.17579	60.883	0.27699
61.066	0.17628	60.883	0.27701
61.066	0.17675	60.883	0.27702
61.067	0.17719	60.884	0.27704
61.067	0.17761	60.884	0.27705
61.067	0.17801	60.884	0.27707
61.067	0.17841	60.884	0.27708
61.067	0.17879	60.884	0.2771
61.067	0.17913	60.884	0.27711
61.067	0.17945	60.884	0.27713
61.068	0.17975	60.885	0.27715
61.068	0.18003	60.885	0.27717
61.068	0.18029	60.885	0.27718
61.068	0.18055	60.885	0.2772
61.068	0.18076	60.885	0.27722
61.068	0.18097	60.885	0.27724
61.068	0.18116	60.885	0.27726
61.069	0.18135	60.886	0.27727
61.069	0.18154	60.886	0.27729
61.069	0.18172	60.886	0.27731
61.069	0.18191	60.886	0.27733
61.069	0.18246	60.886	0.27735
61.069	0.18259	60.886	0.27737
61.070	0.18309	60.886	0.27739
61.070	0.1847	60.887	0.27741
61.070	0.18487	60.887	0.27743
61.070	0.18399	60.887	0.27745
61.070	0.18324	60.887	0.27747
61.070	0.18385	60.887	0.27749
61.070	0.18338	60.887	0.27751
61.071	0.1834	60.887	0.27753
61.071	0.18494	60.888	0.27755
61.071	0.1852	60.888	0.27757
61.071	0.18451	60.888	0.27759
61.071	0.18358	60.888	0.27761
61.071	0.18446	60.888	0.27763
61.071	0.18572	60.888	0.27765
61.072	0.18599	60.888	0.27768
61.072	0.18641	60.888	0.2777
61.072	0.18587	60.889	0.27772
61.072	0.1848	60.889	0.27774
61.072	0.185	60.889	0.27776
61.072	0.18516	60.889	0.27778
61.073	0.18537	60.889	0.27781
61.073	0.18571	60.889	0.27783
61.073	0.18542	60.889	0.27785
61.073	0.18521	60.890	0.27787
61.073	0.18514	60.890	0.2779
61.073	0.18497	60.890	0.27792
61.073	0.18463	60.890	0.27794

61.074	0.18413	60.890	0.27797
61.074	0.184	60.890	0.27799
61.074	0.18382	60.890	0.27801
61.074	0.18355	60.891	0.27804
61.074	0.18318	60.891	0.27806
61.074	0.18272	60.891	0.27808
61.074	0.18218	60.891	0.27811
61.075	0.18156	60.891	0.27813
61.075	0.18089	60.891	0.27816
61.075	0.18021	60.891	0.27818
61.075	0.18	60.891	0.27821
61.075	0.17978	60.892	0.27823
61.075	0.17956	60.892	0.27825
61.075	0.1794	60.892	0.27828
61.076	0.1803	60.892	0.2783
61.076	0.18019	60.892	0.27833
61.076	0.17931	60.892	0.27835
61.076	0.17908	60.892	0.27838
61.076	0.17812	60.893	0.2784
61.076	0.17785	60.893	0.27843
61.077	0.1776	60.893	0.27845
61.077	0.17736	60.893	0.27848
61.077	0.17716	60.893	0.2785
61.077	0.17727	60.893	0.27853
61.077	0.17735	60.893	0.27856
61.077	0.1774	60.894	0.27858
61.077	0.17743	60.894	0.27861
61.078	0.17743	60.894	0.27863
61.078	0.1774	60.894	0.27866
61.078	0.17733	60.894	0.27868
61.078	0.17724	60.894	0.27871
61.078	0.17714	60.894	0.27873
61.078	0.17707	60.895	0.27876
61.078	0.17704	60.895	0.27878
61.079	0.17697	60.895	0.27881
61.079	0.17688	60.895	0.27883
61.079	0.17676	60.895	0.27886
61.079	0.17661	60.895	0.27889
61.079	0.17644	60.895	0.27891
61.079	0.17625	60.895	0.27894
61.079	0.17603	60.896	0.27896
61.079	0.1758	60.896	0.27899
61.080	0.17557	60.896	0.27901
61.080	0.17533	60.896	0.27904
61.080	0.17511	60.896	0.27906
61.080	0.17489	60.896	0.27909
61.080	0.17468	60.896	0.27911
61.080	0.17447	60.897	0.27914
61.080	0.17427	60.897	0.27916
61.081	0.17407	60.897	0.27919
61.081	0.17387	60.897	0.27921
61.081	0.17366	60.897	0.27924
61.081	0.17345	60.897	0.27926

61.081	0.17324	60.897	0.27929
61.081	0.17303	60.898	0.27931
61.081	0.17282	60.898	0.27934
61.082	0.17266	60.898	0.27936
61.082	0.17251	60.898	0.27939
61.082	0.17237	60.898	0.27941
61.082	0.17222	60.898	0.27944
61.082	0.17207	60.898	0.27946
61.082	0.17191	60.899	0.27949
61.082	0.17177	60.899	0.27951
61.083	0.17165	60.899	0.27953
61.083	0.17154	60.899	0.27956
61.083	0.17143	60.899	0.27958
61.083	0.17133	60.899	0.27961
61.083	0.17122	60.899	0.27963
61.083	0.17115	60.900	0.27966
61.083	0.17111	60.900	0.27968
61.084	0.17108	60.900	0.27971
61.084	0.17105	60.900	0.27973
61.084	0.17101	60.900	0.27976
61.084	0.17098	60.900	0.27978
61.084	0.17094	60.900	0.27981
61.084	0.1709	60.901	0.27983
61.084	0.17087	60.901	0.27986
61.084	0.17096	60.901	0.27988
61.085	0.17106	60.901	0.27991
61.085	0.17121	60.901	0.27993
61.085	0.1713	60.901	0.27996
61.085	0.17134	60.901	0.27998
61.085	0.17131	60.901	0.28
61.085	0.17123	60.902	0.28003
61.085	0.17114	60.902	0.28005
61.086	0.17104	60.902	0.28008
61.086	0.17091	60.902	0.2801
61.086	0.17075	60.902	0.28012
61.086	0.17057	60.902	0.28015
61.086	0.17037	60.902	0.28017
61.086	0.17016	60.903	0.28019
61.086	0.16995	60.903	0.28022
61.087	0.16984	60.903	0.28024
61.087	0.16972	60.903	0.28026
61.087	0.16959	60.903	0.28029
61.087	0.16945	60.903	0.28031
61.087	0.16929	60.903	0.28033
61.087	0.16913	60.904	0.28035
61.087	0.16895	60.904	0.28038
61.088	0.16875	60.904	0.2804
61.088	0.16854	60.904	0.28042
61.088	0.16832	60.904	0.28045
61.088	0.16809	60.904	0.28047
61.088	0.16784	60.904	0.28049
61.088	0.16758	60.905	0.28052
61.088	0.16732	60.905	0.28054

61.089	0.16705	60.905	0.28056
61.089	0.16678	60.905	0.28059
61.089	0.1665	60.905	0.28061
61.089	0.16663	60.905	0.28063
61.089	0.16681	60.905	0.28066
61.089	0.16699	60.906	0.28068
61.089	0.16716	60.906	0.2807
61.089	0.16733	60.906	0.28073
61.090	0.16748	60.906	0.28075
61.090	0.16763	60.906	0.28077
61.090	0.16776	60.906	0.28079
61.090	0.16788	60.906	0.28081
61.090	0.16798	60.907	0.28083
61.090	0.16806	60.907	0.28086
61.090	0.16813	60.907	0.28088
61.091	0.16818	60.907	0.2809
61.091	0.16821	60.907	0.28092
61.091	0.16823	60.907	0.28094
61.091	0.16823	60.908	0.28096
61.091	0.16823	60.908	0.28099
61.091	0.16822	60.908	0.28101
61.091	0.16821	60.908	0.28103
61.092	0.16821	60.908	0.28105
61.092	0.16822	60.908	0.28107
61.092	0.16823	60.908	0.28109
61.092	0.16826	60.909	0.2811
61.092	0.16828	60.909	0.28112
61.092	0.16831	60.909	0.28114
61.092	0.16833	60.909	0.28116
61.093	0.16834	60.909	0.28118
61.093	0.16833	60.909	0.2812
61.093	0.1683	60.909	0.28122
61.093	0.16826	60.910	0.28124
61.093	0.16821	60.910	0.28126
61.093	0.16815	60.910	0.28128
61.093	0.16808	60.910	0.2813
61.094	0.168	60.910	0.28132
61.094	0.16792	60.910	0.28134
61.094	0.16784	60.910	0.28136
61.094	0.16776	60.911	0.28138
61.094	0.16768	60.911	0.2814
61.094	0.1676	60.911	0.28142
61.094	0.16751	60.911	0.28144
61.094	0.16743	60.911	0.28146
61.095	0.16734	60.911	0.28148
61.095	0.16727	60.911	0.28149
61.095	0.1672	60.912	0.28151
61.095	0.16713	60.912	0.28153
61.095	0.16707	60.912	0.28155
61.095	0.16699	60.912	0.28157
61.095	0.16691	60.912	0.28159
61.096	0.16682	60.912	0.28161
61.096	0.16671	60.912	0.28163

61.096	0.16659	60.913	0.28165
61.096	0.16648	60.913	0.28167
61.096	0.16637	60.913	0.28169
61.096	0.1664	60.913	0.28171
61.096	0.16673	60.913	0.28173
61.097	0.16704	60.913	0.28175
61.097	0.16734	60.914	0.28177
61.097	0.16763	60.914	0.28179
61.097	0.1679	60.914	0.28181
61.097	0.16815	60.914	0.28183
61.097	0.16838	60.914	0.28184
61.097	0.16859	60.914	0.28186
61.098	0.16879	60.914	0.28188
61.098	0.16897	60.915	0.2819
61.098	0.16913	60.915	0.28192
61.098	0.16927	60.915	0.28194
61.098	0.1694	60.915	0.28195
61.098	0.16951	60.915	0.28197
61.098	0.1696	60.915	0.28198
61.099	0.16968	60.915	0.282
61.099	0.16975	60.916	0.28202
61.099	0.1698	60.916	0.28203
61.099	0.16997	60.916	0.28205
61.099	0.17023	60.916	0.28206
61.099	0.17047	60.916	0.28208
61.099	0.1707	60.916	0.28209
61.100	0.17093	60.916	0.2821
61.100	0.17114	60.917	0.28212
61.100	0.17133	60.917	0.28213
61.100	0.17152	60.917	0.28214
61.100	0.17169	60.917	0.28215
61.100	0.17183	60.917	0.28216
61.100	0.17197	60.917	0.28218
61.101	0.17208	60.918	0.28219
61.101	0.17217	60.918	0.2822
61.101	0.17225	60.918	0.28221
61.101	0.1723	60.918	0.28222
61.101	0.17233	60.918	0.28223
61.101	0.17234	60.918	0.28225
61.101	0.17234	60.918	0.28227
61.102	0.17231	60.919	0.28228
61.102	0.17226	60.919	0.2823
61.102	0.1722	60.919	0.28233
61.102	0.17212	60.919	0.28235
61.102	0.17204	60.919	0.28237
61.102	0.17195	60.919	0.2824
61.102	0.17186	60.919	0.28242
61.102	0.17175	60.920	0.28245
61.103	0.17163	60.920	0.28247
61.103	0.17149	60.920	0.2825
61.103	0.17133	60.920	0.28252
61.103	0.17115	60.920	0.28255
61.103	0.17097	60.920	0.28257

61.103	0.17082	60.920	0.2826
61.103	0.17067	60.921	0.28262
61.104	0.17051	60.921	0.28265
61.104	0.17034	60.921	0.28267
61.104	0.17023	60.921	0.2827
61.104	0.17012	60.921	0.28273
61.104	0.17	60.921	0.28275
61.104	0.16986	60.921	0.28278
61.104	0.16971	60.922	0.28281
61.105	0.16955	60.922	0.28283
61.105	0.16938	60.922	0.28286
61.105	0.16924	60.922	0.28289
61.105	0.16909	60.922	0.28291
61.105	0.16893	60.922	0.28294
61.105	0.16894	60.922	0.28296
61.105	0.16902	60.922	0.28299
61.106	0.1691	60.923	0.28302
61.106	0.16917	60.923	0.28304
61.106	0.16923	60.923	0.28307
61.106	0.16928	60.923	0.28309
61.106	0.16932	60.923	0.28311
61.106	0.16936	60.923	0.28314
61.106	0.16939	60.923	0.28316
61.107	0.16941	60.924	0.28319
61.107	0.16943	60.924	0.28321
61.107	0.16944	60.924	0.28323
61.107	0.16944	60.924	0.28326
61.107	0.16944	60.924	0.28328
61.107	0.16944	60.924	0.2833
61.107	0.16943	60.924	0.28333
61.108	0.16941	60.925	0.28335
61.108	0.1694	60.925	0.28337
61.108	0.16937	60.925	0.28339
61.108	0.16935	60.925	0.28341
61.108	0.16932	60.925	0.28344
61.108	0.16941	60.925	0.28346
61.108	0.16986	60.925	0.28348
61.109	0.17029	60.926	0.2835
61.109	0.17071	60.926	0.28352
61.109	0.1711	60.926	0.28354
61.109	0.17146	60.926	0.28356
61.109	0.1718	60.926	0.28358
61.109	0.17211	60.926	0.2836
61.109	0.17239	60.926	0.28362
61.110	0.17263	60.926	0.28364
61.110	0.17285	60.927	0.28366
61.110	0.17303	60.927	0.28367
61.110	0.17318	60.927	0.28369
61.110	0.17329	60.927	0.28371
61.110	0.17337	60.927	0.28373
61.110	0.17341	60.927	0.28375
61.111	0.17342	60.927	0.28376
61.111	0.17339	60.928	0.28378

61.111	0.17333	60.928	0.2838
61.111	0.17324	60.928	0.28381
61.111	0.17311	60.928	0.28383
61.111	0.17296	60.928	0.28385
61.111	0.17277	60.928	0.28386
61.112	0.17256	60.928	0.28388
61.112	0.17232	60.929	0.28389
61.112	0.17206	60.929	0.28391
61.112	0.17178	60.929	0.28392
61.112	0.17148	60.929	0.28394
61.112	0.17116	60.929	0.28395
61.112	0.17083	60.929	0.28396
61.113	0.17048	60.929	0.28398
61.113	0.17012	60.930	0.28399
61.113	0.16975	60.930	0.284
61.113	0.16937	60.930	0.28401
61.113	0.16898	60.930	0.28403
61.113	0.16858	60.930	0.28404
61.113	0.16836	60.930	0.28405
61.114	0.16814	60.930	0.28406
61.114	0.16792	60.930	0.28407
61.114	0.1677	60.931	0.28408
61.114	0.16754	60.931	0.28409
61.114	0.16738	60.931	0.2841
61.114	0.16721	60.931	0.28411
61.114	0.16703	60.931	0.28411
61.115	0.16684	60.931	0.28412
61.115	0.16665	60.931	0.28413
61.115	0.16646	60.932	0.28414
61.115	0.16626	60.932	0.28414
61.115	0.16623	60.932	0.28415
61.115	0.16629	60.932	0.28415
61.115	0.16635	60.932	0.28416
61.116	0.16641	60.932	0.28416
61.116	0.16647	60.932	0.28417
61.116	0.16653	60.933	0.28417
61.116	0.1666	60.933	0.28417
61.116	0.16666	60.933	0.28418
61.116	0.16672	60.933	0.28418
61.116	0.16678	60.933	0.28418
61.117	0.16683	60.933	0.28418
61.117	0.16689	60.933	0.28418
61.117	0.16694	60.934	0.28418
61.117	0.16699	60.934	0.28418
61.117	0.16703	60.934	0.28418
61.117	0.16708	60.934	0.28418
61.117	0.16713	60.934	0.28418
61.118	0.16717	60.934	0.28417
61.118	0.16721	60.934	0.28417
61.118	0.16725	60.935	0.28417
61.118	0.16729	60.935	0.28416
61.118	0.16732	60.935	0.28416
61.118	0.16734	60.935	0.28415

61.118	0.16739	60.935	0.28415
61.119	0.16744	60.935	0.28414
61.119	0.16748	60.935	0.28414
61.119	0.16751	60.936	0.28413
61.119	0.16755	60.936	0.28412
61.119	0.16758	60.936	0.28412
61.119	0.1676	60.936	0.28411
61.119	0.16762	60.936	0.2841
61.119	0.16763	60.936	0.28409
61.120	0.16763	60.936	0.28408
61.120	0.16763	60.937	0.28407
61.120	0.16762	60.937	0.28406
61.120	0.1676	60.937	0.28405
61.120	0.16757	60.937	0.28404
61.120	0.16754	60.937	0.28402
61.120	0.1675	60.937	0.28401
61.121	0.16745	60.938	0.284
61.121	0.1674	60.938	0.28398
61.121	0.16734	60.938	0.28397
61.121	0.16727	60.938	0.28395
61.121	0.16719	60.938	0.28393
61.121	0.16711	60.938	0.28392
61.121	0.16701	60.938	0.2839
61.122	0.16691	60.939	0.28388
61.122	0.1668	60.939	0.28386
61.122	0.16669	60.939	0.28384
61.122	0.16656	60.939	0.28382
61.122	0.16643	60.939	0.2838
61.122	0.16629	60.939	0.28378
61.122	0.16615	60.940	0.28375
61.123	0.16599	60.940	0.28373
61.123	0.16584	60.940	0.28371
61.123	0.16568	60.940	0.28368
61.123	0.16551	60.940	0.28366
61.123	0.16533	60.940	0.28363
61.123	0.16516	60.940	0.2836
61.123	0.16497	60.941	0.28358
61.124	0.1651	60.941	0.28355
61.124	0.16506	60.941	0.28352
61.124	0.16476	60.941	0.28349
61.124	0.16521	60.941	0.28346
61.124	0.16608	60.941	0.28343
61.124	0.16638	60.942	0.28339
61.124	0.1662	60.942	0.28336
61.125	0.16623	60.942	0.28333
61.125	0.16658	60.942	0.2833
61.125	0.16719	60.942	0.28326
61.125	0.16736	60.942	0.28323
61.125	0.16738	60.943	0.28319
61.125	0.16757	60.943	0.28316
61.126	0.16771	60.943	0.28312
61.126	0.16783	60.943	0.28308
61.126	0.16792	60.943	0.28304

61.126	0.16799	60.943	0.283
61.126	0.16809	60.944	0.28296
61.126	0.16825	60.944	0.28292
61.126	0.16837	60.944	0.28288
61.127	0.16844	60.944	0.28284
61.127	0.16847	60.944	0.2828
61.127	0.16848	60.944	0.28276
61.127	0.16848	60.944	0.28272
61.127	0.16845	60.945	0.28268
61.127	0.16842	60.945	0.28264
61.127	0.16842	60.945	0.2826
61.128	0.16838	60.945	0.28256
61.128	0.1683	60.945	0.28252
61.128	0.16819	60.945	0.28248
61.128	0.16802	60.946	0.28243
61.128	0.1678	60.946	0.28239
61.128	0.16754	60.946	0.28235
61.128	0.16723	60.946	0.2823
61.129	0.16689	60.946	0.28226
61.129	0.16651	60.946	0.28221
61.129	0.1661	60.947	0.28217
61.129	0.16568	60.947	0.28212
61.129	0.16525	60.947	0.28208
61.129	0.1648	60.947	0.28203
61.129	0.16435	60.947	0.28198
61.130	0.16396	60.947	0.28193
61.130	0.16356	60.948	0.28189
61.130	0.16314	60.948	0.28184
61.130	0.16295	60.948	0.28179
61.130	0.16289	60.948	0.28174
61.130	0.16281	60.948	0.28169
61.130	0.16272	60.948	0.28165
61.131	0.16261	60.949	0.2816
61.131	0.16248	60.949	0.28155
61.131	0.16234	60.949	0.2815
61.131	0.1622	60.949	0.28146
61.131	0.16203	60.949	0.28141
61.131	0.16186	60.949	0.28137
61.131	0.16167	60.950	0.28132
61.132	0.16146	60.950	0.28127
61.132	0.16125	60.950	0.28123
61.132	0.16138	60.950	0.28119
61.132	0.16167	60.950	0.28114
61.132	0.16194	60.950	0.2811
61.132	0.16217	60.951	0.28106
61.133	0.16237	60.951	0.28102
61.133	0.16254	60.951	0.28097
61.133	0.16268	60.951	0.28093
61.133	0.16279	60.951	0.28089
61.133	0.16306	60.951	0.28085
61.133	0.16332	60.952	0.28081
61.133	0.16353	60.952	0.28077
61.134	0.16369	60.952	0.28072

61.134	0.16381	60.952	0.28068
61.134	0.164	60.952	0.28064
61.134	0.16425	60.952	0.2806
61.134	0.16453	60.953	0.28056
61.134	0.16478	60.953	0.28052
61.134	0.16498	60.953	0.28048
61.135	0.16513	60.953	0.28043
61.135	0.16522	60.953	0.28039
61.135	0.16526	60.953	0.28035
61.135	0.16524	60.954	0.28031
61.135	0.16516	60.954	0.28027
61.135	0.16504	60.954	0.28023
61.135	0.16487	60.954	0.28018
61.136	0.16466	60.954	0.28014
61.136	0.16475	60.954	0.2801
61.136	0.16499	60.955	0.28005
61.136	0.16521	60.955	0.28001
61.136	0.1654	60.955	0.27996
61.136	0.16558	60.955	0.27991
61.137	0.16573	60.955	0.27987
61.137	0.16586	60.955	0.27982
61.137	0.16595	60.956	0.27977
61.137	0.16602	60.956	0.27972
61.137	0.16607	60.956	0.27967
61.137	0.16608	60.956	0.27962
61.137	0.16607	60.956	0.27957
61.138	0.16604	60.956	0.27952
61.138	0.16598	60.957	0.27948
61.138	0.16589	60.957	0.27943
61.138	0.16579	60.957	0.27938
61.138	0.16566	60.957	0.27933
61.138	0.16552	60.957	0.27928
61.138	0.16535	60.957	0.27923
61.139	0.16517	60.958	0.27918
61.139	0.16497	60.958	0.27913
61.139	0.16476	60.958	0.27908
61.139	0.16452	60.958	0.27903
61.139	0.16427	60.958	0.27897
61.139	0.16401	60.958	0.27892
61.140	0.16372	60.959	0.27887
61.140	0.16342	60.959	0.27882
61.140	0.1631	60.959	0.27877
61.140	0.16276	60.959	0.27871
61.140	0.1624	60.959	0.27866
61.140	0.16203	60.959	0.2786
61.140	0.16163	60.960	0.27855
61.141	0.16122	60.960	0.27849
61.141	0.1608	60.960	0.27844
61.141	0.16038	60.960	0.27838
61.141	0.15999	60.960	0.27832
61.141	0.15972	60.961	0.27827
61.141	0.15954	60.961	0.27821
61.141	0.15937	60.961	0.27815

61.142	0.15919	60.961	0.27809
61.142	0.159	60.961	0.27804
61.142	0.1588	60.961	0.27798
61.142	0.15893	60.962	0.27793
61.142	0.15944	60.962	0.27787
61.142	0.15994	60.962	0.27782
61.143	0.16044	60.962	0.27777
61.143	0.16094	60.962	0.27773
61.143	0.16144	60.962	0.27769
61.143	0.16194	60.963	0.27764
61.143	0.16244	60.963	0.2776
61.143	0.16297	60.963	0.27757
61.143	0.16349	60.963	0.27753
61.144	0.16399	60.963	0.2775
61.144	0.16445	60.963	0.27746
61.144	0.16489	60.963	0.27743
61.144	0.16528	60.964	0.27739
61.144	0.16565	60.964	0.27736
61.144	0.16597	60.964	0.27733
61.145	0.16626	60.964	0.2773
61.145	0.16652	60.964	0.27727
61.145	0.16674	60.964	0.27724
61.145	0.16693	60.965	0.27721
61.145	0.16709	60.965	0.27718
61.145	0.16722	60.965	0.27715
61.145	0.16732	60.965	0.27712
61.146	0.1674	60.965	0.27709
61.146	0.16745	60.965	0.27706
61.146	0.16748	60.965	0.27703
61.146	0.16749	60.966	0.27699
61.146	0.16747	60.966	0.27696
61.146	0.16744	60.966	0.27693
61.147	0.16738	60.966	0.2769
61.147	0.1673	60.966	0.27687
61.147	0.16721	60.966	0.27684
61.147	0.1671	60.967	0.27681
61.147	0.16697	60.967	0.27678
61.147	0.16683	60.967	0.27675
61.147	0.16668	60.967	0.27672
61.148	0.16651	60.967	0.27669
61.148	0.16634	60.967	0.27666
61.148	0.16616	60.967	0.27663
61.148	0.16616	60.968	0.2766
61.148	0.1662	60.968	0.27657
61.148	0.16622	60.968	0.27654
61.149	0.16624	60.968	0.27651
61.149	0.16624	60.968	0.27648
61.149	0.16624	60.968	0.27645
61.149	0.16621	60.969	0.27642
61.149	0.16618	60.969	0.27639
61.149	0.16613	60.969	0.27635
61.149	0.16606	60.969	0.27632
61.150	0.16598	60.969	0.27629

61.150	0.16588	60.969	0.27626
61.150	0.16575	60.969	0.27623
61.150	0.16561	60.970	0.27619
61.150	0.16545	60.970	0.27616
61.150	0.16526	60.970	0.27613
61.151	0.16506	60.970	0.2761
61.151	0.16483	60.970	0.27606
61.151	0.16458	60.970	0.27603
61.151	0.1643	60.970	0.276
61.151	0.16401	60.971	0.27597
61.151	0.16369	60.971	0.27593
61.152	0.16335	60.971	0.2759
61.152	0.16299	60.971	0.27587
61.152	0.16321	60.971	0.27584
61.152	0.16371	60.971	0.2758
61.152	0.16423	60.972	0.27577
61.152	0.16471	60.972	0.27574
61.152	0.16514	60.972	0.27571
61.153	0.16552	60.972	0.27568
61.153	0.16585	60.972	0.27564
61.153	0.16614	60.972	0.27561
61.153	0.16638	60.972	0.27558
61.153	0.16658	60.973	0.27555
61.153	0.16674	60.973	0.27551
61.154	0.16685	60.973	0.27548
61.154	0.16693	60.973	0.27545
61.154	0.16697	60.973	0.27542
61.154	0.16697	60.973	0.27538
61.154	0.16693	60.973	0.27535
61.154	0.16685	60.974	0.27532
61.154	0.16675	60.974	0.27529
61.155	0.16661	60.974	0.27526
61.155	0.16645	60.974	0.27522
61.155	0.16626	60.974	0.27519
61.155	0.16627	60.974	0.27516
61.155	0.16651	60.975	0.27513
61.155	0.16674	60.975	0.27509
61.156	0.16695	60.975	0.27506
61.156	0.16715	60.975	0.27503
61.156	0.16734	60.975	0.27499
61.156	0.16752	60.975	0.27496
61.156	0.16768	60.975	0.27493
61.156	0.16782	60.976	0.2749
61.156	0.16795	60.976	0.27486
61.157	0.16805	60.976	0.27483
61.157	0.16814	60.976	0.27479
61.157	0.1682	60.976	0.27476
61.157	0.16825	60.976	0.27473
61.157	0.16828	60.977	0.27469
61.157	0.16828	60.977	0.27466
61.158	0.16826	60.977	0.27462
61.158	0.16823	60.977	0.27459
61.158	0.16817	60.977	0.27456

61.158	0.1681	60.977	0.27452
61.158	0.168	60.977	0.27449
61.158	0.16788	60.978	0.27445
61.158	0.16775	60.978	0.27442
61.159	0.16759	60.978	0.27438
61.159	0.16742	60.978	0.27435
61.159	0.16722	60.978	0.27431
61.159	0.167	60.978	0.27428
61.159	0.16677	60.979	0.27424
61.159	0.16651	60.979	0.27421
61.159	0.16624	60.979	0.27417
61.160	0.16594	60.979	0.27414
61.160	0.16562	60.979	0.2741
61.160	0.16529	60.979	0.27407
61.160	0.16494	60.979	0.27403
61.160	0.16457	60.980	0.274
61.160	0.16419	60.980	0.27396
61.161	0.16379	60.980	0.27392
61.161	0.16337	60.980	0.27389
61.161	0.16295	60.980	0.27385
61.161	0.16251	60.980	0.27381
61.161	0.16206	60.981	0.27378
61.161	0.1616	60.981	0.27374
61.161	0.16114	60.981	0.2737
61.162	0.16067	60.981	0.27366
61.162	0.16019	60.981	0.27363
61.162	0.15988	60.981	0.27359
61.162	0.15994	60.981	0.27355
61.162	0.16001	60.982	0.27352
61.162	0.16007	60.982	0.27348
61.163	0.16013	60.982	0.27344
61.163	0.16019	60.982	0.2734
61.163	0.1604	60.982	0.27337
61.163	0.16072	60.982	0.27333
61.163	0.16104	60.983	0.27329
61.163	0.16137	60.983	0.27325
61.163	0.16168	60.983	0.27322
61.164	0.16197	60.983	0.27318
61.164	0.16224	60.983	0.27314
61.164	0.16249	60.983	0.2731
61.164	0.16272	60.984	0.27307
61.164	0.16292	60.984	0.27303
61.164	0.1631	60.984	0.27299
61.164	0.16326	60.984	0.27295
61.165	0.16341	60.984	0.27292
61.165	0.16353	60.984	0.27288
61.165	0.16364	60.984	0.27284
61.165	0.16373	60.985	0.2728
61.165	0.1638	60.985	0.27276
61.165	0.16387	60.985	0.27273
61.166	0.16393	60.985	0.27269
61.166	0.164	60.985	0.27265
61.166	0.16406	60.985	0.27262

61.166	0.1641	60.986	0.27258
61.166	0.16413	60.986	0.27254
61.166	0.16414	60.986	0.2725
61.166	0.16413	60.986	0.27247
61.167	0.1641	60.986	0.27243
61.167	0.16406	60.986	0.27239
61.167	0.16401	60.987	0.27235
61.167	0.16394	60.987	0.27232
61.167	0.16394	60.987	0.27228
61.167	0.16408	60.987	0.27233
61.167	0.16422	60.987	0.27238
61.168	0.16435	60.987	0.27243
61.168	0.16447	60.988	0.27248
61.168	0.16458	60.988	0.27253
61.168	0.16468	60.988	0.27257
61.168	0.16478	60.988	0.27262
61.168	0.16486	60.988	0.27266
61.169	0.16493	60.988	0.27271
61.169	0.165	60.988	0.27275
61.169	0.16505	60.989	0.2728
61.169	0.1651	60.989	0.27284
61.169	0.16513	60.989	0.27288
61.169	0.16515	60.989	0.27293
61.169	0.16517	60.989	0.27297
61.170	0.16517	60.989	0.27301
61.170	0.16516	60.990	0.27305
61.170	0.16514	60.990	0.27309
61.170	0.16511	60.990	0.27313
61.170	0.16507	60.990	0.27317
61.170	0.16501	60.990	0.27321
61.171	0.16494	60.990	0.27325
61.171	0.16486	60.991	0.27328
61.171	0.16476	60.991	0.27332
61.171	0.16465	60.991	0.27336
61.171	0.16452	60.991	0.27339
61.171	0.16438	60.991	0.27343
61.171	0.16422	60.991	0.27347
61.172	0.16406	60.992	0.2735
61.172	0.16387	60.992	0.27353
61.172	0.16368	60.992	0.27357
61.172	0.16347	60.992	0.2736
61.172	0.16325	60.992	0.27363
61.172	0.16301	60.993	0.27366
61.172	0.16277	60.993	0.27369
61.173	0.16251	60.993	0.27372
61.173	0.16224	60.993	0.27375
61.173	0.16196	60.993	0.27378
61.173	0.16167	60.993	0.2738
61.173	0.16136	60.994	0.27383
61.173	0.16104	60.994	0.27386
61.174	0.16081	60.994	0.27388
61.174	0.16113	60.994	0.27391
61.174	0.16145	60.994	0.27393

61.174	0.16176	60.994	0.27395
61.174	0.16208	60.995	0.27398
61.174	0.16239	60.995	0.274
61.174	0.16269	60.995	0.27402
61.175	0.16298	60.995	0.27404
61.175	0.16327	60.995	0.27406
61.175	0.16355	60.995	0.27408
61.175	0.16381	60.996	0.2741
61.175	0.16407	60.996	0.27412
61.175	0.16432	60.996	0.27413
61.175	0.16456	60.996	0.27415
61.176	0.16479	60.996	0.27417
61.176	0.16501	60.996	0.27418
61.176	0.16521	60.997	0.2742
61.176	0.16541	60.997	0.27421
61.176	0.16559	60.997	0.27423
61.176	0.16577	60.997	0.27424
61.177	0.16593	60.997	0.27425
61.177	0.16608	60.997	0.27426
61.177	0.16621	60.997	0.27427
61.177	0.16634	60.998	0.27428
61.177	0.16645	60.998	0.27429
61.177	0.16655	60.998	0.2743
61.177	0.16664	60.998	0.27431
61.178	0.16671	60.998	0.27432
61.178	0.16677	60.998	0.27433
61.178	0.16682	60.999	0.27434
61.178	0.16685	60.999	0.27434
61.178	0.16687	60.999	0.27435
61.178	0.1669	60.999	0.27435
61.178	0.16692	60.999	0.27436
61.179	0.16693	60.999	0.27436
61.179	0.16692	61.000	0.27436
61.179	0.16691	61.000	0.27437
61.179	0.16688	61.000	0.27437
61.179	0.16684	61.000	0.27437
61.179	0.16679	61.000	0.27437
61.180	0.16673	61.000	0.15376
61.180	0.16665	61.001	0.1536
61.180	0.16656	61.001	0.15343
61.180	0.16646	61.001	0.15325
61.180	0.16635	61.001	0.15307
61.180	0.16623	61.001	0.15288
61.180	0.16609	61.001	0.15267
61.181	0.16595	61.002	0.15246
61.181	0.1658	61.002	0.15223
61.181	0.16563	61.002	0.152
61.181	0.16546	61.002	0.15175
61.181	0.16528	61.002	0.1515
61.181	0.16509	61.002	0.15124
61.182	0.1649	61.002	0.15099
61.182	0.16469	61.003	0.15072
61.182	0.16448	61.003	0.15045

61.182	0.16426	61.003	0.15018
61.182	0.16403	61.003	0.14989
61.182	0.1638	61.003	0.1496
61.182	0.16356	61.003	0.1493
61.183	0.16339	61.004	0.14898
61.183	0.1632	61.004	0.14866
61.183	0.16301	61.004	0.14834
61.183	0.16281	61.004	0.148
61.183	0.16261	61.004	0.14767
61.183	0.16239	61.004	0.14733
61.184	0.16217	61.004	0.14698
61.184	0.16193	61.005	0.14663
61.184	0.16169	61.005	0.14627
61.184	0.16143	61.005	0.14604
61.184	0.16116	61.005	0.14552
61.184	0.16089	61.005	0.14514
61.184	0.1606	61.005	0.14475
61.185	0.16031	61.006	0.14437
61.185	0.16001	61.006	0.14399
61.185	0.1597	61.006	0.1436
61.185	0.15939	61.006	0.14428
61.185	0.15907	61.006	0.14376
61.185	0.15874	61.006	0.14447
61.185	0.15841	61.007	0.14888
61.186	0.15807	61.007	0.15034
61.186	0.15811	61.007	0.14453
61.186	0.15817	61.007	0.14472
61.186	0.15823	61.007	0.14493
61.186	0.15829	61.007	0.15119
61.186	0.15834	61.007	0.14533
61.186	0.15837	61.008	0.15033
61.187	0.15839	61.008	0.14942
61.187	0.15841	61.008	0.1459
61.187	0.15842	61.008	0.14608
61.187	0.15843	61.008	0.14626
61.187	0.15844	61.008	0.14643
61.187	0.15846	61.009	0.1466
61.187	0.15847	61.009	0.14897
61.188	0.15849	61.009	0.14693
61.188	0.15849	61.009	0.14839
61.188	0.15849	61.009	0.14725
61.188	0.15848	61.009	0.1474
61.188	0.15846	61.009	0.14755
61.188	0.15845	61.010	0.14769
61.188	0.15843	61.010	0.14783
61.189	0.15841	61.010	0.14797
61.189	0.1584	61.010	0.1481
61.189	0.15839	61.010	0.14823
61.189	0.15839	61.010	0.14836
61.189	0.15838	61.011	0.14848
61.189	0.15836	61.011	0.1486
61.189	0.15832	61.011	0.14872
61.189	0.15825	61.011	0.14883

61.190	0.15817	61.011	0.14894
61.190	0.15808	61.011	0.14905
61.190	0.15799	61.011	0.14915
61.190	0.1579	61.012	0.14925
61.190	0.15781	61.012	0.14935
61.190	0.15772	61.012	0.14945
61.190	0.15763	61.012	0.14954
61.191	0.15753	61.012	0.14962
61.191	0.15744	61.012	0.14971
61.191	0.15736	61.013	0.14979
61.191	0.15733	61.013	0.14987
61.191	0.15735	61.013	0.14995
61.191	0.1574	61.013	0.15002
61.191	0.15749	61.013	0.1501
61.192	0.1575	61.013	0.15016
61.192	0.15744	61.013	0.15023
61.192	0.15742	61.014	0.1503
61.192	0.15743	61.014	0.15036
61.192	0.15743	61.014	0.1505
61.192	0.15741	61.014	0.15076
61.192	0.15739	61.014	0.15103
61.193	0.15736	61.014	0.15129
61.193	0.15733	61.014	0.15155
61.193	0.15729	61.015	0.1518
61.193	0.15724	61.015	0.15213
61.193	0.15719	61.015	0.15266
61.193	0.15714	61.015	0.15318
61.193	0.15712	61.015	0.1537
61.194	0.1571	61.015	0.15422
61.194	0.15708	61.016	0.15474
61.194	0.15707	61.016	0.15526
61.194	0.15705	61.016	0.15578
61.194	0.15703	61.016	0.15629
61.194	0.15701	61.016	0.15681
61.194	0.15698	61.016	0.15732
61.195	0.15694	61.016	0.15783
61.195	0.1569	61.017	0.15834
61.195	0.15689	61.017	0.15885
61.195	0.1569	61.017	0.15935
61.195	0.15691	61.017	0.15986
61.195	0.1569	61.017	0.16036
61.195	0.15689	61.017	0.16086
61.195	0.15687	61.017	0.16135
61.196	0.15685	61.018	0.16185
61.196	0.15681	61.018	0.16234
61.196	0.15677	61.018	0.16283
61.196	0.15673	61.018	0.16332
61.196	0.15667	61.018	0.1638
61.196	0.15671	61.018	0.16428
61.196	0.15704	61.019	0.16476
61.197	0.15737	61.019	0.16523
61.197	0.16005	61.019	0.16571
61.197	0.16008	61.019	0.16618

61.197	0.15831	61.019	0.16664
61.197	0.15861	61.019	0.16711
61.197	0.15891	61.019	0.16757
61.197	0.1592	61.020	0.16802
61.198	0.15948	61.020	0.16848
61.198	0.15976	61.020	0.16893
61.198	0.16003	61.020	0.16938
61.198	0.1603	61.020	0.16982
61.198	0.16055	61.020	0.17028
61.198	0.16083	61.021	0.17073
61.198	0.16118	61.021	0.17118
61.199	0.16152	61.021	0.17163
61.199	0.16189	61.021	0.17208
61.199	0.16222	61.021	0.17252
61.199	0.16252	61.021	0.17295
61.199	0.1628	61.021	0.17338
61.199	0.16311	61.022	0.17381
61.199	0.16342	61.022	0.17424
61.200	0.16371	61.022	0.17466
61.200	0.16401	61.022	0.17508
61.200	0.16432	61.022	0.17549
61.200	0.16462	61.022	0.1759
61.200	0.1649	61.022	0.1763
61.200	0.16517	61.023	0.1767
61.200	0.16543	61.023	0.1771
61.200	0.16568	61.023	0.17749
61.201	0.16591	61.023	0.17788
61.201	0.16613	61.023	0.17827
61.201	0.16633	61.023	0.17865
61.201	0.16653	61.024	0.17902
61.201	0.1667	61.024	0.17939
61.201	0.16687	61.024	0.17976
61.201	0.16703	61.024	0.18013
61.202	0.16718	61.024	0.18048
61.202	0.16731	61.024	0.18084
61.202	0.16743	61.024	0.18119
61.202	0.16754	61.025	0.18154
61.202	0.16764	61.025	0.18188
61.202	0.1682	61.025	0.18223
61.202	0.16877	61.025	0.18259
61.203	0.16786	61.025	0.18295
61.203	0.16791	61.025	0.1833
61.203	0.16794	61.026	0.18365
61.203	0.16796	61.026	0.18399
61.203	0.168	61.026	0.18434
61.203	0.16802	61.026	0.18468
61.203	0.16804	61.026	0.18501
61.204	0.16806	61.026	0.18535
61.204	0.16808	61.026	0.18568
61.204	0.16808	61.027	0.18601
61.204	0.16807	61.027	0.18633
61.204	0.16805	61.027	0.18666
61.204	0.16808	61.027	0.18698

61.204	0.1681	61.027	0.18729
61.205	0.16807	61.027	0.18761
61.205	0.16801	61.027	0.18791
61.205	0.1679	61.028	0.18822
61.205	0.16776	61.028	0.18852
61.205	0.1676	61.028	0.18882
61.205	0.16742	61.028	0.18911
61.205	0.16727	61.028	0.18941
61.205	0.16712	61.028	0.18969
61.206	0.16699	61.029	0.18997
61.206	0.16685	61.029	0.19025
61.206	0.1667	61.029	0.19053
61.206	0.16654	61.029	0.1908
61.206	0.16638	61.029	0.19106
61.206	0.1662	61.029	0.19132
61.206	0.16602	61.029	0.19158
61.207	0.16584	61.030	0.19183
61.207	0.16564	61.030	0.19207
61.207	0.16544	61.030	0.19231
61.207	0.16523	61.030	0.19255
61.207	0.16501	61.030	0.19278
61.207	0.16479	61.030	0.193
61.207	0.16456	61.031	0.19322
61.208	0.16432	61.031	0.19344
61.208	0.16409	61.031	0.19365
61.208	0.16384	61.031	0.19385
61.208	0.1636	61.031	0.19405
61.208	0.16335	61.031	0.19425
61.208	0.16316	61.031	0.19446
61.208	0.16303	61.032	0.19466
61.209	0.16288	61.032	0.19485
61.209	0.16274	61.032	0.19504
61.209	0.16258	61.032	0.19522
61.209	0.16241	61.032	0.1954
61.209	0.16223	61.032	0.19557
61.209	0.16204	61.033	0.19574
61.209	0.16183	61.033	0.1959
61.210	0.16162	61.033	0.19605
61.210	0.16139	61.033	0.1962
61.210	0.16115	61.033	0.19634
61.210	0.1609	61.033	0.19648
61.210	0.16064	61.033	0.19661
61.210	0.16038	61.034	0.19673
61.210	0.16011	61.034	0.19684
61.210	0.15983	61.034	0.19695
61.211	0.15955	61.034	0.19706
61.211	0.15926	61.034	0.19715
61.211	0.15898	61.034	0.19724
61.211	0.15869	61.035	0.19733
61.211	0.15839	61.035	0.1974
61.211	0.15809	61.035	0.19747
61.211	0.15779	61.035	0.19753
61.212	0.15748	61.035	0.19759

61.212	0.15717	61.035	0.19764
61.212	0.15685	61.035	0.19768
61.212	0.15652	61.036	0.19772
61.212	0.15618	61.036	0.19775
61.212	0.15583	61.036	0.19777
61.212	0.15548	61.036	0.19779
61.213	0.15511	61.036	0.19787
61.213	0.15473	61.036	0.19794
61.213	0.15434	61.037	0.19801
61.213	0.15438	61.037	0.19808
61.213	0.15476	61.037	0.19814
61.213	0.15514	61.037	0.19819
61.213	0.1555	61.037	0.19824
61.214	0.15587	61.037	0.19828
61.214	0.15622	61.037	0.19832
61.214	0.15657	61.038	0.19835
61.214	0.15691	61.038	0.19837
61.214	0.15724	61.038	0.19839
61.214	0.15756	61.038	0.1984
61.214	0.15788	61.038	0.19841
61.214	0.15819	61.038	0.19841
61.215	0.15849	61.039	0.1984
61.215	0.15878	61.039	0.19839
61.215	0.15906	61.039	0.19837
61.215	0.15933	61.039	0.19834
61.215	0.1596	61.039	0.1983
61.215	0.15986	61.039	0.19826
61.215	0.16011	61.040	0.19821
61.216	0.16035	61.040	0.19816
61.216	0.16058	61.040	0.1981
61.216	0.1608	61.040	0.19803
61.216	0.16102	61.040	0.19795
61.216	0.16123	61.040	0.19787
61.216	0.16143	61.040	0.19778
61.216	0.16162	61.041	0.19769
61.217	0.1618	61.041	0.19759
61.217	0.16198	61.041	0.19748
61.217	0.16215	61.041	0.19737
61.217	0.16231	61.041	0.19727
61.217	0.16247	61.041	0.19716
61.217	0.16262	61.042	0.19705
61.217	0.16276	61.042	0.19693
61.218	0.16289	61.042	0.1968
61.218	0.16302	61.042	0.19667
61.218	0.16315	61.042	0.19653
61.218	0.16327	61.042	0.19639
61.218	0.16338	61.042	0.19624
61.218	0.16348	61.043	0.19608
61.218	0.16358	61.043	0.19592
61.219	0.16368	61.043	0.19575
61.219	0.16377	61.043	0.19557
61.219	0.16386	61.043	0.19539
61.219	0.16394	61.043	0.19521

61.219	0.16401	61.044	0.19501
61.219	0.16408	61.044	0.19482
61.219	0.16415	61.044	0.19461
61.219	0.16421	61.044	0.1944
61.220	0.16426	61.044	0.19419
61.220	0.16431	61.044	0.19397
61.220	0.16436	61.045	0.19374
61.220	0.1644	61.045	0.19351
61.220	0.16444	61.045	0.19328
61.220	0.16447	61.045	0.19304
61.220	0.1645	61.045	0.1928
61.221	0.16453	61.045	0.19255
61.221	0.16455	61.045	0.19229
61.221	0.16457	61.046	0.19203
61.221	0.16458	61.046	0.19184
61.221	0.16459	61.046	0.19166
61.221	0.1646	61.046	0.19148
61.221	0.1646	61.046	0.1913
61.222	0.1646	61.046	0.19111
61.222	0.16459	61.047	0.19092
61.222	0.16458	61.047	0.19072
61.222	0.16457	61.047	0.19052
61.222	0.16455	61.047	0.19031
61.222	0.16452	61.047	0.1901
61.222	0.16449	61.047	0.18989
61.223	0.16445	61.048	0.18967
61.223	0.16441	61.048	0.18944
61.223	0.16437	61.048	0.18921
61.223	0.16432	61.048	0.18898
61.223	0.16426	61.048	0.18874
61.223	0.1642	61.048	0.18849
61.223	0.16413	61.049	0.18825
61.223	0.16406	61.049	0.188
61.224	0.16398	61.049	0.18774
61.224	0.1639	61.049	0.18748
61.224	0.16381	61.049	0.18721
61.224	0.16371	61.049	0.18695
61.224	0.16361	61.049	0.18667
61.224	0.1635	61.050	0.1864
61.224	0.16338	61.050	0.18612
61.225	0.16326	61.050	0.18585
61.225	0.16313	61.050	0.18557
61.225	0.16299	61.050	0.1853
61.225	0.16285	61.050	0.18502
61.225	0.1627	61.051	0.18474
61.225	0.16254	61.051	0.18445
61.225	0.16237	61.051	0.18416
61.226	0.1622	61.051	0.18386
61.226	0.16202	61.051	0.18356
61.226	0.16183	61.051	0.18326
61.226	0.16164	61.052	0.18295
61.226	0.16155	61.052	0.18263
61.226	0.16211	61.052	0.18232

61.226	0.16267	61.052	0.182
61.227	0.16321	61.052	0.18168
61.227	0.16376	61.052	0.18135
61.227	0.1643	61.052	0.18102
61.227	0.16483	61.053	0.18069
61.227	0.16537	61.053	0.18035
61.227	0.16589	61.053	0.18001
61.227	0.16642	61.053	0.17967
61.228	0.16694	61.053	0.17932
61.228	0.16748	61.053	0.17897
61.228	0.16803	61.054	0.17862
61.228	0.16861	61.054	0.17826
61.228	0.16919	61.054	0.17791
61.228	0.16978	61.054	0.17755
61.228	0.17036	61.054	0.17719
61.228	0.17095	61.054	0.17682
61.229	0.17154	61.055	0.17646
61.229	0.17212	61.055	0.17609
61.229	0.1727	61.055	0.17573
61.229	0.17327	61.055	0.17536
61.229	0.17384	61.055	0.17499
61.229	0.17441	61.055	0.17461
61.229	0.17497	61.055	0.17424
61.230	0.17552	61.056	0.17387
61.230	0.17606	61.056	0.17349
61.230	0.1766	61.056	0.17312
61.230	0.17713	61.056	0.17275
61.230	0.17765	61.056	0.17237
61.230	0.17817	61.056	0.172
61.230	0.17868	61.057	0.17171
61.231	0.17918	61.057	0.17191
61.231	0.17968	61.057	0.175
61.231	0.18017	61.057	0.17435
61.231	0.18066	61.057	0.17249
61.231	0.18113	61.057	0.17268
61.231	0.18161	61.057	0.17287
61.231	0.18208	61.058	0.17306
61.232	0.18256	61.058	0.17325
61.232	0.18308	61.058	0.17343
61.232	0.1836	61.058	0.17411
61.232	0.18412	61.058	0.1738
61.232	0.18463	61.058	0.17399
61.232	0.18512	61.059	0.17417
61.232	0.18562	61.059	0.17443
61.233	0.1861	61.059	0.17452
61.233	0.18657	61.059	0.1747
61.233	0.18703	61.059	0.17486
61.233	0.18748	61.059	0.17503
61.233	0.18792	61.059	0.17519
61.233	0.18835	61.060	0.17534
61.233	0.18877	61.060	0.17549
61.233	0.18917	61.060	0.17563
61.234	0.18957	61.060	0.17577

61.234	0.18995	61.060	0.1759
61.234	0.19032	61.060	0.17603
61.234	0.19068	61.060	0.17618
61.234	0.19109	61.061	0.17633
61.234	0.19153	61.061	0.17648
61.234	0.19197	61.061	0.17661
61.235	0.1924	61.061	0.17674
61.235	0.19282	61.061	0.17686
61.235	0.19324	61.061	0.17698
61.235	0.19365	61.062	0.17708
61.235	0.19405	61.062	0.17718
61.235	0.19444	61.062	0.17727
61.235	0.19482	61.062	0.17736
61.236	0.1952	61.062	0.17743
61.236	0.19557	61.062	0.1775
61.236	0.19593	61.062	0.17756
61.236	0.19628	61.063	0.17762
61.236	0.19662	61.063	0.17767
61.236	0.19696	61.063	0.17771
61.236	0.19728	61.063	0.17776
61.237	0.1976	61.063	0.1778
61.237	0.19791	61.063	0.17785
61.237	0.19821	61.064	0.17789
61.237	0.19851	61.064	0.17792
61.237	0.1988	61.064	0.17796
61.237	0.19908	61.064	0.18009
61.237	0.19935	61.064	0.17884
61.237	0.19962	61.064	0.17801
61.238	0.19987	61.064	0.17802
61.238	0.20012	61.065	0.17802
61.238	0.20037	61.065	0.17802
61.238	0.2006	61.065	0.17801
61.238	0.20083	61.065	0.17799
61.238	0.20106	61.065	0.17797
61.238	0.20127	61.065	0.17794
61.239	0.20148	61.065	0.17791
61.239	0.20169	61.066	0.17788
61.239	0.20188	61.066	0.17784
61.239	0.20207	61.066	0.1778
61.239	0.20226	61.066	0.17776
61.239	0.20244	61.066	0.1777
61.239	0.20261	61.066	0.17765
61.240	0.20277	61.067	0.17758
61.240	0.20293	61.067	0.17751
61.240	0.20308	61.067	0.17743
61.240	0.20322	61.067	0.17734
61.240	0.20336	61.067	0.17725
61.240	0.20348	61.067	0.17716
61.240	0.20361	61.067	0.17705
61.241	0.20372	61.068	0.17695
61.241	0.20382	61.068	0.17719
61.241	0.20392	61.068	0.17722
61.241	0.20401	61.068	0.17659

61.241	0.20409	61.068	0.17646
61.241	0.20417	61.068	0.17846
61.241	0.20423	61.068	0.17715
61.242	0.20428	61.069	0.17604
61.242	0.20433	61.069	0.17589
61.242	0.20437	61.069	0.17573
61.242	0.20439	61.069	0.17778
61.242	0.20441	61.069	0.17593
61.242	0.20442	61.069	0.17521
61.242	0.20441	61.069	0.17503
61.242	0.2044	61.070	0.17484
61.243	0.20438	61.070	0.17464
61.243	0.20435	61.070	0.17443
61.243	0.2043	61.070	0.17422
61.243	0.20425	61.070	0.174
61.243	0.20419	61.070	0.17452
61.243	0.20412	61.071	0.17358
61.243	0.20403	61.071	0.1733
61.244	0.20394	61.071	0.17305
61.244	0.20384	61.071	0.1728
61.244	0.20372	61.071	0.17254
61.244	0.2036	61.071	0.17227
61.244	0.20347	61.071	0.172
61.244	0.20333	61.072	0.17172
61.244	0.20318	61.072	0.17143
61.245	0.20302	61.072	0.17115
61.245	0.20285	61.072	0.17086
61.245	0.20268	61.072	0.17059
61.245	0.2025	61.072	0.17031
61.245	0.2023	61.072	0.17002
61.245	0.20211	61.073	0.16974
61.245	0.20192	61.073	0.16944
61.246	0.20171	61.073	0.16915
61.246	0.20151	61.073	0.16884
61.246	0.20129	61.073	0.16854
61.246	0.20113	61.073	0.16823
61.246	0.20109	61.073	0.16791
61.246	0.20104	61.074	0.1676
61.246	0.20098	61.074	0.16729
61.247	0.20092	61.074	0.16698
61.247	0.20085	61.074	0.16667
61.247	0.20077	61.074	0.16635
61.247	0.20069	61.074	0.16603
61.247	0.2006	61.075	0.16571
61.247	0.2005	61.075	0.16651
61.247	0.20039	61.075	0.16536
61.248	0.20028	61.075	0.16473
61.248	0.20017	61.075	0.16439
61.248	0.20004	61.075	0.16405
61.248	0.19992	61.075	0.16371
61.248	0.19978	61.076	0.16336
61.248	0.19964	61.076	0.16302
61.248	0.1995	61.076	0.16266

61.248	0.19935	61.076	0.16231
61.249	0.1992	61.076	0.16195
61.249	0.19904	61.076	0.16159
61.249	0.19888	61.076	0.16123
61.249	0.19872	61.077	0.16086
61.249	0.19855	61.077	0.16049
61.249	0.19839	61.077	0.16012
61.249	0.19822	61.077	0.16241
61.250	0.19804	61.077	0.16261
61.250	0.19787	61.077	0.16135
61.250	0.19768	61.077	0.16382
61.250	0.19749	61.078	0.16259
61.250	0.1973	61.078	0.15994
61.250	0.1971	61.078	0.16032
61.250	0.1969	61.078	0.15807
61.251	0.19669	61.078	0.16182
61.251	0.19648	61.078	0.16012
61.251	0.19632	61.079	0.15716
61.251	0.1962	61.079	0.1563
61.251	0.19607	61.079	0.15527
61.251	0.19594	61.079	0.15451
61.251	0.1958	61.079	0.15409
61.252	0.19565	61.079	0.15367
61.252	0.1955	61.079	0.15325
61.252	0.19534	61.080	0.15285
61.252	0.19518	61.080	0.15242
61.252	0.19501	61.080	0.152
61.252	0.19483	61.080	0.1562
61.252	0.19465	61.080	0.15481
61.253	0.19448	61.080	0.1555
61.253	0.19431	61.080	0.15421
61.253	0.19412	61.081	0.15379
61.253	0.19394	61.081	0.15269
61.253	0.19374	61.081	0.15028
61.253	0.19355	61.081	0.14951
61.253	0.19334	61.081	0.15078
61.254	0.19313	61.081	0.14978
61.254	0.19291	61.081	0.14856
61.254	0.19269	61.082	0.15044
61.254	0.19246	61.082	0.15123
61.254	0.19223	61.082	0.15177
61.254	0.19199	61.082	0.15076
61.254	0.19175	61.082	0.15268
61.255	0.1915	61.082	0.15197
61.255	0.19124	61.083	0.15045
61.255	0.19098	61.083	0.14892
61.255	0.19071	61.083	0.15011
61.255	0.19044	61.083	0.1515
61.255	0.19016	61.083	0.15129
61.255	0.18988	61.083	0.14982
61.256	0.1896	61.083	0.15026
61.256	0.1893	61.084	0.14889
61.256	0.18901	61.084	0.14532

61.256	0.18871	61.084	0.14922
61.256	0.18841	61.084	0.14884
61.256	0.1881	61.084	0.1477
61.256	0.18779	61.084	0.14531
61.256	0.18747	61.085	0.14208
61.257	0.18715	61.085	0.14266
61.257	0.18683	61.085	0.14644
61.257	0.1865	61.085	0.14391
61.257	0.18617	61.085	0.1429
61.257	0.18584	61.085	0.14684
61.257	0.18551	61.085	0.14549
61.257	0.18517	61.086	0.1418
61.258	0.18483	61.086	0.14442
61.258	0.18448	61.086	0.14331
61.258	0.18414	61.086	0.14145
61.258	0.18379	61.086	0.14037
61.258	0.18343	61.086	0.13843
61.258	0.18308	61.086	0.13931
61.258	0.18272	61.087	0.13834
61.259	0.18237	61.087	0.14116
61.259	0.18201	61.087	0.13966
61.259	0.18164	61.087	0.14158
61.259	0.18128	61.087	0.14092
61.259	0.18092	61.087	0.1429
61.259	0.18056	61.088	0.14213
61.259	0.18023	61.088	0.13958
61.260	0.17991	61.088	0.13818
61.260	0.17961	61.088	0.13828
61.260	0.17931	61.088	0.13688
61.260	0.17901	61.088	0.13762
61.260	0.17871	61.088	0.13918
61.260	0.17841	61.089	0.13786
61.260	0.1781	61.089	0.13612
61.261	0.17779	61.089	0.13794
61.261	0.17748	61.089	0.13743
61.261	0.17717	61.089	0.13722
61.261	0.17685	61.089	0.13739
61.261	0.17653	61.090	0.13568
61.261	0.17621	61.090	0.13668
61.261	0.17589	61.090	0.13867
61.262	0.17556	61.090	0.13729
61.262	0.17524	61.090	0.135
61.262	0.17491	61.090	0.13532
61.262	0.17457	61.090	0.13472
61.262	0.17424	61.091	0.13547
61.262	0.1739	61.091	0.13524
61.262	0.17356	61.091	0.13443
61.263	0.17321	61.091	0.13534
61.263	0.17287	61.091	0.13407
61.263	0.17252	61.091	0.13477
61.263	0.17217	61.092	0.13376
61.263	0.17182	61.092	0.13363
61.263	0.17147	61.092	0.1354

61.263	0.17111	61.092	0.13496
61.264	0.17075	61.092	0.13323
61.264	0.17039	61.092	0.1331
61.264	0.17003	61.092	0.13375
61.264	0.16966	61.093	0.13284
61.264	0.1693	61.093	0.13271
61.264	0.16893	61.093	0.13377
61.264	0.16856	61.093	0.13342
61.265	0.16819	61.093	0.13666
61.265	0.16783	61.093	0.13641
61.265	0.16746	61.094	0.13404
61.265	0.16709	61.094	0.13195
61.265	0.16672	61.094	0.13215
61.265	0.16635	61.094	0.1317
61.265	0.16598	61.094	0.13157
61.266	0.16561	61.094	0.13144
61.266	0.16523	61.094	0.13139
61.266	0.16492	61.095	0.13118
61.266	0.16522	61.095	0.13452
61.266	0.16552	61.095	0.13439
61.266	0.16581	61.095	0.13227
61.266	0.1661	61.095	0.13163
61.266	0.16638	61.095	0.13113
61.267	0.16665	61.096	0.13038
61.267	0.16692	61.096	0.13025
61.267	0.16718	61.096	0.13011
61.267	0.16744	61.096	0.12997
61.267	0.16769	61.096	0.13052
61.267	0.16794	61.096	0.13319
61.267	0.16818	61.096	0.13267
61.268	0.16841	61.097	0.13116
61.268	0.16864	61.097	0.13318
61.268	0.16886	61.097	0.133
61.268	0.16908	61.097	0.13084
61.268	0.16929	61.097	0.12898
61.268	0.16949	61.097	0.12864
61.268	0.16969	61.098	0.12847
61.269	0.16988	61.098	0.12832
61.269	0.17006	61.098	0.1296
61.269	0.17024	61.098	0.1285
61.269	0.17042	61.098	0.12789
61.269	0.17058	61.098	0.12773
61.269	0.17074	61.099	0.12758
61.269	0.1709	61.099	0.12744
61.270	0.17105	61.099	0.12896
61.270	0.17119	61.099	0.12867
61.270	0.17133	61.099	0.12834
61.270	0.17146	61.099	0.12856
61.270	0.1716	61.099	0.13113
61.270	0.17174	61.100	0.13089
61.270	0.17187	61.100	0.12868
61.271	0.172	61.100	0.12636
61.271	0.17212	61.100	0.12791

61.271	0.17223	61.100	0.12673
61.271	0.17233	61.100	0.12599
61.271	0.17243	61.100	0.12592
61.271	0.17252	61.101	0.12919
61.271	0.1726	61.101	0.12854
61.272	0.17268	61.101	0.126
61.272	0.17275	61.101	0.12635
61.272	0.17281	61.101	0.12878
61.272	0.17287	61.101	0.12704
61.272	0.17292	61.102	0.12542
61.272	0.17297	61.102	0.1263
61.272	0.17301	61.102	0.12549
61.273	0.17304	61.102	0.12522
61.273	0.17307	61.102	0.12515
61.273	0.17309	61.102	0.12743
61.273	0.17311	61.102	0.12738
61.273	0.17312	61.103	0.12598
61.273	0.17313	61.103	0.12493
61.273	0.17313	61.103	0.12716
61.273	0.17313	61.103	0.12759
61.274	0.17312	61.103	0.12735
61.274	0.17311	61.103	0.12691
61.274	0.17309	61.103	0.12705
61.274	0.17307	61.104	0.12737
61.274	0.17304	61.104	0.1255
61.274	0.173	61.104	0.12453
61.274	0.17297	61.104	0.12449
61.275	0.17292	61.104	0.12446
61.275	0.17287	61.104	0.12688
61.275	0.17282	61.104	0.12657
61.275	0.17276	61.105	0.12476
61.275	0.1727	61.105	0.12429
61.275	0.17263	61.105	0.12424
61.275	0.17255	61.105	0.12554
61.276	0.17247	61.105	0.12705
61.276	0.17239	61.105	0.12409
61.276	0.1723	61.105	0.12878
61.276	0.1722	61.106	0.12397
61.276	0.1721	61.106	0.13195
61.276	0.17199	61.106	0.12385
61.276	0.17188	61.106	0.13011
61.277	0.17176	61.106	0.13025
61.277	0.17164	61.106	0.12495
61.277	0.17151	61.106	0.12476
61.277	0.17138	61.107	0.12747
61.277	0.17124	61.107	0.12718
61.277	0.1711	61.107	0.1245
61.277	0.17095	61.107	0.12323
61.278	0.1708	61.107	0.12314
61.278	0.17064	61.107	0.12305
61.278	0.17047	61.107	0.12296
61.278	0.1703	61.108	0.12308
61.278	0.17012	61.108	0.12277

61.278	0.16994	61.108	0.12267
61.278	0.16975	61.108	0.12257
61.279	0.16955	61.108	0.12246
61.279	0.16935	61.108	0.12235
61.279	0.16914	61.108	0.12224
61.279	0.16892	61.109	0.12212
61.279	0.1687	61.109	0.122
61.279	0.16847	61.109	0.12188
61.279	0.16824	61.109	0.12175
61.279	0.168	61.109	0.12163
61.280	0.16776	61.109	0.1215
61.280	0.16751	61.109	0.12138
61.280	0.16725	61.110	0.12125
61.280	0.16699	61.110	0.12112
61.280	0.16672	61.110	0.12193
61.280	0.16644	61.110	0.12302
61.280	0.16616	61.110	0.122
61.281	0.16587	61.110	0.12098
61.281	0.16558	61.110	0.12094
61.281	0.16528	61.111	0.1209
61.281	0.16497	61.111	0.12087
61.281	0.16466	61.111	0.12083
61.281	0.16434	61.111	0.12078
61.281	0.16402	61.111	0.12073
61.282	0.16369	61.111	0.12068
61.282	0.16335	61.111	0.12062
61.282	0.16301	61.112	0.12056
61.282	0.16266	61.112	0.1205
61.282	0.16231	61.112	0.12043
61.282	0.16195	61.112	0.12037
61.282	0.16159	61.112	0.1203
61.283	0.16122	61.112	0.12023
61.283	0.16084	61.112	0.12016
61.283	0.16046	61.113	0.12009
61.283	0.16008	61.113	0.12002
61.283	0.15969	61.113	0.11995
61.283	0.1593	61.113	0.11988
61.283	0.1589	61.113	0.11981
61.284	0.1585	61.113	0.11974
61.284	0.15809	61.113	0.11966
61.284	0.15768	61.114	0.11959
61.284	0.15727	61.114	0.11952
61.284	0.15685	61.114	0.11945
61.284	0.15643	61.114	0.11938
61.284	0.15601	61.114	0.11987
61.284	0.15558	61.114	0.11923
61.285	0.15514	61.114	0.12059
61.285	0.15471	61.115	0.11907
61.285	0.15427	61.115	0.12207
61.285	0.15383	61.115	0.12292
61.285	0.15339	61.115	0.12019
61.285	0.15296	61.115	0.11874
61.285	0.15252	61.115	0.11866

61.286	0.15208	61.115	0.11857
61.286	0.15163	61.116	0.11848
61.286	0.15118	61.116	0.1184
61.286	0.15074	61.116	0.11831
61.286	0.15028	61.116	0.11822
61.286	0.14983	61.116	0.11881
61.286	0.14938	61.116	0.11932
61.287	0.14892	61.116	0.11794
61.287	0.14846	61.117	0.11932
61.287	0.148	61.117	0.11775
61.287	0.14754	61.117	0.11765
61.287	0.14708	61.117	0.11755
61.287	0.14661	61.117	0.11745
61.287	0.14615	61.117	0.11734
61.288	0.14569	61.117	0.11724
61.288	0.14525	61.118	0.11714
61.288	0.14481	61.118	0.11703
61.288	0.14437	61.118	0.11693
61.288	0.14393	61.118	0.11683
61.288	0.14349	61.118	0.11672
61.288	0.14304	61.118	0.11661
61.289	0.14259	61.118	0.11651
61.289	0.14214	61.119	0.1164
61.289	0.14169	61.119	0.11629
61.289	0.14166	61.119	0.11618
61.289	0.14078	61.119	0.11607
61.289	0.14032	61.119	0.11596
61.289	0.13986	61.119	0.11585
61.290	0.1394	61.119	0.11574
61.290	0.13894	61.120	0.11563
61.290	0.13847	61.120	0.11551
61.290	0.13801	61.120	0.1154
61.290	0.13888	61.120	0.11528
61.290	0.13908	61.120	0.11516
61.290	0.13921	61.120	0.11504
61.290	0.14019	61.120	0.11492
61.291	0.13873	61.121	0.11479
61.291	0.13628	61.121	0.11466
61.291	0.13722	61.121	0.11453
61.291	0.13765	61.121	0.1144
61.291	0.13708	61.121	0.11427
61.291	0.13565	61.121	0.11413
61.291	0.13733	61.121	0.114
61.292	0.1378	61.122	0.11386
61.292	0.13629	61.122	0.11372
61.292	0.13614	61.122	0.11359
61.292	0.13508	61.122	0.11345
61.292	0.1348	61.122	0.11331
61.292	0.135	61.122	0.11317
61.292	0.13407	61.122	0.11328
61.293	0.13601	61.122	0.11316
61.293	0.13562	61.123	0.11289
61.293	0.1352	61.123	0.11283

61.293	0.13579	61.123	0.11249
61.293	0.13423	61.123	0.11258
61.293	0.13361	61.123	0.11239
61.293	0.13353	61.123	0.11209
61.294	0.13344	61.123	0.11196
61.294	0.13462	61.124	0.11335
61.294	0.13384	61.124	0.11343
61.294	0.13433	61.124	0.11176
61.294	0.13477	61.124	0.11144
61.294	0.13306	61.124	0.11132
61.294	0.13291	61.124	0.11121
61.295	0.13287	61.124	0.113
61.295	0.13282	61.125	0.113
61.295	0.1333	61.125	0.11131
61.295	0.1339	61.125	0.11111
61.295	0.13268	61.125	0.11108
61.295	0.13262	61.125	0.11104
61.295	0.13257	61.125	0.111
61.295	0.13286	61.125	0.1114
61.296	0.1326	61.126	0.11143
61.296	0.13293	61.126	0.1109
61.296	0.13233	61.126	0.11082
61.296	0.13226	61.126	0.11145
61.296	0.13219	61.126	0.11153
61.296	0.13211	61.126	0.11066
61.296	0.13203	61.126	0.11246
61.297	0.13195	61.127	0.11256
61.297	0.13185	61.127	0.11087
61.297	0.13176	61.127	0.11041
61.297	0.13166	61.127	0.11034
61.297	0.13156	61.127	0.11027
61.297	0.13146	61.127	0.1102
61.297	0.13138	61.127	0.11013
61.298	0.13129	61.128	0.1108
61.298	0.13119	61.128	0.11072
61.298	0.1311	61.128	0.11072
61.298	0.13101	61.128	0.11078
61.298	0.13091	61.128	0.10979
61.298	0.13083	61.128	0.10971
61.298	0.13074	61.128	0.10964
61.299	0.13065	61.128	0.10955
61.299	0.13054	61.129	0.10947
61.299	0.13045	61.129	0.10938
61.299	0.13036	61.129	0.10929
61.299	0.13025	61.129	0.1092
61.299	0.13013	61.129	0.1091
61.299	0.13001	61.129	0.10901
61.300	0.1299	61.129	0.10987
61.300	0.12978	61.130	0.10996
61.300	0.12966	61.130	0.1087
61.300	0.12954	61.130	0.1086
61.300	0.1294	61.130	0.11003
61.300	0.12927	61.130	0.10838

61.300	0.12914	61.130	0.10827
61.301	0.12901	61.130	0.10816
61.301	0.12887	61.131	0.11014
61.301	0.12874	61.131	0.11015
61.301	0.1286	61.131	0.10846
61.301	0.12846	61.131	0.10771
61.301	0.12832	61.131	0.10759
61.301	0.12817	61.131	0.10747
61.302	0.12803	61.131	0.10746
61.302	0.12789	61.132	0.10723
61.302	0.12774	61.132	0.10711
61.302	0.1276	61.132	0.10752
61.302	0.12745	61.132	0.10761
61.302	0.12732	61.132	0.10698
61.302	0.12718	61.132	0.10705
61.303	0.12702	61.132	0.10666
61.303	0.12686	61.133	0.10724
61.303	0.12671	61.133	0.10769
61.303	0.12657	61.133	0.10778
61.303	0.12643	61.133	0.10627
61.303	0.12629	61.133	0.10821
61.303	0.12614	61.133	0.10871
61.304	0.126	61.133	0.10813
61.304	0.12585	61.133	0.1082
61.304	0.1257	61.134	0.1073
61.304	0.12555	61.134	0.10791
61.304	0.1254	61.134	0.10768
61.304	0.12524	61.134	0.10793
61.304	0.12508	61.134	0.10798
61.304	0.12492	61.134	0.10616
61.305	0.12475	61.134	0.10683
61.305	0.12459	61.135	0.10666
61.305	0.12442	61.135	0.10541
61.305	0.12425	61.135	0.1056
61.305	0.12408	61.135	0.10659
61.305	0.1239	61.135	0.10661
61.305	0.12372	61.135	0.10679
61.306	0.12354	61.135	0.10672
61.306	0.12341	61.136	0.10617
61.306	0.12362	61.136	0.10635
61.306	0.12383	61.136	0.1071
61.306	0.12405	61.136	0.10822
61.306	0.12426	61.136	0.10724
61.306	0.12446	61.136	0.10539
61.307	0.12467	61.136	0.10571
61.307	0.12486	61.137	0.10553
61.307	0.12506	61.137	0.1057
61.307	0.12525	61.137	0.10556
61.307	0.12544	61.137	0.10676
61.307	0.12562	61.137	0.10636
61.307	0.1258	61.137	0.10623
61.308	0.12598	61.137	0.10627
61.308	0.12614	61.137	0.1057

61.308	0.12631	61.138	0.10518
61.308	0.12647	61.138	0.1052
61.308	0.12662	61.138	0.10522
61.308	0.12677	61.138	0.10523
61.308	0.12692	61.138	0.10525
61.309	0.12706	61.138	0.10526
61.309	0.12719	61.138	0.10527
61.309	0.12732	61.139	0.10527
61.309	0.12744	61.139	0.10528
61.309	0.12763	61.139	0.10528
61.309	0.12791	61.139	0.10529
61.309	0.12818	61.139	0.10566
61.310	0.12848	61.139	0.10574
61.310	0.1288	61.139	0.1053
61.310	0.12911	61.140	0.10531
61.310	0.12941	61.140	0.10532
61.310	0.1297	61.140	0.10533
61.310	0.12998	61.140	0.10534
61.310	0.13026	61.140	0.10535
61.311	0.13052	61.140	0.10536
61.311	0.13078	61.140	0.10537
61.311	0.13102	61.141	0.10538
61.311	0.13126	61.141	0.10539
61.311	0.1315	61.141	0.1054
61.311	0.13174	61.141	0.1054
61.311	0.13196	61.141	0.1054
61.312	0.13217	61.141	0.1054
61.312	0.13237	61.141	0.1054
61.312	0.13256	61.141	0.1054
61.312	0.13273	61.142	0.1054
61.312	0.13288	61.142	0.10539
61.312	0.13303	61.142	0.10539
61.312	0.13316	61.142	0.10538
61.313	0.13328	61.142	0.10537
61.313	0.13338	61.142	0.10536
61.313	0.13348	61.142	0.10536
61.313	0.13356	61.143	0.10535
61.313	0.13362	61.143	0.10534
61.313	0.13368	61.143	0.10533
61.313	0.13372	61.143	0.10532
61.314	0.13375	61.143	0.10531
61.314	0.13377	61.143	0.10529
61.314	0.13378	61.143	0.10528
61.314	0.13378	61.144	0.10526
61.314	0.13377	61.144	0.10524
61.314	0.13375	61.144	0.10521
61.314	0.13372	61.144	0.10519
61.315	0.13368	61.144	0.10516
61.315	0.13364	61.144	0.10513
61.315	0.13358	61.144	0.10511
61.315	0.13352	61.145	0.10508
61.315	0.13345	61.145	0.10506
61.315	0.13337	61.145	0.10504

61.315	0.13329	61.145	0.10502
61.316	0.1332	61.145	0.105
61.316	0.13311	61.145	0.10499
61.316	0.13301	61.145	0.10498
61.316	0.13291	61.145	0.10497
61.316	0.1328	61.146	0.10496
61.316	0.13268	61.146	0.10495
61.316	0.13257	61.146	0.10494
61.316	0.13244	61.146	0.10493
61.317	0.13232	61.146	0.10491
61.317	0.13219	61.146	0.1049
61.317	0.13206	61.146	0.10487
61.317	0.13192	61.147	0.10485
61.317	0.13178	61.147	0.10482
61.317	0.13164	61.147	0.10479
61.317	0.13149	61.147	0.10476
61.318	0.13135	61.147	0.10472
61.318	0.1312	61.147	0.10468
61.318	0.13104	61.147	0.10465
61.318	0.13089	61.148	0.10461
61.318	0.13073	61.148	0.10457
61.318	0.13057	61.148	0.10453
61.318	0.13041	61.148	0.10449
61.319	0.13025	61.148	0.10445
61.319	0.13008	61.148	0.1044
61.319	0.12991	61.148	0.10436
61.319	0.12974	61.149	0.10432
61.319	0.12957	61.149	0.10427
61.319	0.1294	61.149	0.10422
61.319	0.12922	61.149	0.10417
61.320	0.12904	61.149	0.10412
61.320	0.12886	61.149	0.10407
61.320	0.12868	61.149	0.10402
61.320	0.12849	61.149	0.10397
61.320	0.1283	61.150	0.10391
61.320	0.12811	61.150	0.10386
61.320	0.12792	61.150	0.10381
61.321	0.12773	61.150	0.10377
61.321	0.12753	61.150	0.10374
61.321	0.12734	61.150	0.10378
61.321	0.12714	61.150	0.10382
61.321	0.12694	61.151	0.10386
61.321	0.12673	61.151	0.1039
61.321	0.12653	61.151	0.10393
61.322	0.12632	61.151	0.10397
61.322	0.12611	61.151	0.104
61.322	0.1259	61.151	0.10404
61.322	0.12569	61.151	0.1041
61.322	0.12547	61.152	0.10418
61.322	0.12526	61.152	0.10425
61.322	0.12504	61.152	0.10432
61.323	0.12482	61.152	0.10439
61.323	0.1246	61.152	0.10447

61.323	0.12438	61.152	0.10454
61.323	0.12427	61.152	0.10461
61.323	0.12416	61.153	0.10469
61.323	0.12405	61.153	0.10476
61.323	0.12393	61.153	0.10483
61.324	0.12381	61.153	0.1049
61.324	0.12369	61.153	0.10497
61.324	0.12357	61.153	0.10504
61.324	0.12344	61.153	0.1051
61.324	0.12331	61.153	0.10517
61.324	0.12318	61.154	0.10524
61.324	0.12305	61.154	0.10531
61.325	0.12291	61.154	0.10537
61.325	0.12277	61.154	0.10544
61.325	0.12263	61.154	0.1055
61.325	0.12249	61.154	0.10557
61.325	0.12234	61.154	0.10563
61.325	0.1222	61.155	0.1057
61.325	0.12205	61.155	0.10577
61.326	0.1219	61.155	0.10583
61.326	0.12174	61.155	0.1059
61.326	0.12159	61.155	0.10597
61.326	0.12145	61.155	0.10604
61.326	0.12132	61.155	0.10611
61.326	0.12118	61.156	0.10619
61.326	0.12104	61.156	0.10626
61.327	0.12089	61.156	0.10633
61.327	0.12074	61.156	0.1064
61.327	0.12059	61.156	0.10647
61.327	0.12044	61.156	0.10653
61.327	0.12029	61.156	0.10659
61.327	0.12013	61.156	0.10664
61.327	0.11997	61.157	0.10669
61.328	0.11981	61.157	0.10674
61.328	0.11964	61.157	0.10677
61.328	0.11948	61.157	0.10681
61.328	0.11931	61.157	0.10683
61.328	0.11914	61.157	0.10685
61.328	0.11896	61.157	0.10686
61.328	0.11879	61.158	0.10687
61.329	0.11861	61.158	0.10687
61.329	0.11843	61.158	0.10687
61.329	0.11824	61.158	0.10686
61.329	0.11806	61.158	0.10684
61.329	0.11787	61.158	0.10683
61.329	0.11769	61.158	0.10681
61.329	0.1175	61.159	0.1068
61.330	0.11733	61.159	0.10678
61.330	0.11733	61.159	0.10677
61.330	0.11734	61.159	0.10676
61.330	0.11734	61.159	0.10675
61.330	0.11733	61.159	0.10674
61.330	0.11731	61.159	0.10674

61.330	0.11728	61.160	0.10674
61.331	0.11736	61.160	0.10674
61.331	0.11752	61.160	0.10675
61.331	0.11768	61.160	0.10676
61.331	0.11783	61.160	0.10677
61.331	0.11798	61.160	0.10678
61.331	0.11812	61.160	0.1068
61.331	0.11826	61.160	0.10682
61.332	0.1184	61.161	0.10684
61.332	0.11854	61.161	0.10686
61.332	0.11867	61.161	0.10688
61.332	0.11881	61.161	0.1069
61.332	0.11894	61.161	0.10692
61.332	0.11907	61.161	0.10695
61.332	0.1192	61.161	0.10697
61.333	0.11932	61.162	0.107
61.333	0.11943	61.162	0.10702
61.333	0.11953	61.162	0.10705
61.333	0.11964	61.162	0.10707
61.333	0.11975	61.162	0.10709
61.333	0.11985	61.162	0.10711
61.333	0.11994	61.162	0.10713
61.334	0.12003	61.163	0.10716
61.334	0.12012	61.163	0.1072
61.334	0.1202	61.163	0.10723
61.334	0.12029	61.163	0.10727
61.334	0.12037	61.163	0.1073
61.334	0.12046	61.163	0.10733
61.334	0.12054	61.163	0.10736
61.335	0.12063	61.164	0.10739
61.335	0.1207	61.164	0.10742
61.335	0.12077	61.164	0.10744
61.335	0.12084	61.164	0.10747
61.335	0.12091	61.164	0.10749
61.335	0.12097	61.164	0.10752
61.335	0.12102	61.164	0.10754
61.336	0.12107	61.164	0.10756
61.336	0.1211	61.165	0.10758
61.336	0.12115	61.165	0.1076
61.336	0.12118	61.165	0.10761
61.336	0.12121	61.165	0.10763
61.336	0.12124	61.165	0.10764
61.336	0.12127	61.165	0.10766
61.337	0.12129	61.165	0.10767
61.337	0.12132	61.166	0.10768
61.337	0.12135	61.166	0.10769
61.337	0.12138	61.166	0.1077
61.337	0.12141	61.166	0.10772
61.337	0.12144	61.166	0.10773
61.337	0.12145	61.166	0.10774
61.338	0.12147	61.166	0.10775
61.338	0.12148	61.167	0.10776
61.338	0.12148	61.167	0.10777

61.338	0.12147	61.167	0.10778
61.338	0.12145	61.167	0.10779
61.338	0.12143	61.167	0.1078
61.338	0.12141	61.167	0.10781
61.339	0.12137	61.167	0.10781
61.339	0.12145	61.168	0.10782
61.339	0.12152	61.168	0.10783
61.339	0.12159	61.168	0.10783
61.339	0.12164	61.168	0.10784
61.339	0.12169	61.168	0.10784
61.339	0.12173	61.168	0.10785
61.340	0.12176	61.168	0.10785
61.340	0.12179	61.168	0.10785
61.340	0.1218	61.169	0.10785
61.340	0.12181	61.169	0.10785
61.340	0.12181	61.169	0.10785
61.340	0.12181	61.169	0.10785
61.340	0.12181	61.169	0.10786
61.341	0.1218	61.169	0.10786
61.341	0.12178	61.169	0.10786
61.341	0.12176	61.170	0.10786
61.341	0.12173	61.170	0.10786
61.341	0.1217	61.170	0.10786
61.341	0.12166	61.170	0.10786
61.341	0.12161	61.170	0.10786
61.342	0.12156	61.170	0.10785
61.342	0.12151	61.170	0.10785
61.342	0.12144	61.171	0.10785
61.342	0.12137	61.171	0.10785
61.342	0.1213	61.171	0.10785
61.342	0.12122	61.171	0.1079
61.342	0.12114	61.171	0.10796
61.343	0.12105	61.171	0.10802
61.343	0.12096	61.171	0.10808
61.343	0.12086	61.171	0.10814
61.343	0.12075	61.172	0.1082
61.343	0.12065	61.172	0.10826
61.343	0.12053	61.172	0.10831
61.343	0.12042	61.172	0.10837
61.344	0.1203	61.172	0.10842
61.344	0.12017	61.172	0.10848
61.344	0.12005	61.172	0.10853
61.344	0.11992	61.173	0.10858
61.344	0.11979	61.173	0.10863
61.344	0.11966	61.173	0.10868
61.344	0.11952	61.173	0.10873
61.345	0.1204	61.173	0.10878
61.345	0.12014	61.173	0.10883
61.345	0.11908	61.173	0.10887
61.345	0.11893	61.174	0.10892
61.345	0.11878	61.174	0.10896
61.345	0.11998	61.174	0.109
61.345	0.11952	61.174	0.10904

61.346	0.1183	61.174	0.10908
61.346	0.11813	61.174	0.10912
61.346	0.11797	61.174	0.10915
61.346	0.1178	61.175	0.10919
61.346	0.11763	61.175	0.10922
61.346	0.11745	61.175	0.10926
61.347	0.11907	61.175	0.10929
61.347	0.11907	61.175	0.10932
61.347	0.11819	61.175	0.10934
61.347	0.11699	61.175	0.10937
61.347	0.11659	61.175	0.1094
61.347	0.11642	61.176	0.10942
61.347	0.11673	61.176	0.10945
61.348	0.11897	61.176	0.10947
61.348	0.11876	61.176	0.10949
61.348	0.11759	61.176	0.10951
61.348	0.11614	61.176	0.10953
61.348	0.11582	61.176	0.10954
61.348	0.11574	61.177	0.10956
61.348	0.11565	61.177	0.10958
61.349	0.11557	61.177	0.10959
61.349	0.11549	61.177	0.1096
61.349	0.1154	61.177	0.10961
61.349	0.11532	61.177	0.10962
61.349	0.11523	61.177	0.10963
61.349	0.11514	61.178	0.10963
61.349	0.11506	61.178	0.10964
61.350	0.11497	61.178	0.10964
61.350	0.11487	61.178	0.10964
61.350	0.11484	61.178	0.10964
61.350	0.11499	61.178	0.10964
61.350	0.11514	61.178	0.10964
61.350	0.11529	61.179	0.10963
61.350	0.11544	61.179	0.10963
61.351	0.11559	61.179	0.10962
61.351	0.11575	61.179	0.10961
61.351	0.1159	61.179	0.1096
61.351	0.11608	61.179	0.10958
61.351	0.11629	61.179	0.10957
61.351	0.11649	61.179	0.10955
61.351	0.1167	61.180	0.10953
61.352	0.1169	61.180	0.10952
61.352	0.11711	61.180	0.1095
61.352	0.11732	61.180	0.10947
61.352	0.11752	61.180	0.10945
61.352	0.11773	61.180	0.10943
61.352	0.11793	61.180	0.1094
61.352	0.11814	61.181	0.1095
61.353	0.11834	61.181	0.1096
61.353	0.11854	61.181	0.1097
61.353	0.11874	61.181	0.10981
61.353	0.11894	61.181	0.10991
61.353	0.11913	61.181	0.11

61.353	0.11932	61.181	0.1101
61.353	0.11951	61.182	0.11019
61.354	0.1197	61.182	0.11028
61.354	0.11988	61.182	0.11037
61.354	0.12006	61.182	0.11045
61.354	0.12023	61.182	0.11054
61.354	0.1204	61.182	0.11062
61.354	0.12057	61.182	0.11069
61.354	0.12073	61.183	0.11077
61.355	0.12089	61.183	0.11084
61.355	0.12104	61.183	0.11091
61.355	0.12118	61.183	0.11097
61.355	0.12132	61.183	0.11104
61.355	0.12145	61.183	0.1111
61.355	0.12158	61.183	0.11116
61.355	0.1217	61.183	0.11121
61.356	0.12181	61.184	0.11127
61.356	0.12191	61.184	0.11132
61.356	0.12201	61.184	0.11137
61.356	0.1221	61.184	0.11141
61.356	0.12218	61.184	0.11146
61.356	0.12225	61.184	0.1115
61.356	0.12231	61.184	0.11154
61.356	0.12237	61.185	0.11157
61.357	0.12241	61.185	0.11161
61.357	0.12245	61.185	0.11164
61.357	0.12248	61.185	0.11166
61.357	0.1225	61.185	0.11169
61.357	0.12251	61.185	0.11171
61.357	0.12251	61.185	0.11173
61.357	0.1225	61.186	0.11175
61.358	0.12249	61.186	0.11177
61.358	0.12246	61.186	0.11178
61.358	0.12243	61.186	0.11179
61.358	0.12238	61.186	0.1118
61.358	0.12233	61.186	0.1118
61.358	0.12227	61.186	0.1118
61.358	0.1222	61.187	0.1118
61.359	0.12218	61.187	0.1118
61.359	0.12219	61.187	0.11179
61.359	0.1222	61.187	0.11179
61.359	0.12219	61.187	0.11178
61.359	0.12218	61.187	0.11177
61.359	0.12217	61.187	0.11175
61.359	0.12215	61.187	0.11174
61.360	0.12212	61.188	0.11172
61.360	0.12208	61.188	0.1117
61.360	0.12204	61.188	0.11168
61.360	0.12199	61.188	0.11166
61.360	0.12193	61.188	0.11163
61.360	0.12187	61.188	0.1116
61.360	0.1218	61.188	0.11157
61.361	0.12172	61.189	0.11154

61.361	0.12164	61.189	0.1115
61.361	0.12155	61.189	0.11146
61.361	0.12145	61.189	0.11142
61.361	0.12135	61.189	0.11138
61.361	0.12124	61.189	0.11134
61.361	0.12112	61.189	0.11129
61.362	0.12099	61.190	0.11124
61.362	0.12086	61.190	0.11119
61.362	0.12072	61.190	0.11113
61.362	0.12058	61.190	0.11108
61.362	0.12043	61.190	0.11102
61.362	0.12027	61.190	0.11096
61.362	0.12011	61.190	0.1109
61.363	0.11994	61.191	0.11084
61.363	0.11976	61.191	0.11078
61.363	0.11958	61.191	0.11071
61.363	0.11939	61.191	0.11064
61.363	0.1192	61.191	0.11058
61.363	0.119	61.191	0.11051
61.363	0.11879	61.191	0.11044
61.364	0.11858	61.191	0.11037
61.364	0.11836	61.192	0.1103
61.364	0.11814	61.192	0.11033
61.364	0.11792	61.192	0.11036
61.364	0.1177	61.192	0.11039
61.364	0.11775	61.192	0.11042
61.364	0.11779	61.192	0.11044
61.365	0.11782	61.192	0.11047
61.365	0.11784	61.193	0.11049
61.365	0.11785	61.193	0.11051
61.365	0.11786	61.193	0.11053
61.365	0.11786	61.193	0.11055
61.365	0.11789	61.193	0.11056
61.365	0.11796	61.193	0.11058
61.365	0.11802	61.193	0.11059
61.366	0.11807	61.194	0.11061
61.366	0.11811	61.194	0.11062
61.366	0.11814	61.194	0.11063
61.366	0.11816	61.194	0.11063
61.366	0.11818	61.194	0.11064
61.366	0.11818	61.194	0.11064
61.366	0.11818	61.194	0.11065
61.367	0.11817	61.195	0.11065
61.367	0.11815	61.195	0.11065
61.367	0.11812	61.195	0.11065
61.367	0.11808	61.195	0.11065
61.367	0.11803	61.195	0.11064
61.367	0.11798	61.195	0.11064
61.367	0.11792	61.195	0.11063
61.368	0.11785	61.196	0.11062
61.368	0.11777	61.196	0.11061
61.368	0.11769	61.196	0.1106
61.368	0.1176	61.196	0.11059

61.368	0.11751	61.196	0.11058
61.368	0.1174	61.196	0.11056
61.368	0.11729	61.196	0.11055
61.369	0.11718	61.196	0.11053
61.369	0.11707	61.197	0.11051
61.369	0.11695	61.197	0.11049
61.369	0.11683	61.197	0.11047
61.369	0.11671	61.197	0.11045
61.369	0.11658	61.197	0.11042
61.369	0.11644	61.197	0.1104
61.370	0.1163	61.197	0.11037
61.370	0.11616	61.198	0.11035
61.370	0.116	61.198	0.11032
61.370	0.11585	61.198	0.11029
61.370	0.11569	61.198	0.11026
61.370	0.11552	61.198	0.11023
61.370	0.11535	61.198	0.1102
61.371	0.11518	61.198	0.11017
61.371	0.115	61.199	0.11013
61.371	0.11481	61.199	0.1101
61.371	0.11463	61.199	0.11006
61.371	0.11444	61.199	0.11002
61.371	0.11424	61.199	0.10998
61.371	0.11404	61.199	0.10994
61.371	0.11384	61.199	0.1099
61.372	0.11364	61.200	0.10986
61.372	0.11343	61.200	0.10982
61.372	0.11322	61.200	0.10977
61.372	0.113	61.200	0.10973
61.372	0.11279	61.200	0.10968
61.372	0.11257	61.200	0.10963
61.372	0.11235	61.200	0.10959
61.373	0.11235	61.201	0.10954
61.373	0.11238	61.201	0.10949
61.373	0.11241	61.201	0.10944
61.373	0.11244	61.201	0.10939
61.373	0.11251	61.201	0.10934
61.373	0.11259	61.201	0.10929
61.373	0.11267	61.201	0.10924
61.374	0.11276	61.201	0.10918
61.374	0.11284	61.202	0.10913
61.374	0.11291	61.202	0.10908
61.374	0.11299	61.202	0.10902
61.374	0.11306	61.202	0.10897
61.374	0.11314	61.202	0.10891
61.374	0.11321	61.202	0.10886
61.375	0.11327	61.202	0.1088
61.375	0.11334	61.203	0.10874
61.375	0.1134	61.203	0.10869
61.375	0.11347	61.203	0.10863
61.375	0.11353	61.203	0.10857
61.375	0.11359	61.203	0.10851
61.375	0.11365	61.203	0.10846

61.376	0.1137	61.203	0.1084
61.376	0.11376	61.204	0.10834
61.376	0.11381	61.204	0.10828
61.376	0.11386	61.204	0.10822
61.376	0.11391	61.204	0.10816
61.376	0.11396	61.204	0.1081
61.376	0.11401	61.204	0.10804
61.377	0.11406	61.204	0.10819
61.377	0.1141	61.205	0.10836
61.377	0.11415	61.205	0.10848
61.377	0.11419	61.205	0.10858
61.377	0.11423	61.205	0.10865
61.377	0.11427	61.205	0.10876
61.377	0.11431	61.205	0.10888
61.377	0.11435	61.205	0.109
61.378	0.11438	61.206	0.10912
61.378	0.11442	61.206	0.10924
61.378	0.11445	61.206	0.10936
61.378	0.11448	61.206	0.10948
61.378	0.11451	61.206	0.1096
61.378	0.11454	61.206	0.10972
61.378	0.11456	61.206	0.10983
61.379	0.11458	61.207	0.10995
61.379	0.11461	61.207	0.11007
61.379	0.11463	61.207	0.11019
61.379	0.11465	61.207	0.11031
61.379	0.11466	61.207	0.11043
61.379	0.11468	61.207	0.11054
61.379	0.11469	61.207	0.11065
61.380	0.1147	61.208	0.11076
61.380	0.11471	61.208	0.11087
61.380	0.11472	61.208	0.11098
61.380	0.11473	61.208	0.11109
61.380	0.11473	61.208	0.1112
61.380	0.11474	61.208	0.1113
61.380	0.11474	61.208	0.1114
61.381	0.11474	61.208	0.11151
61.381	0.11473	61.209	0.11161
61.381	0.11473	61.209	0.11171
61.381	0.11473	61.209	0.1118
61.381	0.11472	61.209	0.1119
61.381	0.11471	61.209	0.11199
61.381	0.1147	61.209	0.11209
61.382	0.11469	61.209	0.11218
61.382	0.11468	61.210	0.11227
61.382	0.11466	61.210	0.11236
61.382	0.11465	61.210	0.11245
61.382	0.11463	61.210	0.11253
61.382	0.11467	61.210	0.11262
61.382	0.1147	61.210	0.1127
61.383	0.11473	61.210	0.11278
61.383	0.11476	61.211	0.11286
61.383	0.11479	61.211	0.11293

61.383	0.11482	61.211	0.11301
61.383	0.11484	61.211	0.11308
61.383	0.11487	61.211	0.11315
61.383	0.11489	61.211	0.11322
61.384	0.11491	61.211	0.11328
61.384	0.11493	61.212	0.11335
61.384	0.11494	61.212	0.11341
61.384	0.11495	61.212	0.11347
61.384	0.11496	61.212	0.11352
61.384	0.11497	61.212	0.11358
61.384	0.11498	61.212	0.11363
61.385	0.11499	61.212	0.11368
61.385	0.11499	61.213	0.11372
61.385	0.11499	61.213	0.11377
61.385	0.11499	61.213	0.11381
61.385	0.11498	61.213	0.11385
61.385	0.11498	61.213	0.11388
61.385	0.11497	61.213	0.11392
61.386	0.11496	61.213	0.11395
61.386	0.11495	61.213	0.11398
61.386	0.11494	61.214	0.114
61.386	0.11492	61.214	0.11403
61.386	0.11491	61.214	0.11405
61.386	0.11489	61.214	0.11406
61.386	0.11487	61.214	0.11408
61.387	0.11485	61.214	0.11409
61.387	0.11483	61.214	0.11411
61.387	0.11481	61.215	0.11411
61.387	0.11478	61.215	0.11412
61.387	0.11476	61.215	0.11412
61.387	0.11473	61.215	0.11413
61.387	0.1147	61.215	0.11412
61.388	0.11467	61.215	0.11412
61.388	0.11464	61.215	0.11411
61.388	0.1146	61.216	0.1141
61.388	0.11457	61.216	0.11409
61.388	0.11453	61.216	0.11408
61.388	0.11449	61.216	0.11406
61.388	0.11445	61.216	0.11404
61.389	0.1144	61.216	0.11401
61.389	0.11436	61.216	0.11399
61.389	0.11431	61.217	0.11408
61.389	0.11426	61.217	0.11418
61.389	0.11421	61.217	0.11428
61.389	0.11416	61.217	0.11439
61.389	0.1141	61.217	0.11448
61.390	0.11404	61.217	0.11458
61.390	0.11398	61.217	0.11467
61.390	0.11392	61.218	0.11476
61.390	0.11386	61.218	0.11485
61.390	0.11379	61.218	0.11493
61.390	0.11372	61.218	0.11501
61.390	0.11365	61.218	0.11508

61.391	0.11358	61.218	0.11515
61.391	0.11351	61.218	0.11522
61.391	0.11343	61.218	0.11529
61.391	0.11336	61.219	0.11535
61.391	0.11328	61.219	0.11541
61.391	0.1132	61.219	0.11547
61.391	0.11311	61.219	0.11553
61.392	0.11303	61.219	0.11558
61.392	0.11295	61.219	0.11563
61.392	0.11286	61.219	0.11567
61.392	0.11277	61.220	0.11571
61.392	0.11269	61.220	0.11574
61.392	0.1126	61.220	0.11578
61.392	0.11255	61.220	0.11581
61.393	0.11252	61.220	0.11584
61.393	0.1125	61.220	0.11586
61.393	0.11248	61.220	0.11589
61.393	0.11246	61.221	0.11591
61.393	0.11244	61.221	0.11593
61.393	0.11241	61.221	0.11594
61.393	0.11241	61.221	0.11596
61.394	0.11249	61.221	0.11597
61.394	0.11257	61.221	0.11598
61.394	0.11265	61.221	0.11599
61.394	0.11272	61.222	0.116
61.394	0.1128	61.222	0.116
61.394	0.11288	61.222	0.116
61.394	0.11296	61.222	0.116
61.395	0.11304	61.222	0.11599
61.395	0.11312	61.222	0.11598
61.395	0.11319	61.222	0.11597
61.395	0.11327	61.222	0.11595
61.395	0.11334	61.223	0.11594
61.395	0.11342	61.223	0.11592
61.395	0.11349	61.223	0.1159
61.396	0.11356	61.223	0.11588
61.396	0.11363	61.223	0.11585
61.396	0.1137	61.223	0.11582
61.396	0.11377	61.223	0.11581
61.396	0.11383	61.224	0.11581
61.396	0.1139	61.224	0.1158
61.396	0.11396	61.224	0.11579
61.397	0.11403	61.224	0.11577
61.397	0.11408	61.224	0.11576
61.397	0.11414	61.224	0.11574
61.397	0.1142	61.224	0.11571
61.397	0.11425	61.225	0.11569
61.397	0.1143	61.225	0.11566
61.398	0.11434	61.225	0.11563
61.398	0.11439	61.225	0.1156
61.398	0.11443	61.225	0.11557
61.398	0.11447	61.225	0.11553
61.398	0.1145	61.225	0.11549

61.398	0.11454	61.226	0.11544
61.398	0.11457	61.226	0.1154
61.399	0.1146	61.226	0.11535
61.399	0.11463	61.226	0.11535
61.399	0.11466	61.226	0.11537
61.399	0.11468	61.226	0.11539
61.399	0.11471	61.226	0.1154
61.399	0.11473	61.227	0.11541
61.399	0.11475	61.227	0.11542
61.400	0.11477	61.227	0.11543
61.400	0.11478	61.227	0.11544
61.400	0.1148	61.227	0.11544
61.400	0.11481	61.227	0.11544
61.400	0.11481	61.227	0.11543
61.400	0.11482	61.227	0.11543
61.400	0.11482	61.228	0.11542
61.401	0.11482	61.228	0.11541
61.401	0.11481	61.228	0.1154
61.401	0.1148	61.228	0.11538
61.401	0.11479	61.228	0.11536
61.401	0.11478	61.228	0.11534
61.401	0.11476	61.228	0.11532
61.401	0.11474	61.229	0.11529
61.402	0.11471	61.229	0.11526
61.402	0.11468	61.229	0.11523
61.402	0.11465	61.229	0.11519
61.402	0.11462	61.229	0.11516
61.402	0.11459	61.229	0.11512
61.402	0.11455	61.229	0.11507
61.402	0.11451	61.230	0.11503
61.403	0.11447	61.230	0.11498
61.403	0.11442	61.230	0.11493
61.403	0.11437	61.230	0.11488
61.403	0.11432	61.230	0.11482
61.403	0.11427	61.230	0.11477
61.403	0.11422	61.230	0.11471
61.404	0.11416	61.231	0.11464
61.404	0.1141	61.231	0.11458
61.404	0.11411	61.231	0.11451
61.404	0.11418	61.231	0.11444
61.404	0.11425	61.231	0.11437
61.404	0.11432	61.231	0.1143
61.404	0.11438	61.231	0.11422
61.405	0.11445	61.232	0.11414
61.405	0.1145	61.232	0.11406
61.405	0.11456	61.232	0.11398
61.405	0.11461	61.232	0.11389
61.405	0.11465	61.232	0.11381
61.405	0.11469	61.232	0.11372
61.405	0.11473	61.232	0.11363
61.406	0.11477	61.232	0.11353
61.406	0.1148	61.233	0.11344
61.406	0.11483	61.233	0.11334

61.406	0.11485	61.233	0.11324
61.406	0.11488	61.233	0.11314
61.406	0.1149	61.233	0.11304
61.406	0.11492	61.233	0.11293
61.407	0.11494	61.233	0.11283
61.407	0.11495	61.234	0.11272
61.407	0.11496	61.234	0.11261
61.407	0.11497	61.234	0.1125
61.407	0.11498	61.234	0.11238
61.407	0.11498	61.234	0.11227
61.407	0.11498	61.234	0.11215
61.408	0.11497	61.234	0.11203
61.408	0.11496	61.235	0.11191
61.408	0.11495	61.235	0.11179
61.408	0.11494	61.235	0.11166
61.408	0.11491	61.235	0.11154
61.408	0.11489	61.235	0.11141
61.408	0.11486	61.235	0.11128
61.409	0.11483	61.235	0.11115
61.409	0.1148	61.236	0.11103
61.409	0.11476	61.236	0.11089
61.409	0.11472	61.236	0.11076
61.409	0.11468	61.236	0.11063
61.409	0.11463	61.236	0.1105
61.409	0.11458	61.236	0.11048
61.410	0.11453	61.236	0.1105
61.410	0.11447	61.237	0.11053
61.410	0.11441	61.237	0.11055
61.410	0.11435	61.237	0.11057
61.410	0.11429	61.237	0.1106
61.410	0.11422	61.237	0.11062
61.411	0.11415	61.237	0.11064
61.411	0.11407	61.237	0.11067
61.411	0.114	61.238	0.11069
61.411	0.11392	61.238	0.11071
61.411	0.11384	61.238	0.11073
61.411	0.11376	61.238	0.11076
61.411	0.11367	61.238	0.11078
61.412	0.11359	61.238	0.1108
61.412	0.1135	61.238	0.11082
61.412	0.11341	61.238	0.11093
61.412	0.11331	61.239	0.11108
61.412	0.11322	61.239	0.11124
61.412	0.11312	61.239	0.11139
61.412	0.11302	61.239	0.11154
61.413	0.11292	61.239	0.11169
61.413	0.11282	61.239	0.11184
61.413	0.11271	61.239	0.11199
61.413	0.1126	61.240	0.11214
61.413	0.11249	61.240	0.11228
61.413	0.11238	61.240	0.11243
61.413	0.11227	61.240	0.11257
61.414	0.11216	61.240	0.11271

61.414	0.11204	61.240	0.11285
61.414	0.11192	61.240	0.11299
61.414	0.11181	61.241	0.11313
61.414	0.11169	61.241	0.11327
61.414	0.11157	61.241	0.1134
61.414	0.11144	61.241	0.11354
61.415	0.11132	61.241	0.11367
61.415	0.11122	61.241	0.1138
61.415	0.11112	61.241	0.11393
61.415	0.11102	61.242	0.11406
61.415	0.11091	61.242	0.11418
61.415	0.11081	61.242	0.11431
61.415	0.1107	61.242	0.11443
61.416	0.11066	61.242	0.11456
61.416	0.11075	61.242	0.11468
61.416	0.11084	61.242	0.1148
61.416	0.11092	61.243	0.11492
61.416	0.111	61.243	0.11503
61.416	0.11108	61.243	0.11515
61.417	0.11116	61.243	0.11526
61.417	0.11123	61.243	0.11537
61.417	0.11131	61.243	0.11549
61.417	0.11137	61.243	0.1156
61.417	0.11144	61.244	0.1157
61.417	0.11151	61.244	0.11581
61.417	0.11157	61.244	0.11592
61.418	0.11163	61.244	0.11602
61.418	0.11168	61.244	0.11612
61.418	0.11174	61.244	0.11623
61.418	0.11179	61.244	0.11633
61.418	0.11184	61.244	0.11642
61.418	0.11188	61.245	0.11652
61.418	0.11193	61.245	0.11662
61.419	0.11198	61.245	0.11671
61.419	0.11203	61.245	0.1168
61.419	0.11207	61.245	0.11689
61.419	0.11212	61.245	0.11698
61.419	0.11216	61.245	0.11707
61.419	0.11219	61.246	0.11716
61.419	0.11223	61.246	0.11725
61.420	0.11226	61.246	0.11733
61.420	0.11229	61.246	0.11741
61.420	0.11232	61.246	0.11749
61.420	0.11234	61.246	0.11757
61.420	0.11236	61.246	0.11765
61.420	0.11238	61.247	0.11773
61.420	0.11239	61.247	0.1178
61.421	0.1124	61.247	0.11787
61.421	0.11241	61.247	0.11795
61.421	0.11242	61.247	0.11802
61.421	0.11242	61.247	0.11809
61.421	0.11242	61.247	0.11815
61.421	0.11242	61.248	0.11822

61.422	0.11242	61.248	0.11828
61.422	0.11241	61.248	0.11834
61.422	0.1124	61.248	0.11841
61.422	0.11239	61.248	0.11847
61.422	0.11237	61.248	0.11852
61.422	0.11235	61.248	0.11858
61.422	0.11233	61.249	0.11864
61.423	0.11231	61.249	0.11869
61.423	0.11228	61.249	0.11874
61.423	0.11225	61.249	0.11879
61.423	0.11222	61.249	0.11884
61.423	0.11219	61.249	0.11889
61.423	0.11215	61.249	0.11893
61.423	0.11211	61.249	0.11898
61.424	0.11207	61.250	0.11902
61.424	0.11203	61.250	0.11906
61.424	0.11198	61.250	0.1191
61.424	0.11193	61.250	0.11914
61.424	0.11188	61.250	0.11917
61.424	0.11183	61.250	0.11921
61.424	0.11178	61.250	0.11924
61.425	0.11172	61.251	0.11927
61.425	0.11166	61.251	0.1193
61.425	0.1116	61.251	0.11933
61.425	0.11153	61.251	0.11936
61.425	0.11147	61.251	0.11938
61.425	0.1114	61.251	0.1194
61.425	0.11133	61.251	0.11942
61.426	0.11126	61.252	0.11944
61.426	0.11119	61.252	0.11946
61.426	0.11112	61.252	0.11948
61.426	0.11104	61.252	0.11949
61.426	0.11096	61.252	0.11951
61.426	0.11088	61.252	0.11952
61.427	0.1108	61.252	0.11953
61.427	0.11072	61.253	0.11953
61.427	0.11063	61.253	0.11954
61.427	0.11055	61.253	0.11954
61.427	0.11046	61.253	0.11955
61.427	0.11037	61.253	0.11955
61.427	0.11029	61.253	0.11955
61.428	0.1102	61.253	0.11954
61.428	0.11011	61.254	0.11954
61.428	0.11001	61.254	0.11953
61.428	0.10992	61.254	0.11958
61.428	0.10983	61.254	0.11968
61.428	0.10973	61.254	0.11977
61.428	0.10963	61.254	0.11986
61.429	0.10953	61.254	0.11995
61.429	0.10943	61.254	0.12003
61.429	0.10933	61.255	0.12011
61.429	0.10923	61.255	0.12019
61.429	0.10913	61.255	0.12027

61.429	0.10902	61.255	0.12034
61.429	0.10892	61.255	0.12042
61.430	0.10881	61.255	0.12049
61.430	0.1087	61.255	0.12056
61.430	0.10859	61.256	0.12062
61.430	0.1085	61.256	0.12069
61.430	0.10841	61.256	0.12075
61.430	0.10831	61.256	0.12081
61.430	0.10822	61.256	0.12087
61.431	0.10812	61.256	0.12092
61.431	0.10803	61.256	0.12098
61.431	0.10793	61.257	0.12103
61.431	0.10783	61.257	0.12108
61.431	0.10787	61.257	0.12112
61.431	0.10794	61.257	0.12117
61.432	0.10801	61.257	0.12121
61.432	0.10808	61.257	0.12125
61.432	0.10815	61.257	0.12128
61.432	0.10822	61.258	0.12132
61.432	0.10828	61.258	0.12135
61.432	0.10835	61.258	0.12138
61.432	0.10841	61.258	0.1214
61.433	0.10847	61.258	0.12143
61.433	0.10853	61.258	0.12145
61.433	0.10859	61.258	0.12147
61.433	0.10865	61.258	0.12148
61.433	0.1087	61.259	0.1215
61.433	0.10876	61.259	0.12151
61.433	0.10881	61.259	0.12152
61.434	0.10886	61.259	0.12152
61.434	0.10891	61.259	0.12153
61.434	0.10896	61.259	0.12153
61.434	0.109	61.259	0.12152
61.434	0.10905	61.260	0.12152
61.434	0.10909	61.260	0.12151
61.434	0.10913	61.260	0.12151
61.435	0.10917	61.260	0.12149
61.435	0.10921	61.260	0.12148
61.435	0.10925	61.260	0.12146
61.435	0.10929	61.260	0.12145
61.435	0.10932	61.261	0.12142
61.435	0.10936	61.261	0.1214
61.436	0.10939	61.261	0.12137
61.436	0.10942	61.261	0.12135
61.436	0.10945	61.261	0.12132
61.436	0.10949	61.261	0.12128
61.436	0.10953	61.261	0.12125
61.436	0.10956	61.262	0.12121
61.436	0.10959	61.262	0.12117
61.437	0.10963	61.262	0.12113
61.437	0.10966	61.262	0.12109
61.437	0.10969	61.262	0.12104
61.437	0.10971	61.262	0.121

61.437	0.10974	61.262	0.12095
61.437	0.10976	61.262	0.12089
61.437	0.10978	61.263	0.12084
61.438	0.1098	61.263	0.12079
61.438	0.10981	61.263	0.12073
61.438	0.10983	61.263	0.12067
61.438	0.10984	61.263	0.12061
61.438	0.10985	61.263	0.12056
61.438	0.10985	61.263	0.12051
61.438	0.10986	61.264	0.12047
61.439	0.10986	61.264	0.12042
61.439	0.10986	61.264	0.12037
61.439	0.10985	61.264	0.12031
61.439	0.10985	61.264	0.12026
61.439	0.10984	61.264	0.1202
61.439	0.10983	61.264	0.12014
61.440	0.10982	61.265	0.12008
61.440	0.1098	61.265	0.12001
61.440	0.10978	61.265	0.11995
61.440	0.10976	61.265	0.11988
61.440	0.10974	61.265	0.11981
61.440	0.10972	61.265	0.11974
61.440	0.10969	61.265	0.11967
61.441	0.10966	61.266	0.11959
61.441	0.10963	61.266	0.11952
61.441	0.10959	61.266	0.11944
61.441	0.10956	61.266	0.11936
61.441	0.1096	61.266	0.11927
61.441	0.10974	61.266	0.11919
61.441	0.10987	61.266	0.11911
61.442	0.11	61.266	0.11902
61.442	0.11013	61.267	0.11893
61.442	0.11025	61.267	0.11884
61.442	0.11037	61.267	0.11875
61.442	0.11049	61.267	0.11866
61.442	0.1106	61.267	0.11875
61.442	0.11071	61.267	0.11885
61.443	0.11081	61.267	0.11894
61.443	0.11091	61.268	0.11903
61.443	0.11101	61.268	0.11912
61.443	0.1111	61.268	0.11921
61.443	0.11119	61.268	0.1193
61.443	0.11128	61.268	0.11939
61.444	0.11136	61.268	0.11948
61.444	0.11144	61.268	0.11956
61.444	0.11151	61.269	0.11964
61.444	0.11158	61.269	0.11977
61.444	0.11165	61.269	0.1199
61.444	0.11171	61.269	0.12003
61.444	0.11177	61.269	0.12015
61.445	0.11182	61.269	0.12028
61.445	0.11188	61.269	0.1204
61.445	0.11192	61.270	0.12053

61.445	0.11197	61.270	0.12065
61.445	0.11201	61.270	0.12077
61.445	0.11204	61.270	0.1209
61.445	0.11207	61.270	0.12102
61.446	0.1121	61.270	0.12114
61.446	0.11213	61.270	0.12126
61.446	0.11215	61.270	0.12137
61.446	0.11217	61.271	0.12149
61.446	0.11218	61.271	0.12161
61.446	0.11219	61.271	0.12172
61.447	0.1122	61.271	0.12186
61.447	0.1122	61.271	0.12199
61.447	0.1122	61.271	0.12213
61.447	0.1122	61.271	0.12226
61.447	0.1122	61.272	0.12239
61.447	0.11219	61.272	0.12252
61.447	0.11217	61.272	0.12265
61.448	0.11217	61.272	0.12278
61.448	0.11216	61.272	0.12291
61.448	0.11216	61.272	0.12303
61.448	0.11215	61.272	0.12316
61.448	0.11214	61.273	0.12328
61.448	0.11212	61.273	0.1234
61.448	0.1121	61.273	0.12353
61.449	0.11208	61.273	0.12365
61.449	0.11205	61.273	0.12376
61.449	0.11202	61.273	0.12388
61.449	0.11198	61.273	0.124
61.449	0.11194	61.274	0.12411
61.449	0.1119	61.274	0.12423
61.449	0.11185	61.274	0.12434
61.450	0.1118	61.274	0.12445
61.450	0.11175	61.274	0.12456
61.450	0.11169	61.274	0.12467
61.450	0.11163	61.274	0.12478
61.450	0.11156	61.275	0.12489
61.450	0.1115	61.275	0.12499
61.451	0.11142	61.275	0.1251
61.451	0.11135	61.275	0.1252
61.451	0.11127	61.275	0.1253
61.451	0.11119	61.275	0.1254
61.451	0.1111	61.275	0.1255
61.451	0.11101	61.275	0.1256
61.451	0.11092	61.276	0.1257
61.452	0.11083	61.276	0.12579
61.452	0.11073	61.276	0.12589
61.452	0.11063	61.276	0.12598
61.452	0.11052	61.276	0.12607
61.452	0.11041	61.276	0.12616
61.452	0.1103	61.276	0.12625
61.452	0.11019	61.277	0.12634
61.453	0.11007	61.277	0.12643
61.453	0.10995	61.277	0.12652

61.453	0.10983	61.277	0.1266
61.453	0.1097	61.277	0.12668
61.453	0.10957	61.277	0.12677
61.453	0.10944	61.277	0.12685
61.454	0.10931	61.278	0.12693
61.454	0.10917	61.278	0.12701
61.454	0.10904	61.278	0.12709
61.454	0.10889	61.278	0.12716
61.454	0.10875	61.278	0.12724
61.454	0.10861	61.278	0.12731
61.454	0.10846	61.278	0.12739
61.455	0.10831	61.279	0.12746
61.455	0.10816	61.279	0.12753
61.455	0.108	61.279	0.1276
61.455	0.10784	61.279	0.12767
61.455	0.10769	61.279	0.12774
61.455	0.10753	61.279	0.12781
61.456	0.10737	61.279	0.12788
61.456	0.1072	61.280	0.12794
61.456	0.10704	61.280	0.128
61.456	0.10687	61.280	0.12807
61.456	0.1067	61.280	0.12813
61.456	0.10653	61.280	0.1282
61.456	0.10636	61.280	0.12826
61.457	0.10619	61.280	0.12832
61.457	0.10601	61.280	0.12838
61.457	0.10584	61.281	0.12844
61.457	0.10566	61.281	0.1285
61.457	0.10548	61.281	0.12855
61.457	0.1053	61.281	0.12861
61.457	0.10512	61.281	0.12866
61.458	0.10494	61.281	0.12871
61.458	0.10476	61.281	0.12877
61.458	0.10457	61.282	0.12882
61.458	0.10439	61.282	0.12886
61.458	0.10421	61.282	0.12891
61.458	0.10402	61.282	0.12896
61.459	0.10383	61.282	0.129
61.459	0.10365	61.282	0.12905
61.459	0.10346	61.282	0.12909
61.459	0.10327	61.283	0.12913
61.459	0.10309	61.283	0.12917
61.459	0.1029	61.283	0.12921
61.459	0.10271	61.283	0.12925
61.460	0.10255	61.283	0.12928
61.460	0.10241	61.283	0.12932
61.460	0.10228	61.283	0.12935
61.460	0.10215	61.284	0.12938
61.460	0.10201	61.284	0.12941
61.460	0.10187	61.284	0.12944
61.460	0.10173	61.284	0.12946
61.461	0.10159	61.284	0.12949
61.461	0.10145	61.284	0.12951

61.461	0.10148	61.284	0.12953
61.461	0.10161	61.285	0.12956
61.461	0.10174	61.285	0.12957
61.461	0.10187	61.285	0.12959
61.462	0.10199	61.285	0.12961
61.462	0.10212	61.285	0.12962
61.462	0.10224	61.285	0.12963
61.462	0.10235	61.285	0.12964
61.462	0.10247	61.286	0.12965
61.462	0.10258	61.286	0.12966
61.462	0.10269	61.286	0.12966
61.463	0.10279	61.286	0.12967
61.463	0.10289	61.286	0.12967
61.463	0.10299	61.286	0.12967
61.463	0.10308	61.286	0.12967
61.463	0.10317	61.287	0.12966
61.463	0.10326	61.287	0.12966
61.464	0.10334	61.287	0.12965
61.464	0.10341	61.287	0.12964
61.464	0.10349	61.287	0.12963
61.464	0.10356	61.287	0.12962
61.464	0.10362	61.287	0.1296
61.464	0.10369	61.288	0.12959
61.464	0.10374	61.288	0.12957
61.465	0.1038	61.288	0.12955
61.465	0.10385	61.288	0.12952
61.465	0.1039	61.288	0.1295
61.465	0.10394	61.288	0.12947
61.465	0.10398	61.288	0.12944
61.465	0.10401	61.289	0.12941
61.465	0.10404	61.289	0.12938
61.466	0.10407	61.289	0.12934
61.466	0.10409	61.289	0.12931
61.466	0.10411	61.289	0.12927
61.466	0.10413	61.289	0.12922
61.466	0.10414	61.289	0.12918
61.466	0.10415	61.290	0.12913
61.467	0.10416	61.290	0.12908
61.467	0.10416	61.290	0.12903
61.467	0.10416	61.290	0.12898
61.467	0.10415	61.290	0.12892
61.467	0.10415	61.290	0.12886
61.467	0.10414	61.290	0.1288
61.467	0.10412	61.291	0.12874
61.468	0.10411	61.291	0.12868
61.468	0.10409	61.291	0.12861
61.468	0.10407	61.291	0.12854
61.468	0.10404	61.291	0.12846
61.468	0.10402	61.291	0.12839
61.468	0.10405	61.291	0.12831
61.469	0.10408	61.292	0.12823
61.469	0.1041	61.292	0.12815
61.469	0.10413	61.292	0.12806

61.469	0.10414	61.292	0.12797
61.469	0.10416	61.292	0.12788
61.469	0.10417	61.292	0.12779
61.469	0.10418	61.292	0.1277
61.470	0.10419	61.293	0.1276
61.470	0.10419	61.293	0.1275
61.470	0.10419	61.293	0.12739
61.470	0.10419	61.293	0.12729
61.470	0.10419	61.293	0.12718
61.470	0.10418	61.293	0.12707
61.471	0.10417	61.293	0.12696
61.471	0.10415	61.294	0.12684
61.471	0.10413	61.294	0.12673
61.471	0.10411	61.294	0.12661
61.471	0.10409	61.294	0.12648
61.471	0.10406	61.294	0.12636
61.471	0.10403	61.294	0.12623
61.472	0.10399	61.294	0.1261
61.472	0.10396	61.295	0.12597
61.472	0.10392	61.295	0.12583
61.472	0.10387	61.295	0.1257
61.472	0.10383	61.295	0.12556
61.472	0.10378	61.295	0.12542
61.472	0.10372	61.295	0.12527
61.473	0.10367	61.295	0.12513
61.473	0.10361	61.296	0.12498
61.473	0.10354	61.296	0.12483
61.473	0.10348	61.296	0.12468
61.473	0.10341	61.296	0.12452
61.473	0.10334	61.296	0.12437
61.474	0.10326	61.296	0.12421
61.474	0.10318	61.296	0.12405
61.474	0.1031	61.297	0.12389
61.474	0.10302	61.297	0.12372
61.474	0.10293	61.297	0.12356
61.474	0.10284	61.297	0.12339
61.474	0.10275	61.297	0.12322
61.475	0.10266	61.297	0.12305
61.475	0.10256	61.297	0.12288
61.475	0.10246	61.298	0.1227
61.475	0.10236	61.298	0.12253
61.475	0.10225	61.298	0.12235
61.475	0.10214	61.298	0.12217
61.476	0.10203	61.298	0.12199
61.476	0.10192	61.298	0.12181
61.476	0.1018	61.298	0.12162
61.476	0.10169	61.299	0.12144
61.476	0.10157	61.299	0.12125
61.476	0.10145	61.299	0.12106
61.476	0.10132	61.299	0.12087
61.477	0.1012	61.299	0.12068
61.477	0.10107	61.299	0.12049
61.477	0.10094	61.299	0.1203

61.477	0.10081	61.300	0.12011
61.477	0.10068	61.300	0.11991
61.477	0.10054	61.300	0.11972
61.478	0.10041	61.300	0.11952
61.478	0.10027	61.300	0.11932
61.478	0.10013	61.300	0.11913
61.478	0.1	61.300	0.11893
61.478	0.09985	61.301	0.11873
61.478	0.09971	61.301	0.11853
61.478	0.09957	61.301	0.11832
61.479	0.09943	61.301	0.11812
61.479	0.09928	61.301	0.11792
61.479	0.09914	61.301	0.11771
61.479	0.09899	61.301	0.11751
61.479	0.09885	61.302	0.1173
61.479	0.0987	61.302	0.1171
61.480	0.09855	61.302	0.11689
61.480	0.0984	61.302	0.11668
61.480	0.09825	61.302	0.11648
61.480	0.0981	61.302	0.11627
61.480	0.09795	61.302	0.11606
61.480	0.09781	61.303	0.11585
61.480	0.09769	61.303	0.11564
61.481	0.09756	61.303	0.11543
61.481	0.09744	61.303	0.11522
61.481	0.09731	61.303	0.11501
61.481	0.09721	61.303	0.1148
61.481	0.09766	61.304	0.11459
61.481	0.09811	61.304	0.11438
61.482	0.09855	61.304	0.11417
61.482	0.09899	61.304	0.11396
61.482	0.09943	61.304	0.11374
61.482	0.09986	61.304	0.11354
61.482	0.10028	61.304	0.11334
61.482	0.1007	61.305	0.11315
61.482	0.10111	61.305	0.11295
61.483	0.10151	61.305	0.11275
61.483	0.10191	61.305	0.11255
61.483	0.10229	61.305	0.11235
61.483	0.10267	61.305	0.11215
61.483	0.10304	61.305	0.11194
61.483	0.10339	61.306	0.11174
61.483	0.10374	61.306	0.11154
61.484	0.10407	61.306	0.11134
61.484	0.1044	61.306	0.11114
61.484	0.10471	61.306	0.11093
61.484	0.10501	61.306	0.11073
61.484	0.1053	61.306	0.11053
61.484	0.10557	61.307	0.11033
61.485	0.10584	61.307	0.11024
61.485	0.1061	61.307	0.11026
61.485	0.10634	61.307	0.11028
61.485	0.10657	61.307	0.11029

61.485	0.10679	61.307	0.11031
61.485	0.10699	61.308	0.11032
61.485	0.10718	61.308	0.11033
61.486	0.10735	61.308	0.11034
61.486	0.10751	61.308	0.11034
61.486	0.10766	61.308	0.11035
61.486	0.10779	61.308	0.11035
61.486	0.10791	61.308	0.11035
61.486	0.10802	61.309	0.11035
61.487	0.10811	61.309	0.11035
61.487	0.10819	61.309	0.11034
61.487	0.10826	61.309	0.11033
61.487	0.10832	61.309	0.11033
61.487	0.10837	61.309	0.11032
61.487	0.10841	61.309	0.1103
61.487	0.10844	61.310	0.11029
61.488	0.10845	61.310	0.11027
61.488	0.10846	61.310	0.11026
61.488	0.10846	61.310	0.11024
61.488	0.10844	61.310	0.11021
61.488	0.10842	61.310	0.11019
61.488	0.10839	61.310	0.11016
61.489	0.10835	61.311	0.11014
61.489	0.1083	61.311	0.11011
61.489	0.10825	61.311	0.11008
61.489	0.10819	61.311	0.11004
61.489	0.10812	61.311	0.11001
61.489	0.10804	61.311	0.10997
61.490	0.10795	61.312	0.10993
61.490	0.10786	61.312	0.10989
61.490	0.10777	61.312	0.10985
61.490	0.10766	61.312	0.10981
61.490	0.10756	61.312	0.10976
61.490	0.10745	61.312	0.10972
61.490	0.10733	61.312	0.10967
61.491	0.10721	61.313	0.10962
61.491	0.10708	61.313	0.10957
61.491	0.10696	61.313	0.10951
61.491	0.10684	61.313	0.10946
61.491	0.10671	61.313	0.1094
61.491	0.10657	61.313	0.10934
61.492	0.10644	61.313	0.10928
61.492	0.1063	61.314	0.10922
61.492	0.10616	61.314	0.10916
61.492	0.10602	61.314	0.10909
61.492	0.10588	61.314	0.10905
61.492	0.10573	61.314	0.109
61.492	0.10558	61.314	0.10895
61.493	0.10543	61.315	0.1089
61.493	0.10528	61.315	0.10885
61.493	0.10513	61.315	0.1088
61.493	0.10498	61.315	0.10875
61.493	0.10483	61.315	0.10869

61.493	0.10468	61.315	0.10864
61.494	0.10453	61.315	0.10858
61.494	0.10437	61.316	0.10852
61.494	0.10422	61.316	0.10846
61.494	0.10407	61.316	0.1084
61.494	0.10391	61.316	0.10834
61.494	0.10376	61.316	0.10827
61.495	0.1036	61.316	0.10821
61.495	0.10345	61.316	0.10814
61.495	0.10329	61.317	0.10807
61.495	0.10314	61.317	0.108
61.495	0.10308	61.317	0.10793
61.495	0.10305	61.317	0.10786
61.495	0.10303	61.317	0.10778
61.496	0.10303	61.317	0.10771
61.496	0.10303	61.318	0.10763
61.496	0.10304	61.318	0.10755
61.496	0.10307	61.318	0.10747
61.496	0.10312	61.318	0.10739
61.496	0.10316	61.318	0.1073
61.497	0.10322	61.318	0.10722
61.497	0.10329	61.318	0.10713
61.497	0.10338	61.319	0.10704
61.497	0.10347	61.319	0.10695
61.497	0.10359	61.319	0.10686
61.497	0.10371	61.319	0.10677
61.498	0.10384	61.319	0.10668
61.498	0.10399	61.319	0.10658
61.498	0.10414	61.319	0.10649
61.498	0.10432	61.320	0.10639
61.498	0.10449	61.320	0.10629
61.498	0.10469	61.320	0.10619
61.498	0.10489	61.320	0.10608
61.499	0.10511	61.320	0.10598
61.499	0.10534	61.320	0.10588
61.499	0.10557	61.321	0.10577
61.499	0.10581	61.321	0.10566
61.499	0.10607	61.321	0.10555
61.499	0.10633	61.321	0.10544
61.500	0.1066	61.321	0.10533
61.500	0.10688	61.321	0.10521
61.500	0.10716	61.321	0.1051
61.500	0.10745	61.322	0.10498
61.500	0.10776	61.322	0.10487
61.500	0.10807	61.322	0.10475
61.501	0.10839	61.322	0.10463
61.501	0.10871	61.322	0.10452
61.501	0.10905	61.322	0.10441
61.501	0.10939	61.323	0.1043
61.501	0.10973	61.323	0.10419
61.501	0.11007	61.323	0.10408
61.501	0.11041	61.323	0.10396
61.502	0.11075	61.323	0.10384

61.502	0.11108	61.323	0.10373
61.502	0.11142	61.323	0.10361
61.502	0.11175	61.324	0.10348
61.502	0.11208	61.324	0.10336
61.502	0.1124	61.324	0.10324
61.503	0.11272	61.324	0.1032
61.503	0.11304	61.324	0.10319
61.503	0.11336	61.324	0.10318
61.503	0.11366	61.324	0.10316
61.503	0.11397	61.325	0.10315
61.503	0.11427	61.325	0.10313
61.504	0.11456	61.325	0.10312
61.504	0.11485	61.325	0.1031
61.504	0.11514	61.325	0.10308
61.504	0.11541	61.325	0.10307
61.504	0.11568	61.326	0.10305
61.504	0.11594	61.326	0.10303
61.505	0.1162	61.326	0.10301
61.505	0.11645	61.326	0.10298
61.505	0.1167	61.326	0.10296
61.505	0.11693	61.326	0.10294
61.505	0.11716	61.326	0.10291
61.505	0.11738	61.327	0.10289
61.505	0.11759	61.327	0.10286
61.506	0.11779	61.327	0.10284
61.506	0.11798	61.327	0.10281
61.506	0.11816	61.327	0.10278
61.506	0.11833	61.327	0.10275
61.506	0.11849	61.328	0.10272
61.506	0.11864	61.328	0.10269
61.507	0.11878	61.328	0.10266
61.507	0.11891	61.328	0.10263
61.507	0.11903	61.328	0.1026
61.507	0.11914	61.328	0.10256
61.507	0.11923	61.328	0.10253
61.507	0.11932	61.329	0.10249
61.508	0.1194	61.329	0.10246
61.508	0.11946	61.329	0.10242
61.508	0.11952	61.329	0.10239
61.508	0.11967	61.329	0.10235
61.508	0.11986	61.329	0.10231
61.508	0.12005	61.330	0.10227
61.509	0.12022	61.330	0.10223
61.509	0.12038	61.330	0.10219
61.509	0.12053	61.330	0.10215
61.509	0.12067	61.330	0.1021
61.509	0.12079	61.330	0.10206
61.509	0.1209	61.330	0.10202
61.510	0.121	61.331	0.10197
61.510	0.12109	61.331	0.10192
61.510	0.12116	61.331	0.10188
61.510	0.12122	61.331	0.10183
61.510	0.12127	61.331	0.10178

61.510	0.1213	61.331	0.10173
61.511	0.12132	61.332	0.10168
61.511	0.12133	61.332	0.10163
61.511	0.12133	61.332	0.10158
61.511	0.12131	61.332	0.10153
61.511	0.12128	61.332	0.10147
61.511	0.12124	61.332	0.10142
61.511	0.12119	61.332	0.10136
61.512	0.12113	61.333	0.10131
61.512	0.12107	61.333	0.10125
61.512	0.12101	61.333	0.1012
61.512	0.12094	61.333	0.10114
61.512	0.12088	61.333	0.10108
61.512	0.12081	61.333	0.10102
61.513	0.12074	61.333	0.10097
61.513	0.12067	61.334	0.10091
61.513	0.12058	61.334	0.10085
61.513	0.12049	61.334	0.10078
61.513	0.12039	61.334	0.10072
61.513	0.12028	61.334	0.10066
61.514	0.12017	61.334	0.10059
61.514	0.12005	61.335	0.10053
61.514	0.11992	61.335	0.10046
61.514	0.11978	61.335	0.1004
61.514	0.11964	61.335	0.10033
61.514	0.11949	61.335	0.10026
61.515	0.11933	61.335	0.10019
61.515	0.11917	61.335	0.10012
61.515	0.11901	61.336	0.10005
61.515	0.11883	61.336	0.09997
61.515	0.11865	61.336	0.0999
61.515	0.11846	61.336	0.09983
61.516	0.11827	61.336	0.09976
61.516	0.11807	61.336	0.09968
61.516	0.11787	61.337	0.09961
61.516	0.11766	61.337	0.09953
61.516	0.11745	61.337	0.09946
61.516	0.11723	61.337	0.09938
61.517	0.117	61.337	0.0993
61.517	0.11677	61.337	0.09922
61.517	0.11654	61.337	0.09914
61.517	0.1163	61.338	0.09906
61.517	0.11605	61.338	0.09898
61.517	0.11581	61.338	0.0989
61.518	0.11555	61.338	0.09882
61.518	0.11529	61.338	0.09874
61.518	0.11503	61.338	0.09865
61.518	0.11477	61.339	0.09857
61.518	0.11449	61.339	0.09848
61.518	0.11422	61.339	0.0984
61.519	0.11394	61.339	0.09831
61.519	0.11366	61.339	0.09822
61.519	0.11338	61.339	0.09813

61.519	0.11309	61.339	0.09804
61.519	0.11279	61.340	0.09795
61.519	0.1125	61.340	0.09786
61.520	0.1122	61.340	0.09777
61.520	0.1119	61.340	0.09767
61.520	0.1116	61.340	0.09758
61.520	0.11129	61.340	0.09748
61.520	0.11098	61.341	0.09738
61.520	0.11068	61.341	0.09729
61.521	0.11037	61.341	0.09719
61.521	0.11006	61.341	0.09709
61.521	0.10975	61.341	0.09698
61.521	0.10943	61.341	0.09688
61.521	0.10912	61.341	0.09678
61.521	0.1088	61.342	0.09667
61.522	0.10848	61.342	0.09657
61.522	0.10816	61.342	0.09646
61.522	0.10784	61.342	0.09635
61.522	0.10751	61.342	0.09624
61.522	0.10719	61.342	0.09613
61.522	0.10686	61.343	0.09602
61.523	0.10653	61.343	0.09591
61.523	0.1062	61.343	0.0958
61.523	0.10588	61.343	0.09568
61.523	0.10555	61.343	0.09557
61.523	0.10524	61.343	0.09545
61.523	0.10499	61.344	0.09533
61.524	0.10476	61.344	0.09522
61.524	0.10455	61.344	0.0951
61.524	0.10435	61.344	0.09498
61.524	0.10451	61.344	0.09485
61.524	0.10518	61.344	0.09473
61.524	0.10464	61.344	0.09461
61.525	0.10373	61.345	0.09448
61.525	0.1036	61.345	0.09436
61.525	0.10347	61.345	0.09423
61.525	0.10335	61.345	0.0941
61.525	0.10354	61.345	0.09397
61.525	0.10377	61.345	0.09384
61.526	0.10359	61.346	0.09371
61.526	0.10317	61.346	0.09359
61.526	0.10298	61.346	0.09356
61.526	0.10293	61.346	0.09353
61.526	0.10288	61.346	0.0935
61.526	0.10283	61.346	0.09346
61.527	0.10279	61.346	0.09343
61.527	0.10276	61.347	0.09339
61.527	0.10273	61.347	0.09335
61.527	0.10276	61.347	0.09331
61.527	0.1028	61.347	0.09327
61.527	0.10286	61.347	0.09322
61.528	0.10294	61.347	0.09317
61.528	0.10302	61.348	0.09312

61.528	0.1031	61.348	0.09307
61.528	0.10318	61.348	0.09302
61.528	0.10326	61.348	0.09297
61.528	0.10333	61.348	0.09291
61.529	0.1034	61.348	0.09285
61.529	0.10347	61.348	0.09279
61.529	0.10353	61.349	0.09273
61.529	0.10359	61.349	0.09267
61.529	0.10365	61.349	0.0926
61.529	0.1037	61.349	0.09254
61.530	0.10374	61.349	0.09247
61.530	0.10378	61.349	0.0924
61.530	0.10381	61.350	0.09232
61.530	0.10384	61.350	0.09225
61.530	0.10386	61.350	0.09217
61.530	0.10387	61.350	0.09209
61.531	0.10388	61.350	0.09201
61.531	0.10388	61.350	0.09193
61.531	0.10387	61.351	0.09185
61.531	0.10385	61.351	0.09176
61.531	0.10383	61.351	0.09167
61.531	0.1038	61.351	0.09158
61.532	0.10375	61.351	0.09149
61.532	0.10371	61.351	0.0914
61.532	0.10365	61.351	0.09131
61.532	0.10358	61.352	0.09121
61.532	0.10351	61.352	0.09111
61.532	0.10342	61.352	0.09101
61.533	0.10333	61.352	0.09091
61.533	0.10323	61.352	0.09081
61.533	0.10312	61.352	0.09071
61.533	0.103	61.353	0.0906
61.533	0.10287	61.353	0.09049
61.533	0.10274	61.353	0.09038
61.534	0.10259	61.353	0.09027
61.534	0.10244	61.353	0.09016
61.534	0.10228	61.353	0.09005
61.534	0.10211	61.353	0.08993
61.534	0.10194	61.354	0.08982
61.534	0.10176	61.354	0.0897
61.535	0.10158	61.354	0.08958
61.535	0.10138	61.354	0.08945
61.535	0.10118	61.354	0.08933
61.535	0.10098	61.354	0.0892
61.535	0.10077	61.355	0.08908
61.536	0.10055	61.355	0.08895
61.536	0.10032	61.355	0.08882
61.536	0.10009	61.355	0.08868
61.536	0.09985	61.355	0.08855
61.536	0.09961	61.355	0.08842
61.536	0.09937	61.356	0.08828
61.537	0.09911	61.356	0.08814
61.537	0.09886	61.356	0.088

61.537	0.0986	61.356	0.08786
61.537	0.09833	61.356	0.08772
61.537	0.09806	61.356	0.08757
61.537	0.09779	61.356	0.08743
61.538	0.09752	61.357	0.08728
61.538	0.09724	61.357	0.08714
61.538	0.09696	61.357	0.08699
61.538	0.09667	61.357	0.08684
61.538	0.0964	61.357	0.08669
61.538	0.09614	61.357	0.08654
61.539	0.09588	61.358	0.08639
61.539	0.09563	61.358	0.08623
61.539	0.09538	61.358	0.08608
61.539	0.09513	61.358	0.08593
61.539	0.09489	61.358	0.08577
61.539	0.09476	61.358	0.08561
61.540	0.09477	61.358	0.08546
61.540	0.09478	61.359	0.0853
61.540	0.09479	61.359	0.08514
61.540	0.09479	61.359	0.08498
61.540	0.0948	61.359	0.08483
61.540	0.0948	61.359	0.08467
61.541	0.0948	61.359	0.08451
61.541	0.0948	61.360	0.08434
61.541	0.0948	61.360	0.08418
61.541	0.0948	61.360	0.08402
61.541	0.09479	61.360	0.08386
61.542	0.09479	61.360	0.08369
61.542	0.09478	61.360	0.08353
61.542	0.09478	61.361	0.08337
61.542	0.09477	61.361	0.0832
61.542	0.09476	61.361	0.08304
61.542	0.09475	61.361	0.08288
61.543	0.09474	61.361	0.0835
61.543	0.09473	61.361	0.08333
61.543	0.09472	61.361	0.08238
61.543	0.09471	61.362	0.08222
61.543	0.09469	61.362	0.08205
61.543	0.09468	61.362	0.08216
61.544	0.09466	61.362	0.08228
61.544	0.09465	61.362	0.08253
61.544	0.09463	61.362	0.0819
61.544	0.09461	61.363	0.08123
61.544	0.0946	61.363	0.08113
61.544	0.09458	61.363	0.08279
61.545	0.09456	61.363	0.08256
61.545	0.09454	61.363	0.08142
61.545	0.09452	61.363	0.08175
61.545	0.0945	61.364	0.08151
61.545	0.09448	61.364	0.08087
61.546	0.09446	61.364	0.08082
61.546	0.09444	61.364	0.0808
61.546	0.09442	61.364	0.08193

61.546	0.0944	61.364	0.08194
61.546	0.09438	61.365	0.08102
61.546	0.09436	61.365	0.08078
61.547	0.09434	61.365	0.08077
61.547	0.09436	61.365	0.08076
61.547	0.09439	61.365	0.08075
61.547	0.09442	61.365	0.08172
61.547	0.09444	61.365	0.08155
61.547	0.09447	61.366	0.08073
61.548	0.09449	61.366	0.08072
61.548	0.09451	61.366	0.08095
61.548	0.09454	61.366	0.08071
61.548	0.09456	61.366	0.0807
61.548	0.09458	61.366	0.08079
61.548	0.0946	61.367	0.08087
61.549	0.09461	61.367	0.08068
61.549	0.09463	61.367	0.08113
61.549	0.09465	61.367	0.08083
61.549	0.09466	61.367	0.08064
61.549	0.09467	61.367	0.08063
61.550	0.09468	61.368	0.08061
61.550	0.0947	61.368	0.0806
61.550	0.09471	61.368	0.08059
61.550	0.09471	61.368	0.08057
61.550	0.09472	61.368	0.08056
61.550	0.09473	61.368	0.08054
61.551	0.09473	61.368	0.08053
61.551	0.09474	61.369	0.08052
61.551	0.09474	61.369	0.0805
61.551	0.09474	61.369	0.08049
61.551	0.09474	61.369	0.08048
61.551	0.09474	61.369	0.08046
61.552	0.09474	61.369	0.08045
61.552	0.09474	61.370	0.08043
61.552	0.09473	61.370	0.08041
61.552	0.09473	61.370	0.08039
61.552	0.09473	61.370	0.08037
61.553	0.09472	61.370	0.08035
61.553	0.09472	61.370	0.08033
61.553	0.09472	61.371	0.0803
61.553	0.09471	61.371	0.08027
61.553	0.0947	61.371	0.08025
61.553	0.09469	61.371	0.08022
61.554	0.09468	61.371	0.08019
61.554	0.09467	61.371	0.08016
61.554	0.09466	61.372	0.08013
61.554	0.09464	61.372	0.0801
61.554	0.09462	61.372	0.08007
61.554	0.09461	61.372	0.08003
61.555	0.09459	61.372	0.08
61.555	0.09456	61.372	0.07997
61.555	0.09454	61.372	0.07994
61.555	0.09452	61.373	0.07991

61.555	0.09449	61.373	0.07988
61.556	0.09446	61.373	0.07984
61.556	0.09444	61.373	0.07981
61.556	0.09441	61.373	0.07978
61.556	0.09437	61.373	0.07975
61.556	0.09434	61.374	0.07971
61.556	0.09431	61.374	0.07968
61.557	0.09427	61.374	0.07964
61.557	0.09424	61.374	0.0796
61.557	0.0942	61.374	0.07956
61.557	0.09416	61.374	0.07952
61.557	0.09412	61.375	0.07948
61.557	0.09407	61.375	0.07944
61.558	0.09403	61.375	0.07939
61.558	0.09398	61.375	0.07935
61.558	0.09393	61.375	0.07931
61.558	0.09388	61.375	0.07926
61.558	0.09383	61.376	0.07922
61.559	0.09378	61.376	0.07917
61.559	0.09372	61.376	0.07913
61.559	0.09367	61.376	0.07908
61.559	0.09361	61.376	0.07903
61.559	0.09355	61.376	0.07899
61.559	0.09349	61.377	0.07894
61.560	0.09343	61.377	0.07889
61.560	0.09336	61.377	0.07884
61.560	0.0933	61.377	0.07879
61.560	0.09323	61.377	0.07874
61.560	0.09316	61.377	0.07869
61.561	0.09309	61.378	0.07864
61.561	0.09302	61.378	0.07858
61.561	0.09294	61.378	0.07853
61.561	0.09287	61.378	0.07847
61.561	0.09279	61.378	0.07841
61.561	0.09272	61.378	0.07836
61.562	0.09265	61.378	0.0783
61.562	0.09257	61.379	0.07824
61.562	0.0925	61.379	0.07818
61.562	0.09242	61.379	0.07812
61.562	0.09234	61.379	0.07806
61.563	0.09226	61.379	0.078
61.563	0.09218	61.379	0.07795
61.563	0.0921	61.380	0.07789
61.563	0.09202	61.380	0.07783
61.563	0.09193	61.380	0.07777
61.563	0.09185	61.380	0.07771
61.564	0.09257	61.380	0.07764
61.564	0.09201	61.380	0.07758
61.564	0.09158	61.381	0.07752
61.564	0.09149	61.381	0.07745
61.564	0.0914	61.381	0.07738
61.565	0.0913	61.381	0.07731
61.565	0.09121	61.381	0.07724

61.565	0.09111	61.381	0.07717
61.565	0.09101	61.382	0.07709
61.565	0.09101	61.382	0.07702
61.565	0.09123	61.382	0.07694
61.566	0.09088	61.382	0.07687
61.566	0.09087	61.382	0.07679
61.566	0.09087	61.382	0.07672
61.566	0.09086	61.383	0.07664
61.566	0.09084	61.383	0.07657
61.567	0.09173	61.383	0.07649
61.567	0.09113	61.383	0.07642
61.567	0.09111	61.383	0.07634
61.567	0.0909	61.383	0.07627
61.567	0.09073	61.384	0.07619
61.567	0.09079	61.384	0.07612
61.568	0.09068	61.384	0.07604
61.568	0.09066	61.384	0.07596
61.568	0.09063	61.384	0.07588
61.568	0.09059	61.384	0.0758
61.568	0.09056	61.385	0.07572
61.569	0.0906	61.385	0.07564
61.569	0.09267	61.385	0.07555
61.569	0.092	61.385	0.07547
61.569	0.09048	61.385	0.07538
61.569	0.09036	61.385	0.0753
61.569	0.09031	61.386	0.07521
61.570	0.09027	61.386	0.07512
61.570	0.09022	61.386	0.07503
61.570	0.09017	61.386	0.07494
61.570	0.09029	61.386	0.07485
61.570	0.09007	61.387	0.07475
61.571	0.09002	61.387	0.07466
61.571	0.08997	61.387	0.07457
61.571	0.08992	61.387	0.07447
61.571	0.08986	61.387	0.07438
61.571	0.09025	61.387	0.07429
61.571	0.09	61.388	0.0742
61.572	0.08969	61.388	0.07411
61.572	0.08963	61.388	0.07402
61.572	0.08957	61.388	0.07393
61.572	0.08949	61.388	0.07393
61.572	0.08951	61.388	0.07398
61.573	0.09147	61.389	0.07403
61.573	0.0908	61.389	0.07428
61.573	0.08931	61.389	0.07412
61.573	0.08926	61.389	0.07416
61.573	0.08905	61.389	0.07419
61.573	0.08897	61.389	0.07423
61.574	0.0889	61.390	0.07426
61.574	0.08882	61.390	0.0743
61.574	0.08876	61.390	0.07434
61.574	0.08911	61.390	0.07438
61.574	0.09063	61.390	0.07442

61.575	0.0899	61.390	0.07447
61.575	0.08893	61.391	0.07462
61.575	0.08861	61.391	0.07455
61.575	0.08824	61.391	0.0746
61.575	0.08813	61.391	0.07464
61.576	0.08803	61.391	0.07467
61.576	0.08794	61.392	0.07471
61.576	0.08784	61.392	0.07475
61.576	0.08898	61.392	0.07479
61.576	0.08848	61.392	0.07483
61.576	0.08754	61.392	0.07486
61.577	0.08781	61.392	0.0749
61.577	0.08748	61.393	0.07493
61.577	0.08982	61.393	0.07496
61.577	0.08961	61.393	0.07499
61.577	0.08839	61.393	0.07502
61.578	0.08754	61.393	0.07504
61.578	0.08795	61.393	0.07507
61.578	0.08726	61.394	0.07509
61.578	0.08696	61.394	0.07511
61.578	0.08691	61.394	0.07514
61.578	0.08688	61.394	0.07517
61.579	0.0877	61.394	0.0752
61.579	0.08701	61.395	0.07524
61.579	0.08679	61.395	0.07529
61.579	0.08675	61.395	0.07532
61.579	0.08672	61.395	0.07534
61.580	0.08669	61.395	0.07537
61.580	0.08666	61.395	0.07539
61.580	0.08662	61.396	0.07542
61.580	0.08659	61.396	0.07545
61.580	0.08655	61.396	0.07548
61.581	0.08689	61.396	0.07551
61.581	0.08775	61.396	0.07553
61.581	0.08823	61.397	0.07555
61.581	0.08754	61.397	0.07558
61.581	0.0865	61.397	0.0756
61.581	0.08652	61.397	0.07562
61.582	0.08654	61.397	0.07565
61.582	0.08656	61.397	0.07589
61.582	0.08832	61.398	0.07582
61.582	0.08813	61.398	0.07572
61.582	0.08753	61.398	0.07575
61.583	0.08942	61.398	0.07577
61.583	0.08929	61.398	0.07579
61.583	0.08873	61.399	0.07582
61.583	0.08881	61.399	0.07584
61.583	0.08812	61.399	0.07586
61.583	0.08664	61.399	0.07588
61.584	0.08664	61.399	0.0759
61.584	0.08664	61.399	0.07593
61.584	0.08664	61.400	0.07596
61.584	0.08664	61.400	0.07598

61.584	0.08663	61.400	0.07601
61.585	0.08663	61.400	0.07603
61.585	0.08662	61.400	0.07606
61.585	0.08661	61.401	0.07609
61.585	0.0866	61.401	0.07612
61.585	0.08659	61.401	0.07614
61.585	0.08657	61.401	0.07616
61.586	0.08656	61.401	0.07618
61.586	0.08703	61.402	0.07618
61.586	0.08652	61.402	0.07619
61.586	0.0865	61.402	0.0762
61.586	0.08763	61.402	0.0762
61.587	0.09022	61.402	0.07621
61.587	0.08945	61.402	0.07621
61.587	0.08756	61.403	0.07621
61.587	0.08639	61.403	0.07621
61.587	0.08636	61.403	0.07621
61.588	0.08633	61.403	0.0762
61.588	0.0863	61.403	0.07671
61.588	0.08627	61.404	0.07618
61.588	0.08624	61.404	0.07617
61.588	0.0862	61.404	0.07615
61.588	0.08617	61.404	0.07614
61.589	0.08719	61.404	0.07613
61.589	0.08993	61.405	0.07611
61.589	0.08924	61.405	0.07608
61.589	0.08866	61.405	0.07605
61.589	0.08795	61.405	0.07603
61.590	0.08624	61.405	0.076
61.590	0.08591	61.406	0.07597
61.590	0.08587	61.406	0.0771
61.590	0.08583	61.406	0.07648
61.590	0.08641	61.406	0.07589
61.590	0.0872	61.406	0.07687
61.591	0.08662	61.407	0.07584
61.591	0.0857	61.407	0.07603
61.591	0.08697	61.407	0.07578
61.591	0.08627	61.407	0.07575
61.591	0.08552	61.407	0.07571
61.592	0.08548	61.407	0.07632
61.592	0.08543	61.408	0.07562
61.592	0.08539	61.408	0.07636
61.592	0.08534	61.408	0.07683
61.592	0.0853	61.408	0.07598
61.593	0.08526	61.408	0.07708
61.593	0.08522	61.409	0.07608
61.593	0.08572	61.409	0.07532
61.593	0.08513	61.409	0.07605
61.593	0.08926	61.409	0.07522
61.593	0.08973	61.409	0.07516
61.594	0.08717	61.410	0.07511
61.594	0.08496	61.410	0.07611
61.594	0.08493	61.410	0.07501

61.594	0.0849	61.410	0.07495
61.594	0.08487	61.410	0.0749
61.595	0.08484	61.411	0.077
61.595	0.08574	61.411	0.07585
61.595	0.08833	61.411	0.07776
61.595	0.08762	61.411	0.07619
61.595	0.08607	61.411	0.07619
61.595	0.08535	61.412	0.07456
61.596	0.0868	61.412	0.07635
61.596	0.08629	61.412	0.07733
61.596	0.08678	61.412	0.07624
61.596	0.08894	61.412	0.07757
61.596	0.08783	61.413	0.07662
61.597	0.08447	61.413	0.07696
61.597	0.08638	61.413	0.07692
61.597	0.08761	61.413	0.07642
61.597	0.08628	61.413	0.07833
61.597	0.08474	61.414	0.07672
61.597	0.0844	61.414	0.07625
61.598	0.08424	61.414	0.07562
61.598	0.08581	61.414	0.07407
61.598	0.08453	61.414	0.07621
61.598	0.08934	61.415	0.07667
61.598	0.08979	61.415	0.07457
61.599	0.08741	61.415	0.07497
61.599	0.08674	61.415	0.07554
61.599	0.08508	61.415	0.0768
61.599	0.08399	61.416	0.07511
61.599	0.08397	61.416	0.07391
61.600	0.08396	61.416	0.0768
61.600	0.08408	61.416	0.07861
61.600	0.08605	61.416	0.07694
61.600	0.08542	61.417	0.07582
61.600	0.08393	61.417	0.07609
61.600	0.08499	61.417	0.07633
61.601	0.0844	61.417	0.07397
61.601	0.08385	61.418	0.07658
61.601	0.08383	61.418	0.07742
61.601	0.08382	61.418	0.0751
61.601	0.0838	61.418	0.07398
61.602	0.08379	61.418	0.07447
61.602	0.08377	61.419	0.07579
61.602	0.08378	61.419	0.0752
61.602	0.08374	61.419	0.07715
61.602	0.08372	61.419	0.0788
61.602	0.0837	61.419	0.07726
61.603	0.08369	61.420	0.07624
61.603	0.08367	61.420	0.07693
61.603	0.08365	61.420	0.07393
61.603	0.08363	61.420	0.07609
61.603	0.08361	61.420	0.07742
61.604	0.08359	61.421	0.07534
61.604	0.08358	61.421	0.07578

61.604	0.08356	61.421	0.07504
61.604	0.08354	61.421	0.07797
61.604	0.08352	61.422	0.07729
61.604	0.08433	61.422	0.07676
61.605	0.08378	61.422	0.07501
61.605	0.08346	61.422	0.07716
61.605	0.08344	61.422	0.07865
61.605	0.08341	61.423	0.07667
61.605	0.08339	61.423	0.07579
61.606	0.08337	61.423	0.07668
61.606	0.08334	61.423	0.07371
61.606	0.08332	61.424	0.07843
61.606	0.08329	61.424	0.07534
61.606	0.08326	61.424	0.07722
61.606	0.08323	61.424	0.07582
61.607	0.0832	61.424	0.07517
61.607	0.08317	61.425	0.07534
61.607	0.08314	61.425	0.07538
61.607	0.08311	61.425	0.07324
61.607	0.08307	61.425	0.0732
61.608	0.08304	61.425	0.07458
61.608	0.083	61.426	0.07533
61.608	0.08297	61.426	0.07307
61.608	0.08294	61.426	0.07302
61.608	0.08501	61.426	0.07666
61.608	0.08297	61.427	0.07805
61.609	0.08521	61.427	0.07603
61.609	0.08514	61.427	0.07417
61.609	0.08455	61.427	0.07333
61.609	0.08533	61.427	0.07304
61.609	0.08339	61.428	0.07287
61.610	0.08259	61.428	0.07272
61.610	0.08255	61.428	0.0727
61.610	0.0825	61.428	0.07263
61.610	0.08245	61.429	0.07407
61.610	0.0824	61.429	0.07532
61.610	0.08235	61.429	0.07357
61.611	0.08299	61.429	0.07278
61.611	0.08224	61.429	0.07286
61.611	0.08254	61.430	0.07376
61.611	0.08235	61.430	0.07232
61.611	0.08207	61.430	0.07217
61.611	0.08201	61.430	0.07212
61.612	0.08196	61.431	0.07207
61.612	0.0819	61.431	0.07202
61.612	0.08184	61.431	0.07197
61.612	0.08178	61.431	0.07192
61.612	0.08176	61.431	0.07187
61.613	0.08175	61.432	0.07509
61.613	0.08173	61.432	0.0765
61.613	0.08171	61.432	0.07171
61.613	0.0817	61.432	0.07641
61.613	0.08168	61.433	0.0716

61.613	0.08166	61.433	0.07749
61.614	0.08164	61.433	0.07149
61.614	0.08162	61.433	0.07611
61.614	0.08159	61.433	0.07503
61.614	0.08157	61.434	0.07556
61.614	0.08154	61.434	0.07347
61.615	0.08152	61.434	0.07162
61.615	0.08149	61.434	0.07221
61.615	0.08146	61.435	0.07123
61.615	0.08143	61.435	0.07133
61.615	0.0814	61.435	0.07124
61.615	0.08137	61.435	0.07189
61.616	0.08133	61.435	0.07247
61.616	0.0813	61.436	0.07176
61.616	0.08126	61.436	0.07177
61.616	0.08122	61.436	0.07406
61.616	0.0812	61.436	0.07565
61.617	0.0812	61.437	0.07403
61.617	0.08119	61.437	0.07189
61.617	0.08118	61.437	0.07157
61.617	0.08116	61.437	0.07157
61.617	0.08115	61.438	0.07463
61.617	0.08114	61.438	0.07579
61.618	0.08112	61.438	0.07344
61.618	0.0811	61.438	0.0718
61.618	0.08108	61.438	0.07466
61.618	0.08106	61.439	0.07428
61.618	0.08104	61.439	0.07747
61.618	0.08102	61.439	0.07344
61.619	0.08099	61.439	0.07545
61.619	0.08097	61.440	0.07541
61.619	0.08094	61.440	0.07325
61.619	0.08092	61.440	0.07153
61.619	0.08089	61.440	0.0729
61.620	0.08086	61.441	0.07394
61.620	0.08158	61.441	0.07497
61.620	0.0808	61.441	0.07335
61.620	0.08183	61.441	0.07156
61.620	0.08073	61.441	0.07156
61.620	0.08222	61.442	0.07157
61.621	0.08218	61.442	0.07157
61.621	0.08064	61.442	0.07158
61.621	0.08059	61.442	0.07163
61.621	0.08056	61.443	0.07331
61.621	0.08052	61.443	0.07424
61.621	0.08048	61.443	0.07203
61.622	0.08044	61.443	0.07178
61.622	0.0804	61.444	0.07189
61.622	0.08036	61.444	0.07213
61.622	0.08032	61.444	0.07198
61.622	0.08028	61.444	0.0716
61.623	0.08023	61.445	0.07161
61.623	0.08019	61.445	0.07385

61.623	0.08076	61.445	0.07488
61.623	0.08038	61.445	0.07305
61.623	0.08006	61.445	0.074
61.623	0.08002	61.446	0.07276
61.624	0.08071	61.446	0.07329
61.624	0.0808	61.446	0.07243
61.624	0.08029	61.446	0.07366
61.624	0.08118	61.447	0.07329
61.624	0.08107	61.447	0.07545
61.625	0.07974	61.447	0.07184
61.625	0.0797	61.447	0.07439
61.625	0.08008	61.448	0.07321
61.625	0.07962	61.448	0.07168
61.625	0.08008	61.448	0.07361
61.625	0.07951	61.448	0.0741
61.626	0.07946	61.448	0.07364
61.626	0.07942	61.449	0.07166
61.626	0.07937	61.449	0.07267
61.626	0.07932	61.449	0.07173
61.626	0.07927	61.449	0.07178
61.626	0.08018	61.450	0.07196
61.627	0.07949	61.450	0.0722
61.627	0.07914	61.450	0.07224
61.627	0.0791	61.450	0.0721
61.627	0.07944	61.451	0.07315
61.627	0.07918	61.451	0.07206
61.628	0.079	61.451	0.07212
61.628	0.07896	61.451	0.07217
61.628	0.07921	61.452	0.07223
61.628	0.0789	61.452	0.07287
61.628	0.07887	61.452	0.07234
61.628	0.07884	61.452	0.07239
61.629	0.07881	61.453	0.07245
61.629	0.07879	61.453	0.0725
61.629	0.07885	61.453	0.07255
61.629	0.07873	61.453	0.0726
61.629	0.07893	61.453	0.07266
61.630	0.08042	61.454	0.07271
61.630	0.07877	61.454	0.07276
61.630	0.08071	61.454	0.07281
61.630	0.07996	61.454	0.07286
61.630	0.08114	61.455	0.07291
61.630	0.08113	61.455	0.07295
61.631	0.07875	61.455	0.073
61.631	0.0785	61.455	0.07305
61.631	0.07847	61.456	0.0731
61.631	0.07847	61.456	0.07314
61.631	0.07899	61.456	0.07319
61.631	0.07941	61.456	0.07323
61.632	0.07877	61.457	0.07328
61.632	0.07834	61.457	0.07332
61.632	0.07831	61.457	0.07336
61.632	0.07829	61.457	0.07341

61.632	0.07826	61.458	0.07345
61.633	0.07824	61.458	0.07349
61.633	0.07851	61.458	0.07353
61.633	0.07825	61.458	0.07357
61.633	0.07814	61.458	0.07361
61.633	0.07835	61.459	0.07365
61.633	0.07808	61.459	0.07369
61.634	0.07805	61.459	0.07372
61.634	0.07802	61.459	0.07376
61.634	0.07799	61.460	0.0738
61.634	0.07796	61.460	0.07383
61.634	0.07807	61.460	0.07387
61.635	0.07929	61.460	0.0739
61.635	0.07868	61.461	0.07394
61.635	0.07784	61.461	0.07397
61.635	0.07848	61.461	0.074
61.635	0.0781	61.461	0.07547
61.635	0.07774	61.462	0.07406
61.636	0.07853	61.462	0.07409
61.636	0.07808	61.462	0.07412
61.636	0.07765	61.462	0.07415
61.636	0.07762	61.463	0.07418
61.636	0.07758	61.463	0.0742
61.636	0.07755	61.463	0.07423
61.637	0.07789	61.463	0.07425
61.637	0.07974	61.464	0.07428
61.637	0.0793	61.464	0.0743
61.637	0.07808	61.464	0.07432
61.637	0.07738	61.464	0.07434
61.638	0.07734	61.465	0.07436
61.638	0.07731	61.465	0.07438
61.638	0.078	61.465	0.0744
61.638	0.07985	61.465	0.07442
61.638	0.07943	61.466	0.07443
61.638	0.07825	61.466	0.07445
61.639	0.07873	61.466	0.07447
61.639	0.0779	61.466	0.07448
61.639	0.07819	61.466	0.07449
61.639	0.07919	61.467	0.0745
61.639	0.07873	61.467	0.07452
61.640	0.07775	61.467	0.07453
61.640	0.0791	61.467	0.07475
61.640	0.07838	61.468	0.07455
61.640	0.0771	61.468	0.07455
61.640	0.07676	61.468	0.07456
61.640	0.07671	61.468	0.07457
61.641	0.07746	61.469	0.07457
61.641	0.07852	61.469	0.07458
61.641	0.07787	61.469	0.07458
61.641	0.07726	61.469	0.07458
61.641	0.07659	61.470	0.07459
61.642	0.07695	61.470	0.07459
61.642	0.07662	61.470	0.07459

61.642	0.07645	61.470	0.07459
61.642	0.07631	61.471	0.07458
61.642	0.07673	61.471	0.07458
61.642	0.0776	61.471	0.07458
61.643	0.07736	61.471	0.07457
61.643	0.07684	61.472	0.07457
61.643	0.07607	61.472	0.07456
61.643	0.07603	61.472	0.07456
61.643	0.07609	61.472	0.07455
61.644	0.07669	61.473	0.07497
61.644	0.07642	61.473	0.07453
61.644	0.07615	61.473	0.07454
61.644	0.07791	61.473	0.07455
61.644	0.07733	61.474	0.07455
61.645	0.0785	61.474	0.07456
61.645	0.07799	61.474	0.07457
61.645	0.07645	61.474	0.07457
61.645	0.07726	61.475	0.07487
61.645	0.07839	61.475	0.07458
61.645	0.07746	61.475	0.07458
61.646	0.07773	61.475	0.07458
61.646	0.07819	61.476	0.07457
61.646	0.07729	61.476	0.07457
61.646	0.07742	61.476	0.07494
61.646	0.07675	61.476	0.07456
61.647	0.07574	61.476	0.07495
61.647	0.0761	61.477	0.07455
61.647	0.07586	61.477	0.07454
61.647	0.0765	61.477	0.07453
61.647	0.07608	61.477	0.07452
61.647	0.07568	61.478	0.07451
61.648	0.07572	61.478	0.0745
61.648	0.07885	61.478	0.07449
61.648	0.0772	61.478	0.07447
61.648	0.07841	61.479	0.07446
61.648	0.07775	61.479	0.07444
61.649	0.07632	61.479	0.07442
61.649	0.07568	61.479	0.0744
61.649	0.07595	61.480	0.07438
61.649	0.07562	61.480	0.07436
61.649	0.07624	61.480	0.07434
61.650	0.07559	61.480	0.07434
61.650	0.07519	61.481	0.07433
61.650	0.07523	61.481	0.07613
61.650	0.07648	61.481	0.07432
61.650	0.07563	61.481	0.07431
61.650	0.07661	61.482	0.0743
61.651	0.07604	61.482	0.07429
61.651	0.07539	61.482	0.07428
61.651	0.07508	61.482	0.07427
61.651	0.07502	61.482	0.07426
61.651	0.075	61.483	0.07425
61.652	0.07499	61.483	0.07424

61.652	0.07497	61.483	0.07423
61.652	0.07552	61.483	0.07421
61.652	0.07622	61.484	0.0742
61.652	0.07555	61.484	0.0743
61.653	0.07533	61.484	0.07424
61.653	0.0755	61.484	0.07416
61.653	0.07554	61.485	0.07414
61.653	0.07525	61.485	0.07412
61.653	0.0748	61.485	0.07411
61.654	0.07479	61.485	0.07409
61.654	0.07471	61.486	0.07407
61.654	0.07485	61.486	0.07405
61.654	0.07498	61.486	0.07406
61.654	0.07495	61.486	0.07402
61.654	0.07554	61.486	0.07433
61.655	0.07469	61.487	0.07397
61.655	0.07443	61.487	0.07395
61.655	0.07438	61.487	0.07393
61.655	0.07471	61.487	0.07391
61.655	0.07512	61.488	0.07388
61.656	0.07444	61.488	0.07386
61.656	0.07422	61.488	0.07426
61.656	0.07418	61.488	0.07381
61.656	0.0744	61.489	0.07378
61.656	0.07413	61.489	0.07376
61.657	0.07458	61.489	0.07485
61.657	0.07488	61.489	0.0737
61.657	0.07468	61.489	0.07452
61.657	0.0747	61.490	0.07364
61.657	0.07438	61.490	0.07428
61.658	0.07473	61.490	0.07358
61.658	0.0741	61.490	0.07386
61.658	0.07459	61.491	0.07352
61.658	0.07431	61.491	0.07348
61.658	0.07389	61.491	0.07345
61.659	0.07384	61.491	0.07342
61.659	0.0737	61.491	0.07338
61.659	0.07365	61.492	0.0744
61.659	0.07354	61.492	0.07331
61.659	0.07376	61.492	0.07328
61.659	0.07433	61.492	0.07326
61.660	0.07385	61.493	0.07324
61.660	0.07466	61.493	0.07322
61.660	0.07422	61.493	0.07319
61.660	0.07387	61.493	0.07422
61.660	0.07443	61.494	0.07315
61.661	0.07375	61.494	0.07313
61.661	0.07354	61.494	0.0731
61.661	0.0733	61.494	0.07308
61.661	0.07334	61.494	0.07306
61.661	0.07335	61.495	0.07399
61.662	0.07335	61.495	0.07319
61.662	0.07335	61.495	0.07322

61.662	0.07335	61.495	0.07459
61.662	0.07336	61.496	0.07328
61.662	0.07337	61.496	0.07405
61.663	0.07338	61.496	0.07326
61.663	0.07339	61.496	0.07317
61.663	0.0734	61.496	0.07329
61.663	0.07341	61.497	0.07386
61.663	0.07342	61.497	0.07326
61.664	0.07343	61.497	0.07401
61.664	0.07344	61.497	0.07332
61.664	0.07357	61.498	0.07464
61.664	0.07361	61.498	0.07419
61.664	0.07355	61.498	0.0738
61.665	0.07347	61.498	0.07343
61.665	0.07347	61.498	0.07346
61.665	0.07379	61.499	0.07349
61.665	0.07398	61.499	0.07351
61.665	0.074	61.499	0.07358
61.666	0.0739	61.499	0.07356
61.666	0.07378	61.500	0.07358
61.666	0.0738	61.500	0.07361
61.666	0.07377	61.500	0.07366
61.666	0.07373	61.500	0.07365
61.666	0.07371	61.500	0.07367
61.667	0.07367	61.501	0.07369
61.667	0.07369	61.501	0.07371
61.667	0.0737	61.501	0.07373
61.667	0.07372	61.501	0.07397
61.667	0.07373	61.502	0.07414
61.668	0.07374	61.502	0.07378
61.668	0.07377	61.502	0.07379
61.668	0.07402	61.502	0.07381
61.668	0.07386	61.502	0.07382
61.668	0.07387	61.503	0.07384
61.669	0.0739	61.503	0.07401
61.669	0.07393	61.503	0.07386
61.669	0.07396	61.503	0.07387
61.669	0.07398	61.504	0.07513
61.669	0.07401	61.504	0.07435
61.670	0.07404	61.504	0.0739
61.670	0.07406	61.504	0.07431
61.670	0.07428	61.504	0.07392
61.670	0.07412	61.505	0.07576
61.670	0.07414	61.505	0.07396
61.671	0.07417	61.505	0.07466
61.671	0.07419	61.505	0.07401
61.671	0.07421	61.506	0.07403
61.671	0.07424	61.506	0.07406
61.671	0.07426	61.506	0.07408
61.672	0.07428	61.506	0.0741
61.672	0.0743	61.506	0.07412
61.672	0.07432	61.507	0.07413
61.672	0.07434	61.507	0.07415

61.672	0.07447	61.507	0.07417
61.673	0.07437	61.507	0.07418
61.673	0.07439	61.508	0.07419
61.673	0.07441	61.508	0.07421
61.673	0.07442	61.508	0.0744
61.673	0.07444	61.508	0.07582
61.674	0.07445	61.508	0.07423
61.674	0.07446	61.509	0.07424
61.674	0.07448	61.509	0.07425
61.674	0.07449	61.509	0.07425
61.674	0.0745	61.509	0.07425
61.675	0.07451	61.510	0.07425
61.675	0.07452	61.510	0.07425
61.675	0.07452	61.510	0.07425
61.675	0.07453	61.510	0.07425
61.675	0.07453	61.511	0.07424
61.676	0.07453	61.511	0.07424
61.676	0.07455	61.511	0.07423
61.676	0.07454	61.511	0.07422
61.676	0.07453	61.511	0.07421
61.676	0.07453	61.512	0.0742
61.677	0.07453	61.512	0.07418
61.677	0.07453	61.512	0.07417
61.677	0.07452	61.512	0.07415
61.677	0.07452	61.513	0.07413
61.677	0.07451	61.513	0.07503
61.677	0.0745	61.513	0.07409
61.678	0.07449	61.513	0.07407
61.678	0.07448	61.513	0.07404
61.678	0.07447	61.514	0.07402
61.678	0.07446	61.514	0.07399
61.678	0.07444	61.514	0.07396
61.679	0.07443	61.514	0.07393
61.679	0.07441	61.515	0.0739
61.679	0.07439	61.515	0.07386
61.679	0.07437	61.515	0.07383
61.679	0.07435	61.515	0.07379
61.680	0.07433	61.515	0.07463
61.680	0.07431	61.516	0.076
61.680	0.07429	61.516	0.07385
61.680	0.07427	61.516	0.07363
61.680	0.07424	61.516	0.07358
61.681	0.07422	61.517	0.07354
61.681	0.07419	61.517	0.07349
61.681	0.07416	61.517	0.07344
61.681	0.07413	61.517	0.07423
61.681	0.0741	61.517	0.07499
61.682	0.07407	61.518	0.07355
61.682	0.07404	61.518	0.07324
61.682	0.074	61.518	0.07496
61.682	0.07397	61.518	0.07625
61.682	0.07393	61.519	0.07403
61.683	0.07389	61.519	0.07308

61.683	0.07386	61.519	0.07305
61.683	0.07382	61.519	0.07302
61.683	0.07378	61.519	0.07299
61.683	0.07374	61.520	0.07295
61.684	0.07369	61.520	0.07292
61.684	0.07365	61.520	0.07289
61.684	0.07361	61.520	0.07515
61.684	0.07356	61.521	0.07655
61.684	0.07352	61.521	0.07486
61.685	0.07347	61.521	0.07587
61.685	0.07342	61.521	0.07406
61.685	0.07337	61.522	0.07467
61.685	0.07332	61.522	0.07557
61.685	0.07327	61.522	0.07393
61.686	0.07322	61.522	0.07447
61.686	0.07317	61.522	0.07627
61.686	0.07312	61.523	0.07441
61.686	0.07306	61.523	0.07256
61.686	0.07301	61.523	0.07253
61.687	0.07295	61.523	0.07251
61.687	0.0729	61.524	0.07248
61.687	0.07284	61.524	0.07246
61.687	0.07279	61.524	0.07447
61.687	0.07273	61.524	0.07626
61.687	0.07268	61.524	0.0744
61.688	0.07265	61.525	0.07235
61.688	0.07261	61.525	0.07233
61.688	0.07258	61.525	0.07231
61.688	0.07254	61.525	0.07359
61.688	0.07251	61.526	0.07542
61.689	0.07247	61.526	0.07388
61.689	0.07243	61.526	0.07219
61.689	0.07239	61.526	0.0746
61.689	0.07235	61.526	0.07213
61.689	0.07231	61.527	0.07752
61.690	0.07228	61.527	0.07668
61.690	0.07223	61.527	0.07207
61.690	0.07219	61.527	0.07382
61.690	0.07215	61.528	0.07419
61.690	0.07211	61.528	0.07378
61.691	0.07207	61.528	0.0759
61.691	0.07203	61.528	0.07679
61.691	0.07198	61.528	0.07417
61.691	0.07194	61.529	0.07251
61.691	0.0719	61.529	0.07312
61.692	0.07185	61.529	0.07242
61.692	0.07181	61.529	0.07203
61.692	0.07176	61.530	0.07196
61.692	0.07172	61.530	0.07218
61.692	0.07167	61.530	0.07529
61.693	0.07163	61.530	0.07664
61.693	0.07158	61.530	0.07519
61.693	0.07153	61.531	0.07395

61.693	0.07149	61.531	0.07475
61.693	0.07144	61.531	0.0733
61.694	0.07139	61.531	0.07232
61.694	0.07134	61.532	0.07592
61.694	0.0713	61.532	0.07678
61.694	0.07125	61.532	0.07415
61.694	0.07121	61.532	0.07244
61.694	0.07116	61.532	0.07245
61.695	0.07111	61.533	0.0725
61.695	0.07107	61.533	0.07255
61.695	0.07102	61.533	0.0728
61.695	0.07097	61.533	0.07366
61.695	0.07092	61.534	0.0728
61.696	0.07088	61.534	0.07278
61.696	0.07085	61.534	0.07285
61.696	0.07086	61.534	0.07293
61.696	0.07087	61.534	0.073
61.696	0.07087	61.535	0.07615
61.697	0.07088	61.535	0.07696
61.697	0.07089	61.535	0.0743
61.697	0.07089	61.535	0.07393
61.697	0.0709	61.536	0.07484
61.697	0.07091	61.536	0.07445
61.698	0.07091	61.536	0.07575
61.698	0.07091	61.536	0.07452
61.698	0.07092	61.536	0.07369
61.698	0.07092	61.537	0.07376
61.698	0.07092	61.537	0.07431
61.699	0.07093	61.537	0.07395
61.699	0.07094	61.537	0.07397
61.699	0.07094	61.538	0.07404
61.699	0.07095	61.538	0.07628
61.699	0.07095	61.538	0.07706
61.700	0.07096	61.538	0.07438
61.700	0.07096	61.538	0.07429
61.700	0.07096	61.539	0.07435
61.700	0.07097	61.539	0.07441
61.700	0.07097	61.539	0.07447
61.701	0.07097	61.539	0.07453
61.701	0.07097	61.540	0.07459
61.701	0.07097	61.540	0.07464
61.701	0.07097	61.540	0.07469
61.701	0.07096	61.540	0.07662
61.702	0.07096	61.541	0.0773
61.702	0.07096	61.541	0.07485
61.702	0.07095	61.541	0.07489
61.702	0.07094	61.541	0.07494
61.702	0.07094	61.541	0.07498
61.702	0.07093	61.542	0.07503
61.703	0.07092	61.542	0.07507
61.703	0.07091	61.542	0.07511
61.703	0.0709	61.542	0.07515
61.703	0.07089	61.543	0.07518

61.703	0.07087	61.543	0.07522
61.704	0.07086	61.543	0.07668
61.704	0.07084	61.543	0.07735
61.704	0.07083	61.543	0.07532
61.704	0.07081	61.544	0.07535
61.704	0.07079	61.544	0.07538
61.705	0.07077	61.544	0.07541
61.705	0.07078	61.544	0.07543
61.705	0.07079	61.545	0.07586
61.705	0.0708	61.545	0.07711
61.705	0.07081	61.545	0.07551
61.706	0.07081	61.545	0.07553
61.706	0.07082	61.545	0.07555
61.706	0.07082	61.546	0.07557
61.706	0.07083	61.546	0.07559
61.706	0.07083	61.546	0.07592
61.707	0.07083	61.546	0.07711
61.707	0.07083	61.547	0.07566
61.707	0.07083	61.547	0.07568
61.707	0.07083	61.547	0.07573
61.707	0.07082	61.547	0.07579
61.708	0.07082	61.548	0.07584
61.708	0.07081	61.548	0.07666
61.708	0.07081	61.548	0.07594
61.708	0.0708	61.548	0.076
61.708	0.07079	61.548	0.07605
61.709	0.07078	61.549	0.0761
61.709	0.07077	61.549	0.07615
61.709	0.07076	61.549	0.07673
61.709	0.07074	61.549	0.07625
61.709	0.07073	61.550	0.07631
61.710	0.07071	61.550	0.07636
61.710	0.0707	61.550	0.07641
61.710	0.07068	61.550	0.07646
61.710	0.07066	61.550	0.0765
61.710	0.07064	61.551	0.07655
61.710	0.07062	61.551	0.0766
61.711	0.0706	61.551	0.07665
61.711	0.07057	61.551	0.0767
61.711	0.07055	61.552	0.07674
61.711	0.07053	61.552	0.07679
61.711	0.0705	61.552	0.07683
61.712	0.07047	61.552	0.07688
61.712	0.07045	61.553	0.07692
61.712	0.07042	61.553	0.07697
61.712	0.07039	61.553	0.07701
61.712	0.07036	61.553	0.07705
61.713	0.07033	61.553	0.07709
61.713	0.0703	61.554	0.07713
61.713	0.07027	61.554	0.07717
61.713	0.07024	61.554	0.07721
61.713	0.0702	61.554	0.07724
61.714	0.07017	61.555	0.07728

61.714	0.07013	61.555	0.07737
61.714	0.0701	61.555	0.07794
61.714	0.07006	61.555	0.07738
61.714	0.07002	61.556	0.07771
61.715	0.06998	61.556	0.07744
61.715	0.06994	61.556	0.07746
61.715	0.0699	61.556	0.07749
61.715	0.06986	61.557	0.07751
61.715	0.06981	61.557	0.07754
61.716	0.06977	61.557	0.07756
61.716	0.06973	61.557	0.07758
61.716	0.06968	61.557	0.0776
61.716	0.06964	61.558	0.07836
61.716	0.06959	61.558	0.07763
61.717	0.06954	61.558	0.07764
61.717	0.0695	61.558	0.07765
61.717	0.06945	61.559	0.07766
61.717	0.0694	61.559	0.07767
61.717	0.06935	61.559	0.07768
61.718	0.0693	61.559	0.07768
61.718	0.06925	61.560	0.07769
61.718	0.0692	61.560	0.07769
61.718	0.06915	61.560	0.07769
61.718	0.0691	61.560	0.07768
61.719	0.06905	61.561	0.07768
61.719	0.069	61.561	0.07767
61.719	0.06895	61.561	0.07766
61.719	0.06894	61.561	0.07765
61.719	0.06895	61.562	0.07764
61.719	0.06896	61.562	0.07762
61.720	0.06897	61.562	0.07761
61.720	0.06898	61.562	0.07759
61.720	0.06898	61.563	0.07757
61.720	0.06899	61.563	0.07754
61.720	0.069	61.563	0.07752
61.721	0.069	61.563	0.07749
61.721	0.06901	61.563	0.07746
61.721	0.06901	61.564	0.07743
61.721	0.06901	61.564	0.0774
61.721	0.06901	61.564	0.07736
61.722	0.06901	61.564	0.07732
61.722	0.06901	61.565	0.07728
61.722	0.06901	61.565	0.07724
61.722	0.06901	61.565	0.07719
61.722	0.06901	61.565	0.07715
61.723	0.069	61.566	0.0771
61.723	0.069	61.566	0.07704
61.723	0.06899	61.566	0.07699
61.723	0.06898	61.566	0.07693
61.723	0.06898	61.567	0.07687
61.724	0.06897	61.567	0.07681
61.724	0.06896	61.567	0.07675
61.724	0.06895	61.567	0.07668

61.724	0.06894	61.568	0.07661
61.724	0.06892	61.568	0.07654
61.725	0.06891	61.568	0.07647
61.725	0.0689	61.568	0.0764
61.725	0.06888	61.569	0.07632
61.725	0.06886	61.569	0.07624
61.725	0.06885	61.569	0.07616
61.726	0.06883	61.569	0.07608
61.726	0.06881	61.570	0.076
61.726	0.06879	61.570	0.07591
61.726	0.06877	61.570	0.07585
61.726	0.06875	61.570	0.07579
61.727	0.06872	61.571	0.07572
61.727	0.0687	61.571	0.07565
61.727	0.06868	61.571	0.07558
61.727	0.06865	61.571	0.0755
61.727	0.06862	61.572	0.07543
61.728	0.0686	61.572	0.07535
61.728	0.06857	61.572	0.07527
61.728	0.06854	61.572	0.07519
61.728	0.06851	61.573	0.07511
61.728	0.06848	61.573	0.07502
61.729	0.06845	61.573	0.07494
61.729	0.06841	61.573	0.07485
61.729	0.06838	61.574	0.07476
61.729	0.06834	61.574	0.07467
61.729	0.06831	61.574	0.07457
61.730	0.06827	61.574	0.07448
61.730	0.06823	61.575	0.07438
61.730	0.06819	61.575	0.07428
61.730	0.06816	61.575	0.07418
61.730	0.06812	61.576	0.07408
61.731	0.06807	61.576	0.07398
61.731	0.06803	61.576	0.07387
61.731	0.06799	61.576	0.07376
61.731	0.06798	61.577	0.07375
61.731	0.06798	61.577	0.07385
61.732	0.06797	61.577	0.07343
61.732	0.06797	61.577	0.07472
61.732	0.06796	61.578	0.07321
61.732	0.06795	61.578	0.07309
61.732	0.06794	61.578	0.07297
61.733	0.06793	61.578	0.07285
61.733	0.06792	61.579	0.07273
61.733	0.0679	61.579	0.07429
61.733	0.06789	61.579	0.07249
61.733	0.06788	61.579	0.07413
61.734	0.06786	61.580	0.07224
61.734	0.06784	61.580	0.07222
61.734	0.06783	61.580	0.0727
61.734	0.06781	61.581	0.07186
61.734	0.06779	61.581	0.07174
61.735	0.06777	61.581	0.07208

61.735	0.06775	61.581	0.07235
61.735	0.06772	61.582	0.07135
61.735	0.0677	61.582	0.07171
61.735	0.06768	61.582	0.07167
61.736	0.06765	61.582	0.07127
61.736	0.06763	61.583	0.07289
61.736	0.0676	61.583	0.07224
61.736	0.06757	61.583	0.07165
61.736	0.06754	61.584	0.07042
61.737	0.06751	61.584	0.07069
61.737	0.06748	61.584	0.07072
61.737	0.06745	61.584	0.07188
61.737	0.06742	61.585	0.07177
61.737	0.06738	61.585	0.07111
61.738	0.06735	61.585	0.07062
61.738	0.06731	61.585	0.07106
61.738	0.06728	61.586	0.06981
61.738	0.06724	61.586	0.07292
61.738	0.0672	61.586	0.07078
61.739	0.06717	61.587	0.07158
61.739	0.06713	61.587	0.06986
61.739	0.06709	61.587	0.06999
61.739	0.06704	61.587	0.06895
61.739	0.067	61.588	0.06886
61.740	0.06696	61.588	0.06996
61.740	0.06692	61.588	0.07053
61.740	0.06687	61.588	0.07003
61.740	0.06683	61.589	0.07169
61.740	0.06678	61.589	0.06994
61.741	0.06674	61.589	0.07002
61.741	0.06669	61.589	0.0698
61.741	0.0669	61.590	0.07015
61.741	0.0666	61.590	0.06917
61.741	0.06655	61.590	0.06939
61.742	0.0665	61.590	0.06943
61.742	0.06645	61.591	0.06835
61.742	0.0664	61.591	0.0688
61.742	0.06688	61.591	0.06989
61.742	0.0663	61.591	0.06916
61.743	0.06624	61.592	0.06759
61.743	0.06619	61.592	0.06738
61.743	0.06614	61.592	0.06787
61.743	0.06608	61.592	0.06725
61.743	0.06683	61.593	0.06798
61.744	0.06601	61.593	0.06675
61.744	0.06592	61.593	0.06815
61.744	0.06591	61.593	0.06883
61.744	0.06593	61.594	0.06874
61.744	0.06671	61.594	0.06812
61.745	0.06596	61.594	0.06872
61.745	0.06597	61.594	0.06701
61.745	0.06598	61.595	0.06771
61.745	0.066	61.595	0.06771

61.745	0.06657	61.595	0.06848
61.746	0.06601	61.595	0.067
61.746	0.06602	61.596	0.06772
61.746	0.06603	61.596	0.06592
61.746	0.06603	61.596	0.06633
61.746	0.06651	61.596	0.0662
61.747	0.06603	61.596	0.06798
61.747	0.06603	61.597	0.06821
61.747	0.06603	61.597	0.06961
61.747	0.06663	61.597	0.0681
61.747	0.06603	61.597	0.06766
61.748	0.06602	61.598	0.06686
61.748	0.06601	61.598	0.06786
61.748	0.0663	61.598	0.06637
61.748	0.06599	61.598	0.06749
61.748	0.06643	61.599	0.06734
61.749	0.06682	61.599	0.06584
61.749	0.06595	61.599	0.06882
61.749	0.06616	61.599	0.06636
61.749	0.06592	61.600	0.06813
61.749	0.06743	61.600	0.06745
61.750	0.06715	61.600	0.06724
61.750	0.06586	61.600	0.06586
61.750	0.06584	61.600	0.06694
61.750	0.06581	61.601	0.06682
61.750	0.06578	61.601	0.06559
61.751	0.06576	61.601	0.06591
61.751	0.06573	61.601	0.06686
61.751	0.06569	61.602	0.06688
61.751	0.06566	61.602	0.06626
61.751	0.06563	61.602	0.06626
61.752	0.06559	61.602	0.06801
61.752	0.06555	61.602	0.0668
61.752	0.06552	61.603	0.06601
61.752	0.06547	61.603	0.06537
61.752	0.06543	61.603	0.06589
61.753	0.06539	61.603	0.06597
61.753	0.06534	61.604	0.06582
61.753	0.0653	61.604	0.065
61.753	0.06525	61.604	0.06611
61.753	0.0652	61.604	0.06429
61.754	0.06515	61.605	0.0651
61.754	0.0651	61.605	0.06428
61.754	0.06504	61.605	0.06532
61.754	0.06499	61.605	0.06488
61.755	0.06493	61.605	0.06552
61.755	0.06487	61.606	0.06601
61.755	0.06481	61.606	0.06554
61.755	0.06475	61.606	0.06418
61.755	0.06469	61.606	0.06311
61.756	0.06462	61.607	0.06308
61.756	0.06456	61.607	0.06433
61.756	0.06449	61.607	0.0658

61.756	0.06442	61.607	0.06419
61.756	0.06435	61.607	0.06344
61.757	0.06428	61.608	0.06388
61.757	0.06421	61.608	0.06523
61.757	0.06413	61.608	0.06442
61.757	0.06406	61.608	0.06294
61.757	0.06398	61.609	0.06406
61.758	0.06391	61.609	0.06491
61.758	0.06383	61.609	0.06434
61.758	0.06375	61.609	0.06493
61.758	0.06367	61.610	0.06466
61.758	0.06364	61.610	0.06302
61.759	0.06361	61.610	0.06448
61.759	0.06358	61.610	0.06317
61.759	0.06355	61.610	0.0648
61.759	0.06352	61.611	0.06423
61.759	0.06348	61.611	0.06405
61.760	0.06345	61.611	0.06334
61.760	0.06341	61.611	0.06453
61.760	0.06338	61.612	0.06344
61.760	0.06334	61.612	0.06402
61.760	0.0633	61.612	0.0637
61.761	0.06326	61.612	0.06398
61.761	0.06322	61.612	0.06243
61.761	0.06318	61.613	0.06356
61.761	0.06314	61.613	0.062
61.761	0.06309	61.613	0.06347
61.762	0.06305	61.613	0.06408
61.762	0.06301	61.614	0.06323
61.762	0.06296	61.614	0.06358
61.762	0.06291	61.614	0.06304
61.762	0.06287	61.614	0.06341
61.763	0.06282	61.615	0.06467
61.763	0.06277	61.615	0.06335
61.763	0.06272	61.615	0.06377
61.763	0.06267	61.615	0.06328
61.763	0.06261	61.615	0.06275
61.764	0.06256	61.616	0.0617
61.764	0.06251	61.616	0.06222
61.764	0.06245	61.616	0.06211
61.764	0.0624	61.616	0.06295
61.764	0.06234	61.617	0.06241
61.765	0.06228	61.617	0.06276
61.765	0.06223	61.617	0.06173
61.765	0.06217	61.617	0.06248
61.765	0.06211	61.617	0.06225
61.765	0.06205	61.618	0.06103
61.766	0.06199	61.618	0.06272
61.766	0.06192	61.618	0.06135
61.766	0.06186	61.618	0.06284
61.766	0.06182	61.619	0.06137
61.766	0.06181	61.619	0.06239
61.767	0.06179	61.619	0.06104

61.767	0.06177	61.619	0.06039
61.767	0.06176	61.620	0.06036
61.767	0.06174	61.620	0.0603
61.767	0.06172	61.620	0.06028
61.768	0.0617	61.620	0.06132
61.768	0.06167	61.620	0.06029
61.768	0.06165	61.621	0.06105
61.768	0.06163	61.621	0.06143
61.769	0.06161	61.621	0.06043
61.769	0.06158	61.621	0.06155
61.769	0.06155	61.622	0.06018
61.769	0.06153	61.622	0.06134
61.769	0.0615	61.622	0.06095
61.770	0.06147	61.622	0.06003
61.770	0.06144	61.623	0.0605
61.770	0.06142	61.623	0.06126
61.770	0.06139	61.623	0.05989
61.770	0.06135	61.623	0.05985
61.771	0.06132	61.623	0.06034
61.771	0.06129	61.624	0.0607
61.771	0.06126	61.624	0.05971
61.771	0.06122	61.624	0.06043
61.771	0.06119	61.624	0.06052
61.772	0.06115	61.625	0.05964
61.772	0.06112	61.625	0.06028
61.772	0.06108	61.625	0.06032
61.772	0.06104	61.625	0.06036
61.773	0.061	61.625	0.06036
61.773	0.06097	61.626	0.05951
61.773	0.06093	61.626	0.06011
61.773	0.06089	61.626	0.05989
61.773	0.06085	61.626	0.0602
61.774	0.0608	61.627	0.05942
61.774	0.06076	61.627	0.05973
61.774	0.06072	61.627	0.05999
61.774	0.06068	61.627	0.05907
61.774	0.06063	61.627	0.05901
61.775	0.06059	61.628	0.05966
61.775	0.06054	61.628	0.05951
61.775	0.0605	61.628	0.05988
61.775	0.06045	61.628	0.05966
61.776	0.0604	61.629	0.05873
61.776	0.06036	61.629	0.05867
61.776	0.06031	61.629	0.05929
61.776	0.06041	61.629	0.05856
61.776	0.06148	61.630	0.05852
61.777	0.06116	61.630	0.05929
61.777	0.06047	61.630	0.05839
61.777	0.06041	61.630	0.05833
61.777	0.06044	61.630	0.05974
61.777	0.06047	61.631	0.05839
61.778	0.0605	61.631	0.05815
61.778	0.06054	61.631	0.05809

61.778	0.06084	61.631	0.05803
61.778	0.0606	61.632	0.05796
61.779	0.06062	61.632	0.0579
61.779	0.06065	61.632	0.05783
61.779	0.06068	61.632	0.05854
61.779	0.06125	61.633	0.0577
61.779	0.06073	61.633	0.05764
61.780	0.06076	61.633	0.05797
61.780	0.06078	61.633	0.05792
61.780	0.06081	61.633	0.05748
61.780	0.06083	61.634	0.05766
61.781	0.06086	61.634	0.05795
61.781	0.06088	61.634	0.05731
61.781	0.0609	61.634	0.05725
61.781	0.06092	61.635	0.05719
61.781	0.06094	61.635	0.05713
61.782	0.06097	61.635	0.0577
61.782	0.06103	61.635	0.05701
61.782	0.06109	61.636	0.05755
61.782	0.06116	61.636	0.0569
61.783	0.06122	61.636	0.05682
61.783	0.06129	61.636	0.05739
61.783	0.06135	61.636	0.05669
61.783	0.06142	61.637	0.05662
61.783	0.06149	61.637	0.05655
61.784	0.06155	61.637	0.05693
61.784	0.06162	61.637	0.05642
61.784	0.06168	61.638	0.05635
61.784	0.06175	61.638	0.05628
61.785	0.06181	61.638	0.0569
61.785	0.06187	61.638	0.05682
61.785	0.06193	61.639	0.05606
61.785	0.06199	61.639	0.05599
61.786	0.06205	61.639	0.05629
61.786	0.06211	61.639	0.05679
61.786	0.06216	61.640	0.05655
61.786	0.06222	61.640	0.05576
61.786	0.06227	61.640	0.05627
61.787	0.06232	61.640	0.05582
61.787	0.06237	61.640	0.05616
61.787	0.06242	61.641	0.0554
61.787	0.06246	61.641	0.05536
61.788	0.06251	61.641	0.05601
61.788	0.06255	61.641	0.05541
61.788	0.06259	61.642	0.05602
61.788	0.06263	61.642	0.05501
61.788	0.06267	61.642	0.05589
61.789	0.0627	61.642	0.05506
61.789	0.06274	61.643	0.05553
61.789	0.06277	61.643	0.05473
61.789	0.0628	61.643	0.05584
61.790	0.06283	61.643	0.05452
61.790	0.06285	61.644	0.0558

61.790	0.06288	61.644	0.05463
61.790	0.0629	61.644	0.05428
61.791	0.06312	61.644	0.05432
61.791	0.06294	61.645	0.05411
61.791	0.06296	61.645	0.05403
61.791	0.06297	61.645	0.05394
61.792	0.06299	61.645	0.05386
61.792	0.063	61.646	0.05415
61.792	0.06301	61.646	0.05369
61.792	0.06302	61.646	0.05408
61.792	0.06303	61.646	0.05352
61.793	0.06339	61.646	0.05403
61.793	0.06304	61.647	0.05364
61.793	0.06304	61.647	0.05408
61.793	0.06304	61.647	0.0533
61.794	0.06304	61.647	0.05324
61.794	0.06304	61.648	0.05319
61.794	0.06304	61.648	0.05313
61.794	0.06304	61.648	0.05308
61.795	0.06304	61.648	0.05337
61.795	0.06304	61.649	0.05377
61.795	0.06305	61.649	0.0532
61.795	0.06309	61.649	0.05379
61.795	0.06314	61.649	0.05371
61.796	0.06319	61.650	0.0538
61.796	0.06324	61.650	0.05323
61.796	0.06329	61.650	0.05374
61.796	0.06334	61.650	0.05271
61.797	0.06338	61.651	0.05252
61.797	0.06343	61.651	0.05267
61.797	0.06348	61.651	0.05344
61.797	0.06352	61.651	0.05302
61.798	0.06357	61.652	0.05324
61.798	0.06361	61.652	0.0526
61.798	0.06365	61.652	0.05303
61.798	0.0637	61.652	0.0524
61.799	0.06374	61.653	0.05289
61.799	0.06378	61.653	0.052
61.799	0.06382	61.653	0.05234
61.799	0.06386	61.653	0.05244
61.800	0.0639	61.654	0.05279
61.800	0.06393	61.654	0.05209
61.800	0.06397	61.654	0.05213
61.800	0.064	61.654	0.05249
61.800	0.06404	61.655	0.05237
61.801	0.06407	61.655	0.05226
61.801	0.0641	61.655	0.05267
61.801	0.06414	61.655	0.05234
61.801	0.06417	61.656	0.05238
61.802	0.06419	61.656	0.05242
61.802	0.06422	61.656	0.05246
61.802	0.06425	61.656	0.0525
61.802	0.06427	61.657	0.05254

61.803	0.0643	61.657	0.05257
61.803	0.06432	61.657	0.05261
61.803	0.06434	61.657	0.05264
61.803	0.06436	61.658	0.05268
61.804	0.06437	61.658	0.05272
61.804	0.06439	61.658	0.05276
61.804	0.06441	61.658	0.0528
61.804	0.06442	61.659	0.05285
61.805	0.06443	61.659	0.05289
61.805	0.06444	61.659	0.05293
61.805	0.06445	61.659	0.05297
61.805	0.06445	61.660	0.05301
61.806	0.06446	61.660	0.05305
61.806	0.06446	61.660	0.05309
61.806	0.06446	61.660	0.05312
61.806	0.06448	61.661	0.05316
61.807	0.06458	61.661	0.05319
61.807	0.06468	61.661	0.05323
61.807	0.06478	61.661	0.05326
61.807	0.06488	61.662	0.05329
61.807	0.06497	61.662	0.05333
61.808	0.06506	61.662	0.05336
61.808	0.06515	61.662	0.05339
61.808	0.06524	61.663	0.05342
61.808	0.06533	61.663	0.05345
61.809	0.06541	61.663	0.05347
61.809	0.06549	61.664	0.0535
61.809	0.06557	61.664	0.05353
61.809	0.06565	61.664	0.05355
61.810	0.06572	61.664	0.05358
61.810	0.06579	61.665	0.0536
61.810	0.06586	61.665	0.05362
61.810	0.06593	61.665	0.05364
61.811	0.06599	61.665	0.05366
61.811	0.06605	61.666	0.05368
61.811	0.06611	61.666	0.0537
61.811	0.06616	61.666	0.05372
61.812	0.06621	61.666	0.05373
61.812	0.06626	61.667	0.05375
61.812	0.0663	61.667	0.05376
61.812	0.06634	61.667	0.05378
61.813	0.06637	61.667	0.05379
61.813	0.06641	61.668	0.0538
61.813	0.06644	61.668	0.05381
61.813	0.06646	61.668	0.05382
61.814	0.06648	61.668	0.05383
61.814	0.0665	61.669	0.05384
61.814	0.06651	61.669	0.05384
61.814	0.06652	61.669	0.05385
61.815	0.06653	61.669	0.05385
61.815	0.06653	61.670	0.05386
61.815	0.06653	61.670	0.05386
61.815	0.06652	61.670	0.05386

61.815	0.06651	61.670	0.05386
61.816	0.0665	61.671	0.05386
61.816	0.06648	61.671	0.05386
61.816	0.06646	61.671	0.05388
61.816	0.06643	61.671	0.0539
61.817	0.0664	61.672	0.05392
61.817	0.06637	61.672	0.05395
61.817	0.06633	61.672	0.05397
61.817	0.06629	61.672	0.05399
61.818	0.06624	61.673	0.05401
61.818	0.06619	61.673	0.05403
61.818	0.06614	61.673	0.05404
61.818	0.06608	61.674	0.05406
61.819	0.06602	61.674	0.05408
61.819	0.06596	61.674	0.0541
61.819	0.06589	61.674	0.05411
61.819	0.06582	61.675	0.05413
61.820	0.06575	61.675	0.05414
61.820	0.06567	61.675	0.05416
61.820	0.06559	61.675	0.05417
61.820	0.06551	61.676	0.05418
61.821	0.06542	61.676	0.05419
61.821	0.06533	61.676	0.05421
61.821	0.06524	61.676	0.05422
61.821	0.06514	61.677	0.05423
61.822	0.06504	61.677	0.05424
61.822	0.06494	61.677	0.05425
61.822	0.06484	61.677	0.05426
61.822	0.06473	61.678	0.05427
61.823	0.06462	61.678	0.05428
61.823	0.06451	61.678	0.05429
61.823	0.06439	61.678	0.0543
61.823	0.06428	61.679	0.05432
61.824	0.06416	61.679	0.05433
61.824	0.06403	61.679	0.05435
61.824	0.06391	61.679	0.05436
61.824	0.06378	61.680	0.05438
61.825	0.06365	61.680	0.05439
61.825	0.06352	61.680	0.05441
61.825	0.06339	61.680	0.05442
61.825	0.06325	61.681	0.05444
61.826	0.06312	61.681	0.05445
61.826	0.06298	61.681	0.05447
61.826	0.06311	61.682	0.05448
61.826	0.06346	61.682	0.0545
61.826	0.06265	61.682	0.05451
61.827	0.06263	61.682	0.05452
61.827	0.06273	61.683	0.05453
61.827	0.06285	61.683	0.05455
61.827	0.06298	61.683	0.05456
61.828	0.06312	61.683	0.05457
61.828	0.06327	61.684	0.05458
61.828	0.06343	61.684	0.05459

61.828	0.0636	61.684	0.0546
61.829	0.06376	61.684	0.05461
61.829	0.06392	61.685	0.05462
61.829	0.06408	61.685	0.05463
61.829	0.06424	61.685	0.05464
61.830	0.0644	61.685	0.05465
61.830	0.06455	61.686	0.05466
61.830	0.0647	61.686	0.05466
61.830	0.06485	61.686	0.05467
61.831	0.06499	61.686	0.05468
61.831	0.06512	61.687	0.05468
61.831	0.06525	61.687	0.05469
61.831	0.06538	61.687	0.05469
61.832	0.06551	61.687	0.0547
61.832	0.06562	61.688	0.0547
61.832	0.06574	61.688	0.0547
61.832	0.06584	61.688	0.0547
61.833	0.06594	61.688	0.0547
61.833	0.06604	61.689	0.0547
61.833	0.06613	61.689	0.0547
61.833	0.06621	61.689	0.0547
61.834	0.06629	61.690	0.0547
61.834	0.06636	61.690	0.0547
61.834	0.06642	61.690	0.0547
61.834	0.06648	61.690	0.05469
61.835	0.06653	61.691	0.05469
61.835	0.06657	61.691	0.05468
61.835	0.0666	61.691	0.05468
61.835	0.06663	61.691	0.05467
61.836	0.06665	61.692	0.05466
61.836	0.06667	61.692	0.05465
61.836	0.06667	61.692	0.05465
61.836	0.06668	61.692	0.05464
61.837	0.06667	61.693	0.05463
61.837	0.06666	61.693	0.05461
61.837	0.06664	61.693	0.0546
61.837	0.06661	61.693	0.05459
61.838	0.06658	61.694	0.05458
61.838	0.06654	61.694	0.05456
61.838	0.06649	61.694	0.05455
61.838	0.06644	61.694	0.05453
61.839	0.06638	61.695	0.05451
61.839	0.06631	61.695	0.0545
61.839	0.06624	61.695	0.05448
61.839	0.06617	61.695	0.05446
61.840	0.06608	61.696	0.05444
61.840	0.066	61.696	0.05442
61.840	0.0659	61.696	0.0544
61.840	0.0658	61.696	0.05437
61.841	0.0657	61.697	0.05435
61.841	0.06559	61.697	0.05433
61.841	0.06547	61.697	0.0543
61.841	0.06535	61.697	0.05428

61.842	0.06523	61.698	0.05425
61.842	0.0651	61.698	0.05422
61.842	0.06497	61.698	0.0542
61.842	0.06484	61.698	0.05417
61.843	0.0647	61.699	0.05418
61.843	0.06455	61.699	0.0542
61.843	0.06441	61.699	0.05422
61.843	0.06426	61.699	0.05424
61.844	0.0641	61.700	0.05426
61.844	0.06395	61.700	0.05427
61.844	0.06379	61.700	0.05429
61.844	0.06363	61.700	0.05431
61.845	0.06346	61.701	0.05432
61.845	0.06344	61.701	0.05434
61.845	0.06366	61.701	0.05435
61.845	0.06296	61.701	0.05436
61.846	0.06279	61.702	0.05438
61.846	0.06261	61.702	0.05439
61.846	0.06361	61.702	0.0544
61.846	0.06387	61.703	0.05441
61.847	0.06238	61.703	0.05442
61.847	0.06202	61.703	0.05443
61.847	0.06196	61.703	0.05443
61.847	0.0619	61.704	0.05444
61.848	0.06368	61.704	0.05445
61.848	0.06439	61.704	0.05445
61.848	0.06294	61.704	0.05446
61.848	0.06272	61.705	0.05446
61.849	0.06298	61.705	0.05446
61.849	0.06195	61.705	0.05447
61.849	0.06219	61.705	0.05447
61.849	0.06212	61.706	0.05447
61.850	0.0622	61.706	0.05447
61.850	0.06228	61.706	0.05447
61.850	0.06236	61.706	0.05446
61.850	0.06244	61.707	0.05446
61.851	0.06252	61.707	0.05446
61.851	0.06259	61.707	0.05445
61.851	0.06267	61.707	0.05445
61.851	0.06274	61.708	0.05444
61.852	0.0628	61.708	0.05443
61.852	0.06324	61.708	0.05443
61.852	0.06381	61.708	0.05442
61.852	0.06299	61.709	0.05441
61.853	0.06305	61.709	0.0544
61.853	0.0631	61.709	0.05439
61.853	0.06314	61.709	0.05437
61.854	0.06319	61.710	0.05436
61.854	0.06323	61.710	0.05435
61.854	0.06326	61.710	0.05433
61.854	0.06329	61.710	0.05432
61.855	0.06331	61.711	0.0543
61.855	0.06333	61.711	0.05429

61.855	0.06335	61.711	0.05427
61.855	0.06336	61.711	0.05425
61.856	0.06336	61.712	0.05423
61.856	0.06336	61.712	0.05421
61.856	0.06336	61.712	0.05419
61.856	0.06336	61.712	0.05417
61.857	0.0634	61.713	0.05415
61.857	0.06344	61.713	0.05413
61.857	0.06347	61.713	0.0541
61.857	0.0635	61.714	0.05408
61.858	0.06352	61.714	0.05406
61.858	0.06353	61.714	0.05403
61.858	0.06354	61.714	0.05401
61.858	0.06355	61.715	0.05398
61.859	0.06354	61.715	0.05395
61.859	0.06353	61.715	0.05393
61.859	0.06352	61.715	0.0539
61.860	0.0635	61.716	0.05387
61.860	0.06347	61.716	0.05384
61.860	0.06343	61.716	0.05381
61.860	0.06339	61.716	0.05378
61.861	0.06335	61.717	0.05375
61.861	0.0633	61.717	0.05372
61.861	0.06324	61.717	0.05369
61.861	0.06317	61.717	0.05366
61.862	0.0631	61.718	0.05363
61.862	0.06303	61.718	0.0536
61.862	0.06294	61.718	0.05357
61.862	0.06286	61.718	0.05354
61.863	0.06276	61.719	0.0535
61.863	0.06267	61.719	0.05347
61.863	0.06256	61.719	0.05344
61.863	0.06245	61.719	0.0534
61.864	0.06234	61.720	0.05338
61.864	0.06222	61.720	0.05339
61.864	0.0621	61.720	0.0534
61.865	0.06197	61.720	0.0534
61.865	0.06184	61.721	0.0534
61.865	0.06177	61.721	0.05341
61.865	0.06173	61.721	0.05341
61.866	0.06169	61.721	0.05341
61.866	0.06164	61.722	0.05341
61.866	0.06159	61.722	0.05341
61.866	0.06153	61.722	0.05341
61.867	0.06148	61.723	0.05341
61.867	0.06142	61.723	0.05341
61.867	0.06135	61.723	0.0534
61.867	0.06128	61.723	0.0534
61.868	0.06121	61.724	0.05339
61.868	0.06114	61.724	0.05339
61.868	0.06107	61.724	0.05338
61.869	0.06099	61.724	0.05337
61.869	0.06091	61.725	0.05337

61.869	0.06083	61.725	0.05336
61.869	0.06074	61.725	0.05335
61.870	0.06066	61.725	0.05334
61.870	0.06057	61.726	0.05333
61.870	0.06048	61.726	0.05332
61.870	0.06038	61.726	0.0533
61.871	0.06029	61.726	0.05329
61.871	0.06019	61.727	0.05328
61.871	0.0601	61.727	0.05326
61.871	0.06	61.727	0.05325
61.872	0.0599	61.727	0.05324
61.872	0.05979	61.728	0.05322
61.872	0.05969	61.728	0.0532
61.873	0.05958	61.728	0.05319
61.873	0.05968	61.728	0.05317
61.873	0.05937	61.729	0.05315
61.873	0.05926	61.729	0.05313
61.874	0.05915	61.729	0.05311
61.874	0.05917	61.729	0.05309
61.874	0.05893	61.730	0.05307
61.874	0.05882	61.730	0.05305
61.875	0.05878	61.730	0.05303
61.875	0.0586	61.730	0.05301
61.875	0.05898	61.731	0.05298
61.875	0.05899	61.731	0.05296
61.876	0.05869	61.731	0.05294
61.876	0.05855	61.731	0.05291
61.876	0.05869	61.732	0.05289
61.877	0.05848	61.732	0.05286
61.877	0.05847	61.732	0.05284
61.877	0.05846	61.733	0.05281
61.877	0.05844	61.733	0.05278
61.878	0.05842	61.733	0.05276
61.878	0.0584	61.733	0.05273
61.878	0.05838	61.734	0.0527
61.878	0.05836	61.734	0.05267
61.879	0.05836	61.734	0.05264
61.879	0.05842	61.734	0.05262
61.879	0.05848	61.735	0.05259
61.880	0.05853	61.735	0.05256
61.880	0.05858	61.735	0.05253
61.880	0.05862	61.735	0.05249
61.880	0.05866	61.736	0.05246
61.881	0.0587	61.736	0.05243
61.881	0.05872	61.736	0.0524
61.881	0.05875	61.736	0.05237
61.881	0.05877	61.737	0.05233
61.882	0.05878	61.737	0.0523
61.882	0.05968	61.737	0.05227
61.882	0.05943	61.737	0.05223
61.882	0.05879	61.738	0.0522
61.883	0.0594	61.738	0.05216
61.883	0.05877	61.738	0.05213

61.883	0.05939	61.738	0.05209
61.884	0.05872	61.739	0.05206
61.884	0.05869	61.739	0.05202
61.884	0.05865	61.739	0.05199
61.884	0.05861	61.739	0.05195
61.885	0.05856	61.740	0.05191
61.885	0.05851	61.740	0.05187
61.885	0.05845	61.740	0.05184
61.885	0.05838	61.741	0.0518
61.886	0.05831	61.741	0.05176
61.886	0.05824	61.741	0.05172
61.886	0.05816	61.741	0.05168
61.887	0.05808	61.742	0.05164
61.887	0.05799	61.742	0.0516
61.887	0.0579	61.742	0.05156
61.887	0.0578	61.742	0.05152
61.888	0.0577	61.743	0.05148
61.888	0.05759	61.743	0.05144
61.888	0.05748	61.743	0.0514
61.888	0.05737	61.743	0.05135
61.889	0.05725	61.744	0.05131
61.889	0.05712	61.744	0.05127
61.889	0.057	61.744	0.05123
61.890	0.05687	61.744	0.05118
61.890	0.05673	61.745	0.05114
61.890	0.05659	61.745	0.0511
61.890	0.05645	61.745	0.05105
61.891	0.05631	61.745	0.05101
61.891	0.05617	61.746	0.05096
61.891	0.05626	61.746	0.05092
61.892	0.05622	61.746	0.05087
61.892	0.05572	61.746	0.05083
61.892	0.05619	61.747	0.05078
61.892	0.05653	61.747	0.05073
61.893	0.05537	61.747	0.05069
61.893	0.05528	61.748	0.05064
61.893	0.05519	61.748	0.05059
61.893	0.0551	61.748	0.05055
61.894	0.05503	61.748	0.0505
61.894	0.05497	61.749	0.05045
61.894	0.05491	61.749	0.0504
61.895	0.05485	61.749	0.05036
61.895	0.05479	61.749	0.05031
61.895	0.05473	61.750	0.05026
61.895	0.05467	61.750	0.05021
61.896	0.05491	61.750	0.05016
61.896	0.05533	61.750	0.05011
61.896	0.05529	61.751	0.05006
61.896	0.05479	61.751	0.05001
61.897	0.05437	61.751	0.0501
61.897	0.05432	61.751	0.05002
61.897	0.05426	61.752	0.05002
61.898	0.05421	61.752	0.05003

61.898	0.05416	61.752	0.05004
61.898	0.05412	61.753	0.05006
61.898	0.05408	61.753	0.05008
61.899	0.05403	61.753	0.0501
61.899	0.05476	61.753	0.05012
61.899	0.05468	61.754	0.05014
61.899	0.05407	61.754	0.05016
61.900	0.05386	61.754	0.05019
61.900	0.05382	61.754	0.05021
61.900	0.05442	61.755	0.05023
61.901	0.05461	61.755	0.05025
61.901	0.0548	61.755	0.05027
61.901	0.05387	61.755	0.05029
61.901	0.05416	61.756	0.05031
61.902	0.05397	61.756	0.05033
61.902	0.05401	61.756	0.05034
61.902	0.05405	61.756	0.05036
61.903	0.05409	61.757	0.05038
61.903	0.05413	61.757	0.0504
61.903	0.05417	61.757	0.05041
61.903	0.05421	61.758	0.05043
61.904	0.05428	61.758	0.05045
61.904	0.05435	61.758	0.05046
61.904	0.05441	61.758	0.05048
61.904	0.05447	61.759	0.05049
61.905	0.05453	61.759	0.0505
61.905	0.05458	61.759	0.05052
61.905	0.05463	61.759	0.05053
61.906	0.05467	61.760	0.05054
61.906	0.05471	61.760	0.05055
61.906	0.05475	61.760	0.05056
61.906	0.05478	61.761	0.05057
61.907	0.05481	61.761	0.05058
61.907	0.05483	61.761	0.05058
61.907	0.05485	61.761	0.05059
61.907	0.05487	61.762	0.0506
61.908	0.05488	61.762	0.0506
61.908	0.05489	61.762	0.0506
61.908	0.05489	61.762	0.05061
61.908	0.05489	61.763	0.05061
61.909	0.05488	61.763	0.05061
61.909	0.05487	61.763	0.05061
61.909	0.05486	61.763	0.05061
61.910	0.05485	61.764	0.05061
61.910	0.05484	61.764	0.05061
61.910	0.05482	61.764	0.0506
61.910	0.0548	61.765	0.0506
61.911	0.05477	61.765	0.05059
61.911	0.05474	61.765	0.05059
61.911	0.05471	61.765	0.05058
61.911	0.05467	61.766	0.05057
61.912	0.05463	61.766	0.05056
61.912	0.05458	61.766	0.05055

61.912	0.05453	61.766	0.05054
61.913	0.05448	61.767	0.05053
61.913	0.05442	61.767	0.05052
61.913	0.05436	61.767	0.05051
61.913	0.05429	61.768	0.05049
61.914	0.05422	61.768	0.05048
61.914	0.05415	61.768	0.05046
61.914	0.05407	61.768	0.05045
61.914	0.05399	61.769	0.05043
61.915	0.05391	61.769	0.05041
61.915	0.05383	61.769	0.05039
61.915	0.05389	61.769	0.05037
61.915	0.05364	61.770	0.05035
61.916	0.05355	61.770	0.05033
61.916	0.05345	61.770	0.05031
61.916	0.05336	61.770	0.05029
61.917	0.05329	61.771	0.05027
61.917	0.05366	61.771	0.05024
61.917	0.05305	61.771	0.05022
61.917	0.05294	61.772	0.05019
61.918	0.05283	61.772	0.05017
61.918	0.05272	61.772	0.05014
61.918	0.05262	61.772	0.05012
61.918	0.05251	61.773	0.05009
61.919	0.0524	61.773	0.05006
61.919	0.05238	61.773	0.05004
61.919	0.05219	61.773	0.05001
61.919	0.05208	61.774	0.04998
61.920	0.05197	61.774	0.04995
61.920	0.05186	61.774	0.04992
61.920	0.05175	61.775	0.04989
61.920	0.05167	61.775	0.04986
61.921	0.05158	61.775	0.04983
61.921	0.0515	61.775	0.0498
61.921	0.05143	61.776	0.04977
61.922	0.05194	61.776	0.04974
61.922	0.05206	61.776	0.04971
61.922	0.05127	61.776	0.04968
61.922	0.05219	61.777	0.04965
61.923	0.0521	61.777	0.04962
61.923	0.05257	61.777	0.04958
61.923	0.05156	61.778	0.04955
61.923	0.05098	61.778	0.04952
61.924	0.05091	61.778	0.04949
61.924	0.05172	61.778	0.04946
61.924	0.05202	61.779	0.04942
61.924	0.05211	61.779	0.04939
61.925	0.05224	61.779	0.04936
61.925	0.05108	61.779	0.04933
61.925	0.0505	61.780	0.04929
61.925	0.05052	61.780	0.04926
61.926	0.05197	61.780	0.04923
61.926	0.05205	61.781	0.0492

61.926	0.05144	61.781	0.04917
61.927	0.05207	61.781	0.04913
61.927	0.05224	61.781	0.0491
61.927	0.05087	61.782	0.04907
61.927	0.05077	61.782	0.04904
61.928	0.05177	61.782	0.04901
61.928	0.0518	61.782	0.04899
61.928	0.05087	61.783	0.04897
61.928	0.0509	61.783	0.04896
61.929	0.0513	61.783	0.04894
61.929	0.05144	61.784	0.04892
61.929	0.05169	61.784	0.0489
61.929	0.05213	61.784	0.04888
61.930	0.05157	61.784	0.04886
61.930	0.05111	61.785	0.04884
61.930	0.05209	61.785	0.04882
61.930	0.0511	61.785	0.0488
61.931	0.05112	61.785	0.04877
61.931	0.05107	61.786	0.04875
61.931	0.05108	61.786	0.04872
61.932	0.05108	61.786	0.04869
61.932	0.05107	61.787	0.04867
61.932	0.05106	61.787	0.04864
61.932	0.05114	61.787	0.04861
61.933	0.05174	61.787	0.04858
61.933	0.05185	61.788	0.04855
61.933	0.051	61.788	0.04852
61.933	0.05098	61.788	0.04849
61.934	0.05111	61.789	0.04845
61.934	0.05122	61.789	0.04842
61.934	0.05088	61.789	0.04839
61.934	0.05084	61.789	0.04835
61.935	0.0508	61.790	0.04832
61.935	0.05077	61.790	0.04828
61.935	0.05092	61.790	0.04825
61.935	0.05065	61.791	0.04822
61.936	0.0506	61.791	0.04819
61.936	0.05054	61.791	0.04816
61.936	0.05071	61.791	0.04813
61.936	0.05083	61.792	0.0481
61.937	0.05034	61.792	0.04806
61.937	0.05027	61.792	0.04804
61.937	0.0502	61.792	0.04801
61.938	0.05012	61.793	0.04799
61.938	0.05004	61.793	0.04797
61.938	0.04996	61.793	0.04795
61.938	0.04987	61.794	0.04792
61.939	0.04983	61.794	0.0479
61.939	0.04998	61.794	0.04788
61.939	0.05048	61.794	0.04787
61.939	0.05044	61.795	0.04786
61.940	0.05081	61.795	0.04784
61.940	0.05072	61.795	0.04783

61.940	0.04989	61.796	0.04782
61.940	0.0491	61.796	0.0478
61.941	0.04899	61.796	0.04779
61.941	0.04889	61.796	0.04778
61.941	0.04928	61.797	0.04776
61.941	0.04939	61.797	0.04775
61.942	0.04912	61.797	0.04773
61.942	0.04929	61.798	0.04772
61.942	0.04833	61.798	0.0477
61.942	0.04893	61.798	0.04768
61.943	0.04907	61.798	0.04767
61.943	0.04856	61.799	0.04765
61.943	0.04943	61.799	0.04763
61.944	0.04961	61.799	0.04763
61.944	0.04852	61.800	0.04765
61.944	0.04886	61.800	0.04767
61.944	0.04853	61.800	0.04769
61.945	0.04879	61.800	0.0477
61.945	0.0491	61.801	0.04772
61.945	0.0484	61.801	0.04774
61.945	0.04881	61.801	0.04775
61.946	0.04784	61.802	0.04777
61.946	0.04812	61.802	0.04779
61.946	0.04846	61.802	0.0478
61.946	0.04775	61.802	0.04781
61.947	0.04804	61.803	0.04783
61.947	0.04796	61.803	0.04784
61.947	0.04777	61.803	0.04785
61.947	0.04834	61.804	0.04786
61.948	0.04823	61.804	0.04787
61.948	0.04741	61.804	0.04788
61.948	0.04764	61.804	0.04789
61.948	0.04733	61.805	0.0479
61.949	0.04719	61.805	0.04791
61.949	0.04732	61.805	0.04792
61.949	0.04741	61.806	0.04792
61.949	0.04771	61.806	0.04793
61.950	0.04792	61.806	0.04794
61.950	0.04697	61.806	0.04794
61.950	0.04698	61.807	0.04794
61.950	0.047	61.807	0.04795
61.951	0.04737	61.807	0.04795
61.951	0.04752	61.808	0.04795
61.951	0.04703	61.808	0.04795
61.952	0.04731	61.808	0.04795
61.952	0.04705	61.809	0.04795
61.952	0.04706	61.809	0.04795
61.952	0.04707	61.809	0.04794
61.953	0.04731	61.809	0.04794
61.953	0.04709	61.810	0.04793
61.953	0.0471	61.810	0.04793
61.953	0.04711	61.810	0.04792
61.954	0.04712	61.811	0.04791

61.954	0.04713	61.811	0.0479
61.954	0.04714	61.811	0.04789
61.954	0.04715	61.811	0.04788
61.955	0.04716	61.812	0.04787
61.955	0.04717	61.812	0.04786
61.955	0.04718	61.812	0.04785
61.955	0.04719	61.813	0.04783
61.956	0.0472	61.813	0.04782
61.956	0.04721	61.813	0.0478
61.956	0.04722	61.813	0.04778
61.956	0.04723	61.814	0.04776
61.957	0.04724	61.814	0.04774
61.957	0.04725	61.814	0.04772
61.957	0.04725	61.815	0.0477
61.957	0.04726	61.815	0.04768
61.958	0.04727	61.815	0.04766
61.958	0.04728	61.815	0.04763
61.958	0.04729	61.816	0.04761
61.958	0.04729	61.816	0.04758
61.959	0.0473	61.816	0.04756
61.959	0.04731	61.817	0.04753
61.959	0.04732	61.817	0.0475
61.959	0.04732	61.817	0.04747
61.960	0.04733	61.817	0.04744
61.960	0.04734	61.818	0.04741
61.960	0.04735	61.818	0.04738
61.960	0.04735	61.818	0.04734
61.961	0.04736	61.819	0.04731
61.961	0.04737	61.819	0.04728
61.961	0.04737	61.819	0.04724
61.961	0.04738	61.819	0.04721
61.962	0.04739	61.820	0.04717
61.962	0.04739	61.820	0.04713
61.962	0.0474	61.820	0.04709
61.962	0.04741	61.821	0.04705
61.963	0.04741	61.821	0.04702
61.963	0.04742	61.821	0.04701
61.963	0.04743	61.821	0.04703
61.963	0.04743	61.822	0.04704
61.964	0.04744	61.822	0.04705
61.964	0.04744	61.822	0.04706
61.964	0.04745	61.823	0.04707
61.964	0.04745	61.823	0.04707
61.965	0.04745	61.823	0.04708
61.965	0.04746	61.823	0.04709
61.965	0.04746	61.824	0.0471
61.965	0.04746	61.824	0.0471
61.966	0.04746	61.824	0.04711
61.966	0.04747	61.825	0.04711
61.966	0.04747	61.825	0.04711
61.966	0.04747	61.825	0.04712
61.967	0.04747	61.825	0.04712
61.967	0.04747	61.826	0.04712

61.967	0.04748	61.826	0.04712
61.967	0.04748	61.826	0.04712
61.968	0.04748	61.827	0.04712
61.968	0.04748	61.827	0.04712
61.968	0.04748	61.827	0.04711
61.968	0.04748	61.827	0.04711
61.969	0.04748	61.828	0.04711
61.969	0.04748	61.828	0.0471
61.969	0.04748	61.828	0.0471
61.969	0.04748	61.829	0.04709
61.970	0.04748	61.829	0.04708
61.970	0.04747	61.829	0.04707
61.970	0.04747	61.829	0.04706
61.970	0.04747	61.830	0.04705
61.971	0.04746	61.830	0.04704
61.971	0.04746	61.830	0.04703
61.971	0.04746	61.831	0.04702
61.971	0.04745	61.831	0.04701
61.972	0.04745	61.831	0.04699
61.972	0.04744	61.831	0.04698
61.972	0.04744	61.832	0.04696
61.972	0.04743	61.832	0.04694
61.973	0.04742	61.832	0.04693
61.973	0.04742	61.833	0.04691
61.973	0.04741	61.833	0.04689
61.973	0.0474	61.833	0.04687
61.974	0.0474	61.833	0.04685
61.974	0.04739	61.834	0.04683
61.974	0.04738	61.834	0.04681
61.974	0.04737	61.834	0.04679
61.975	0.04736	61.835	0.04676
61.975	0.04735	61.835	0.04674
61.975	0.04733	61.835	0.04671
61.975	0.04732	61.835	0.04669
61.976	0.04731	61.836	0.04666
61.976	0.04729	61.836	0.04664
61.976	0.04728	61.836	0.04661
61.976	0.04727	61.837	0.04658
61.977	0.04725	61.837	0.04655
61.977	0.04724	61.837	0.04652
61.977	0.04722	61.837	0.04649
61.977	0.04721	61.838	0.04646
61.978	0.04719	61.838	0.04643
61.978	0.04718	61.838	0.0464
61.978	0.04716	61.839	0.04637
61.978	0.04714	61.839	0.04633
61.979	0.04712	61.839	0.0463
61.979	0.04711	61.839	0.04627
61.979	0.04709	61.840	0.04623
61.979	0.04707	61.840	0.0462
61.980	0.04705	61.840	0.04616
61.980	0.04703	61.841	0.04613
61.980	0.04701	61.841	0.04609

61.980	0.04699	61.841	0.04605
61.981	0.04697	61.841	0.04602
61.981	0.04695	61.842	0.04598
61.981	0.04693	61.842	0.04594
61.981	0.04691	61.842	0.0459
61.982	0.04688	61.842	0.04586
61.982	0.04686	61.843	0.04583
61.982	0.04684	61.843	0.04579
61.982	0.04681	61.843	0.04575
61.983	0.04679	61.844	0.04571
61.983	0.04677	61.844	0.04567
61.983	0.04674	61.844	0.04562
61.983	0.04672	61.844	0.04558
61.984	0.0467	61.845	0.04554
61.984	0.04667	61.845	0.0455
61.984	0.04664	61.845	0.04546
61.984	0.04662	61.846	0.04542
61.985	0.04659	61.846	0.04537
61.985	0.04657	61.846	0.04533
61.985	0.04654	61.846	0.04529
61.985	0.04651	61.847	0.04524
61.986	0.04649	61.847	0.0452
61.986	0.04646	61.847	0.04516
61.986	0.04643	61.848	0.04511
61.986	0.0464	61.848	0.04507
61.987	0.04638	61.848	0.04502
61.987	0.04635	61.848	0.04498
61.987	0.04632	61.849	0.04493
61.987	0.04629	61.849	0.04491
61.988	0.04626	61.849	0.04488
61.988	0.04624	61.850	0.04485
61.988	0.04621	61.850	0.04483
61.988	0.04618	61.850	0.0448
61.989	0.04615	61.850	0.04477
61.989	0.04611	61.851	0.04475
61.989	0.04608	61.851	0.04473
61.989	0.04605	61.851	0.04472
61.990	0.04602	61.852	0.0447
61.990	0.04599	61.852	0.04468
61.990	0.04595	61.852	0.04466
61.990	0.04592	61.852	0.04464
61.991	0.04589	61.853	0.04462
61.991	0.04586	61.853	0.0446
61.991	0.04582	61.853	0.04458
61.991	0.04579	61.854	0.04456
61.992	0.04576	61.854	0.04454
61.992	0.04572	61.854	0.04452
61.992	0.04569	61.854	0.0445
61.992	0.04566	61.855	0.04448
61.993	0.04562	61.855	0.04446
61.993	0.04559	61.855	0.04444
61.993	0.04555	61.855	0.04442
61.993	0.04552	61.856	0.0444

61.994	0.04548	61.856	0.04438
61.994	0.04544	61.856	0.04436
61.994	0.04541	61.857	0.04434
61.995	0.04537	61.857	0.04431
61.995	0.04534	61.857	0.04429
61.995	0.0453	61.857	0.04427
61.995	0.04526	61.858	0.04425
61.996	0.04523	61.858	0.04422
61.996	0.04519	61.858	0.0442
61.996	0.04515	61.859	0.04417
61.996	0.04512	61.859	0.04415
61.997	0.04508	61.859	0.04412
61.997	0.04504	61.859	0.0441
61.997	0.045	61.860	0.04407
61.997	0.04497	61.860	0.04405
61.998	0.04493	61.860	0.04402
61.998	0.04489	61.861	0.04399
61.998	0.04485	61.861	0.04396
61.998	0.04481	61.861	0.04394
61.999	0.04478	61.861	0.04391
61.999	0.04474	61.862	0.04388
61.999	0.0447	61.862	0.04385
61.999	0.04466	61.862	0.04382
62.000	0.04462	61.863	0.04379
62.000	0.04458	61.863	0.04376
62.000	0.04454	61.863	0.04372
62.000	0.0445	61.863	0.04369
62.001	0.04447	61.864	0.04366
62.001	0.04443	61.864	0.04362
62.001	0.04439	61.864	0.04359
62.001	0.04435	61.865	0.04355
62.002	0.04431	61.865	0.04352
62.002	0.04427	61.865	0.04348
62.002	0.04423	61.865	0.04344
62.002	0.04419	61.866	0.04341
62.003	0.04415	61.866	0.04337
62.003	0.04411	61.866	0.04333
62.003	0.04407	61.867	0.04329
62.003	0.04402	61.867	0.04325
62.004	0.04398	61.867	0.0432
62.004	0.04394	61.867	0.04316
62.004	0.0439	61.868	0.04312
62.005	0.04386	61.868	0.04307
62.005	0.04382	61.868	0.04303
62.005	0.04377	61.868	0.04298
62.005	0.04373	61.869	0.04294
62.006	0.04369	61.869	0.04289
62.006	0.04365	61.869	0.04284
62.006	0.0436	61.870	0.04279
62.006	0.04356	61.870	0.04274
62.007	0.04352	61.870	0.04269
62.007	0.04348	61.870	0.04264
62.007	0.04343	61.871	0.04259

62.007	0.04339	61.871	0.04253
62.008	0.04335	61.871	0.04248
62.008	0.0433	61.872	0.04242
62.008	0.04326	61.872	0.04237
62.008	0.04322	61.872	0.04231
62.009	0.04317	61.872	0.04225
62.009	0.04313	61.873	0.04219
62.009	0.04309	61.873	0.04213
62.009	0.04304	61.873	0.04207
62.010	0.043	61.874	0.04201
62.010	0.04296	61.874	0.04195
62.010	0.04291	61.874	0.04189
62.010	0.04287	61.875	0.04182
62.011	0.04283	61.875	0.04176
62.011	0.04278	61.875	0.04169
62.011	0.04274	61.875	0.04163
62.012	0.0427	61.876	0.04156
62.012	0.04265	61.876	0.04149
62.012	0.04261	61.876	0.04142
62.012	0.04257	61.877	0.04135
62.013	0.04252	61.877	0.04128
62.013	0.04248	61.877	0.04121
62.013	0.04243	61.877	0.04114
62.013	0.04239	61.878	0.04107
62.014	0.04235	61.878	0.041
62.014	0.0423	61.878	0.04092
62.014	0.04226	61.879	0.04085
62.014	0.04222	61.879	0.04077
62.015	0.04218	61.879	0.0407
62.015	0.04213	61.879	0.04062
62.015	0.04209	61.880	0.04054
62.015	0.04205	61.880	0.04047
62.016	0.04201	61.880	0.04039
62.016	0.04196	61.881	0.04031
62.016	0.04192	61.881	0.04023
62.016	0.04188	61.881	0.04015
62.017	0.04184	61.881	0.04007
62.017	0.0418	61.882	0.03999
62.017	0.04176	61.882	0.03991
62.018	0.04172	61.882	0.03983
62.018	0.04168	61.883	0.03975
62.018	0.04163	61.883	0.03966
62.018	0.04159	61.883	0.03958
62.019	0.04155	61.883	0.0395
62.019	0.04151	61.884	0.03941
62.019	0.04147	61.884	0.03933
62.019	0.04143	61.884	0.03924
62.020	0.04139	61.885	0.03916
62.020	0.04135	61.885	0.03907
62.020	0.04131	61.885	0.03899
62.020	0.04127	61.886	0.0389
62.021	0.04123	61.886	0.03882
62.021	0.04119	61.886	0.03873

62.021	0.04116	61.886	0.03864
62.021	0.04112	61.887	0.03856
62.022	0.04108	61.887	0.03847
62.022	0.04104	61.887	0.03838
62.022	0.041	61.888	0.0383
62.022	0.04097	61.888	0.03821
62.023	0.04093	61.888	0.03813
62.023	0.04089	61.888	0.03806
62.023	0.04085	61.889	0.03799
62.024	0.04082	61.889	0.03792
62.024	0.04078	61.889	0.03785
62.024	0.04075	61.890	0.03778
62.024	0.04071	61.890	0.03772
62.025	0.04068	61.890	0.03765
62.025	0.04064	61.891	0.03758
62.025	0.04061	61.891	0.03751
62.025	0.04058	61.891	0.03745
62.026	0.04054	61.891	0.03738
62.026	0.04051	61.892	0.03732
62.026	0.04048	61.892	0.03726
62.026	0.04044	61.892	0.0372
62.027	0.04041	61.893	0.03715
62.027	0.04038	61.893	0.03709
62.027	0.04035	61.893	0.03703
62.027	0.04031	61.894	0.03697
62.028	0.04028	61.894	0.03694
62.028	0.04025	61.894	0.0369
62.028	0.04022	61.894	0.03686
62.029	0.04019	61.895	0.03683
62.029	0.04016	61.895	0.03679
62.029	0.04013	61.895	0.03675
62.029	0.0401	61.896	0.03671
62.030	0.04007	61.896	0.03667
62.030	0.04005	61.896	0.03663
62.030	0.04002	61.897	0.03659
62.030	0.03999	61.897	0.03655
62.031	0.03997	61.897	0.03651
62.031	0.03996	61.897	0.03646
62.031	0.03994	61.898	0.03642
62.031	0.03993	61.898	0.03637
62.032	0.03992	61.898	0.03633
62.032	0.0399	61.899	0.03628
62.032	0.03989	61.899	0.03624
62.032	0.03987	61.899	0.03619
62.033	0.03985	61.900	0.03614
62.033	0.03984	61.900	0.03609
62.033	0.03982	61.900	0.03604
62.034	0.0398	61.900	0.03599
62.034	0.03978	61.901	0.03596
62.034	0.03976	61.901	0.03593
62.034	0.03974	61.901	0.0359
62.035	0.03972	61.902	0.03588
62.035	0.0397	61.902	0.03588

62.035	0.03968	61.902	0.03587
62.035	0.03965	61.903	0.03587
62.036	0.03963	61.903	0.03586
62.036	0.03961	61.903	0.03586
62.036	0.03958	61.903	0.03585
62.036	0.03956	61.904	0.03584
62.037	0.03953	61.904	0.03584
62.037	0.0395	61.904	0.03583
62.037	0.03948	61.905	0.03582
62.037	0.03945	61.905	0.03581
62.038	0.03942	61.905	0.0358
62.038	0.03939	61.906	0.03579
62.038	0.03936	61.906	0.03577
62.039	0.03933	61.906	0.03576
62.039	0.0393	61.906	0.03575
62.039	0.03927	61.907	0.03573
62.039	0.03924	61.907	0.03572
62.040	0.03921	61.907	0.0357
62.040	0.03918	61.908	0.03568
62.040	0.03915	61.908	0.03567
62.040	0.03913	61.908	0.03565
62.041	0.03911	61.909	0.03563
62.041	0.03909	61.909	0.03561
62.041	0.03907	61.909	0.03559
62.041	0.03905	61.910	0.03557
62.042	0.03904	61.910	0.03555
62.042	0.03902	61.910	0.03553
62.042	0.039	61.910	0.0355
62.043	0.03898	61.911	0.03548
62.043	0.03896	61.911	0.03545
62.043	0.03894	61.911	0.03543
62.043	0.03892	61.912	0.0354
62.044	0.03891	61.912	0.03538
62.044	0.03889	61.912	0.03535
62.044	0.03888	61.913	0.03532
62.044	0.03886	61.913	0.03529
62.045	0.03885	61.913	0.03526
62.045	0.03883	61.913	0.03523
62.045	0.03882	61.914	0.0352
62.045	0.03881	61.914	0.03517
62.046	0.03879	61.914	0.03514
62.046	0.03878	61.915	0.0351
62.046	0.03876	61.915	0.03507
62.047	0.03875	61.915	0.03504
62.047	0.03874	61.916	0.035
62.047	0.03872	61.916	0.03496
62.047	0.03871	61.916	0.03493
62.048	0.03869	61.917	0.03489
62.048	0.03868	61.917	0.03485
62.048	0.03867	61.917	0.03482
62.048	0.03865	61.918	0.03478
62.049	0.03864	61.918	0.03474
62.049	0.03863	61.918	0.0347

62.049	0.03861	61.918	0.03466
62.049	0.0386	61.919	0.03462
62.050	0.03858	61.919	0.03457
62.050	0.03857	61.919	0.03453
62.050	0.03855	61.920	0.03449
62.051	0.03854	61.920	0.03445
62.051	0.03853	61.920	0.0344
62.051	0.03851	61.921	0.03436
62.051	0.0385	61.921	0.03432
62.052	0.03848	61.921	0.03427
62.052	0.03847	61.922	0.03423
62.052	0.03845	61.922	0.03418
62.052	0.03844	61.922	0.03414
62.053	0.03842	61.922	0.03409
62.053	0.03841	61.923	0.03404
62.053	0.0384	61.923	0.034
62.053	0.03838	61.923	0.03395
62.054	0.03837	61.924	0.0339
62.054	0.03835	61.924	0.03386
62.054	0.03834	61.924	0.03381
62.055	0.03833	61.925	0.03376
62.055	0.03831	61.925	0.03371
62.055	0.0383	61.925	0.03367
62.055	0.03829	61.926	0.03362
62.056	0.03827	61.926	0.03357
62.056	0.03826	61.926	0.03352
62.056	0.03825	61.927	0.03347
62.056	0.03823	61.927	0.03342
62.057	0.03822	61.927	0.03338
62.057	0.0382	61.927	0.03333
62.057	0.03819	61.928	0.03328
62.057	0.03818	61.928	0.03323
62.058	0.03816	61.928	0.03318
62.058	0.03815	61.929	0.03313
62.058	0.03814	61.929	0.03308
62.059	0.03812	61.929	0.03303
62.059	0.03811	61.930	0.03298
62.059	0.03809	61.930	0.03293
62.059	0.03808	61.930	0.03288
62.060	0.03807	61.931	0.03283
62.060	0.03805	61.931	0.03278
62.060	0.03804	61.931	0.03273
62.060	0.03802	61.932	0.03268
62.061	0.03801	61.932	0.03263
62.061	0.03799	61.932	0.03258
62.061	0.03798	61.932	0.03253
62.062	0.03796	61.933	0.03247
62.062	0.03795	61.933	0.03242
62.062	0.03794	61.933	0.03237
62.062	0.03792	61.934	0.03232
62.063	0.03791	61.934	0.03227
62.063	0.03789	61.934	0.03222
62.063	0.03788	61.935	0.03217

62.063	0.03786	61.935	0.03212
62.064	0.03785	61.935	0.03208
62.064	0.03783	61.936	0.03204
62.064	0.03782	61.936	0.03199
62.064	0.0378	61.936	0.03224
62.065	0.03779	61.937	0.03212
62.065	0.03777	61.937	0.03187
62.065	0.03776	61.937	0.03183
62.066	0.03774	61.937	0.03178
62.066	0.03773	61.938	0.03192
62.066	0.03771	61.938	0.03185
62.066	0.0377	61.938	0.03177
62.067	0.03768	61.939	0.03174
62.067	0.03766	61.939	0.03211
62.067	0.03765	61.939	0.03198
62.067	0.03763	61.940	0.03207
62.068	0.03762	61.940	0.0324
62.068	0.0376	61.940	0.03244
62.068	0.03758	61.941	0.0319
62.069	0.03757	61.941	0.03201
62.069	0.03755	61.941	0.03188
62.069	0.03753	61.942	0.03183
62.069	0.03752	61.942	0.03193
62.070	0.0375	61.942	0.0319
62.070	0.03748	61.943	0.03169
62.070	0.03747	61.943	0.03251
62.070	0.03745	61.943	0.03239
62.071	0.03743	61.944	0.03201
62.071	0.03741	61.944	0.0325
62.071	0.0374	61.944	0.03236
62.072	0.03738	61.945	0.03185
62.072	0.03736	61.945	0.03217
62.072	0.03734	61.945	0.03225
62.072	0.03732	61.945	0.03336
62.073	0.03731	61.946	0.0332
62.073	0.03729	61.946	0.03232
62.073	0.03727	61.946	0.03204
62.073	0.03725	61.947	0.03231
62.074	0.03723	61.947	0.03213
62.074	0.03721	61.947	0.03222
62.074	0.03719	61.948	0.03316
62.075	0.03717	61.948	0.03222
62.075	0.03715	61.948	0.0331
62.075	0.03713	61.949	0.03269
62.075	0.03711	61.949	0.03232
62.076	0.03709	61.949	0.03286
62.076	0.03707	61.950	0.03261
62.076	0.03705	61.950	0.03308
62.077	0.03703	61.950	0.03291
62.077	0.037	61.951	0.03269
62.077	0.03698	61.951	0.03326
62.077	0.03696	61.951	0.03304
62.078	0.03694	61.952	0.03313

62.078	0.03692	61.952	0.03357
62.078	0.03689	61.952	0.0334
62.078	0.03687	61.953	0.03286
62.079	0.03685	61.953	0.03351
62.079	0.03682	61.953	0.03336
62.079	0.0368	61.953	0.03346
62.080	0.03677	61.954	0.0333
62.080	0.03675	61.954	0.03322
62.080	0.03672	61.954	0.03309
62.080	0.0367	61.955	0.0339
62.081	0.03667	61.955	0.03372
62.081	0.03665	61.955	0.03325
62.081	0.03662	61.956	0.03318
62.082	0.0366	61.956	0.03412
62.082	0.03657	61.956	0.03443
62.082	0.03655	61.957	0.03331
62.082	0.03652	61.957	0.03303
62.083	0.03649	61.957	0.03352
62.083	0.03646	61.958	0.03383
62.083	0.03644	61.958	0.03401
62.084	0.03641	61.958	0.03373
62.084	0.03638	61.959	0.03413
62.084	0.03635	61.959	0.03414
62.084	0.03633	61.959	0.03378
62.085	0.0363	61.960	0.0342
62.085	0.03627	61.960	0.03407
62.085	0.03624	61.960	0.03339
62.085	0.03621	61.961	0.03425
62.086	0.03618	61.961	0.0341
62.086	0.03615	61.961	0.03336
62.086	0.03612	61.962	0.03353
62.087	0.03609	61.962	0.03416
62.087	0.03606	61.962	0.03408
62.087	0.03603	61.963	0.03426
62.087	0.036	61.963	0.03402
62.088	0.03597	61.963	0.03463
62.088	0.03594	61.964	0.03403
62.088	0.03591	61.964	0.0345
62.089	0.03589	61.964	0.03417
62.089	0.03586	61.965	0.03447
62.089	0.03583	61.965	0.03418
62.089	0.03579	61.965	0.03412
62.090	0.03576	61.966	0.03375
62.090	0.03573	61.966	0.03389
62.090	0.0357	61.966	0.03454
62.091	0.03567	61.966	0.03426
62.091	0.03564	61.967	0.03386
62.091	0.03561	61.967	0.03389
62.091	0.03557	61.967	0.03392
62.092	0.03554	61.968	0.03394
62.092	0.03551	61.968	0.03413
62.092	0.03548	61.968	0.03399
62.093	0.03544	61.969	0.03401

62.093	0.03541	61.969	0.03409
62.093	0.0354	61.969	0.0341
62.094	0.03539	61.970	0.03408
62.094	0.03538	61.970	0.0341
62.094	0.03536	61.971	0.0343
62.094	0.03535	61.971	0.03421
62.095	0.03534	61.971	0.0346
62.095	0.03532	61.972	0.03424
62.095	0.03531	61.972	0.03433
62.096	0.0353	61.972	0.03448
62.096	0.03528	61.973	0.03492
62.096	0.03526	61.973	0.03428
62.096	0.03525	61.973	0.03435
62.097	0.03523	61.974	0.03435
62.097	0.03521	61.974	0.03462
62.097	0.0352	61.974	0.03493
62.098	0.03518	61.975	0.03458
62.098	0.03544	61.975	0.03463
62.098	0.03543	61.975	0.0345
62.098	0.03512	61.976	0.03453
62.099	0.0351	61.976	0.03456
62.099	0.03508	61.976	0.03459
62.099	0.03506	61.977	0.03471
62.100	0.03504	61.977	0.0347
62.100	0.03571	61.977	0.03475
62.100	0.03575	61.978	0.03468
62.101	0.03497	61.978	0.03485
62.101	0.03498	61.978	0.03472
62.101	0.03498	61.979	0.03474
62.101	0.03489	61.979	0.03523
62.102	0.03558	61.979	0.03485
62.102	0.03609	61.980	0.0348
62.102	0.03613	61.980	0.03481
62.103	0.03487	61.980	0.03507
62.103	0.03477	61.981	0.03484
62.103	0.03507	61.981	0.03485
62.104	0.03509	61.981	0.03487
62.104	0.03497	61.982	0.03488
62.104	0.03651	61.982	0.03489
62.104	0.03655	61.982	0.0349
62.105	0.03528	61.983	0.03491
62.105	0.03528	61.983	0.03491
62.105	0.0354	61.983	0.03492
62.106	0.03483	61.984	0.03493
62.106	0.03664	61.984	0.03493
62.106	0.03677	61.984	0.03493
62.106	0.03558	61.985	0.03494
62.107	0.03545	61.985	0.03494
62.107	0.03542	61.985	0.03494
62.107	0.03531	61.986	0.03518
62.108	0.03699	61.986	0.03524
62.108	0.0371	61.986	0.03494
62.108	0.03589	61.987	0.03494

62.109	0.03544	61.987	0.03494
62.109	0.03538	61.987	0.035
62.109	0.03567	61.988	0.03494
62.109	0.03746	61.988	0.0353
62.110	0.03742	61.988	0.03498
62.110	0.03664	61.989	0.03507
62.110	0.0362	61.989	0.03492
62.111	0.03713	61.989	0.03521
62.111	0.03736	61.990	0.03491
62.111	0.03811	61.990	0.0349
62.112	0.03634	61.990	0.03489
62.112	0.03749	61.991	0.03488
62.112	0.03802	61.991	0.03487
62.112	0.03766	61.992	0.03509
62.113	0.03844	61.992	0.03501
62.113	0.03767	61.992	0.03485
62.113	0.03695	61.993	0.03484
62.114	0.03782	61.993	0.03534
62.114	0.03777	61.993	0.03504
62.114	0.03725	61.994	0.03519
62.115	0.03734	61.994	0.035
62.115	0.03795	61.994	0.03517
62.115	0.03794	61.995	0.03507
62.115	0.03762	61.995	0.03476
62.116	0.03771	61.995	0.03475
62.116	0.03815	61.996	0.03473
62.116	0.03813	61.996	0.03472
62.117	0.03796	61.996	0.03488
62.117	0.03865	61.997	0.0347
62.117	0.03839	61.997	0.03489
62.118	0.03833	61.997	0.03467
62.118	0.03855	61.998	0.03466
62.118	0.03836	61.998	0.0349
62.118	0.03843	61.998	0.03464
62.119	0.03864	61.999	0.03469
62.119	0.03857	61.999	0.03471
62.119	0.0395	61.999	0.03548
62.120	0.03871	62.000	0.035
62.120	0.03938	62.000	0.03461
62.120	0.03883	62.000	0.03464
62.121	0.03889	62.001	0.03487
62.121	0.03895	62.001	0.03527
62.121	0.03901	62.001	0.03507
62.121	0.03906	62.002	0.03502
62.122	0.03911	62.002	0.03517
62.122	0.03916	62.002	0.03513
62.122	0.0392	62.003	0.03501
62.123	0.03925	62.003	0.0352
62.123	0.03929	62.003	0.03489
62.123	0.03933	62.004	0.03515
62.124	0.03937	62.004	0.03477
62.124	0.0394	62.004	0.03462
62.124	0.03943	62.005	0.03448

62.124	0.03946	62.005	0.03456
62.125	0.03949	62.005	0.03445
62.125	0.03952	62.006	0.03444
62.125	0.03954	62.006	0.03442
62.126	0.03956	62.007	0.03472
62.126	0.03958	62.007	0.03439
62.126	0.0396	62.007	0.03495
62.127	0.03962	62.008	0.03458
62.127	0.03963	62.008	0.03488
62.127	0.03964	62.008	0.03544
62.127	0.03966	62.009	0.03451
62.128	0.03966	62.009	0.03437
62.128	0.03967	62.009	0.03441
62.128	0.03968	62.010	0.03431
62.129	0.03968	62.010	0.03422
62.129	0.03968	62.010	0.03421
62.129	0.03968	62.011	0.03506
62.130	0.03968	62.011	0.03418
62.130	0.03967	62.011	0.03484
62.130	0.03967	62.012	0.03462
62.130	0.03966	62.012	0.03457
62.131	0.03965	62.012	0.03448
62.131	0.03964	62.013	0.03491
62.131	0.03963	62.013	0.03476
62.132	0.03962	62.013	0.03496
62.132	0.0396	62.014	0.03453
62.132	0.03958	62.014	0.03481
62.133	0.03957	62.014	0.03457
62.133	0.03955	62.015	0.03441
62.133	0.03953	62.015	0.0347
62.134	0.03951	62.016	0.03428
62.134	0.03948	62.016	0.034
62.134	0.03946	62.016	0.03398
62.134	0.03943	62.017	0.0349
62.135	0.03941	62.017	0.03462
62.135	0.03938	62.017	0.03509
62.135	0.03935	62.018	0.03427
62.136	0.03979	62.018	0.03477
62.136	0.03981	62.018	0.0347
62.136	0.03927	62.019	0.03425
62.137	0.03924	62.019	0.03385
62.137	0.03921	62.019	0.03389
62.137	0.03917	62.020	0.03381
62.138	0.03914	62.020	0.03475
62.138	0.03911	62.020	0.03408
62.138	0.03908	62.021	0.03504
62.138	0.03912	62.021	0.03437
62.139	0.03901	62.021	0.03371
62.139	0.03897	62.022	0.03475
62.139	0.03948	62.022	0.03453
62.140	0.03894	62.023	0.03371
62.140	0.03898	62.023	0.03369
62.140	0.03887	62.023	0.03433

62.141	0.03879	62.024	0.03411
62.141	0.03876	62.024	0.03368
62.141	0.03872	62.024	0.03471
62.142	0.03883	62.025	0.0345
62.142	0.03865	62.025	0.03404
62.142	0.03862	62.025	0.03366
62.143	0.0386	62.026	0.03395
62.143	0.03976	62.026	0.03365
62.143	0.03974	62.026	0.03364
62.143	0.03875	62.027	0.03363
62.144	0.03853	62.027	0.03456
62.144	0.03851	62.027	0.03448
62.144	0.0385	62.028	0.03404
62.145	0.03848	62.028	0.0337
62.145	0.03846	62.029	0.03357
62.145	0.03845	62.029	0.03409
62.146	0.03843	62.029	0.03386
62.146	0.03946	62.030	0.03395
62.146	0.0394	62.030	0.03381
62.147	0.03846	62.030	0.03349
62.147	0.03836	62.031	0.03347
62.147	0.03834	62.031	0.03373
62.148	0.03832	62.031	0.03398
62.148	0.03867	62.032	0.03345
62.148	0.03827	62.032	0.03428
62.148	0.03825	62.033	0.03385
62.149	0.03929	62.033	0.03396
62.149	0.03923	62.033	0.03403
62.149	0.03818	62.034	0.03349
62.150	0.03946	62.034	0.03439
62.150	0.03924	62.034	0.03322
62.150	0.03878	62.035	0.03406
62.151	0.03928	62.035	0.0338
62.151	0.03894	62.035	0.03403
62.151	0.03802	62.036	0.0331
62.152	0.03886	62.036	0.03417
62.152	0.03883	62.037	0.03382
62.152	0.03841	62.037	0.0338
62.153	0.03803	62.037	0.03361
62.153	0.03851	62.038	0.03298
62.153	0.03826	62.038	0.03289
62.154	0.03802	62.038	0.03365
62.154	0.03789	62.039	0.03333
62.154	0.0379	62.039	0.03278
62.155	0.03793	62.039	0.03338
62.155	0.0387	62.040	0.03358
62.155	0.03864	62.040	0.03364
62.156	0.03806	62.041	0.03334
62.156	0.03805	62.041	0.03283
62.156	0.03809	62.041	0.03365
62.157	0.03812	62.042	0.0335
62.157	0.03815	62.042	0.03307
62.157	0.03818	62.042	0.03332

62.157	0.03821	62.043	0.03299
62.158	0.03824	62.043	0.03341
62.158	0.03844	62.044	0.03282
62.158	0.03836	62.044	0.03338
62.159	0.03833	62.044	0.03318
62.159	0.03836	62.045	0.03347
62.159	0.03838	62.045	0.03288
62.160	0.03841	62.045	0.03226
62.160	0.03843	62.046	0.03301
62.160	0.03846	62.046	0.0325
62.161	0.03848	62.046	0.03201
62.161	0.0385	62.047	0.03238
62.161	0.03853	62.047	0.03307
62.162	0.03855	62.048	0.03269
62.162	0.03857	62.048	0.0322
62.162	0.03858	62.048	0.03291
62.163	0.0386	62.049	0.03307
62.163	0.03862	62.049	0.03361
62.163	0.03863	62.049	0.03295
62.164	0.03865	62.050	0.03316
62.164	0.03866	62.050	0.03284
62.164	0.03867	62.051	0.03225
62.165	0.03868	62.051	0.03259
62.165	0.03868	62.051	0.03204
62.165	0.03869	62.052	0.03274
62.166	0.03869	62.052	0.03217
62.166	0.0387	62.053	0.03259
62.166	0.0387	62.053	0.03285
62.167	0.0387	62.053	0.03277
62.167	0.0387	62.054	0.0332
62.167	0.03869	62.054	0.03287
62.168	0.03869	62.054	0.03274
62.168	0.03868	62.055	0.0326
62.168	0.03867	62.055	0.03203
62.169	0.03866	62.056	0.03279
62.169	0.03864	62.056	0.03235
62.169	0.03863	62.056	0.03252
62.170	0.03861	62.057	0.0319
62.170	0.03859	62.057	0.03203
62.170	0.03857	62.057	0.03247
62.171	0.03855	62.058	0.03187
62.171	0.03852	62.058	0.0326
62.171	0.03849	62.059	0.03193
62.172	0.03846	62.059	0.0314
62.172	0.03843	62.059	0.0312
62.172	0.0384	62.060	0.03252
62.173	0.03836	62.060	0.0324
62.173	0.03832	62.061	0.03201
62.173	0.03828	62.061	0.03192
62.174	0.03824	62.061	0.03144
62.174	0.03819	62.062	0.03204
62.174	0.03815	62.062	0.03217
62.175	0.0381	62.062	0.03209

62.175	0.03805	62.063	0.03207
62.175	0.0383	62.063	0.03219
62.176	0.03794	62.064	0.0318
62.176	0.03788	62.064	0.03208
62.176	0.03783	62.064	0.03221
62.177	0.03776	62.065	0.03215
62.177	0.0377	62.065	0.03177
62.177	0.03764	62.066	0.03148
62.178	0.03757	62.066	0.03219
62.178	0.0375	62.066	0.03167
62.178	0.03744	62.067	0.03192
62.179	0.03736	62.067	0.03148
62.179	0.03729	62.067	0.03106
62.179	0.0376	62.068	0.03079
62.180	0.03739	62.068	0.03188
62.180	0.03706	62.069	0.03149
62.180	0.03699	62.069	0.03161
62.181	0.0369	62.069	0.03117
62.181	0.03696	62.070	0.03083
62.181	0.03679	62.070	0.03141
62.182	0.03666	62.071	0.03122
62.182	0.03691	62.071	0.03165
62.182	0.03668	62.071	0.03124
62.183	0.0364	62.072	0.03102
62.183	0.03631	62.072	0.03186
62.183	0.03691	62.073	0.03161
62.184	0.03662	62.073	0.0314
62.184	0.03641	62.073	0.03148
62.184	0.03663	62.074	0.03152
62.185	0.03617	62.074	0.03143
62.185	0.0366	62.074	0.03155
62.185	0.03657	62.075	0.03119
62.186	0.036	62.075	0.03108
62.186	0.03582	62.076	0.03049
62.187	0.03539	62.076	0.0314
62.187	0.03529	62.076	0.03076
62.187	0.0352	62.077	0.03055
62.188	0.0351	62.077	0.03046
62.188	0.03501	62.078	0.03016
62.188	0.03496	62.078	0.03111
62.189	0.03481	62.078	0.03125
62.189	0.03525	62.079	0.03115
62.189	0.03497	62.079	0.03118
62.190	0.03543	62.080	0.0309
62.190	0.03522	62.080	0.0308
62.190	0.03466	62.080	0.03038
62.191	0.03535	62.081	0.03002
62.191	0.03542	62.081	0.03046
62.191	0.03468	62.082	0.03138
62.192	0.03425	62.082	0.03073
62.192	0.03499	62.082	0.03092
62.192	0.03472	62.083	0.03041
62.193	0.03502	62.083	0.03066

62.193	0.03479	62.083	0.03063
62.193	0.03413	62.084	0.03075
62.194	0.03393	62.084	0.03041
62.194	0.03433	62.085	0.03067
62.194	0.03495	62.085	0.03077
62.195	0.03459	62.085	0.03016
62.195	0.03428	62.086	0.03009
62.196	0.03428	62.086	0.03027
62.196	0.03411	62.087	0.02968
62.196	0.03439	62.087	0.03031
62.197	0.03401	62.087	0.03049
62.197	0.03397	62.088	0.03064
62.197	0.03408	62.088	0.03014
62.198	0.03451	62.089	0.03018
62.198	0.03429	62.089	0.02967
62.198	0.03396	62.089	0.03042
62.199	0.03371	62.090	0.03032
62.199	0.0335	62.090	0.03026
62.199	0.03372	62.090	0.03019
62.200	0.03409	62.091	0.03016
62.200	0.03403	62.091	0.02963
62.200	0.03402	62.092	0.02941
62.201	0.0337	62.092	0.02962
62.201	0.03402	62.092	0.02946
62.201	0.03389	62.093	0.02929
62.202	0.03418	62.093	0.0295
62.202	0.03412	62.094	0.02965
62.202	0.0343	62.094	0.02923
62.203	0.0341	62.094	0.03002
62.203	0.034	62.095	0.02962
62.204	0.0338	62.095	0.02938
62.204	0.03404	62.095	0.02962
62.204	0.03441	62.096	0.02908
62.205	0.03428	62.096	0.02917
62.205	0.03327	62.097	0.02941
62.205	0.0341	62.097	0.02958
62.206	0.03373	62.097	0.02957
62.206	0.0342	62.098	0.0291
62.206	0.03436	62.098	0.0299
62.207	0.03431	62.099	0.0293
62.207	0.03375	62.099	0.02876
62.207	0.03338	62.099	0.02889
62.208	0.03384	62.100	0.02942
62.208	0.03347	62.100	0.02962
62.208	0.03452	62.101	0.02973
62.209	0.03445	62.101	0.02915
62.209	0.03344	62.101	0.02925
62.209	0.03329	62.102	0.02887
62.210	0.03299	62.102	0.02951
62.210	0.03371	62.102	0.02928
62.210	0.0346	62.103	0.02922
62.211	0.03455	62.103	0.0288
62.211	0.03418	62.104	0.02849

62.212	0.03387	62.104	0.02894
62.212	0.03427	62.104	0.02943
62.212	0.03454	62.105	0.02933
62.213	0.0345	62.105	0.02927
62.213	0.03345	62.106	0.02898
62.213	0.03353	62.106	0.02876
62.214	0.03332	62.106	0.02838
62.214	0.03399	62.107	0.02861
62.214	0.03478	62.107	0.02906
62.215	0.03469	62.107	0.0286
62.215	0.03352	62.108	0.02914
62.215	0.03326	62.108	0.02908
62.216	0.0338	62.109	0.02882
62.216	0.03456	62.109	0.02843
62.216	0.03453	62.109	0.02888
62.217	0.03443	62.110	0.02905
62.217	0.03389	62.110	0.02868
62.217	0.03397	62.110	0.0289
62.218	0.03459	62.111	0.02898
62.218	0.03453	62.111	0.02846
62.218	0.03387	62.112	0.02863
62.219	0.03427	62.112	0.02847
62.219	0.03379	62.112	0.02875
62.220	0.03453	62.113	0.02863
62.220	0.03447	62.113	0.02873
62.220	0.03364	62.114	0.02867
62.221	0.0332	62.114	0.02876
62.221	0.03401	62.114	0.02854
62.221	0.03441	62.115	0.02851
62.222	0.03434	62.115	0.02846
62.222	0.03327	62.115	0.02889
62.222	0.03335	62.116	0.02905
62.223	0.03321	62.116	0.02807
62.223	0.03424	62.117	0.02878
62.223	0.03416	62.117	0.02851
62.224	0.03354	62.117	0.02859
62.224	0.03333	62.118	0.02827
62.224	0.0325	62.118	0.02801
62.225	0.03403	62.118	0.02849
62.225	0.03394	62.119	0.02821
62.225	0.03283	62.119	0.02789
62.226	0.03249	62.120	0.02905
62.226	0.03233	62.120	0.0285
62.226	0.03377	62.120	0.02862
62.227	0.03367	62.121	0.0281
62.227	0.03256	62.121	0.02829
62.228	0.03405	62.121	0.02847
62.228	0.0335	62.122	0.02851
62.228	0.03329	62.122	0.02874
62.229	0.03255	62.123	0.02798
62.229	0.03196	62.123	0.02814
62.229	0.03344	62.123	0.02824
62.230	0.03304	62.124	0.02812

62.230	0.03201	62.124	0.02811
62.230	0.03166	62.124	0.02815
62.231	0.03292	62.125	0.02772
62.231	0.03308	62.125	0.02817
62.231	0.03266	62.126	0.02857
62.232	0.03225	62.126	0.02839
62.232	0.03264	62.126	0.02805
62.232	0.03245	62.127	0.02881
62.233	0.03265	62.127	0.02799
62.233	0.03228	62.127	0.02757
62.233	0.03232	62.128	0.02832
62.234	0.03213	62.128	0.0276
62.234	0.03155	62.129	0.02858
62.234	0.03229	62.129	0.02838
62.235	0.03197	62.129	0.02785
62.235	0.03178	62.130	0.02781
62.236	0.03162	62.130	0.02788
62.236	0.03128	62.130	0.02755
62.236	0.03185	62.131	0.02753
62.237	0.03145	62.131	0.02775
62.237	0.03084	62.132	0.0273
62.237	0.03079	62.132	0.02738
62.238	0.0313	62.132	0.0272
62.238	0.03142	62.133	0.02694
62.238	0.03102	62.133	0.02743
62.239	0.03105	62.133	0.02718
62.239	0.03096	62.134	0.0276
62.239	0.03077	62.134	0.02724
62.240	0.03098	62.135	0.02732
62.240	0.03059	62.135	0.02746
62.240	0.0306	62.135	0.02798
62.241	0.03041	62.136	0.02752
62.241	0.03051	62.136	0.02762
62.241	0.03067	62.136	0.02754
62.242	0.03031	62.137	0.02711
62.242	0.03021	62.137	0.02733
62.242	0.03017	62.137	0.02779
62.243	0.02977	62.138	0.02738
62.243	0.02961	62.138	0.02744
62.244	0.03004	62.139	0.02742
62.244	0.02983	62.139	0.02734
62.244	0.0298	62.139	0.02698
62.245	0.02938	62.140	0.0267
62.245	0.02931	62.140	0.02683
62.245	0.02911	62.140	0.02663
62.246	0.02943	62.141	0.0268
62.246	0.02934	62.141	0.02722
62.246	0.02989	62.142	0.02719
62.247	0.02954	62.142	0.02704
62.247	0.02892	62.142	0.02682
62.247	0.02889	62.143	0.02709
62.248	0.02913	62.143	0.02759
62.248	0.02903	62.143	0.02717

62.248	0.02877	62.144	0.02713
62.249	0.029	62.144	0.02703
62.249	0.0287	62.145	0.02687
62.249	0.02884	62.145	0.02651
62.250	0.02873	62.145	0.02685
62.250	0.02868	62.146	0.02697
62.250	0.02917	62.146	0.02667
62.251	0.02884	62.146	0.02693
62.251	0.02863	62.147	0.02653
62.252	0.02872	62.147	0.02608
62.252	0.02917	62.147	0.02657
62.252	0.02884	62.148	0.02624
62.253	0.02849	62.148	0.02622
62.253	0.02838	62.149	0.0267
62.253	0.02897	62.149	0.02676
62.254	0.02865	62.149	0.0269
62.254	0.02832	62.150	0.02705
62.254	0.02832	62.150	0.02666
62.255	0.02828	62.150	0.02716
62.255	0.02878	62.151	0.02672
62.255	0.02855	62.151	0.02646
62.256	0.0286	62.152	0.02653
62.256	0.02862	62.152	0.02663
62.256	0.0284	62.152	0.0266
62.257	0.02828	62.153	0.02629
62.257	0.02829	62.153	0.02562
62.257	0.0283	62.153	0.02682
62.258	0.0283	62.154	0.0264
62.258	0.02831	62.154	0.02605
62.258	0.02838	62.154	0.02586
62.259	0.02832	62.155	0.02632
62.259	0.02832	62.155	0.02591
62.259	0.02833	62.156	0.02612
62.260	0.02833	62.156	0.02577
62.260	0.02833	62.156	0.02595
62.261	0.02834	62.157	0.0256
62.261	0.02834	62.157	0.0254
62.261	0.02834	62.157	0.02543
62.262	0.02834	62.158	0.02591
62.262	0.02835	62.158	0.0258
62.262	0.02835	62.159	0.02539
62.263	0.02835	62.159	0.02581
62.263	0.02835	62.159	0.02593
62.263	0.02834	62.160	0.02569
62.264	0.02834	62.160	0.0254
62.264	0.02834	62.160	0.02517
62.264	0.02834	62.161	0.0256
62.265	0.02833	62.161	0.0256
62.265	0.02833	62.161	0.02517
62.265	0.02833	62.162	0.02499
62.266	0.02832	62.162	0.0255
62.266	0.02831	62.163	0.025
62.266	0.0283	62.163	0.02518

62.267	0.02871	62.163	0.02586
62.267	0.02829	62.164	0.02542
62.267	0.02863	62.164	0.02524
62.268	0.02826	62.164	0.02515
62.268	0.02825	62.165	0.02483
62.268	0.02824	62.165	0.02508
62.269	0.02822	62.166	0.02522
62.269	0.02821	62.166	0.02478
62.270	0.02819	62.166	0.02494
62.270	0.02817	62.167	0.02492
62.270	0.02815	62.167	0.02502
62.271	0.02813	62.167	0.02471
62.271	0.02811	62.168	0.0251
62.271	0.02809	62.168	0.02542
62.272	0.02807	62.168	0.025
62.272	0.02804	62.169	0.02508
62.272	0.02801	62.169	0.02488
62.273	0.02824	62.170	0.02509
62.273	0.02811	62.170	0.02472
62.273	0.02802	62.170	0.02549
62.274	0.0279	62.171	0.02492
62.274	0.02787	62.171	0.0252
62.274	0.02783	62.171	0.02486
62.275	0.0278	62.172	0.0245
62.275	0.02778	62.172	0.02444
62.275	0.02775	62.173	0.02442
62.276	0.02773	62.173	0.02465
62.276	0.0277	62.173	0.02446
62.276	0.02767	62.174	0.02434
62.277	0.02764	62.174	0.02431
62.277	0.02761	62.174	0.02428
62.277	0.02758	62.175	0.0244
62.278	0.02755	62.175	0.02423
62.278	0.02751	62.175	0.0242
62.279	0.02748	62.176	0.02417
62.279	0.02744	62.176	0.02414
62.279	0.0274	62.177	0.02411
62.280	0.02736	62.177	0.02407
62.280	0.02732	62.177	0.02404
62.280	0.02737	62.178	0.02418
62.281	0.02725	62.178	0.02397
62.281	0.02718	62.178	0.02394
62.281	0.02713	62.179	0.0239
62.282	0.02708	62.179	0.02387
62.282	0.02727	62.180	0.02383
62.282	0.02703	62.180	0.02379
62.283	0.02693	62.180	0.02375
62.283	0.02687	62.181	0.02371
62.283	0.02709	62.181	0.02367
62.284	0.02694	62.181	0.02363
62.284	0.0267	62.182	0.02368
62.284	0.02728	62.182	0.02355
62.285	0.02659	62.183	0.02351

62.285	0.02713	62.183	0.02347
62.285	0.02668	62.183	0.02342
62.286	0.02666	62.184	0.02338
62.286	0.02658	62.184	0.02333
62.286	0.02628	62.184	0.02329
62.287	0.02622	62.185	0.02324
62.287	0.02615	62.185	0.02333
62.288	0.02613	62.186	0.02315
62.288	0.02607	62.186	0.0231
62.288	0.02606	62.186	0.02305
62.289	0.02606	62.187	0.023
62.289	0.02605	62.187	0.02295
62.289	0.02604	62.187	0.02311
62.290	0.02603	62.188	0.02285
62.290	0.02603	62.188	0.0228
62.290	0.02602	62.189	0.02274
62.291	0.02601	62.189	0.02269
62.291	0.026	62.189	0.02277
62.291	0.02599	62.190	0.02267
62.292	0.02598	62.190	0.02256
62.292	0.02597	62.190	0.02247
62.292	0.02596	62.191	0.02242
62.293	0.02594	62.191	0.02236
62.293	0.02593	62.192	0.02231
62.293	0.02592	62.192	0.02253
62.294	0.0259	62.192	0.02241
62.294	0.02589	62.193	0.02213
62.294	0.02587	62.193	0.02208
62.295	0.02586	62.194	0.02233
62.295	0.02584	62.194	0.02215
62.295	0.02582	62.194	0.02223
62.296	0.02581	62.195	0.02191
62.296	0.02579	62.195	0.02205
62.297	0.02577	62.195	0.02192
62.297	0.02575	62.196	0.02168
62.297	0.02574	62.196	0.02192
62.298	0.02572	62.197	0.02175
62.298	0.0257	62.197	0.02202
62.298	0.02568	62.197	0.02161
62.299	0.02566	62.198	0.02184
62.299	0.02564	62.198	0.02164
62.299	0.02562	62.198	0.02139
62.300	0.02559	62.199	0.02176
62.300	0.02557	62.199	0.02144
62.300	0.02555	62.200	0.02124
62.301	0.02552	62.200	0.02162
62.301	0.0255	62.200	0.02149
62.301	0.02547	62.201	0.02155
62.302	0.02545	62.201	0.02122
62.302	0.02543	62.202	0.02107
62.302	0.02541	62.202	0.02113
62.303	0.02538	62.202	0.02135
62.303	0.02536	62.203	0.02139

62.303	0.02534	62.203	0.02129
62.304	0.02531	62.203	0.02117
62.304	0.02529	62.204	0.021
62.304	0.02526	62.204	0.02067
62.305	0.02524	62.205	0.02093
62.305	0.02521	62.205	0.02076
62.306	0.02519	62.205	0.02095
62.306	0.02516	62.206	0.02097
62.306	0.02513	62.206	0.02063
62.307	0.0251	62.206	0.0207
62.307	0.02508	62.207	0.02067
62.307	0.02505	62.207	0.02077
62.308	0.02502	62.208	0.02049
62.308	0.02499	62.208	0.02061
62.308	0.02498	62.208	0.0206
62.309	0.02496	62.209	0.02035
62.309	0.02494	62.209	0.02064
62.309	0.02493	62.210	0.02029
62.310	0.02491	62.210	0.02052
62.310	0.02489	62.210	0.02042
62.310	0.02488	62.211	0.02049
62.311	0.02486	62.211	0.02043
62.311	0.02484	62.211	0.02037
62.311	0.02483	62.212	0.01982
62.312	0.02481	62.212	0.02044
62.312	0.02479	62.213	0.02017
62.312	0.02477	62.213	0.02016
62.313	0.02476	62.213	0.02006
62.313	0.02474	62.214	0.02014
62.314	0.02472	62.214	0.01979
62.314	0.0247	62.215	0.01966
62.314	0.02469	62.215	0.01955
62.315	0.02467	62.215	0.01989
62.315	0.02465	62.216	0.0197
62.315	0.02463	62.216	0.01979
62.316	0.02461	62.217	0.0197
62.316	0.02459	62.217	0.01952
62.316	0.02457	62.217	0.01952
62.317	0.02455	62.218	0.01972
62.317	0.02453	62.218	0.0194
62.317	0.02452	62.218	0.01961
62.318	0.0245	62.219	0.01933
62.318	0.02448	62.219	0.01908
62.318	0.02446	62.220	0.01937
62.319	0.02444	62.220	0.01919
62.319	0.02442	62.220	0.01902
62.319	0.0244	62.221	0.019
62.320	0.02438	62.221	0.01921
62.320	0.02436	62.222	0.01924
62.320	0.02434	62.222	0.01912
62.321	0.02432	62.222	0.01908
62.321	0.0243	62.223	0.0189
62.322	0.02429	62.223	0.01907

62.322	0.02427	62.224	0.01901
62.322	0.02425	62.224	0.01884
62.323	0.02424	62.224	0.01882
62.323	0.02422	62.225	0.01898
62.323	0.0242	62.225	0.01878
62.324	0.02418	62.226	0.01876
62.324	0.02416	62.226	0.01874
62.324	0.02414	62.226	0.01872
62.325	0.02412	62.227	0.0187
62.325	0.0241	62.227	0.01868
62.325	0.02408	62.228	0.01866
62.326	0.02406	62.228	0.01865
62.326	0.02404	62.228	0.01863
62.326	0.02402	62.229	0.01861
62.327	0.024	62.229	0.01859
62.327	0.02398	62.230	0.01856
62.327	0.02396	62.230	0.01854
62.328	0.02395	62.230	0.01852
62.328	0.02393	62.231	0.0185
62.328	0.02391	62.231	0.01848
62.329	0.02389	62.232	0.01845
62.329	0.02387	62.232	0.01843
62.329	0.02385	62.232	0.01842
62.330	0.02383	62.233	0.01845
62.330	0.02381	62.233	0.01847
62.331	0.02379	62.234	0.01849
62.331	0.02377	62.234	0.01851
62.331	0.02375	62.234	0.01853
62.332	0.02373	62.235	0.01855
62.332	0.02372	62.235	0.01857
62.332	0.0237	62.236	0.01859
62.333	0.02368	62.236	0.0186
62.333	0.02366	62.236	0.01862
62.333	0.02364	62.237	0.01863
62.334	0.02362	62.237	0.01865
62.334	0.0236	62.238	0.01866
62.334	0.02357	62.238	0.01867
62.335	0.02355	62.238	0.01868
62.335	0.02354	62.239	0.01869
62.335	0.02352	62.239	0.0187
62.336	0.0235	62.240	0.01871
62.336	0.02349	62.240	0.01872
62.336	0.02347	62.240	0.01873
62.337	0.02345	62.241	0.01873
62.337	0.02344	62.241	0.01874
62.337	0.02342	62.242	0.01875
62.338	0.0234	62.242	0.01875
62.338	0.02337	62.243	0.01875
62.338	0.02335	62.243	0.01876
62.339	0.02333	62.243	0.01876
62.339	0.02332	62.244	0.01878
62.339	0.02332	62.244	0.01881
62.340	0.02332	62.245	0.01884

62.340	0.02332	62.245	0.01886
62.340	0.02332	62.245	0.01889
62.341	0.02332	62.246	0.01891
62.341	0.02332	62.246	0.01894
62.341	0.02331	62.247	0.01896
62.342	0.02331	62.247	0.01898
62.342	0.02331	62.247	0.01901
62.342	0.02331	62.248	0.01903
62.343	0.02331	62.248	0.01905
62.343	0.0233	62.249	0.01908
62.343	0.0233	62.249	0.0191
62.344	0.0233	62.250	0.01912
62.344	0.0233	62.250	0.01914
62.344	0.02329	62.250	0.01917
62.345	0.02329	62.251	0.01919
62.345	0.02328	62.251	0.01921
62.345	0.02328	62.252	0.01923
62.346	0.02327	62.252	0.01925
62.346	0.02327	62.252	0.01927
62.346	0.02326	62.253	0.01929
62.347	0.02325	62.253	0.01931
62.347	0.02325	62.254	0.01933
62.347	0.02324	62.254	0.01935
62.348	0.02324	62.255	0.01936
62.348	0.02323	62.255	0.01938
62.348	0.02322	62.255	0.0194
62.349	0.02322	62.256	0.01942
62.349	0.02321	62.256	0.01943
62.349	0.0232	62.257	0.01945
62.350	0.02319	62.257	0.01948
62.350	0.02318	62.258	0.01953
62.350	0.02317	62.258	0.01957
62.351	0.02317	62.258	0.01962
62.351	0.02316	62.259	0.01966
62.351	0.02315	62.259	0.01971
62.352	0.02313	62.260	0.01975
62.352	0.02312	62.260	0.0198
62.352	0.02311	62.261	0.01984
62.353	0.0231	62.261	0.01988
62.353	0.02309	62.261	0.01993
62.353	0.02308	62.262	0.01997
62.354	0.02306	62.262	0.02001
62.354	0.02305	62.263	0.02005
62.354	0.02304	62.263	0.02009
62.355	0.02302	62.264	0.02013
62.355	0.02301	62.264	0.02017
62.355	0.02299	62.264	0.02021
62.356	0.02298	62.265	0.02025
62.356	0.02296	62.265	0.02029
62.357	0.02294	62.266	0.02032
62.357	0.02293	62.266	0.02036
62.357	0.02291	62.267	0.02039
62.358	0.02289	62.267	0.02043

62.358	0.02287	62.267	0.02046
62.358	0.02286	62.268	0.0205
62.359	0.02284	62.268	0.02053
62.359	0.02282	62.269	0.02056
62.359	0.0228	62.269	0.02059
62.360	0.02278	62.270	0.02062
62.360	0.02276	62.270	0.02065
62.360	0.02273	62.271	0.02068
62.361	0.02271	62.271	0.02071
62.361	0.02269	62.271	0.02074
62.361	0.02267	62.272	0.02076
62.362	0.02264	62.272	0.02079
62.362	0.02262	62.273	0.02081
62.362	0.0226	62.273	0.02084
62.363	0.02258	62.274	0.02086
62.363	0.02256	62.274	0.02088
62.363	0.02254	62.275	0.0209
62.364	0.02251	62.275	0.02092
62.364	0.02249	62.275	0.02094
62.364	0.02247	62.276	0.02096
62.365	0.02244	62.276	0.02098
62.365	0.02242	62.277	0.021
62.365	0.02239	62.277	0.02101
62.366	0.02237	62.278	0.02103
62.366	0.02234	62.278	0.02104
62.366	0.02232	62.278	0.02105
62.367	0.02229	62.279	0.02106
62.367	0.02227	62.279	0.02107
62.367	0.02224	62.280	0.02108
62.368	0.02221	62.280	0.02109
62.368	0.02219	62.281	0.0211
62.368	0.02216	62.281	0.02111
62.369	0.02213	62.282	0.02111
62.369	0.0221	62.282	0.02112
62.370	0.02208	62.282	0.02112
62.370	0.02205	62.283	0.02113
62.370	0.02202	62.283	0.02113
62.371	0.02199	62.284	0.02113
62.371	0.02196	62.284	0.02113
62.371	0.02193	62.285	0.02113
62.372	0.0219	62.285	0.02113
62.372	0.02187	62.286	0.02112
62.372	0.02184	62.286	0.02112
62.373	0.02181	62.287	0.02112
62.373	0.02179	62.287	0.02111
62.373	0.02179	62.287	0.02111
62.374	0.02178	62.288	0.0211
62.374	0.02177	62.288	0.02109
62.374	0.02177	62.289	0.02108
62.375	0.02176	62.289	0.02107
62.375	0.02175	62.290	0.02106
62.375	0.02174	62.290	0.02105
62.376	0.02174	62.291	0.02104

62.376	0.02173	62.291	0.02102
62.376	0.02172	62.291	0.02101
62.377	0.02171	62.292	0.02099
62.377	0.0217	62.292	0.02098
62.377	0.02169	62.293	0.02096
62.378	0.02168	62.293	0.02094
62.378	0.02167	62.294	0.02093
62.379	0.02166	62.294	0.02091
62.379	0.02166	62.295	0.02089
62.379	0.02167	62.295	0.02087
62.380	0.02168	62.296	0.02085
62.380	0.02168	62.296	0.02082
62.380	0.02169	62.296	0.0208
62.381	0.0217	62.297	0.02078
62.381	0.0217	62.297	0.02075
62.381	0.02171	62.298	0.02073
62.382	0.02172	62.298	0.02071
62.382	0.02172	62.299	0.02068
62.382	0.02172	62.299	0.02065
62.383	0.02173	62.300	0.02063
62.383	0.02173	62.300	0.0206
62.383	0.02175	62.301	0.02057
62.384	0.02176	62.301	0.02054
62.384	0.02177	62.301	0.02051
62.385	0.02178	62.302	0.02048
62.385	0.02179	62.302	0.02045
62.385	0.0218	62.303	0.02042
62.386	0.02181	62.303	0.02039
62.386	0.02181	62.304	0.02036
62.386	0.02181	62.304	0.02033
62.387	0.02182	62.305	0.0203
62.387	0.02182	62.305	0.02027
62.387	0.02182	62.306	0.02024
62.388	0.02182	62.306	0.0202
62.388	0.02182	62.307	0.02017
62.388	0.02181	62.307	0.02016
62.389	0.02181	62.307	0.02016
62.389	0.02181	62.308	0.02017
62.390	0.0218	62.308	0.02017
62.390	0.02179	62.309	0.02017
62.390	0.02178	62.309	0.02018
62.391	0.02177	62.310	0.02018
62.391	0.02176	62.310	0.02018
62.391	0.02175	62.311	0.02018
62.392	0.02174	62.311	0.02018
62.392	0.02173	62.312	0.02018
62.392	0.02171	62.312	0.02018
62.393	0.0217	62.313	0.02018
62.393	0.02168	62.313	0.02018
62.394	0.02166	62.313	0.02018
62.394	0.02165	62.314	0.02017
62.394	0.02163	62.314	0.02017
62.395	0.02161	62.315	0.02017

62.395	0.02159	62.315	0.02016
62.395	0.02157	62.316	0.02016
62.396	0.02155	62.316	0.02016
62.396	0.02153	62.317	0.02017
62.396	0.0215	62.317	0.02017
62.397	0.02148	62.318	0.02018
62.397	0.02146	62.318	0.02018
62.398	0.02143	62.319	0.02019
62.398	0.02141	62.319	0.02019
62.398	0.02139	62.319	0.02019
62.399	0.02136	62.320	0.0202
62.399	0.02134	62.320	0.0202
62.399	0.02131	62.321	0.0202
62.400	0.02128	62.321	0.0202
62.400	0.02126	62.322	0.02021
62.400	0.02123	62.322	0.02021
62.401	0.0212	62.323	0.02021
62.401	0.02118	62.323	0.02021
62.402	0.02115	62.324	0.02021
62.402	0.02112	62.324	0.02021
62.402	0.0211	62.325	0.02021
62.403	0.02107	62.325	0.02021
62.403	0.02104	62.326	0.0202
62.403	0.02102	62.326	0.0202
62.404	0.02101	62.326	0.0202
62.404	0.021	62.327	0.02019
62.405	0.02098	62.327	0.02019
62.405	0.02097	62.328	0.02018
62.405	0.02096	62.328	0.02018
62.406	0.02094	62.329	0.02017
62.406	0.02092	62.329	0.02016
62.407	0.02091	62.330	0.02015
62.407	0.02089	62.330	0.02014
62.407	0.02087	62.331	0.02013
62.408	0.02085	62.331	0.02012
62.408	0.02083	62.332	0.02011
62.408	0.02081	62.332	0.0201
62.409	0.02078	62.333	0.02008
62.409	0.02076	62.333	0.02007
62.410	0.02074	62.333	0.02005
62.410	0.02071	62.334	0.02004
62.410	0.02069	62.334	0.02002
62.411	0.02066	62.335	0.02
62.411	0.02064	62.335	0.01998
62.411	0.02061	62.336	0.01996
62.412	0.02058	62.336	0.01994
62.412	0.02055	62.337	0.01992
62.413	0.02053	62.337	0.01989
62.413	0.0205	62.338	0.01987
62.413	0.02047	62.338	0.01985
62.414	0.02045	62.339	0.01982
62.414	0.02042	62.339	0.01979
62.415	0.02039	62.340	0.01976

62.415	0.02036	62.340	0.01973
62.415	0.02035	62.340	0.0197
62.416	0.02038	62.341	0.01967
62.416	0.0204	62.341	0.01964
62.417	0.02042	62.342	0.01961
62.417	0.02044	62.342	0.01957
62.417	0.02047	62.343	0.01954
62.418	0.02049	62.343	0.0195
62.418	0.02051	62.344	0.01946
62.419	0.02053	62.344	0.01943
62.419	0.02056	62.345	0.01939
62.419	0.02058	62.345	0.01935
62.420	0.0206	62.346	0.01931
62.420	0.02061	62.346	0.01926
62.420	0.02063	62.347	0.01922
62.421	0.02065	62.347	0.01918
62.421	0.02069	62.347	0.01913
62.422	0.02073	62.348	0.01909
62.422	0.02077	62.348	0.01904
62.422	0.02081	62.349	0.01899
62.423	0.02085	62.349	0.01894
62.423	0.02088	62.350	0.0189
62.424	0.02092	62.350	0.01885
62.424	0.02095	62.351	0.0188
62.424	0.02098	62.351	0.01875
62.425	0.02101	62.352	0.0187
62.425	0.02104	62.352	0.01865
62.426	0.02107	62.353	0.01859
62.426	0.02109	62.353	0.01854
62.426	0.02111	62.353	0.01849
62.427	0.02113	62.354	0.01844
62.427	0.02115	62.354	0.01838
62.428	0.02117	62.355	0.01833
62.428	0.02118	62.355	0.01827
62.428	0.02119	62.356	0.01822
62.429	0.0212	62.356	0.01817
62.429	0.02121	62.357	0.01811
62.430	0.02122	62.357	0.01806
62.430	0.02122	62.358	0.01803
62.430	0.02122	62.358	0.01801
62.431	0.02122	62.359	0.018
62.431	0.02122	62.359	0.01798
62.432	0.02121	62.359	0.01797
62.432	0.0212	62.360	0.01795
62.432	0.02119	62.360	0.01794
62.433	0.02118	62.361	0.01792
62.433	0.02119	62.361	0.01791
62.434	0.02122	62.362	0.01789
62.434	0.02124	62.362	0.01787
62.434	0.02127	62.363	0.01786
62.435	0.02129	62.363	0.01784
62.435	0.02132	62.364	0.01782
62.436	0.02134	62.364	0.01781

62.436	0.02136	62.364	0.01779
62.436	0.02138	62.365	0.01777
62.437	0.0214	62.365	0.01775
62.437	0.02142	62.366	0.01774
62.438	0.02144	62.366	0.01772
62.438	0.02145	62.367	0.0177
62.438	0.02147	62.367	0.01768
62.439	0.02148	62.368	0.01766
62.439	0.0215	62.368	0.01764
62.440	0.02151	62.369	0.01762
62.440	0.02152	62.369	0.0176
62.440	0.02154	62.369	0.01759
62.441	0.02155	62.370	0.01757
62.441	0.02156	62.370	0.01755
62.442	0.02157	62.371	0.01753
62.442	0.02158	62.371	0.01751
62.442	0.02159	62.372	0.01748
62.443	0.0216	62.372	0.01746
62.443	0.02161	62.373	0.01744
62.444	0.02162	62.373	0.01742
62.444	0.02172	62.374	0.01741
62.444	0.02163	62.374	0.01739
62.445	0.02164	62.374	0.01738
62.445	0.02165	62.375	0.01736
62.446	0.02166	62.375	0.01735
62.446	0.0217	62.376	0.01733
62.446	0.02183	62.376	0.01732
62.447	0.02177	62.377	0.0173
62.447	0.02181	62.377	0.01729
62.448	0.02184	62.378	0.01727
62.448	0.02188	62.378	0.01725
62.448	0.02191	62.379	0.01723
62.449	0.02194	62.379	0.01721
62.449	0.02207	62.380	0.01719
62.450	0.02201	62.380	0.01718
62.450	0.02203	62.380	0.01716
62.450	0.02206	62.381	0.01713
62.451	0.02209	62.381	0.01711
62.451	0.02211	62.382	0.01709
62.452	0.02213	62.382	0.01707
62.452	0.02215	62.383	0.01705
62.452	0.02217	62.383	0.01702
62.453	0.02219	62.384	0.017
62.453	0.02221	62.384	0.01697
62.454	0.02222	62.385	0.01695
62.454	0.02223	62.385	0.01692
62.454	0.02224	62.386	0.0169
62.455	0.02225	62.386	0.01687
62.455	0.02226	62.386	0.01684
62.456	0.02264	62.387	0.01681
62.456	0.02239	62.387	0.01678
62.456	0.02227	62.388	0.01675
62.457	0.02228	62.388	0.01672

62.457	0.02228	62.389	0.01669
62.458	0.02228	62.389	0.01666
62.458	0.02228	62.390	0.01663
62.459	0.02227	62.390	0.0166
62.459	0.0225	62.391	0.01656
62.459	0.02228	62.391	0.01653
62.460	0.02227	62.392	0.01649
62.460	0.02228	62.392	0.01646
62.461	0.0223	62.393	0.01642
62.461	0.02233	62.393	0.01639
62.461	0.02235	62.393	0.01635
62.462	0.02237	62.394	0.01632
62.462	0.02239	62.394	0.01628
62.463	0.02244	62.395	0.01624
62.463	0.02243	62.395	0.0162
62.463	0.02245	62.396	0.01616
62.464	0.02247	62.396	0.01612
62.464	0.02249	62.397	0.01608
62.465	0.0225	62.397	0.01604
62.465	0.02252	62.398	0.016
62.465	0.02254	62.398	0.01596
62.466	0.02255	62.399	0.01592
62.466	0.02256	62.399	0.01588
62.467	0.02258	62.400	0.01584
62.467	0.02259	62.400	0.0158
62.468	0.0226	62.401	0.01576
62.468	0.02261	62.401	0.01571
62.468	0.02262	62.402	0.01568
62.469	0.02262	62.402	0.01565
62.469	0.02263	62.403	0.01562
62.470	0.02263	62.403	0.01558
62.470	0.02264	62.403	0.01555
62.470	0.02264	62.404	0.01552
62.471	0.02264	62.404	0.01549
62.471	0.02264	62.405	0.01545
62.472	0.02264	62.405	0.01542
62.472	0.02263	62.406	0.0154
62.472	0.02263	62.406	0.01538
62.473	0.02262	62.407	0.01536
62.473	0.02261	62.407	0.01534
62.474	0.0226	62.408	0.01532
62.474	0.02258	62.408	0.0153
62.475	0.02257	62.409	0.01528
62.475	0.02255	62.409	0.01526
62.475	0.02254	62.410	0.01524
62.476	0.02252	62.410	0.01521
62.476	0.02249	62.411	0.01519
62.477	0.02247	62.411	0.01517
62.477	0.02245	62.412	0.01515
62.477	0.02242	62.412	0.01513
62.478	0.02239	62.413	0.01511
62.478	0.02236	62.413	0.01508
62.479	0.02232	62.414	0.01506

62.479	0.02229	62.414	0.01504
62.479	0.02225	62.415	0.01501
62.480	0.02221	62.415	0.01499
62.480	0.02217	62.416	0.01497
62.481	0.02213	62.416	0.01494
62.481	0.02208	62.417	0.01492
62.482	0.02204	62.417	0.01489
62.482	0.02199	62.418	0.01487
62.482	0.02194	62.418	0.01484
62.483	0.02189	62.419	0.01482
62.483	0.02183	62.419	0.01479
62.484	0.02178	62.420	0.01476
62.484	0.02172	62.420	0.01473
62.484	0.02166	62.421	0.01471
62.485	0.0216	62.421	0.01468
62.485	0.02154	62.422	0.01467
62.486	0.02148	62.422	0.01466
62.486	0.02141	62.423	0.01464
62.487	0.02135	62.423	0.01463
62.487	0.02128	62.424	0.01461
62.487	0.02121	62.424	0.01459
62.488	0.02114	62.425	0.01458
62.488	0.02107	62.425	0.01456
62.489	0.021	62.426	0.01454
62.489	0.02093	62.426	0.01453
62.489	0.02086	62.427	0.01451
62.490	0.02078	62.427	0.01449
62.490	0.02071	62.428	0.01447
62.491	0.0208	62.428	0.01445
62.491	0.02064	62.429	0.01443
62.492	0.02048	62.429	0.01442
62.492	0.0204	62.430	0.01439
62.492	0.02042	62.430	0.01437
62.493	0.02024	62.431	0.01435
62.493	0.02049	62.432	0.01433
62.494	0.0202	62.432	0.01431
62.494	0.0201	62.433	0.01429
62.495	0.02036	62.433	0.01426
62.495	0.02005	62.434	0.01424
62.495	0.02005	62.434	0.01421
62.496	0.02019	62.435	0.01419
62.496	0.02013	62.435	0.01416
62.497	0.02004	62.436	0.01414
62.497	0.01997	62.436	0.01411
62.498	0.02001	62.437	0.01408
62.498	0.01985	62.437	0.01406
62.498	0.01992	62.438	0.01403
62.499	0.01991	62.438	0.01401
62.499	0.01996	62.439	0.01398
62.500	0.01963	62.439	0.01396
62.500	0.01935	62.440	0.01393
62.501	0.01982	62.441	0.0139
62.501	0.01953	62.441	0.01387

62.501	0.01976	62.442	0.01384
62.502	0.01948	62.442	0.01381
62.502	0.01927	62.443	0.01378
62.503	0.01977	62.443	0.01375
62.503	0.01974	62.444	0.01371
62.504	0.01943	62.444	0.01368
62.504	0.01925	62.445	0.01365
62.504	0.01917	62.445	0.01361
62.505	0.01915	62.446	0.01357
62.505	0.01959	62.446	0.01354
62.506	0.0196	62.447	0.0135
62.506	0.01945	62.447	0.01346
62.507	0.01941	62.448	0.01342
62.507	0.0194	62.448	0.01338
62.507	0.01921	62.449	0.01334
62.508	0.01925	62.450	0.01329
62.508	0.01938	62.450	0.01325
62.509	0.01907	62.451	0.01321
62.509	0.01887	62.451	0.01316
62.510	0.01884	62.452	0.01312
62.510	0.01926	62.452	0.01307
62.510	0.01897	62.453	0.01303
62.511	0.01913	62.453	0.01298
62.511	0.01906	62.454	0.01293
62.512	0.01922	62.454	0.01289
62.512	0.0189	62.455	0.01284
62.513	0.01859	62.455	0.01279
62.513	0.01903	62.456	0.01274
62.513	0.01909	62.456	0.01269
62.514	0.01876	62.457	0.01264
62.514	0.01875	62.458	0.01259
62.515	0.01859	62.458	0.01258
62.515	0.01859	62.459	0.01256
62.516	0.01886	62.459	0.01255
62.516	0.01858	62.460	0.01253
62.517	0.01858	62.460	0.01252
62.517	0.0188	62.461	0.0125
62.517	0.01857	62.461	0.01248
62.518	0.01857	62.462	0.01247
62.518	0.01856	62.463	0.01245
62.519	0.01856	62.463	0.01243
62.519	0.01855	62.464	0.01242
62.520	0.01854	62.464	0.0124
62.520	0.01854	62.465	0.01238
62.520	0.01853	62.465	0.01236
62.521	0.01852	62.466	0.01234
62.521	0.01851	62.466	0.01232
62.522	0.01849	62.467	0.0123
62.522	0.01848	62.468	0.01228
62.523	0.01846	62.468	0.01226
62.523	0.01845	62.469	0.01224
62.524	0.01843	62.469	0.01222
62.524	0.01841	62.470	0.0122

62.524	0.01839	62.470	0.01218
62.525	0.01837	62.471	0.01216
62.525	0.01835	62.472	0.01214
62.526	0.01832	62.472	0.01211
62.526	0.0183	62.473	0.01209
62.527	0.01827	62.473	0.01207
62.527	0.01824	62.474	0.01204
62.528	0.01821	62.474	0.01202
62.528	0.01818	62.475	0.012
62.528	0.01814	62.475	0.01197
62.529	0.01811	62.476	0.01195
62.529	0.01807	62.477	0.01192
62.530	0.01803	62.477	0.01189
62.530	0.01799	62.478	0.01187
62.531	0.01795	62.478	0.01184
62.531	0.01792	62.479	0.01181
62.532	0.01788	62.479	0.01178
62.532	0.01785	62.480	0.01175
62.532	0.01781	62.480	0.01172
62.533	0.01777	62.481	0.01169
62.533	0.01773	62.482	0.01166
62.534	0.01768	62.482	0.01163
62.534	0.01764	62.483	0.0116
62.535	0.01759	62.483	0.01157
62.535	0.01755	62.484	0.01153
62.536	0.0175	62.484	0.0115
62.536	0.01745	62.485	0.01146
62.536	0.0174	62.485	0.01143
62.537	0.01735	62.486	0.01139
62.537	0.0173	62.487	0.01135
62.538	0.01725	62.487	0.01131
62.538	0.01719	62.488	0.01127
62.539	0.01714	62.488	0.01123
62.539	0.01709	62.489	0.01119
62.540	0.01703	62.489	0.01115
62.540	0.01697	62.490	0.01111
62.540	0.01692	62.490	0.01106
62.541	0.01686	62.491	0.01102
62.541	0.01681	62.492	0.01097
62.542	0.01675	62.492	0.01092
62.542	0.01669	62.493	0.01088
62.543	0.01663	62.493	0.01083
62.543	0.01658	62.494	0.01078
62.544	0.01653	62.494	0.01073
62.544	0.01649	62.495	0.01067
62.545	0.01644	62.496	0.01062
62.545	0.01641	62.496	0.01057
62.545	0.01639	62.497	0.01051
62.546	0.01636	62.497	0.01046
62.546	0.01634	62.498	0.0104
62.547	0.01631	62.498	0.01035
62.547	0.01629	62.499	0.01029
62.548	0.01626	62.500	0.01023

62.548	0.01623	62.500	0.01017
62.549	0.01621	62.501	0.01012
62.549	0.01618	62.501	0.01006
62.550	0.01615	62.502	0.01
62.550	0.01612	62.502	0.00994
62.550	0.0161	62.503	0.00988
62.551	0.01607	62.504	0.00981
62.551	0.01604	62.504	0.00975
62.552	0.01601	62.505	0.00969
62.552	0.01598	62.505	0.00963
62.553	0.01595	62.506	0.00961
62.553	0.01592	62.506	0.00959
62.554	0.0159	62.507	0.00957
62.554	0.01587	62.507	0.00955
62.554	0.01585	62.508	0.00953
62.555	0.01582	62.509	0.00951
62.555	0.01579	62.509	0.00949
62.556	0.01577	62.510	0.00947
62.556	0.01574	62.510	0.00946
62.557	0.01571	62.511	0.00944
62.557	0.01568	62.511	0.00942
62.558	0.01565	62.512	0.0094
62.558	0.01563	62.512	0.00938
62.559	0.0156	62.513	0.00936
62.559	0.01558	62.514	0.00934
62.559	0.01557	62.514	0.00932
62.560	0.01556	62.515	0.00931
62.560	0.01554	62.515	0.00929
62.561	0.01553	62.516	0.00927
62.561	0.01551	62.516	0.00925
62.562	0.0155	62.517	0.00923
62.562	0.01548	62.518	0.00921
62.563	0.01546	62.518	0.00919
62.563	0.01545	62.519	0.00918
62.564	0.01543	62.519	0.00916
62.564	0.01541	62.520	0.00914
62.565	0.01539	62.520	0.00912
62.565	0.01537	62.521	0.0091
62.565	0.01535	62.522	0.00908
62.566	0.01532	62.522	0.00907
62.566	0.0153	62.523	0.00905
62.567	0.01528	62.523	0.00903
62.567	0.01525	62.524	0.00901
62.568	0.01523	62.524	0.00899
62.568	0.0152	62.525	0.00897
62.569	0.01517	62.526	0.00895
62.569	0.01515	62.526	0.00893
62.570	0.01512	62.527	0.00891
62.570	0.01509	62.527	0.00889
62.570	0.01506	62.528	0.00887
62.571	0.01503	62.528	0.00885
62.571	0.015	62.529	0.00883
62.572	0.01496	62.530	0.00881

62.572	0.01493	62.530	0.00879
62.573	0.0149	62.531	0.00877
62.573	0.01486	62.531	0.00875
62.574	0.01483	62.532	0.00873
62.574	0.01479	62.532	0.00871
62.575	0.01475	62.533	0.00869
62.575	0.01472	62.534	0.00867
62.575	0.01468	62.534	0.00865
62.576	0.01464	62.535	0.00863
62.576	0.0146	62.535	0.00861
62.577	0.01458	62.536	0.00859
62.577	0.01458	62.536	0.00857
62.578	0.01458	62.537	0.00855
62.578	0.01458	62.538	0.00854
62.579	0.01458	62.538	0.00853
62.579	0.01458	62.539	0.00852
62.580	0.01458	62.539	0.00851
62.580	0.01457	62.540	0.0085
62.580	0.01456	62.540	0.00849
62.581	0.01456	62.541	0.00848
62.581	0.01455	62.541	0.00847
62.582	0.01454	62.542	0.00846
62.582	0.01453	62.543	0.00845
62.583	0.01451	62.543	0.00844
62.583	0.0145	62.544	0.00842
62.584	0.01448	62.544	0.00841
62.584	0.01446	62.545	0.0084
62.585	0.01444	62.546	0.00839
62.585	0.01442	62.546	0.00838
62.585	0.0144	62.547	0.00837
62.586	0.01437	62.547	0.00836
62.586	0.01435	62.548	0.00835
62.587	0.01432	62.548	0.00834
62.587	0.01429	62.549	0.00833
62.588	0.01426	62.550	0.00832
62.588	0.01422	62.550	0.00831
62.589	0.01419	62.551	0.00836
62.589	0.01415	62.551	0.00835
62.590	0.01411	62.552	0.00827
62.590	0.01407	62.552	0.00826
62.590	0.01403	62.553	0.00832
62.591	0.01399	62.554	0.00847
62.591	0.01395	62.554	0.00842
62.592	0.0139	62.555	0.00837
62.592	0.01385	62.555	0.00851
62.593	0.0138	62.556	0.00853
62.593	0.01375	62.557	0.00849
62.594	0.0137	62.557	0.00853
62.594	0.01365	62.558	0.00849
62.594	0.0136	62.558	0.00843
62.595	0.01354	62.559	0.00852
62.595	0.01349	62.559	0.00851
62.596	0.01343	62.560	0.00844

62.596	0.01341	62.561	0.00856
62.597	0.01341	62.561	0.00845
62.597	0.01341	62.562	0.00871
62.598	0.01341	62.562	0.00882
62.598	0.01341	62.563	0.00865
62.599	0.01341	62.564	0.00863
62.599	0.01341	62.564	0.00857
62.599	0.01341	62.565	0.00877
62.600	0.0134	62.565	0.00865
62.600	0.0134	62.566	0.00873
62.601	0.0134	62.567	0.00867
62.601	0.0134	62.567	0.00883
62.602	0.0134	62.568	0.00884
62.602	0.01339	62.568	0.00895
62.603	0.01339	62.569	0.00887
62.603	0.01339	62.570	0.00883
62.603	0.01339	62.570	0.00911
62.604	0.01338	62.571	0.00887
62.604	0.01338	62.571	0.00881
62.605	0.01337	62.572	0.00888
62.605	0.01337	62.573	0.00907
62.606	0.01337	62.573	0.00887
62.606	0.01337	62.574	0.00885
62.607	0.01338	62.574	0.00885
62.607	0.01338	62.575	0.00894
62.608	0.01338	62.576	0.00916
62.608	0.01338	62.576	0.00902
62.608	0.01338	62.577	0.0092
62.609	0.01338	62.578	0.00906
62.609	0.01338	62.578	0.00902
62.610	0.01338	62.579	0.00956
62.610	0.01338	62.579	0.00906
62.611	0.01338	62.580	0.00924
62.611	0.01337	62.581	0.00915
62.612	0.01337	62.581	0.00922
62.612	0.01337	62.582	0.00919
62.612	0.01337	62.582	0.00923
62.613	0.01336	62.583	0.00939
62.613	0.01336	62.584	0.00935
62.614	0.01336	62.584	0.00935
62.614	0.01335	62.585	0.00941
62.615	0.01335	62.586	0.00946
62.615	0.01335	62.586	0.00946
62.616	0.01334	62.587	0.0095
62.616	0.01334	62.588	0.00954
62.617	0.01333	62.588	0.00957
62.617	0.01333	62.589	0.00961
62.617	0.01332	62.589	0.00965
62.618	0.01332	62.590	0.00968
62.618	0.01331	62.591	0.00972
62.619	0.0133	62.591	0.00975
62.619	0.0133	62.592	0.00978
62.620	0.01329	62.593	0.00982

62.620	0.01328	62.593	0.00985
62.621	0.01328	62.594	0.00988
62.621	0.01327	62.595	0.00991
62.621	0.01326	62.595	0.00994
62.622	0.01325	62.596	0.00996
62.622	0.01325	62.596	0.00999
62.623	0.01324	62.597	0.01002
62.623	0.01323	62.598	0.01004
62.624	0.01322	62.598	0.01007
62.624	0.01321	62.599	0.01009
62.625	0.0132	62.600	0.01011
62.625	0.01319	62.600	0.01013
62.626	0.01318	62.601	0.01015
62.626	0.01317	62.602	0.01017
62.626	0.01316	62.602	0.01018
62.627	0.01315	62.603	0.0102
62.627	0.01314	62.604	0.01021
62.628	0.01313	62.604	0.01023
62.628	0.01312	62.605	0.01024
62.629	0.01311	62.606	0.01025
62.629	0.01309	62.606	0.01026
62.630	0.01308	62.607	0.01027
62.630	0.01307	62.608	0.01028
62.631	0.01306	62.608	0.01035
62.631	0.01304	62.609	0.01029
62.631	0.01303	62.610	0.01029
62.632	0.01302	62.610	0.01029
62.632	0.013	62.611	0.01029
62.633	0.01299	62.612	0.01029
62.633	0.01298	62.612	0.01029
62.634	0.01296	62.613	0.01028
62.634	0.01295	62.614	0.01028
62.635	0.01293	62.614	0.01027
62.635	0.01292	62.615	0.01026
62.636	0.0129	62.616	0.01026
62.636	0.01289	62.616	0.01032
62.637	0.01287	62.617	0.01023
62.637	0.01286	62.618	0.01022
62.637	0.01284	62.618	0.01021
62.638	0.01283	62.619	0.01019
62.638	0.01281	62.620	0.01018
62.639	0.01279	62.620	0.01016
62.639	0.01278	62.621	0.01015
62.640	0.01276	62.622	0.01013
62.640	0.01274	62.622	0.01011
62.641	0.01273	62.623	0.01009
62.641	0.01271	62.624	0.01007
62.642	0.01269	62.625	0.01005
62.642	0.01267	62.625	0.01003
62.643	0.01266	62.626	0.01
62.643	0.01264	62.627	0.00998
62.644	0.01262	62.627	0.00995
62.644	0.0126	62.628	0.00993

62.644	0.01258	62.629	0.00999
62.645	0.01256	62.629	0.0099
62.645	0.01254	62.630	0.00989
62.646	0.01252	62.631	0.00989
62.646	0.0125	62.632	0.00988
62.647	0.01248	62.632	0.00987
62.647	0.01246	62.633	0.00988
62.648	0.01244	62.634	0.00988
62.648	0.01242	62.634	0.00984
62.649	0.0124	62.635	0.00985
62.649	0.01238	62.636	0.00982
62.650	0.01236	62.637	0.00995
62.650	0.01234	62.637	0.00979
62.651	0.01232	62.638	0.00978
62.651	0.0123	62.639	0.00976
62.652	0.01228	62.639	0.00975
62.652	0.01226	62.640	0.00973
62.653	0.01224	62.641	0.00972
62.653	0.01222	62.642	0.00974
62.653	0.01219	62.642	0.00969
62.654	0.01217	62.643	0.00969
62.654	0.01215	62.644	0.00973
62.655	0.01213	62.644	0.00971
62.655	0.0121	62.645	0.00961
62.656	0.01208	62.646	0.00963
62.656	0.01206	62.647	0.00977
62.657	0.01204	62.647	0.00957
62.657	0.01201	62.648	0.00953
62.658	0.01199	62.649	0.0097
62.658	0.01197	62.650	0.00963
62.659	0.01195	62.650	0.0095
62.659	0.01192	62.651	0.00947
62.660	0.0119	62.652	0.00942
62.660	0.01188	62.653	0.00952
62.661	0.01185	62.653	0.00937
62.661	0.01183	62.654	0.00943
62.662	0.01181	62.655	0.00948
62.662	0.01178	62.656	0.00947
62.663	0.01176	62.656	0.00936
62.663	0.01174	62.657	0.00929
62.664	0.01171	62.658	0.00919
62.664	0.01169	62.659	0.00916
62.665	0.01166	62.660	0.00926
62.665	0.01164	62.660	0.0092
62.666	0.01162	62.661	0.00915
62.666	0.01159	62.662	0.00919
62.667	0.01157	62.663	0.00912
62.667	0.01155	62.663	0.00917
62.668	0.01152	62.664	0.00907
62.668	0.0115	62.665	0.00907
62.669	0.01148	62.666	0.0089
62.669	0.01145	62.667	0.00894
62.670	0.01143	62.667	0.00882

62.670	0.01141	62.668	0.00893
62.671	0.01138	62.669	0.00887
62.671	0.01136	62.670	0.00876
62.672	0.01134	62.670	0.00868
62.672	0.01132	62.671	0.00866
62.673	0.01129	62.672	0.0087
62.673	0.01127	62.673	0.00863
62.674	0.01125	62.674	0.00859
62.674	0.01123	62.675	0.00859
62.675	0.01121	62.675	0.00845
62.675	0.01118	62.676	0.00847
62.676	0.01116	62.677	0.00833
62.676	0.01114	62.678	0.00835
62.677	0.01112	62.679	0.00834
62.677	0.0111	62.679	0.00821
62.678	0.01107	62.680	0.00818
62.678	0.01105	62.681	0.00825
62.679	0.01103	62.682	0.00813
62.679	0.01101	62.683	0.00816
62.680	0.01099	62.684	0.00808
62.680	0.01097	62.684	0.00815
62.681	0.01095	62.685	0.00806
62.681	0.01093	62.686	0.00798
62.682	0.01091	62.687	0.00798
62.682	0.0109	62.688	0.00796
62.683	0.01089	62.689	0.00786
62.683	0.01088	62.689	0.00785
62.684	0.01087	62.690	0.00791
62.684	0.01086	62.691	0.00777
62.685	0.01085	62.692	0.00776
62.685	0.01084	62.693	0.00771
62.686	0.01083	62.694	0.00768
62.686	0.01081	62.695	0.00767
62.687	0.0108	62.695	0.00763
62.688	0.01079	62.696	0.00758
62.688	0.01077	62.697	0.00756
62.689	0.01076	62.698	0.00752
62.689	0.01075	62.699	0.00748
62.690	0.01073	62.700	0.00744
62.690	0.01072	62.701	0.00746
62.691	0.0107	62.701	0.00737
62.691	0.01068	62.702	0.00742
62.692	0.01067	62.703	0.00733
62.692	0.01065	62.704	0.00725
62.693	0.01063	62.705	0.00721
62.693	0.01062	62.706	0.00724
62.694	0.01061	62.707	0.00724
62.694	0.0106	62.708	0.00709
62.695	0.01059	62.709	0.00713
62.695	0.01058	62.709	0.00712
62.696	0.01057	62.710	0.00694
62.696	0.01055	62.711	0.00693
62.697	0.01054	62.712	0.007

62.698	0.01053	62.713	0.00684
62.698	0.01052	62.714	0.00683
62.699	0.01051	62.715	0.0069
62.699	0.01049	62.716	0.00676
62.700	0.01048	62.717	0.00671
62.700	0.01047	62.718	0.00666
62.701	0.01045	62.719	0.00679
62.701	0.01044	62.720	0.00665
62.702	0.01042	62.720	0.00656
62.702	0.01041	62.721	0.00667
62.703	0.01039	62.722	0.00653
62.703	0.01037	62.723	0.00648
62.704	0.01036	62.724	0.00647
62.704	0.01034	62.725	0.00656
62.705	0.01033	62.726	0.00641
62.706	0.01033	62.727	0.00632
62.706	0.01032	62.728	0.00629
62.707	0.01031	62.729	0.00643
62.707	0.0103	62.730	0.00628
62.708	0.0103	62.731	0.00621
62.708	0.01029	62.732	0.0062
62.709	0.01028	62.733	0.0063
62.709	0.01027	62.734	0.00612
62.710	0.01026	62.734	0.00605
62.710	0.01025	62.735	0.00603
62.711	0.01024	62.736	0.00602
62.711	0.01023	62.737	0.00613
62.712	0.01022	62.738	0.00592
62.713	0.01021	62.739	0.00591
62.713	0.01021	62.740	0.00586
62.714	0.01021	62.741	0.00582
62.714	0.01022	62.742	0.0058
62.715	0.01022	62.743	0.00579
62.715	0.01022	62.744	0.00589
62.716	0.01022	62.745	0.00572
62.716	0.01022	62.746	0.00569
62.717	0.01022	62.747	0.00564
62.717	0.01022	62.748	0.00564
62.718	0.01022	62.749	0.00556
62.718	0.01022	62.750	0.00552
62.719	0.01022	62.751	0.00553
62.720	0.01022	62.752	0.00548
62.720	0.01022	62.752	0.00543
62.721	0.01022	62.753	0.00539
62.721	0.01023	62.754	0.00533
62.722	0.01024	62.755	0.00532
62.722	0.01025	62.756	0.00526
62.723	0.01026	62.757	0.00525
62.723	0.01027	62.758	0.00519
62.724	0.01027	62.759	0.00519
62.724	0.01028	62.760	0.00515
62.725	0.01029	62.761	0.0051
62.726	0.01029	62.762	0.00506

62.726	0.01029	62.763	0.00503
62.727	0.0103	62.764	0.00504
62.727	0.0103	62.765	0.00494
62.728	0.0103	62.765	0.00494
62.728	0.0103	62.766	0.00489
62.729	0.0103	62.767	0.0048
62.729	0.0103	62.768	0.00485
62.730	0.01029	62.769	0.00473
62.731	0.01029	62.770	0.00473
62.731	0.01028	62.771	0.0047
62.732	0.01028	62.772	0.00465
62.732	0.01027	62.773	0.0046
62.733	0.01026	62.774	0.00456
62.733	0.01025	62.775	0.00448
62.734	0.01024	62.775	0.00452
62.734	0.01023	62.776	0.0045
62.735	0.01022	62.777	0.00446
62.736	0.0102	62.778	0.00437
62.736	0.01019	62.779	0.00433
62.737	0.01017	62.780	0.00435
62.737	0.01015	62.781	0.00439
62.738	0.01013	62.782	0.00431
62.738	0.01012	62.783	0.00428
62.739	0.0101	62.784	0.00419
62.739	0.01007	62.784	0.00417
62.740	0.01005	62.785	0.00418
62.741	0.01003	62.786	0.00412
62.741	0.01001	62.787	0.0041
62.742	0.00998	62.788	0.00408
62.742	0.00996	62.789	0.00406
62.743	0.00994	62.790	0.00403
62.743	0.00992	62.791	0.00401
62.744	0.00989	62.791	0.00398
62.744	0.00987	62.792	0.00396
62.745	0.00985	62.793	0.00394
62.746	0.00983	62.794	0.00391
62.746	0.0098	62.795	0.00388
62.747	0.00978	62.796	0.00386
62.747	0.00975	62.797	0.00384
62.748	0.00984	62.798	0.00381
62.748	0.00973	62.799	0.00381
62.749	0.00967	62.799	0.0038
62.750	0.00964	62.800	0.0038
62.750	0.00961	62.801	0.0038
62.751	0.00958	62.802	0.00379
62.751	0.00955	62.803	0.00379
62.752	0.00952	62.804	0.00378
62.752	0.00949	62.805	0.00378
62.753	0.00946	62.806	0.00377
62.754	0.00943	62.806	0.00377
62.754	0.00949	62.807	0.00376
62.755	0.00941	62.808	0.00376
62.755	0.00946	62.809	0.00376

62.756	0.00976	62.810	0.00375
62.756	0.00949	62.811	0.00375
62.757	0.00943	62.812	0.00374
62.758	0.00963	62.813	0.00374
62.758	0.00955	62.814	0.00373
62.759	0.0096	62.814	0.00373
62.759	0.00966	62.815	0.00372
62.760	0.00971	62.816	0.00371
62.761	0.00977	62.817	0.00371
62.761	0.00982	62.818	0.0037
62.762	0.00987	62.819	0.00369
62.762	0.00993	62.820	0.00368
62.763	0.00998	62.821	0.00368
62.763	0.01003	62.822	0.00367
62.764	0.01008	62.822	0.00367
62.765	0.01013	62.823	0.00367
62.765	0.01018	62.824	0.00366
62.766	0.01023	62.825	0.00366
62.766	0.01028	62.826	0.00365
62.767	0.01032	62.827	0.00364
62.768	0.01037	62.828	0.00364
62.768	0.01042	62.829	0.00363
62.769	0.01046	62.830	0.00362
62.769	0.0105	62.831	0.00362
62.770	0.01054	62.832	0.00361
62.771	0.01058	62.833	0.0036
62.771	0.01062	62.833	0.00359
62.772	0.01066	62.834	0.00358
62.772	0.0107	62.835	0.00357
62.773	0.01073	62.836	0.00356
62.774	0.01076	62.837	0.00354
62.774	0.0108	62.838	0.00353
62.775	0.01083	62.839	0.00352
62.775	0.01086	62.840	0.00351
62.776	0.01089	62.841	0.00351
62.777	0.01091	62.842	0.0035
62.777	0.01094	62.843	0.00349
62.778	0.01096	62.844	0.00348
62.778	0.01099	62.845	0.00348
62.779	0.01101	62.846	0.00347
62.780	0.01103	62.847	0.00346
62.780	0.01105	62.848	0.00346
62.781	0.01106	62.849	0.00346
62.781	0.01108	62.850	0.00346
62.782	0.01109	62.851	0.00346
62.783	0.0111	62.852	0.00346
62.783	0.01111	62.853	0.00346
62.784	0.01112	62.854	0.00346
62.785	0.01112	62.855	0.00346
62.785	0.01113	62.856	0.00347
62.786	0.01113	62.857	0.00348
62.786	0.01113	62.858	0.00351
62.787	0.01112	62.859	0.00354

62.788	0.01112	62.860	0.00356
62.788	0.01111	62.861	0.00358
62.789	0.0111	62.862	0.00361
62.789	0.01109	62.863	0.00363
62.790	0.01108	62.864	0.00365
62.791	0.01107	62.865	0.00367
62.791	0.01105	62.866	0.00369
62.792	0.01103	62.867	0.00371
62.793	0.01101	62.868	0.00373
62.793	0.01098	62.869	0.00375
62.794	0.01096	62.870	0.00377
62.794	0.01093	62.871	0.00378
62.795	0.0109	62.872	0.0038
62.796	0.01087	62.873	0.00382
62.796	0.01084	62.874	0.00383
62.797	0.0108	62.875	0.00385
62.798	0.01076	62.876	0.00386
62.798	0.01072	62.877	0.00389
62.799	0.01068	62.878	0.00395
62.799	0.01064	62.879	0.004
62.800	0.0106	62.880	0.00405
62.801	0.01055	62.882	0.0041
62.801	0.0105	62.883	0.00415
62.802	0.01045	62.884	0.00419
62.803	0.0104	62.885	0.00424
62.803	0.01035	62.886	0.00429
62.804	0.01029	62.887	0.00433
62.805	0.01024	62.888	0.00438
62.805	0.01018	62.889	0.00442
62.806	0.01021	62.890	0.00446
62.806	0.0101	62.891	0.0045
62.807	0.0101	62.892	0.00454
62.808	0.0101	62.894	0.00458
62.808	0.0101	62.895	0.00461
62.809	0.01009	62.896	0.00465
62.810	0.01009	62.897	0.00468
62.810	0.01008	62.898	0.00471
62.811	0.01007	62.899	0.00474
62.812	0.01006	62.900	0.00476
62.812	0.01005	62.901	0.00479
62.813	0.01004	62.903	0.00481
62.813	0.01002	62.904	0.00483
62.814	0.01001	62.905	0.00485
62.815	0.00999	62.906	0.00487
62.815	0.00997	62.907	0.00488
62.816	0.00995	62.908	0.00489
62.817	0.00993	62.909	0.00491
62.817	0.00991	62.911	0.00491
62.818	0.00989	62.912	0.00492
62.819	0.00986	62.913	0.00493
62.819	0.00984	62.914	0.00493
62.820	0.00981	62.915	0.00493
62.821	0.00978	62.916	0.00493

62.821	0.00975	62.918	0.00493
62.822	0.00972	62.919	0.00493
62.823	0.00969	62.920	0.00492
62.823	0.00965	62.921	0.00491
62.824	0.00964	62.922	0.0049
62.825	0.00958	62.924	0.00489
62.825	0.00955	62.925	0.00488
62.826	0.00951	62.926	0.00487
62.827	0.00947	62.927	0.00485
62.827	0.00943	62.929	0.00485
62.828	0.00939	62.930	0.00487
62.828	0.00935	62.931	0.00489
62.829	0.0093	62.932	0.00491
62.830	0.00926	62.933	0.00492
62.830	0.00921	62.935	0.00494
62.831	0.00917	62.936	0.00495
62.832	0.00912	62.937	0.00496
62.832	0.00907	62.938	0.00496
62.833	0.00902	62.940	0.00497
62.834	0.00907	62.941	0.00497
62.834	0.009	62.942	0.00497
62.835	0.00902	62.943	0.00497
62.836	0.00884	62.945	0.00496
62.836	0.00889	62.946	0.00495
62.837	0.00876	62.947	0.00494
62.838	0.00872	62.949	0.00493
62.838	0.00869	62.950	0.00491
62.839	0.00863	62.951	0.0049
62.840	0.00887	62.953	0.00487
62.841	0.00861	62.954	0.00485
62.841	0.00863	62.955	0.00482
62.842	0.00861	62.956	0.00479
62.843	0.00843	62.958	0.00476
62.843	0.00841	62.959	0.00473
62.844	0.00835	62.960	0.00469
62.845	0.00832	62.962	0.00465
62.845	0.00828	62.963	0.00461
62.846	0.00824	62.964	0.00456
62.847	0.00832	62.966	0.00451
62.847	0.00817	62.967	0.00447
62.848	0.00816	62.969	0.00441
62.849	0.00808	62.970	0.00436
62.849	0.00804	62.971	0.00431
62.850	0.008	62.973	0.00425
62.851	0.00798	62.974	0.00419
62.851	0.00791	62.975	0.00413
62.852	0.00789	62.977	0.00407
62.853	0.00782	62.978	0.00401
62.853	0.00777	62.980	0.00395
62.854	0.00773	62.981	0.0039
62.855	0.00768	62.982	0.00387
62.856	0.00763	62.984	0.00385
62.856	0.00759	62.985	0.00382

62.857	0.00758	62.987	0.00379
62.858	0.00756	62.988	0.00376
62.858	0.00755	62.989	0.00372
62.859	0.00753	62.991	0.00369
62.860	0.00751	62.992	0.00365
62.860	0.00749	62.994	0.00362
62.861	0.00747	62.995	0.00358
62.862	0.00745	62.997	0.00354
62.862	0.00743	62.998	0.0035
62.863	0.00741	62.999	0.00346
62.864	0.00739	63.001	0.00341
62.865	0.00737	63.002	0.00337
62.865	0.00734	63.004	0.00332
62.866	0.00732	63.005	0.00327
62.867	0.00729	63.007	0.00322
62.867	0.00726	63.008	0.00317
62.868	0.00724	63.010	0.00312
62.869	0.00722	63.011	0.00307
62.870	0.00719	63.013	0.00302
62.870	0.00717	63.014	0.00296
62.871	0.00714	63.016	0.0029
62.872	0.00711	63.017	0.00285
62.872	0.00709	63.019	0.00279
62.873	0.00706	63.020	0.00273
62.874	0.00703	63.022	0.00267
62.875	0.007	63.023	0.0026
62.875	0.00697	63.024	0.00254
62.876	0.00694	63.026	0.0025
62.877	0.0069	63.027	0.00246
62.877	0.00687	63.029	0.00242
62.878	0.00684	63.030	0.00237
62.879	0.0068	63.032	0.00233
62.880	0.00677	63.033	0.0023
62.880	0.00674	63.035	0.00227
62.881	0.00671	63.036	0.00224
62.882	0.0067	63.038	0.00221
62.882	0.00668	63.039	0.00218
62.883	0.00666	63.041	0.00214
62.884	0.00664	63.042	0.00211
62.885	0.00662	63.044	0.00208
62.885	0.0066	63.045	0.00204
62.886	0.00658	63.047	0.00201
62.887	0.00655	63.048	0.00197
62.888	0.00653	63.050	0.00193
62.888	0.0065	63.051	0.00189
62.889	0.00647	63.053	0.00186
62.890	0.00644	63.054	0.00182
62.891	0.00641	63.056	0.00178
62.891	0.00637	63.057	0.00173
62.892	0.00634	63.059	0.00169
62.893	0.0063	63.060	0.00165
62.893	0.00626	63.062	0.00161
62.894	0.00622	63.063	0.00156

62.895	0.00618	63.065	0.00152
62.896	0.00614	63.066	0.00147
62.896	0.0061	63.068	0.00143
62.897	0.00605	63.069	0.00138
62.898	0.006	63.071	0.00134
62.899	0.00596	63.072	0.00129
62.899	0.00591	63.074	0.00124
62.900	0.00586	63.075	0.00122
62.901	0.00583	63.077	0.0012
62.902	0.0058	63.078	0.00119
62.902	0.00578	63.080	0.00118
62.903	0.00576	63.081	0.00116
62.904	0.00573	63.083	0.00115
62.905	0.00571	63.084	0.00113
62.905	0.00568	63.086	0.00112
62.906	0.00566	63.087	0.0011
62.907	0.00563	63.089	0.00109
62.908	0.0056	63.090	0.00107
62.908	0.00557	63.092	0.00106
62.909	0.00555	63.093	0.00104
62.910	0.00552	63.095	0.00103
62.911	0.00549	63.096	0.00101
62.911	0.00546	63.098	0.001
62.912	0.00543	63.099	0.00098
62.913	0.0054	63.101	0.00097
62.914	0.00536	63.102	0.00095
62.914	0.00533	63.104	0.00093
62.915	0.0053	63.105	0.00092
62.916	0.00527	63.107	0.0009
62.917	0.00526	63.108	0.00089
62.918	0.00526	63.110	0.00087
62.918	0.00526	63.111	0.00085
62.919	0.00525	63.113	0.00084
62.920	0.00525	63.114	0.00082
62.921	0.00524	63.116	0.0008
62.921	0.00523	63.117	0.00079
62.922	0.00522	63.119	0.00077
62.923	0.00521	63.120	0.00076
62.924	0.0052	63.122	0.00074
62.924	0.00518	63.123	0.00073
62.925	0.00517	63.125	0.00071
62.926	0.00518	63.126	0.0007
62.927	0.00515	63.128	0.00068
62.927	0.00512	63.129	0.00067
62.928	0.0051	63.131	0.00065
62.929	0.00508	63.132	0.00064
62.930	0.00507	63.134	0.00062
62.930	0.00507	63.135	0.00061
62.931	0.00503	63.137	0.00059
62.932	0.00506	63.138	0.00058
62.933	0.00501	63.140	0.00057
62.933	0.005	63.141	0.00055
62.934	0.00501	63.143	0.00054

62.935	0.00501	63.144	0.00053
62.936	0.00502	63.146	0.00054
62.936	0.00504	63.147	0.00056
62.937	0.00503	63.149	0.00056
62.938	0.00504	63.150	0.00057
62.938	0.00505	63.152	0.00058
62.939	0.00506	63.153	0.00059
62.940	0.00506	63.155	0.0006
62.941	0.00507	63.156	0.00061
62.941	0.00508	63.158	0.00062
62.942	0.00508	63.159	0.00063
62.943	0.00509	63.161	0.00064
62.944	0.00509	63.162	0.00065
62.944	0.0051	63.164	0.00066
62.945	0.00511	63.165	0.00066
62.946	0.00511	63.167	0.00067
62.947	0.00512	63.168	0.00068
62.947	0.00512	63.170	0.0007
62.948	0.00513	63.171	0.00072
62.949	0.00513	63.173	0.00074
62.950	0.00514	63.174	0.00076
62.950	0.00514	63.176	0.00081
62.951	0.00514	63.177	0.0008
62.952	0.00515	63.179	0.00088
62.952	0.00515	63.181	0.00085
62.953	0.00515	63.182	0.00089
62.954	0.00516	63.184	0.00094
62.955	0.00516	63.185	0.00098
62.955	0.00516	63.187	0.00099
62.956	0.00516	63.188	0.00104
62.957	0.00516	63.190	0.00103
62.958	0.00517	63.191	0.00109
62.958	0.00517	63.193	0.00108
62.959	0.00517	63.194	0.00112
62.960	0.00517	63.196	0.00115
62.960	0.00517	63.197	0.00119
62.961	0.00516	63.199	0.00121
62.962	0.00516	63.200	0.00125
62.963	0.00516	63.202	0.00128
62.963	0.00516	63.203	0.00132
62.964	0.00516	63.205	0.00136
62.965	0.00515	63.206	0.00139
62.966	0.00515	63.208	0.00143
62.966	0.00515	63.209	0.00147
62.967	0.00514	63.211	0.0015
62.968	0.00514	63.212	0.00154
62.968	0.00513	63.214	0.00157
62.969	0.00513	63.215	0.0016
62.970	0.00512	63.217	0.00164
62.971	0.00512	63.218	0.00166
62.971	0.00511	63.220	0.00169
62.972	0.0051	63.221	0.00172
62.973	0.0051	63.223	0.00174

62.974	0.00509	63.225	0.00177
62.974	0.00508	63.226	0.00179
62.975	0.00507	63.228	0.00181
62.976	0.00506	63.229	0.00183
62.977	0.00506	63.231	0.00185
62.977	0.00505	63.232	0.00186
62.978	0.00504	63.234	0.00188
62.979	0.00503	63.235	0.00189
62.979	0.00502	63.237	0.0019
62.980	0.00501	63.238	0.00191
62.981	0.00499	63.240	0.00192
62.982	0.00498	63.241	0.00192
62.982	0.00497	63.243	0.00193
62.983	0.00496	63.244	0.00193
62.984	0.00495	63.246	0.00194
62.985	0.00493	63.247	0.00194
62.985	0.00492	63.249	0.00194
62.986	0.00491	63.250	0.00194
62.987	0.00489	63.252	0.00195
62.988	0.00488	63.253	0.00195
62.988	0.00487	63.255	0.00196
62.989	0.00485	63.256	0.00196
62.990	0.00484	63.258	0.00196
62.991	0.00482	63.260	0.00196
62.991	0.00481	63.261	0.00196
62.992	0.00479	63.263	0.00196
62.993	0.00478	63.264	0.00196
62.994	0.00476	63.266	0.00195
62.994	0.00475	63.267	0.00194
62.995	0.00473	63.269	0.00193
62.996	0.00472	63.270	0.00192
62.997	0.0047	63.272	0.00191
62.997	0.00468	63.273	0.0019
62.998	0.00467	63.275	0.00188
62.999	0.00465	63.276	0.00187
63.000	0.00463	63.278	0.00185
63.000	0.00462	63.279	0.00183
63.001	0.00461	63.281	0.00181
63.002	0.00461	63.282	0.00179
63.003	0.00461	63.284	0.00177
63.003	0.00461	63.285	0.00174
63.004	0.00462	63.287	0.00172
63.005	0.00462	63.289	0.00169
63.006	0.00462	63.290	0.00167
63.006	0.00462	63.292	0.00164
63.007	0.00462	63.293	0.00161
63.008	0.00462	63.295	0.00158
63.009	0.00462	63.296	0.00155
63.009	0.00462	63.298	0.00152
63.010	0.00462	63.299	0.00149
63.011	0.00461	63.301	0.00146
63.012	0.00461	63.302	0.00144
63.013	0.0046	63.304	0.00141

63.013	0.0046	63.305	0.00139
63.014	0.00459	63.307	0.00136
63.015	0.00459	63.308	0.00134
63.016	0.00458	63.310	0.00131
63.017	0.00457	63.312	0.00128
63.017	0.00456	63.313	0.00126
63.018	0.00455	63.315	0.00123
63.019	0.00454	63.316	0.0012
63.020	0.00453	63.318	0.00117
63.021	0.00452	63.319	0.00114
63.021	0.00451	63.321	0.00111
63.022	0.0045	63.322	0.00108
63.023	0.00449	63.324	0.00105
63.024	0.00448	63.325	0.00102
63.025	0.00448	63.327	0.001
63.025	0.00447	63.328	0.00096
63.026	0.00447	63.330	0.00094
63.027	0.00446	63.331	0.00091
63.028	0.00445	63.333	0.00087
63.029	0.00445	63.334	0.00084
63.029	0.00444	63.336	0.00084
63.030	0.00443	63.338	0.00079
63.031	0.00442	63.339	0.00077
63.032	0.00441	63.341	0.00074
63.033	0.0044	63.342	0.00071
63.034	0.00439	63.344	0.00069
63.034	0.00438	63.345	0.00066
63.035	0.00437	63.347	0.00064
63.036	0.00436	63.348	0.00062
63.037	0.00434	63.350	0.0006
63.038	0.00434	63.351	0.00058
63.039	0.00434	63.353	0.00057
63.039	0.00434	63.354	0.00055
63.040	0.00434	63.356	0.00053
63.041	0.00433	63.358	0.00051
63.042	0.00433	63.359	0.0005
63.043	0.00433	63.361	0.00048
63.044	0.00434	63.362	0.00047
63.045	0.00434	63.364	0.00045
63.045	0.00435	63.365	0.00044
63.046	0.00436	63.367	0.00042
63.047	0.00436	63.368	0.00041
63.048	0.00437	63.370	0.00039
63.049	0.00437	63.371	0.00037
63.050	0.00437	63.373	0.00036
63.051	0.00438	63.374	0.00034
63.051	0.00438	63.376	0.00032
63.052	0.00438	63.377	0.00031
63.053	0.00437	63.379	0.00029
63.054	0.00437	63.381	0.00027
63.055	0.00437	63.382	0.00026
63.056	0.00436	63.384	0.00024
63.057	0.00436	63.385	0.00022

63.058	0.00435	63.387	0.0002
63.058	0.00434	63.388	0.00019
63.059	0.00434	63.390	0.00017
63.060	0.00433	63.391	0.00015
63.061	0.00431	63.393	0.00013
63.062	0.0043	63.394	0.00011
63.063	0.00429	63.396	0.0001
63.064	0.00427	63.397	0.0001
63.065	0.00426	63.399	0.0001
63.066	0.00424	63.401	0.0001
63.067	0.00422	63.402	0.0001
63.067	0.0042	63.404	0.0001
63.068	0.00418	63.405	0.0001
63.069	0.00416	63.407	0.0001
63.070	0.00414	63.408	0.0001
63.071	0.00411	63.410	0.0001
63.072	0.00411	63.411	0.0001
63.073	0.00411	63.413	0.00012
63.074	0.0041	63.414	0.00015
63.075	0.0041	63.416	0.00017
63.076	0.00409	63.417	0.0002
63.077	0.00409	63.419	0.00023
63.078	0.00408	63.421	0.00025
63.079	0.00408	63.422	0.00028
63.079	0.00407	63.424	0.00031
63.080	0.00406	63.425	0.00033
63.081	0.00405	63.427	0.00035
63.082	0.00404	63.428	0.00038
63.083	0.00403	63.430	0.0004
63.084	0.00401	63.431	0.00042
63.085	0.004	63.433	0.00045
63.086	0.00399	63.434	0.00048
63.087	0.00397	63.436	0.0005
63.088	0.00396	63.438	0.00053
63.089	0.00396	63.439	0.00055
63.090	0.00395	63.441	0.00058
63.091	0.00395	63.442	0.0006
63.092	0.00396	63.444	0.00062
63.093	0.00398	63.445	0.00065
63.094	0.004	63.447	0.00067
63.095	0.00401	63.448	0.0007
63.096	0.00403	63.450	0.00073
63.097	0.00405	63.451	0.00076
63.098	0.00406	63.453	0.00078
63.099	0.00408	63.455	0.00081
63.100	0.00409	63.456	0.00084
63.101	0.0041	63.458	0.00086
63.102	0.00412	63.459	0.00089
63.103	0.00413	63.461	0.00091
63.104	0.00414	63.462	0.00094
63.105	0.00415	63.464	0.00096
63.106	0.00415	63.465	0.00099
63.107	0.00416	63.467	0.00101

63.108	0.00417	63.468	0.00104
63.109	0.00417	63.470	0.00107
63.110	0.00417	63.472	0.0011
63.111	0.00417	63.473	0.00112
63.112	0.00417	63.475	0.00115
63.113	0.00417	63.476	0.00118
63.114	0.00416	63.478	0.00121
63.115	0.00415	63.479	0.00123
63.116	0.00414	63.481	0.00126
63.117	0.00415	63.482	0.00129
63.118	0.00416	63.484	0.00131
63.119	0.00417	63.485	0.00134
63.120	0.00418	63.487	0.00136
63.121	0.00418	63.489	0.00139
63.123	0.00418	63.490	0.00141
63.124	0.00418	63.492	0.00144
63.125	0.00418	63.493	0.00146
63.126	0.00417	63.495	0.00148
63.127	0.00416	63.496	0.00151
63.128	0.00415	63.498	0.00153
63.129	0.00413	63.499	0.00155
63.130	0.00411	63.501	0.00157
63.131	0.00409	63.502	0.00159
63.132	0.00407	63.504	0.00161
63.134	0.00404	63.506	0.00164
63.135	0.00402	63.507	0.00167
63.136	0.00399	63.509	0.0017
63.137	0.00395	63.510	0.00173
63.138	0.00392	63.512	0.00175
63.139	0.00388	63.513	0.00178
63.141	0.00384	63.515	0.00181
63.142	0.0038	63.516	0.00184
63.143	0.00375	63.518	0.00186
63.144	0.00371	63.520	0.00189
63.145	0.00366	63.521	0.00191
63.147	0.00361	63.523	0.00193
63.148	0.00356	63.524	0.00196
63.149	0.0035	63.526	0.00198
63.150	0.00345	63.527	0.002
63.151	0.00339	63.529	0.00201
63.153	0.00333	63.530	0.00203
63.154	0.00327	63.532	0.00205
63.155	0.00321	63.533	0.00206
63.156	0.00315	63.535	0.00208
63.158	0.00309	63.537	0.00209
63.159	0.00302	63.538	0.0021
63.160	0.00296	63.540	0.00211
63.162	0.00289	63.541	0.00212
63.163	0.00282	63.543	0.00213
63.164	0.00275	63.544	0.00214
63.165	0.00268	63.546	0.00214
63.167	0.00261	63.547	0.00215
63.168	0.00257	63.549	0.00215

63.169	0.00254	63.551	0.00215
63.171	0.00253	63.552	0.00216
63.172	0.00252	63.554	0.00216
63.173	0.00251	63.555	0.00215
63.175	0.00249	63.557	0.00215
63.176	0.00248	63.558	0.00216
63.177	0.00246	63.560	0.00216
63.179	0.00244	63.561	0.00217
63.180	0.00242	63.563	0.00217
63.182	0.0024	63.565	0.00217
63.183	0.00237	63.566	0.00217
63.184	0.00235	63.568	0.00217
63.186	0.00232	63.569	0.00217
63.187	0.00228	63.571	0.00216
63.189	0.00225	63.572	0.00215
63.190	0.00223	63.574	0.00215
63.191	0.0022	63.575	0.00214
63.193	0.00218	63.577	0.00213
63.194	0.00215	63.579	0.00211
63.196	0.00211	63.580	0.0021
63.197	0.00208	63.582	0.00209
63.199	0.00204	63.583	0.00207
63.200	0.002	63.585	0.00205
63.201	0.00196	63.586	0.00203
63.203	0.00192	63.588	0.00201
63.204	0.0019	63.589	0.00199
63.206	0.00187	63.591	0.00196
63.207	0.00183	63.593	0.00194
63.209	0.0018	63.594	0.00191
63.210	0.00177	63.596	0.00189
63.211	0.00173	63.597	0.00186
63.213	0.0017	63.599	0.00183
63.214	0.00167	63.600	0.0018
63.216	0.00165	63.602	0.00176
63.217	0.00163	63.603	0.00173
63.219	0.0016	63.605	0.0017
63.220	0.00158	63.607	0.00166
63.222	0.00155	63.608	0.00163
63.223	0.00153	63.610	0.0016
63.224	0.00151	63.611	0.00157
63.226	0.00149	63.613	0.00154
63.227	0.00148	63.614	0.00151
63.229	0.00147	63.616	0.00148
63.230	0.00146	63.617	0.00145
63.232	0.00146	63.619	0.00141
63.233	0.00145	63.621	0.00138
63.234	0.00144	63.622	0.00135
63.236	0.00144	63.624	0.00131
63.237	0.00144	63.625	0.00128
63.239	0.00146	63.627	0.00124
63.240	0.00148	63.628	0.00121
63.242	0.00149	63.630	0.00117
63.243	0.0015	63.632	0.00113

63.244	0.00151	63.633	0.0011
63.246	0.00151	63.635	0.00106
63.247	0.00151	63.636	0.00102
63.249	0.00151	63.638	0.00098
63.250	0.0015	63.639	0.00094
63.252	0.0015	63.641	0.0009
63.253	0.00148	63.642	0.00086
63.254	0.00147	63.644	0.00083
63.256	0.00145	63.646	0.00079
63.257	0.00143	63.647	0.00075
63.259	0.00141	63.649	0.00072
63.260	0.00139	63.650	0.00068
63.262	0.00136	63.652	0.00065
63.263	0.00136	63.653	0.00063
63.265	0.00136	63.655	0.00061
63.266	0.00136	63.656	0.00058
63.267	0.00135	63.658	0.00056
63.269	0.00135	63.660	0.00054
63.270	0.00134	63.661	0.00052
63.272	0.00134	63.663	0.0005
63.273	0.00133	63.664	0.00048
63.275	0.00134	63.666	0.00046
63.276	0.00136	63.667	0.00044
63.277	0.00139	63.669	0.00042
63.279	0.00141	63.671	0.00041
63.280	0.00143	63.672	0.00039
63.282	0.00145	63.674	0.00037
63.283	0.00146	63.675	0.00036
63.285	0.00148	63.677	0.00035
63.286	0.00149	63.678	0.00034
63.288	0.0015	63.680	0.00033
63.289	0.0015	63.681	0.00031
63.290	0.00151	63.683	0.0003
63.292	0.00151	63.685	0.00029
63.293	0.00151	63.686	0.00027
63.295	0.00151	63.688	0.00026
63.296	0.00151	63.689	0.00024
63.298	0.0015	63.691	0.00023
63.299	0.00149	63.692	0.00021
63.300	0.00148	63.694	0.0002
63.302	0.00147	63.696	0.00018
63.303	0.00146	63.697	0.00016
63.305	0.00144	63.699	0.00015
63.306	0.00143	63.700	0.00017
63.308	0.00141	63.702	0.00018
63.309	0.00139		
63.311	0.00137		
63.312	0.00135		
63.313	0.00132		
63.315	0.0013		
63.316	0.00128		
63.318	0.00125		
63.319	0.00123		

63.321	0.00125
63.322	0.00127
63.323	0.0013
63.325	0.00133
63.326	0.00135
63.328	0.00137
63.329	0.00139
63.331	0.00141
63.332	0.00142
63.334	0.00144
63.335	0.00145
63.336	0.00146
63.338	0.00146
63.339	0.00146
63.341	0.00146
63.342	0.00146
63.344	0.00145
63.345	0.00144
63.347	0.00143
63.348	0.00142
63.349	0.0014
63.351	0.00138
63.352	0.00136
63.354	0.00133
63.355	0.00131
63.357	0.00128
63.358	0.00125
63.360	0.00122
63.361	0.00121
63.362	0.00121
63.364	0.0012
63.365	0.00119
63.367	0.00119
63.368	0.00118
63.370	0.00118
63.371	0.00118
63.373	0.00119
63.374	0.00119
63.375	0.00119
63.377	0.00119
63.378	0.0012
63.380	0.00121
63.381	0.00121
63.383	0.00122
63.384	0.00122
63.386	0.00123
63.387	0.00123
63.389	0.00123
63.390	0.00123
63.391	0.00123
63.393	0.00122
63.394	0.00122
63.396	0.00122

63.397	0.00122
63.399	0.00122
63.400	0.00121
63.402	0.0012
63.403	0.00119
63.404	0.00118
63.406	0.00117
63.407	0.00116
63.409	0.00114
63.410	0.00112
63.412	0.00111
63.413	0.00109
63.415	0.00107
63.416	0.00105
63.418	0.00104
63.419	0.00102
63.420	0.00101
63.422	0.001
63.423	0.00102
63.425	0.00106
63.426	0.00106
63.428	0.00108
63.429	0.00111
63.431	0.00114
63.432	0.00116
63.434	0.00118
63.435	0.00121
63.436	0.00122
63.438	0.00124
63.439	0.00125
63.441	0.00127
63.442	0.00128
63.444	0.00128
63.445	0.00129
63.447	0.00129
63.448	0.00128
63.450	0.00128
63.451	0.00127
63.452	0.00126
63.454	0.00124
63.455	0.00123
63.457	0.00121
63.458	0.00118
63.460	0.00116
63.461	0.00113
63.463	0.0011
63.464	0.00106
63.466	0.00103
63.467	0.00099
63.468	0.00095
63.470	0.0009
63.471	0.00086
63.473	0.00081

63.474	0.00077
63.476	0.00075
63.477	0.00073
63.479	0.00072
63.480	0.0007
63.482	0.00068
63.483	0.00065
63.485	0.00063
63.486	0.0006
63.487	0.00057
63.489	0.00054
63.490	0.00051
63.492	0.00048
63.493	0.00044
63.495	0.00041
63.496	0.00037
63.498	0.00033
63.499	0.00029
63.501	0.00025
63.502	0.00023
63.504	0.00025
63.505	0.00026
63.507	0.00027
63.508	0.00028
63.509	0.00029
63.511	0.0003
63.512	0.0003
63.514	0.00031
63.515	0.00031
63.517	0.00032
63.518	0.00032
63.520	0.00033
63.521	0.00033
63.523	0.00034
63.524	0.00034
63.526	0.00035
63.527	0.00036
63.529	0.00037
63.530	0.00038
63.531	0.00039
63.533	0.00041
63.534	0.00043
63.536	0.00045
63.537	0.00047
63.539	0.00048
63.540	0.0005
63.542	0.00051
63.543	0.00053
63.545	0.00055
63.546	0.00056
63.548	0.00058
63.549	0.00059
63.551	0.0006

63.552	0.00062
63.554	0.00063
63.555	0.00064
63.556	0.00065
63.558	0.00067
63.559	0.00068
63.561	0.0007
63.562	0.00071
63.564	0.00073
63.565	0.00074
63.567	0.00075
63.568	0.00076
63.570	0.00077
63.571	0.00078
63.573	0.00079
63.574	0.0008
63.576	0.0008
63.577	0.00081
63.579	0.00082
63.580	0.00082
63.582	0.00083
63.583	0.00083
63.584	0.00085
63.586	0.00087
63.587	0.00088
63.589	0.0009
63.590	0.00092
63.592	0.00093
63.593	0.00094
63.595	0.00096
63.596	0.00097
63.598	0.00098
63.599	0.00099
63.601	0.001
63.602	0.00101
63.604	0.00102
63.605	0.00103
63.607	0.00103
63.608	0.00104
63.610	0.00104
63.611	0.00104
63.613	0.00105
63.614	0.00105
63.615	0.00105
63.617	0.00105
63.618	0.00104
63.620	0.00104
63.621	0.00104
63.623	0.00104
63.624	0.00103
63.626	0.00103
63.627	0.00102
63.629	0.00102

63.630	0.00101
63.632	0.00101
63.633	0.001
63.635	0.001
63.636	0.001
63.638	0.00099
63.639	0.001
63.641	0.00101
63.642	0.00101
63.644	0.00102
63.645	0.00103
63.647	0.00102
63.648	0.00103
63.650	0.00103
63.651	0.00103
63.652	0.00104
63.654	0.00104
63.655	0.00104
63.657	0.00104
63.658	0.00103
63.660	0.00103
63.661	0.00103
63.663	0.00103
63.664	0.00102
63.666	0.00102
63.667	0.00101
63.669	0.001
63.670	0.001
63.672	0.00099
63.673	0.00098
63.675	0.00097
63.676	0.00096
63.678	0.00095
63.679	0.00094
63.681	0.00092
63.682	0.00091
63.684	0.0009
63.685	0.00089
63.687	0.00088
63.688	0.00088
63.690	0.00088
63.691	0.00087
63.693	0.00087
63.694	0.00087
63.696	0.00086
63.697	0.00086
63.698	0.00085
63.700	0.00084
63.701	0.00084
63.703	0.00083
63.704	0.00082
63.706	0.00081
63.707	0.0008

63.709	0.0008
63.710	0.00079
63.712	0.00078
63.713	0.00076
63.715	0.00075
63.716	0.00074
63.718	0.00073
63.719	0.00072
63.721	0.00071
63.722	0.0007
63.724	0.00069
63.725	0.00067
63.727	0.00066
63.728	0.00065
63.730	0.00064
63.731	0.00063
63.733	0.00062
63.734	0.0006
63.736	0.00059
63.737	0.00058
63.739	0.00058
63.740	0.00056
63.742	0.00055
63.743	0.00054
63.745	0.00054
63.746	0.00054
63.748	0.00053
63.749	0.00053
63.751	0.00052
63.752	0.00052
63.754	0.00051
63.755	0.0005
63.757	0.0005
63.758	0.00049
63.760	0.00048
63.761	0.00047
63.763	0.00046
63.764	0.00045
63.766	0.00044
63.767	0.00043
63.769	0.00042
63.770	0.00041
63.772	0.00039
63.773	0.00038
63.775	0.00036
63.776	0.00035
63.778	0.00033
63.779	0.00031
63.781	0.00029
63.782	0.00027
63.783	0.00025
63.785	0.00023
63.786	0.00021

63.788	0.00019
63.789	0.00017
63.791	0.00015
63.792	0.00014
63.794	0.00012
63.795	0.0001
63.797	0.0001
63.798	0.0001
63.800	0.00011
63.801	0.00014
63.803	0.00016
63.804	0.00018
63.806	0.0002
63.807	0.00022
63.809	0.00024
63.810	0.00026
63.812	0.00028
63.813	0.00029
63.815	0.0003
63.816	0.00032
63.818	0.00033
63.819	0.00034
63.821	0.00035
63.822	0.00035
63.824	0.00036
63.825	0.00037
63.827	0.00037
63.828	0.00037
63.830	0.00038
63.831	0.00038
63.833	0.00038
63.834	0.00039
63.836	0.0004
63.837	0.00041
63.839	0.00043
63.840	0.00044
63.842	0.00045
63.843	0.00046
63.845	0.00047
63.846	0.00047
63.848	0.00049
63.849	0.00049
63.851	0.0005
63.852	0.0005
63.854	0.00053
63.855	0.00054
63.857	0.00052
63.858	0.00053
63.860	0.00053
63.862	0.00053
63.863	0.00054
63.865	0.00054
63.866	0.00055

63.868	0.00059
63.869	0.00056
63.871	0.00056
63.872	0.00056
63.874	0.00057
63.875	0.00057
63.877	0.00058
63.878	0.00061
63.880	0.00058
63.881	0.00059
63.883	0.00059
63.884	0.00059
63.886	0.00061
63.887	0.00059
63.889	0.0006
63.890	0.00061
63.892	0.00062
63.893	0.00063
63.895	0.00063
63.896	0.00064
63.898	0.00064
63.899	0.00065
63.901	0.00067
63.902	0.00068
63.904	0.00069
63.905	0.0007
63.907	0.0007
63.908	0.00071
63.910	0.00072
63.911	0.00073
63.913	0.00073
63.914	0.00074
63.916	0.00074
63.917	0.00075
63.919	0.00075
63.920	0.00075
63.922	0.00076
63.923	0.00076
63.925	0.00076
63.926	0.00076
63.928	0.00076
63.929	0.00076
63.931	0.00076
63.932	0.00076
63.934	0.00075
63.935	0.00075
63.937	0.00076
63.938	0.00076
63.940	0.00077
63.942	0.00077
63.943	0.00078
63.945	0.00078
63.946	0.00079

63.948	0.00079
63.949	0.00079
63.951	0.00079
63.952	0.0008
63.954	0.0008
63.955	0.0008
63.957	0.0008
63.958	0.0008
63.960	0.00079
63.961	0.00079
63.963	0.00079
63.964	0.00078
63.966	0.00078
63.967	0.00078
63.969	0.00077
63.970	0.00076
63.972	0.00075
63.973	0.00075
63.975	0.00074
63.976	0.00073
63.978	0.00072
63.979	0.00072
63.981	0.0007
63.982	0.0007
63.984	0.00068
63.985	0.00067
63.987	0.00066
63.989	0.00068
63.990	0.00066
63.992	0.00065
63.993	0.00065
63.995	0.00065
63.996	0.00065
63.998	0.00064
63.999	0.00064
64.001	0.00063
64.002	0.00063
64.004	0.00063
64.005	0.00062
64.007	0.00061
64.008	0.00061
64.010	0.0006
64.011	0.00059
64.013	0.00058
64.014	0.00058
64.016	0.00057
64.017	0.00056
64.019	0.00056
64.020	0.00054
64.022	0.00053
64.023	0.00053
64.025	0.00051
64.027	0.00051

64.028	0.00048
64.030	0.00047
64.031	0.00047
64.033	0.00046
64.034	0.00045
64.036	0.00043
64.037	0.00042
64.039	0.00041
64.040	0.00041
64.042	0.0004
64.043	0.00041
64.045	0.00038
