# Supplementary material for: Gas explosion characteristics and spray control mechanism in underground square
Source: PLoS One. 2024 Apr 24;19(4):e0293421. doi: 10.1371/journal.pone.0293421 (PMC11042696; doi:10.1371/journal.pone.0293421)
Supplement: Attachment — Submitted filename: Response to Reviewers.docx [file pone.0293421.s005.docx]

Modification description

Dear editors and peer reviewers：

First of all, thank you very much for your comments and guidance on the paper 'Gas explosion characteristics and spray control mechanism in underground square'. It has played a great role in promoting the quality of the paper. According to the revision opinions of the reviewers, we have made an overall revision of the paper and marked it accordingly. The specific modifications are as follows :

**1.Please ensure that your manuscript meets PLOS ONE's style requirements, including those for file naming.**

**revision explanation:**We modified the format of the paper with reference to style templates.

**2.Please note that PLOS ONE has specific guidelines on code sharing for submissions in which author-generated code underpins the findings in the manuscript. In these cases, all author-generated code must be made available without restrictions upon publication of the work.**

**revision explanation:**We have modified the paper according to the requirements of the journal.

**3. Thank you for stating the following financial disclosure:**

**“This research was financially supported by National Natural Science Foundation of China (51974149).”**

**Please state what role the funders took in the study.  If the funders had no role, please state: "The funders had no role in study design, data collection and analysis, decision to publish, or preparation of the manuscript."**

**If this statement is not correct you must amend it as needed.**

**Please include this amended Role of Funder statement in your cover letter; we will change the online submission form on your behalf.**

**revision explanation:**We have modified the funding instructions according to the the journal requirements.

**4. Thank you for stating the following in the Acknowledgments Section of your manuscript:**

**“Funding: this work was supported by the National Natural Science Foundation of China (51974149).**

**Thank you very much for the service MJEditor (www.mjeditor.com) for its linguistic assistance during the preparation of this manuscript.”**

**We note that you have provided funding information that is currently declared in your Funding Statement. However, funding information should not appear in the Acknowledgments section or other areas of your manuscript. We will only publish** **funding information present in the Funding Statement section of the online submission form.**

**revision explanation:**We deleted the relevant description of the funding information according to the journal requirements.

5. **PLOS requires an ORCID iD for the corresponding author in Editorial Manager on papers submitted after December 6th, 2016. Please ensure that you have an ORCID iD and that it is validated in Editorial Manager. To do this, go to ‘Update my Information’ (in the upper left-hand corner of the main menu), and click on the Fetch/Validate link next to the ORCID field. This will take you to the ORCID site and allow you to create a new iD or authenticate a pre-existing iD in Editorial Manager. Please see the following video for instructions on linking an ORCID iD to your Editorial Manager account: <https://www.youtube.com/watch?v=_xcclfuvtxQ>**

**revision explanation:**We have verified ORCID according to journal requirements.

**Reviewer #1:**

**1.A comprehensive set of governing equations should be written for the present simulation, along with any constitutive equation that has been used in modeling.**

**revision explanation:**We modified and improved the control equation according to your suggestion, and marked it with green highlight.

**2. All the boundary conditions corresponding to the governing eqs. Should be described in the manuscript.**

**revision explanation：**The boundary conditions we set in this paper are marked with green highlights.

**3.Explain the criterion to choose the ignition source in the simulation.**

**revision explanation：**We have explained the reasons for the selection of the ignition position in Section 1.2 of the article, and marked it with green highlights.

**4.The explanations and interpretation of results based on diffusion is wrong in this case, as the pressure wave creates induced velocity, which lead to considerable effects of convection.**

**revision explanation：**Thank you very much for your guidance. With the increase in spray pressure, the diameter of the water mist produced by the spray system decreases, and the water mist is more likely to evaporate. The smaller the relative velocity with the overpressure shock wave is, the less the hindrance to the shock wave is. However, when the spray pressure exceeds the critical value ( 0.6MPa ), the small-sized water mist is more likely to deform, break, atomize, and disperse under the action of the explosion shock wave, which plays a leading role in the disturbance of turbulence. Therefore, the peak value of explosion overpressure increases gradually.

**5.The English language needs substantial improvement.**

**revision explanation：**Thank you for your comments and suggestions. We have checked and improved the grammar and expression of the article.

**Reviewer #2:**

**Abstract
1. Include brief intro or challenges faced before going into the objectives**

**revision explanation：**We describe the problems we face before entering the topic and highlight them with green.

**2.Put it in paragraph forms instead of point form**

**revision explanation：**We replace the point description with paragraph form and highlight it with green.

**3.State the methodology clearer**

**revision explanation：**We explained the research method more clearly and highlighted it with green.

**Introduction
1.** **Any work done before include the water spray effect by modify the turbulence model as discussed in equations 3-7?**

**revision explanation：**The change of parameters in equations (7) - (11) can change the turbulent combustion rate to reflect the double-sided effect of the water spray system on the explosion scene.

**Result
Section 3
1. Does th**e **explosion study here contain water spray?**

**revision explanation：**This part mainly simulates and analyzes the spatial and temporal distribution characteristics of gas explosion overpressure in an underground square under the condition of an unopened spray system.

2.**Explain why the flame at X=74 ignition point move to X=60m at overpressure stage as shown in Fig 5**.

**revision explanation：**In the overpressure attenuation stage, the flame shape shows an overall attenuation trend, but due to the redistribution of gas and sufficient oxygen at the exit of the ladder, the combustion phenomenon of gas near X = 60m is obvious.

**Section 4
1. Please indicate the axis in Fig 6 properly for explosion and spray pressure.**

**revision explanation：**We have re-marked the explosion overpressure and spray pressure in Fig.6 explained them in the text, and marked them with green highlights.

**2.The statement:****“ The peak arrival time (t0–t3), the duration of the explosion phase (ti–ti+1), and the overall duration of the explosion (t0–tfin) shortened with the increase in spray pressure, ….” Is not clear. The Fig 6 graph looks similar**

**revision explanation：**We modified the expression ' The peak arrival time (t_0_-t_3_), the duration of the explosion phase (t_i_-t_i+1_), and the overall duration of the explosion (t_0_-t_fin_) shortened with the increase in spray pressure,... '. And marked with a green highlight. Fig.6 mainly reflects the influence of different spray pressures on the explosion overpressure from the whole.

**3.Explain how the disturbance of water mist help. You may consider to include velocity vector or other additional info.**

**revision explanation：**In this paper, the influence of the initial velocity of water mist is marked with green highlight when explaining the interference effect of water mist on explosion shock wave.

**4.Put in axis indicator for Figure 7**

**revision explanation：**The abscissa axis has been marked as different spray pressures, and the ordinate axis is the overpressure peak and the overpressure rate, respectively.

**5. Is the Fig 7 including the time effect? Suggest to change the line to other indication method if no.**

**revision explanation：**Fig.7 mainly reflects the influence of different spray pressures on gas explosion from two aspects: explosion overpressure peak and explosion overpressure propagation rate.

**Conclusion
1. Put them in paragraph form and summarise it**

**revision explanation：**We write the main conclusions of the article in the form of paragraphs and highlight them in green.

2. **Conclusion 2 is not well highlighted in text before, maybe can consider explaining them a little**

**revision explanation：**According to your proposed amendments, we have added an explanation and description of Conclusion 2 in the previous article and marked it with green highlights.

Finally, I wish you editors and reviewers a pleasant job, and all the best.
