# Supplementary material for: Gas explosion characteristics and spray control mechanism in underground square
Source: PLoS One. 2024 Apr 24;19(4):e0293421. doi: 10.1371/journal.pone.0293421 (PMC11042696; doi:10.1371/journal.pone.0293421)
Supplement: Attachment — Submitted filename: Response to Reviewers.docx [file pone.0293421.s006.docx]

Modification description

Dear editors and peer reviewers：

First of all, thank you very much for your comments and guidance on the paper 'Gas explosion characteristics and spray control mechanism in underground square'. It has played a great role in promoting the quality of the paper. According to the revision opinions of the reviewers, we have made an overall revision of the paper and marked it accordingly. The specific modifications are as follows :

1. **Journal Requirements:**

**Please review your reference list to ensure that it is complete and correct. If you have cited papers that have been retracted, please include the rationale for doing so in the manuscript text, or remove these references and replace them with relevant current references. Any changes to the reference list should be mentioned in the rebuttal letter that accompanies your revised manuscript. If you need to cite a retracted article, indicate the article’s retracted status in the References list and also include a citation and full reference for the retraction notice.**

**revision explanation:** Thank you for your comments and suggestions. We have completed the inspection of references.

**2.The authors are suggested to mention the condition of ‘unopened’ spray system in the abstract and explained in detail in Section 2. The equations need to be rechecked. In first equation, the first term is density not pressure, the description of ‘f’ in equation 2 should be provided. Any turbulence model used has to be written.**

**revision explanation:** Thank you very much for your guidance. We have mentioned the conditions of the 'unopened' spray system in abstract , which are explained in the text and highlight in red. We also check and modify the equations in the text and highlight them in red.

**3.** **Consider to advise the author to include the statement "This part mainly simulates and analyzes the spatial and temporal distribution characteristics of gas explosion overpressure in an underground square under the condition of an unopened spray system." in the abstract and maybe introduction and maybe title to make it clear that the condition is for unopened spray system to avoid confusion.**

**revision explanation:** Thank you very much for your guidance. In order to avoid confusion, we have added the condition that the spray system is not turned on in the abstract and text, and highlight it in red.

Finally, I wish you editors and reviewers a pleasant job, and all the best.
